# Spatial and temporal analysis of extreme sea level and storm surge events around the coastline of the UK

**DOI:** 10.1038/sdata.2016.107

**Published:** 2016-12-06

**Authors:** Ivan D. Haigh, Matthew P. Wadey, Thomas Wahl, Ozgun Ozsoy, Robert J. Nicholls, Jennifer M. Brown, Kevin Horsburgh, Ben Gouldby

**Affiliations:** 1 Ocean and Earth Science, National Oceanography Centre, University of Southampton, European Way, Southampton SO14 3ZH, UK; 2 Faculty of Engineering and the Environment, University of Southampton, Southampton SO17 1BJ, UK; 3 Department of Civil, Environmental, and Construction Engineering and Sustainable Coastal Systems Cluster, University of Central Florida, 12800 Pegasus Drive, Suite 211, Orlando, Florida 32816-2450, USA; 4 National Oceanography Centre, Joseph Proudman Building, 6 Brownlow Street, Liverpool L3 5DA, UK; 5 Flood Management Group, HR Wallingford, Howbery Park, Wallingford, Oxfordshire OX10 8BA, UK

**Keywords:** Environmental sciences, Civil engineering, Physical oceanography

## Abstract

In this paper we analyse the spatial footprint and temporal clustering of extreme sea level and skew surge events around the UK coast over the last 100 years (1915–2014). The vast majority of the extreme sea level events are generated by moderate, rather than extreme skew surges, combined with spring astronomical high tides. We distinguish four broad categories of spatial footprints of events and the distinct storm tracks that generated them. There have been rare events when extreme levels have occurred along two unconnected coastal regions during the same storm. The events that occur in closest succession (<4 days) typically impact different stretches of coastline. The spring/neap tidal cycle prevents successive extreme sea level events from happening within 4–8 days. Finally, the 2013/14 season was highly unusual in the context of the last 100 years from an extreme sea level perspective.

## Introduction

Coastal floods are a major global hazard leading to long-lasting and wide-ranging social, economic and environmental consequences. In 2005, New Orleans was flooded by Hurricane Katrina, resulting in 800 deaths and $40 billion in damages^1–3^. In 2008, Cyclone Nargis swept seawater 50 km inland in the Irrawaddy Delta, killing 130,000 people^4^. In 2012, Hurricane Sandy caused wide-spread flooding around New York on the US east coast resulting in $71 billion in damages^5^. In 2013, Typhoon Haiyan impacted the Philippines killing 8,000 people and destroyed one million homes, much of the damage due to extreme sea levels^6^. Recently in 2016, Hurricane Matthew caused considerable loss of life and destruction along the coasts of the Caribbean and southeastern US. These events demonstrate the ever-present threat of serious coastal flood impacts despite improved technology and experience, which has provided tools to forecast and mitigate flooding risks. Furthermore, with sea-level rise accelerating^7^, and possible changes in storminess^8^ and tides^9^, high sea levels will occur more frequently in the future^10–14^. Without upgrades to flood protection and other appropriate adaption measures, this will significantly impact the growing populations and cities around the world’s coasts^15^. Continuing to improve the understanding of extreme sea level and coastal flood events is therefore of utmost importance.

Over the winter of 2013/14 the UK experienced a remarkable sequence of extreme storms and coastal floods^16–20^. These events caused an estimated £2.5 billion in damages, but much greater destruction was prevented due to the effectiveness of defences^21^. What appears noteworthy about this period is: (1) the large spatial ‘footprint’ of some of the events (i.e., simultaneous flooding along extended coastline stretches during the same storm); and (2) the temporal ‘clustering’ of the flooding events (i.e., events occurring one after another in close succession)^22^. These two issues have important financial and practical implications for the risk management sector, such as flood management, re-insurance, infrastructure reliability and emergency response; as impact and losses may be spatially and temporally correlated. For example, if multiple ports were damaged during the same storm this would affect national and even international supply chains^23,24^. Temporal clustering of extreme sea levels could lead to amplified flood damages due to attritional effects on defences and inadequate recovery/repair time of natural (e.g., beaches) and human elements within the flood protection system^25,26^. These are particularly important issues for the UK’s long^27^ and highly developed coastline and other densely populated coastlines around the world. Recognition and analysis of spatial and temporal extreme sea level characteristics and associated coastal flooding is, however, lacking. Spatial footprints and temporal clustering have started to be examined for other natural hazards^28–32^ but there has been limited assessment with regard to extreme sea levels.

**Figure 1:**
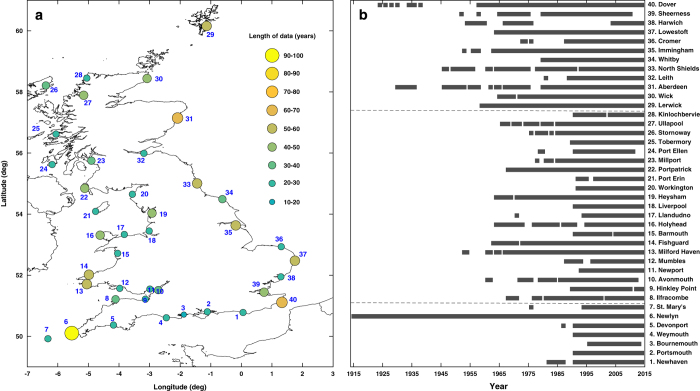
Tide gauge sites and duration. (**a**) Location of tide gauge sites around the UK, with site number; and (**b**) duration of the sea level records.

Here we undertake a detailed spatial and temporal analysis of extreme sea level events around the UK coast using the SurgeWatch database (Data Citation 1) compiled by Haigh *et al.*^33^. This database provides a systematic UK-wide record of extreme sea level events over the last 100 years (1915–2014) from tide gauge data at 40 sites (Fig. 1, Supplementary Table 1). We investigate two types of events: (1) extreme sea level events (relevant to coastal flooding) that reached or exceeded the 1 in 5 year return level as estimated by McMillian *et al.*^34^ and (2) extreme skew surges that reached or exceeded the 1 in 5 year return level that we estimate (see Methods). Some of the skew surge events coincide with the extreme sea level events, when the storm surge occurred around the time of high water of a spring tide; others do not coincide, because the surge occurred near low tide or on a neap tide. We chose the 1 in 5 year threshold, because it gave a manageable number of extreme sea level (96) and skew surge (111) events for analysis. In addition, tides reach a maximum every 4.4 years due to the lunar perigean cycle^35^ and we wanted to ensure that the extreme sea level events identified arose as consequence of a storm surge and not just a large astronomical tide.

## Results

### Comparison of events

**Figure 2:**
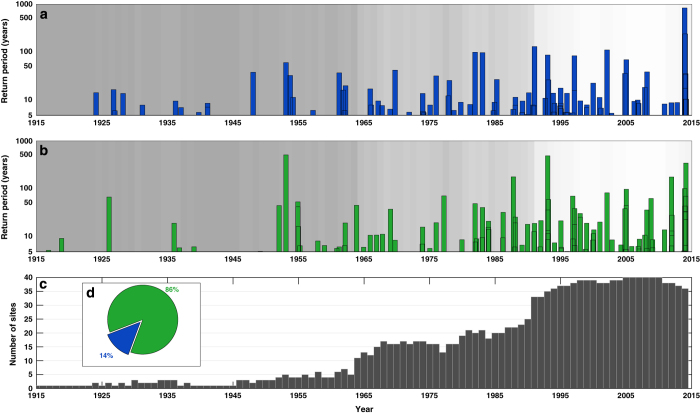
Extreme sea level and skew surge events. (**a**) Return period of the highest sea levels in each of the 96 sea level events, offset for mean sea level; (**b**) return period of the highest skew surges in each of the 111 skew surge events; (**c**) the number of sites per annum for which sea level data is available across the 40 sites; and (**d**) pie chart showing the number of skew surge events that led to sea level events (blue) and the number that did not (green). The grey shading in (**a**,**b**) indicates the number of sites for which data is available for each year—the lighter the grey the more sites for which data is available.

First we compare the dates and return periods of the two types of extreme events and ascertain how many skew surge events resulted in extreme sea levels. 310 high waters reached or exceeded the 1 in 5 year return level across the 40 study sites (Fig. 1). These were generated by 96 distinct storms (Fig. 2a, Supplementary Table 2). In total, 261 skew surges reached or exceeded the 1 in 5 year return period across the 40 sites, generated by 111 distinct storms (Fig. 2b, Supplementary Table 3).

As discussed in Haigh *et al.*^33^, it is important to point out there are unavoidable issues with the database that arise because tide gauge records do not all cover the full 100-year period analysed (i.e., since the start of the Newlyn record in 1915). It is obvious examining Fig. 2c that we are likely missing events before 1990, and particularly before 1965, when records were spatially more sparse. It is also apparent that the highest return period (across sites—shown in Fig. 2a,b) of several events is lower than it should be. Although we have data at some sites for these events, tide gauges were not necessarily operational at the time along the stretches of the coastline where the sea levels or skew surges were likely to have been most extreme; this is the case for the benchmark 1953 event^21^. Notwithstanding these issues, our analysis of the events that we have on record does provide important new insights, as described below.

Interestingly, only 15 of the 111 (14%) extreme skew surge events led to extreme sea levels (Fig. 2d), while the vast majority (86%) did not. Hence, most extreme sea levels arose from moderate (i.e., <1 in 5 year return levels) skew surge events combined with astronomical spring high tides.

### Spatial analysis

Second we assess the spatial characteristics of the events around the coast. We use the number of sites where the 1 in 5 year return period was reached or exceeded during an event, as a proxy for the distance of coastline affected (i.e., the footprint) (Supplementary Fig. 1a,b, Supplementary Tables 2 and 3, column 8). This is a reasonable assumption as the tide gauges are located at approximately evenly spaced distances (Fig. 1a). Because the data is sparse in the early part of the period, the footprint (i.e., number of sites impacted) of larger events are inevitably underestimated.

As expected, there is a significant (95% confidence level) correlation (0.52 for the sea level events and 0.38 for the skew surge event) between the highest return period of the events and the number of sites impacted (Supplementary Fig. 1c,d). The footprints of the skew surge events are consistently smaller than the footprints of extreme sea level events.

**Figure 3:**
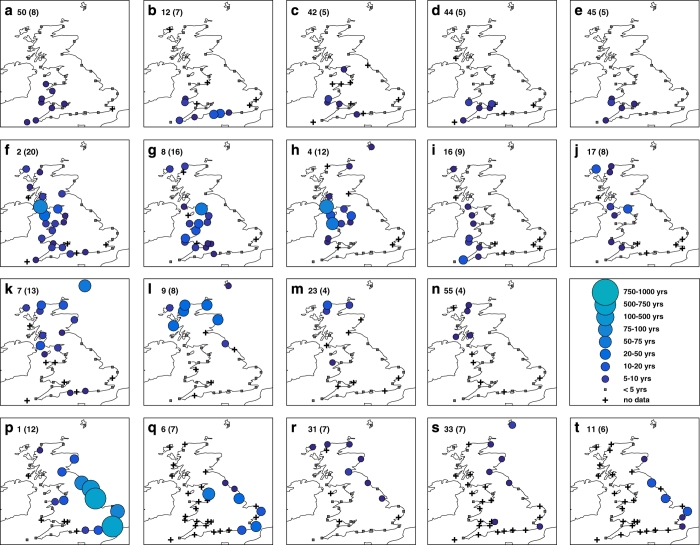
Spatial footprints of extreme sea level events. The spatial footprints of all the sea level events that impacted at least four tide gauge sites. The first number in the top left hand corner indicates the event ranking and the number in the bracket is the number of sites where the 1 in 5 year return level was reached or exceeded. The category one (**a**–**e**), two (**f**–**j**), three (**k**–**o**) and four (**p**–**t**) footprint events are shown in the first, second, third and fourth row of panels, respectively.

Next we examine the 19 sea level events that each impacted at least four sites, to assess the different spatial extents of coastline affected (Fig. 3). We find four broad categories of footprints (with some overlap). Category one events (Fig. 3a–e) cover the southwest coast, mainly including sites between Portsmouth and Fishguard. Category two events (Fig. 3f–j) cover the west coast of Britain, mainly including sites between Ilfracombe and Stornoway. Category three events (Fig. 3k–n) cover the coast of Scotland, mainly including sites between Tobermory and Leith. Category four events (Fig. 3p–t) cover the east coast and southeast coasts, mainly including sites between Aberdeen and Newhaven. Of interest is that several events have footprints along two unconnected stretches of coastlines. For example, during two events extreme sea levels occurred in both Liverpool Bay and along the UK east coast (Fig. 3p,q).

**Figure 4:**
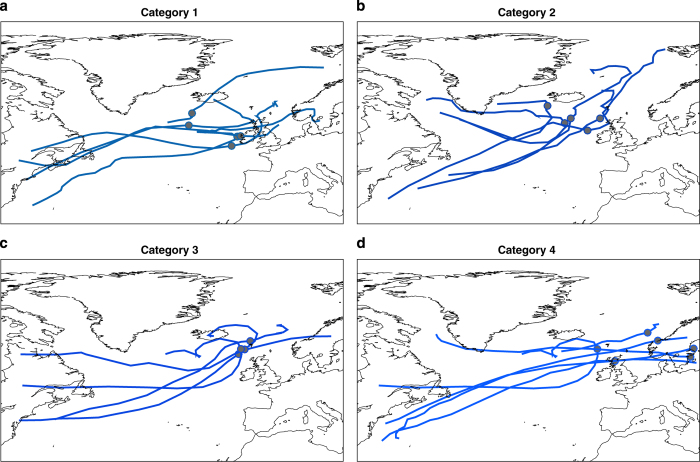
Storm tracks of extreme sea level events for different spatial footprints. The tracks of the storms that generated the sea level events shown in Fig. 3 that impacted at least four tide gauge sites, for category one (**a**), two (**b**), three (**c**) and four (**d**) footprint events. The dots indicate where the storms centres were at time of highest sea level return period.

For the four broad footprint categories, there are similarities in the tracks of the driving storms and locations of the storm centres at the time of highest sea level. The storm tracks of the category one events typically cross the UK (Fig. 4a), while the tracks of the category two, three and four events pass to the north of the UK (Fig. 4b,c). The storm centres (at the time of highest sea level) are typically located to the west of Ireland (Fig. 4a), northwest of Ireland (Fig. 4b), north of Scotland (Fig. 4c) and over Scandinavia (Fig. 4d), for category one to four footprints, respectively. For event categories one to three it is winds to the southeast or south of the storm centre, in combination with low pressure, that generated the large storm surge events along the southwest, west and north coastlines (Supplementary Fig. 2a–n). For event category four, it was mostly northerly winds behind the storm centre once it passes into Scandinavia that generated the elevated sea levels in the North Sea (Supplementary Fig. 2p–t).

Distinct storm tracks are also observed for individual sites (Supplementary Fig. 3). There is a clear northward shift in the average position of storm tracks moving from Newhaven (Site 1) to Wick (Site 30), and then a southward shift of tracks moving from Wick down to Dover (Site 40). Moving from sites 1 to 40, there is a clockwise rotation around the UK in the location of the storm centre at time of maximum sea level (Supplementary Fig. 4).

We also observed four comparable broad categories in the footprints of the skew surge events and corresponding similarities in the tracks of their driving storms (Supplementary Figs 5 and 6). What is striking, however, is that the storms that generated the skew surge events follow a much tighter path across the UK (Supplementary Fig. 7). The location of the storm centre, at the time of maximum skew surge, is also closer to the UK (Supplementary Fig. 4b), particularly for sites on the south and west coasts. This again emphasises that extreme sea level events are mostly generated by moderate rather than extreme skew surges, combined with larger astronomical tides.

### Temporal analysis

**Figure 5:**
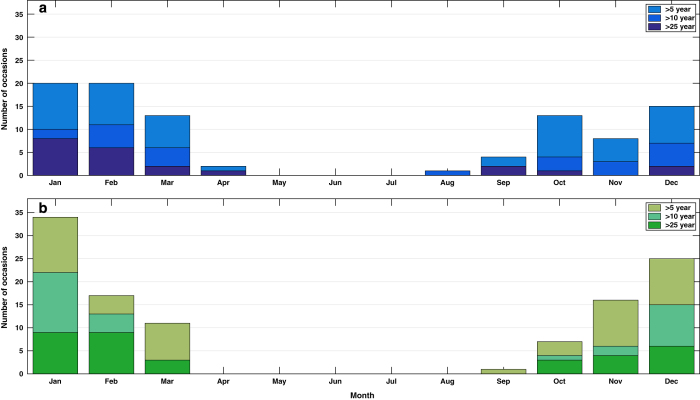
Seasonal distribution of extreme sea level and skew surge events. Number of (**a**) sea level and (**b**) skew surge events per month for different return level thresholds.

Third we examine the temporal variation in events. All of the extreme sea level events occurred between August and April of the following year (Fig. 5a). The large number of events in February, March, and October reflects the influence of astronomical tides, which are largest around the time of the equinoxes^36^. The skew surge events all occurred between September and March (Fig. 5b). This again demonstrates that extreme sea level events, particularly those events occurring in April, August and September, are generated by moderate skew surge events (including the 1 in 27 year extreme sea level event on 7th April 1985). At seasonal and decadal scales there is considerable variation in the number of events (Supplementary Fig. 8), but systematically comparing these over time is not possible because of the incomplete data coverage (Fig. 2c).

**Figure 6:**
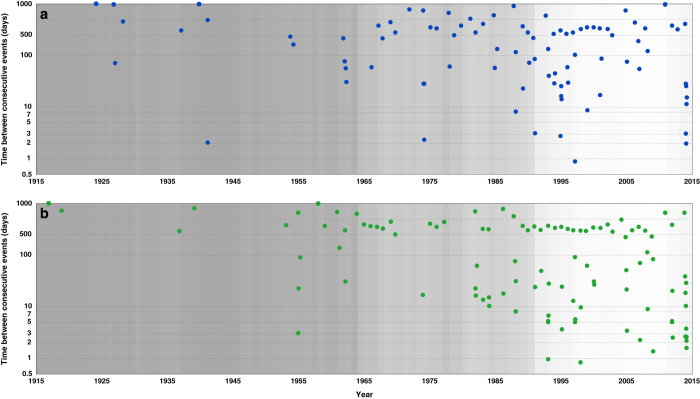
Temporal clustering of extreme sea level and skew surge events. Number of days between successive (**a**) sea level and (**b**) skew surge events. The grey shading indicates the number of sites for which data is available for each year—the lighter the grey the more sites for which data is available.

**Figure 7:**
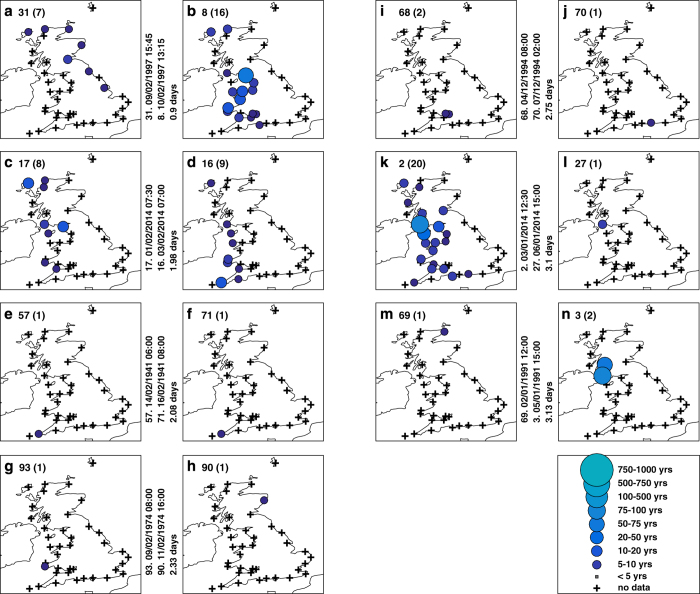
Spatial footprint of sea level events with the closest inter-event spacing. The spatial footprints of the seven pairs of sea level events that had the closest inter-event time spacing (0.9 to 3.1 day intervals). The first number in the top left hand corner indicates the event ranking and the number in the brackets is the number of sites where the 1 in 5 year return level was reached or exceeded.

We examine temporal clustering by considering the number of days between consecutive events. There are seven pairs of sea level events (Fig. 6a) and 13 pairs of skew surge events (Fig. 6b) that occurred within a period of less than four days. Due to lower data coverage in the early part of the record (especially pre-1965), it is likely that the time between consecutive events is overestimated on occasion, because we are missing events. Interestingly, there are no instances of sea level events happening within 4–8 days of each other, whereas there are occasions when pairs of skew surge events occurred within that time interval (Supplementary Fig. 9). Importantly, the pairs of events that occurred in close succession (<4 days) mostly impact different sites and stretches of coastline (Fig. 7).

### The unusual 2013/14 season

Finally we consider how unusual the 2013/14 season was in the context of the last 100 years, from an extreme sea level and skew surge perspective. Across the 40 study sites, the storms during the winter seasons of 2013/14 generated: (1) the maximum-recorded sea level (before accounting for MSL rise) at 20 of the 40 sites; (2) the maximum-recorded sea level return period (after removing MSL rise) at 16 of the 40 sites; and (3) the maximum observed skew surges at 15 of the 40 sites (Supplementary Tables 4 and 5). From an event perspective, the 2013/14 season had: (1) the largest number of extreme sea level and skew surge events—there were almost twice as many skew surge events than in any other season; (2) five occasions in the top 10 largest spatial event footprints; and (3) two instances in the top 10 of the closest inter-event time spacing between events. The seven sea level events in the 2013/14 season all exceeded the 1 in 10 year return level and were in the top 50 events (Supplementary Table 2). The season with the next largest number of extreme sea level events was 1994/95, during which five events exceeded the 1 in 7 year return level, but the ranking of the events was much lower. Four out of 11 skew surge events in the 2013/14 season ranked in the top 20 events, and seven in the top 35 events. The season with the next largest number of extreme skew surge events was 1992/93 with six events, two in the top 20 events. These results suggest that the 2013/14 season was an outlier in the last 100 years. However, it is difficult to quantify this with high statistical certainty because of the reduced spatial coverage in tide gauge sites, and hence incomplete records, especially prior to the 1960s.

## Discussion

We have used observational data to understand actual spatial extents and temporal sequences of extreme sea level and skew surge events around the UK coast over the last 100 years. A key finding is that the vast majority (86%) of the extreme sea level events were generated by moderate, rather than extreme skew surges, combined with high spring tides. The dominant influence of the astronomical tidal component upon observed sea levels is highlighted by the fact that the spatial variation in maximum sea levels around the coast closely resembles the variation in tidal range in most areas (Supplementary Fig. 10).

Using a simple qualitative approach, Zong and Tooley^37^ distinguished three main pathways of storm tracks and three corresponding coastal sectors around the UK influenced by coastal flooding. Our quantitative analysis here identified that there are four main storm track pathways and four broad corresponding footprints of sea level events. These footprints could be used to inform flood management, the insurance sector, and national emergency and infrastructure resilience planning^23,38^. For example, they could be used to assess the likelihood of multiple port failures during the same storm, and thus explore the key national vulnerabilities in the UK port sector and related transport links and supply chains^21^. They could also be used to facilitate a more intelligent spatial treatment of extreme sea level scenarios, rather than assuming a simultaneous and unrealistic 1 in 1000-year return period event, for example, everywhere around the UK coast.

Importantly, we identified that there have been occasional events, when extreme levels have occurred along large lengths of coast and even along two unconnected stretches of coastlines during the same event. This also has important implications for national emergency preparedness and response and resilience planning. Extreme sea levels and flooding occurred along two unconnected stretches of coast during the recent storm Xaver on 5–6/12/2013. Examining the meteorological conditions and the track of the driving storm (Supplementary Fig. 11), it is readily apparent why extreme sea levels occurred along two unconnected stretches of coast. Wadey *et al.*^21^ highlighted that one key difference between this event and the major 1953 event was that the former resulted in flooding in northwest England, as well as along the east (North Sea) and south (English Channel) coasts, whereas the 1953 extreme sea level and flood event appears to have been restricted to the east coast (but the data is more limited in 1953, hindering a more direct comparison).

Clustering of storms is an important issue as it can lead, through compounding effects, to large socioeconomic impacts and cumulative insurance losses^29,32^. Several studies^30–32^, have recently investigated the mechanisms responsible for generating sequences of storms over short periods. From the available data we identified seven pairs of extreme sea level events and 13 pairs of extreme skew surge events that occurred within four days of each other. From a flood risk perspective, a crucial finding is that these close succession events typically impacted different sites and stretches of coastline. This is because the two storms did not follow the same path, but rather deviated to the north or south of each other. Interestingly, we found no recorded instances of extreme sea level events happening within 4–8 days of each other. This is because if storms are separated by 4–8 days, one will always occur during neap tide, and the combined sea level, even with a large storm surge, is unlikely to be high enough to lead to extreme levels (we are aware of one exception—during the St Jude storm in October 2013 flooding occurred on a neap tide and this appeared to be due to an unusual meteorological tsunami^39^). We hypothesize that where tidal ranges are small, such as in the Mediterranean or Baltic Sea, the 4–8 day gap may not be as apparent.

The winter of 2013/14 set records for precipitation totals and the occurrence of extreme wind speeds and experienced the most severe storminess for at least the last 143 years^17^. In regard to extreme sea levels, our results suggest that the 2013/14 season was also exceptional. Since 1915, no season had this number of large extreme sea level and skew surge events. Our analysis is, however, limited by the lack of data going back in time. In a complimentary study we are undertaking a comprehensive review of a variety of ‘soft’ data sources (i.e., unpublished reports, newspaper stories), to see if there is anecdotal evidence of any other season (particularly earlier in the period) when so many events led to coastal flooding (however, this analysis is also influenced by changes to defences over time, which complicate the interpretation^40^). Building on our earlier study^33^, we have reviewed a wide range of relevant sources^37,40–45^. Initial results suggest that no other season in the last 100 years had the number and magnitude of coastal flooding events as seen in the 2013/14 season.

The possible climatological drives of this exceptional season have been discussed in detail in Slingo *et al.*^46^, Huntingford *et al.*^16^ and Wild *et al.*^47^. In short, the UK’s wet and storm winter was linked to record low temperatures on the North American continent^48^, and the contrast between the warm tropical Atlantic and cold air advecting south across the United States are likely to have been partly responsible for the persistence and unusual strength of the North Atlantic jet stream, which created the ideal conditions for generation of storms. Huntingford *et al.*^16^ argue that this in turn could have been related to the filling of the Aleutian Low in the northeast Pacific, which itself could be linked to high sea surface temperatures and a westward displacement of precipitation in the tropical Pacific. They also acknowledge the possible influence of the tropical stratosphere and the potential for Arctic sea ice extent and solar activity to affect the climate of the UK. Kendon and McCarthy^48^ stress the need for further research to better understand the drivers of extreme UK winters and, due to their rare nature and high impact, how they may be affected by climate change.

Herein we have pioneered what we call an ‘Event Analysis’ approach. Most past studies of extreme sea levels have analyzed records from different sites individually. Studies that have assessed the spatial nature of events have typically done this by considering secondary parameters^49,50^ (e.g., the shape parameter of an extreme value distribution), rather than the actual levels among sites. Studies that have compared levels across sites have tended to focus on a limited (<5) number of events^10,21,51^. In contrast we have investigated the characteristics of ~100 events across multiple sites. Our two-staged approach to identify events (see Methods) allowed us to examine a particular event or site, or multiple events or sites, making for a flexible and multifaceted analysis.

We investigated two types of events: (1) extreme sea level events; and (2) extreme skew surges. Extreme sea level events directly lead to the exceedance of critical thresholds and hence are the primary dataset that should be analysed when assessing flooding. However, analyses of skew surge events are also useful, as they provide information on ‘near misses’. As we have shown, some of the skew surge events coincided with the extreme sea level events, when the storm surge occurred around the time of high water of a spring tide; most did not coincide, because the surge occurred near low tide or on a neap tide. Had several of the larger skew surge events occurred on a high spring tide, they would have resulted in higher extreme sea levels than have been observed in the last 100 years. In addition, skew surge time-series are more appropriate proxies for the inter-annual, multi-decadal and longer-term changes in ‘storminess’ and the links to regional climate, compared to extreme sea level time-series that are also influenced by astronomical tides.

The main limitation of our study (and most studies of natural hazards) is the significant decline in data availability going back in time. As we outlined in Haigh *et al.*^33^, there are several ways we could, and plan to, address the issue of missing events and hence the under- or over-estimation of the parameters of interest. In the future we plan to include additional tide-gauge records that do not form part of the National Network (such as at Southampton where a record has been extended back to 1935 (ref. 52)). We also plan to supplement the database with heights of sea level recorded in older journal papers and reports (or even flood markers on buildings). Wadey *et al.*^21^ did this for the 1953 flood event, but this was a time-consuming task, requiring meticulous examination of the data, and uncertainties still remain. Finally, we plan to use sea level predictions from a multi-decadal simulated model hindcast, such as used in various other studies^53–58^.

Waves are also an important component of flooding around the UK coast, which we plan to include in future analyses^40^.

We deliberately removed the influence of MSL, to directly compare the magnitude of events throughout the period analysed. Consistent with other studies^52,59^, when we consider MSL, the number of occurrences of extremes has increased over the last 100 years due to MSL rise and this is expected to continue and accelerate.

Finally, our analysis has focused on the UK coastline, but storm surges and coastal flooding are not limited to national borders during an event. Several of the larger events, such as 31st January—1st February 1953 and 5th—6th December 2013 (refs 21,60), have impacted other countries in northern Europe. Our analysis could be extended to include tide gauge records from other countries to assess the wider spatial footprint of events.

In summary, we have introduced a novel ‘Event Analysis’ approach and used it to assess spatial footprints and temporal clustering of extreme events. Our results have provided important new insights into various aspects of extreme sea level and skew surge events around the UK coast that will improve the ways in which coastal flood risk is identified, assessed and managed. Ongoing investment in the national network of tide gauges and archaeology of historic analogue records^52^ is crucial to continue to provide the datasets upon which our analyses are based. The methods that we developed and implemented can be easily transferred to improve understanding of extreme events in other regions of the world where suitable data exists.

## Methods

The three main data sources we utilized, and the methods we applied to identify extreme sea level and skew surge events in the SurgeWatch database (Data Citation 1), are described in detail by Haigh *et al.*^33^. We therefore provide only a brief overview here, but direct readers to Haigh *et al.*^33^ for a more comprehensive account.

### Data

The primary dataset we use comprises sea level records from the UK National Tide Gauge Network (Data Citation 2). This network currently consists of 43 operational ‘A-class’ tide gauges. It was setup after the 1953 flood event and is owned by the Environment Agency (EA). The network underpins the UK Coastal Monitoring and Forecasting service and is therefore maintained to a high standard. We downloaded the data from the British Oceanographic Data Centre (BODC), who are responsible for the remote monitoring, retrieval, quality-control and archiving of the data. We used data from 40 of the network’s tide gauges (Fig. 1a, Supplementary Table 1). Three sites (Bangor and Portrush in Northern Ireland and Jersey in the Channel Islands) were omitted, because the exceedance probabilities (see description of second type of data below) used to assign return periods to high waters are currently only available for England, Scotland and Wales. The mean data length for all considered records is 38 years (Fig. 1b). We excluded all values which the BODC had flagged as suspect and also undertook our own extensive checks. The frequency of the records changed from hourly to 15-minutes after 1993. We deliberately did not interpolate the data prior to 1993 to 15-minute resolution so that the values could be exactly matched back to the original records.

The second dataset we use is exceedance probabilities from an EA study^34,50^. That study is the most recent in a number of related UK investigations from the last six decades (see Haigh *et al.*^61,^ for a summary) and provides the latest still water level return period estimates around the coastline of England, Scotland and Wales. We extracted the return levels for 16 return periods (from 1 in 1 to 1 in 10,000 years), for each of the 40 tide gauge sites, using information listed in Table 4.1 of McMillian *et al.*^34^. By linearly interpolating these 16 return periods, at each site, we estimated the return periods of each extracted high water and identified those that reached or exceeded the 1 in 5 year return level.

We use a third dataset of gridded mean sea level pressure and near-surface wind fields to: (1) identify distinct extra-tropical storms that produced the extreme sea levels and skew surges events; and (2) to capture the meteorological information from those storms. We used data from the 20th Century global meteorological Reanalysis, Version 2 (ref. 62) (Data Citation 3), which has a spatial and temporal resolution of 2 degrees and 6 h, respectively.

### Analysis approach

For all 40 tide gauges we extracted extreme sea levels and skew surges that reached or exceeded the 1 in 5 year return levels. Extreme sea levels arise as a combination of three main factors: astronomical tide, storm surge and mean sea level (MSL)^36^ (waves can further elevate sea levels, but their effect has not been considered here). We remove the influence of MSL, to directly compare the magnitude of events throughout the period analysed. Many past studies of storm surges in the UK^49,63–66^ have focused on analysing the non-tidal residual—the component that remains once the astronomical tidal component has been removed from the measured sea level record (Fig. 2). The non-tidal residual primarily contains the meteorological contribution termed the ‘surge’, but may also contain harmonic prediction errors or timing errors, and non-linear interactions, which can be large in certain regions, such as the southern North Sea^66^. It is for this reason that we focused our analysis on skew surge, and didn’t use the non-tidal residual. A skew surge is the difference between the maximum observed sea level and the maximum predicted (astronomical) tidal level regardless of their timing during the tidal cycle (Fig. 2). Hence each tidal cycle has one high water value and one associated skew surge value. The advantage of using the skew surge is that it is a simple and unambiguous measure of the storm surge relevant to any predicted high water, and operationally it defines the quantity that may lead to flooding.

Following Haigh *et al.*^33^ we identified the two types of events using a two-staged approach. The first stage of our analysis was to establish when observed sea levels or skew surges reached or exceeded the 1 in 5 year return level. Identifying the extreme sea level events involved several steps. First, we separated the measured sea level at each of the 40 study sites into tidal and non-tidal components, so that the skew surge could be calculated and the relative contribution of the tide could later be identified. The tidal component was predicted using the T-Tide harmonic analysis software^67^. A separate tidal analysis was undertaken for each calendar year using the standard 67 tidal constituents. For years with less than 6 months of data coverage, the tidal component was predicted using harmonic constituents estimated for the nearest year with sufficient data. Second, we extracted all twice-daily measured and predicted high water levels at each site, using a turning point approach^68^, and then calculated skew surges from the measured and predicted high waters. Third, we offset the extracted high waters by the rate of linear MSL rise observed at each site (see Haigh *et al.*^33^ for details). We use a linear MSL trend so that the inter-annual and multi-decadal variations in MSL, which can influence extreme sea levels^13,14^ remain in the time-series. Note, at a few sites with short records the estimated linear trend was unrealistic. In these instances we used a rate interpolated between neighbouring sites with longer records. We offset by MSL to directly compare the return periods of the high water events throughout the record, independently of MSL change. The EA return periods are relative to a baseline level which corresponds to the average sea level in the year 2008 (ref. 34). At sites that have undergone a rise in MSL over the duration of the record, sea levels before 2008 would have a higher return period, and lower return period thereafter. Fourth, we linearly interpolated the EA exceedance probabilities and then estimated the return period of every high water, after offsetting for MSL, so that we could directly compare events throughout the record. Fifth, we stored information associated with the measured high waters, at each of the 40 sites that were equal to or greater than the offset 1 in 5-year return level threshold. For each offset high water that exceeded the threshold we recorded the: (1) date-time of the measured high water; (2) offset return period; (3) measured high water level; (4) predicted high water level; (5) skew surge; and (6) site number (site numbers are listed in Supplementary Table 1 and shown in Fig. 1).

In the SurgeWatch database (Data Citation 1) we stored information about the top 20 skew surges at each site^33^. Here we carried out similar steps to that outlined above (to identify the extreme sea level events) to identify all skew surges that reached or exceeded the 1 in 5 year threshold, so that we could directly compare these to the extreme sea level events identified. The EA study^34^ did not calculate return periods for skew surges. Hence, we calculated our own skew surge return periods for each site. To do this we applied the Peaks over Threshold (POT) method, by fitting a Generalized Pareto distribution (GPD) to all the skew surges that exceeded the 99.75th percentile, at each site in turn. We used a declustering algorithm to ensure independent events. We tested the fit of the GPD distribution using Gringorten’s plotting positions, and found that the GPD fitted the data well at all sites except Portsmouth. The fit at Portsmouth is poor, because this site has a relatively short record (1991–2014), and the largest skew surge value exceeds the second largest value by a large amount (40 cm); this skews the upper tail of the distribution. Hence, at Portsmouth we estimated return periods by fitting a Gumbel distribution to the annual maximum value of each year of data. This provided a better fit to the data and gave return periods that were more comparable to the neighboring sites. The skew surge return levels, at each of the 40 sites, are listed in Supplementary Table 6 for 16 return periods. At each of the 40 sites we stored information associated with the skew surges that were equal to or greater than the 1 in 5-year return level threshold. For each skew surge that reached or exceeded the threshold, we recorded the: (1) date-time of the skew surge; (2) return period; (3) skew surge; (4) measured high water level; (5) predicted high water level; and (6) site number.

The second stage of the analysis was to identify the distinct, extra-tropical storms that produced the extreme sea levels and skew surges that were identified in stage 1, and then to capture the meteorological information about those storms. This involved a two stepped procedure. First, using a simple ‘storm window’ approach, we found that the effect of most storms that caused high sea levels or skew surges in the UK typically lasted up to about 3.5 days. We started with the high water of highest return period, and found all of the other high waters that occurred within a window of 1 day and 18 h before or after that high water (i.e., 3.5 days). We then assigned to these the event number 1. We set all high waters associated with event 1 aside and moved on to the high water with the next highest return period, and so on. Second, we manually used the meteorological data to determine if our above-described procedure had correctly linked high waters to distinct storms. To do this we created an interactive interface in Matlab that displayed the 6-hourly progression of mean sea level pressure and wind vectors over the North Atlantic Ocean and Northern Europe around the time of maximum sea level. On most occasions our simple procedure correctly identified distinct storms. On occasions that it did not (e.g., when examination of the meteorological conditions showed that there were two distinct storms that crossed the UK in this period in close succession; or when our simple procedure identified two events, whereas there was actually only one event, associated with a particularly slow moving storm), we manually separated the high waters into two distinct events, or combined them into one event, and altered the event numbers accordingly. We repeated the procedure for the skew surges.

Using our interactive Matlab interface, we digitized the tracks of all storms that led to the extreme sea level and skew surge events, starting when the low-pressure systems developed, until they dissipated or moved beyond latitude 20°E. We captured the storm tracks by selecting the grid point of lowest atmospheric pressure at each 6-hour time step. From the start to the end of the storm we recorded the 6-hourly: (1) time; (2) latitude; and (3) longitude of the minimum pressure cell; and (4) the minimum mean sea level pressure.

We used the 20th Century Reanalysis because it covers the full 100-year period (i.e., 1915–2014) analysed. It is well known that reanalysis products are prone to inhomogeneities, due to changes in the observing system, and these could influence our storm tracks, particularly earlier in the period assessed^69,70^. However, it is encouragingly that even for events early in the 19th century, a storm was always evident in the reanalysis dataset at the exact time of the extracted extreme sea level or skew surge events. Further, the storm centre, at time of maximum sea level or skew surge was located in the expected region for that event (i.e., to the west of the UK for events that affected the south and west coasts and to the east of the UK for events that affected the east coast). Dangendorf *et al.*^71^ showed that the 20th Century Reanalysis data have a high predictive skill back to the 1910s for the North Sea Region. Further work could assess the difference in storm tracks obtained using a range of reanalysis products and multiple ensemble members within reanlayses. We also recommend that these sorts of studies be updated regularly using the latest reanalysis datasets available.

## Additional information

**How to cite this article:** Haigh, I. D. *et al.* Spatial and temporal analysis of extreme sea level and storm surge events around the coastline of the UK. *Sci. Data* 3:160107 doi: 10.1038/sdata.2016.107 (2016).

**Publisher’s note:** Springer Nature remains neutral with regard to jurisdictional claims in published maps and institutional affiliations.

## Supplementary Material

Supplementary Information
