# Supplementary material for: Spatial and temporal analysis of extreme sea level and storm surge events around the coastline of the UK
Source: Sci Data. 2016 Dec 6;3:160107. doi: 10.1038/sdata.2016.107 (PMC5139689; doi:10.1038/sdata.2016.107)
Supplement: Supplementary Information [file sdata2016107-s1.doc]

**Supplementary Material**

**Spatial footprint and temporal clustering analysis of extreme sea level and storm surge events around the coastline of the UK**

Ivan D. Haigh^1*^, Matthew P. Wadey^1,2^, Thomas Wahl^2,3^, Ozgun Ozsoy^1^, Robert J. Nicholls^2^, Jennifer M. Brown^4^, Kevin Horsburgh^4^ and Ben Gouldby^5^

^1^Ocean and Earth Science, National Oceanography Centre, University of Southampton, European Way, Southampton, SO14 3ZH, UK;

*Corresponding Author: [I.D.Haigh@soton.ac.uk](mailto:I.D.Haigh@soton.ac.uk)

^2^Faculty of Engineering and the Environment, University of Southampton,

Southampton, SO17 1BJ, UK.

^3^Department of Civil, Environmental, and Construction Engineering and Sustainable Coastal Systems Cluster, University of Central Florida, 12800 Pegasus Drive, Suite 211, Orlando, FL 32816-2450, USA.

^4^National Oceanography Centre, Joseph Proudman Building, 6 Brownlow Street,

Liverpool, L3 5DA, UK.

^5^Flood Management Group, HR Wallingford, Howbery Park, Wallingford

Oxfordshire, OX10 8BA

**July 2015**

Scientific Data

**Supplementary Table 1:** Names and locations of study sites.

| **Site Number** | **Site Name** | **Longitude (deg)** | **Latitude**  **(deg)** | **Range** | **Number of Years (Data Range)** | **CD to ODN Conversation (m)** |
| --- | --- | --- | --- | --- | --- | --- |
| 1 | [Newhaven](#Newhaven) | 0.057 | 50.782 | 1982-2014 | 30(33) | -3.52 |
| 2 | [Portsmouth](#Portsmouth) | -1.111 | 50.802 | 1991-2014 | 24(24) | -2.73 |
| 3 | [Bournemouth](#Bournemouth) | -1.875 | 50.714 | 1996-2013 | 18(18) | -1.40 |
| 4 | [Weymouth](#Weymouth) | -2.448 | 50.608 | 1991-2014 | 24(24) | -0.93 |
| 5 | [Devonport](#Devonport) | -4.185 | 50.368 | 1987-2014 | 25(28) | -3.22 |
| 6 | [Newlyn](#Newlyn) | -5.543 | 50.103 | 1915-2014 | 100(100) | -3.05 |
| 7 | [St. Mary’s](#StMarys) | -6.317 | 49.918 | 1976-2014 | 22(39) | -2.91 |
| 8 | [Ilfracombe](#Ilfracombe) | -4.112 | 51.211 | 1968-2014 | 40(47) | -4.80 |
| 9 | [Hinkley](#HinkleyPoint) | -3.134 | 51.215 | 1990-2014 | 24(25) | -5.90 |
| 10 | [Avonmouth](#Avonmouth) | -2.713 | 51.508 | 1961-2012 | 40(52) | -6.50 |
| 11 | [Newport](#Newport) | -2.987 | 51.550 | 1993-2014 | 22(22) | -5.81 |
| 12 | [Mumbles](#Mumbles) | -3.975 | 51.570 | 1988-2014 | 24(27) | -5.00 |
| 13 | [Milford Haven](#MilfordHaven) | -5.052 | 51.707 | 1953-2014 | 54(62) | -3.71 |
| 14 | [Fishguard](#Fishguard) | -4.984 | 52.013 | 1963-2014 | 51(52) | -2.44 |
| 15 | [Barmouth](#Barmouth) | -4.045 | 52.719 | 1991-2014 | 23(24) | -2.44 |
| 16 | [Holyhead](#Holyhead) | -4.620 | 53.314 | 1964-2014 | 44(51) | -3.05 |
| 17 | [Llandudno](#Llandudno) | -3.825 | 53.332 | 1971-2014 | 22(44) | -3.85 |
| 18 | [Liverpool](#Liverpool) | -3.018 | 53.450 | 1991-2014 | 24(24) | -4.93 |
| 19 | [Heysham](#Heysham) | -2.920 | 54.032 | 1964-2014 | 50(51) | -4.90 |
| 20 | [Workington](#Workington) | -3.567 | 54.651 | 1992-2014 | 23(23) | -4.20 |
| 21 | [Port Erin](#PortErin) | -4.768 | 54.085 | 1992-2014 | 21(23) | -2.75 |
| 22 | [Portpatrick](#Portpatrick) | -5.120 | 54.843 | 1968-2014 | 47(47) | -1.80 |
| 23 | [Millport](#Millport) | -4.906 | 55.750 | 1978-2014 | 34(37) | -1.62 |
| 24 | [Port Ellen](#PortEllen) | -6.190 | 55.628 | 1979-2011 | 23(33) | -0.19 |
| 25 | [Tobermory](#Tobermory) | -6.064 | 56.623 | 1990-2014 | 25(25) | -2.39 |
| 26 | [Stornoway](#Stornoway) | -6.389 | 58.208 | 1976-2014 | 36(39) | -2.71 |
| 27 | [Ullapool](#Ullapool) | -5.158 | 57.895 | 1966-2014 | 45(49) | -2.75 |
| 28 | [Kinlochbervie](#Kinlochbervie) | -5.050 | 58.457 | 1991-2014 | 23(24) | -2.50 |
| 29 | [Lerwick](#Lerwick) | -1.140 | 60.154 | 1959-2014 | 56(56) | -1.22 |
| 30 | [Wick](#Wick) | -3.086 | 58.441 | 1965-2014 | 49(50) | -1.71 |
| 31 | [Aberdeen](#Aberdeen) | -2.080 | 57.144 | 1930-2014 | 66(85) | -2.25 |
| 32 | [Leith](#Leith) | -3.182 | 55.990 | 1981-2014 | 27(34) | -2.90 |
| 33 | [North Shields](#NorthShields) | -1.440 | 55.007 | 1946-2014 | 60(69) | -2.60 |
| 34 | [Whitby](#Whitby) | -0.615 | 54.490 | 1980-2014 | 35(35) | -3.00 |
| 35 | [Immingham](#Immingham) | -0.188 | 53.630 | 1953-2014 | 56(62) | -3.90 |
| 36 | [Cromer](#Cromer) | 1.302 | 52.934 | 1973-2014 | 30(42) | -2.75 |
| 37 | [Lowestoft](#Lowestoft) | 1.750 | 52.473 | 1964-2014 | 51(51) | -1.50 |
| 38 | [Harwich](#Harwich) | 1.292 | 51.948 | 1954-2014 | 28(61) | -2.02 |
| 39 | [Sheerness](#Sheerness) | 0.743 | 51.446 | 1952-2010 | 44(59) | -2.90 |
| 40 | [Dover](#Dover) | 1.323 | 51.114 | 1924-2014 | 65(91) | -3.67 |

**Supplementary Table 2:** Extreme sea level events

| **Event Number** | **Date and Time** | **Return Period (years)** | **Water level**  **(m CD)** | **Astronomical Tide (m)** | **Skew Surge (m)** | **Site ID** | **Number of Sites** |
| --- | --- | --- | --- | --- | --- | --- | --- |
| 1 | 06/12/2013 00:45 | 843 | 8.45 | 6.81 | 1.64 | 40 | 12 |
| 2 | 03/01/2014 12:30 | 244 | 5.28 | 4.38 | 0.89 | 22 | 20 |
| 3 | 05/01/1991 15:00 | 134 | 5.16 | 4.06 | 1.10 | 22 | 2 |
| 4 | 01/02/2002 14:00 | 113 | 5.17 | 4.41 | 0.76 | 22 | 12 |
| 5 | 13/12/1981 21:00 | 102 | 15.43 | 13.35 | 2.08 | 10 | 3 |
| 6 | 01/02/1983 01:00 | 100 | 11.56 | 9.82 | 1.74 | 19 | 7 |
| 7 | 11/01/1993 12:30 | 89 | 3.04 | 2.49 | 0.55 | 29 | 13 |
| 8 | 10/02/1997 13:15 | 86 | 9.96 | 9.21 | 0.75 | 20 | 16 |
| 9 | 11/01/2005 19:00 | 71 | 6.06 | 4.54 | 1.52 | 25 | 8 |
| 10 | 31/01/1953 17:00 | 62 | 6.16 | 4.75 | 1.35 | 33 | 2 |
| 11 | 29/09/1969 07:00 | 43 | 8.56 | 7.34 | 1.16 | 35 | 6 |
| 12 | 10/03/2008 09:00 | 40 | 3.04 | 2.48 | 0.53 | 4 | 7 |
| 13 | 29/01/1948 07:00 | 39 | 6.32 | 5.87 | 0.45 | 6 | 1 |
| 14 | 21/03/1961 02:00 | 38 | 7.90 | 6.81 | 1.10 | 40 | 1 |
| 15 | 27/10/2004 16:15 | 37 | 6.42 | 5.64 | 0.78 | 6 | 3 |
| 16 | 03/02/2014 07:00 | 37 | 6.44 | 5.97 | 0.47 | 6 | 9 |
| 17 | 01/02/2014 07:30 | 35 | 5.97 | 5.49 | 0.48 | 26 | 8 |
| 18 | 23/09/1953 18:00 | 34 | 8.09 | 7.83 | 0.26 | 13 | 1 |
| 19 | 03/01/1976 21:00 | 33 | 4.18 | 2.32 | 1.66 | 37 | 3 |
| 20 | 07/04/1985 06:00 | 28 | 6.36 | 5.97 | 0.39 | 6 | 3 |
| 21 | 21/02/1993 09:00 | 27 | 4.18 | 2.32 | 1.77 | 37 | 3 |
| 22 | 11/01/1978 20:00 | 27 | 8.51 | 7.53 | 0.98 | 35 | 2 |
| 23 | 24/12/1999 08:00 | 23 | 6.11 | 5.40 | 0.70 | 28 | 4 |
| 24 | 07/03/1962 07:00 | 21 | 8.06 | 7.83 | 0.23 | 13 | 1 |
| 25 | 09/11/2007 08:15 | 19 | 4.13 | 2.55 | 1.56 | 37 | 1 |
| 26 | 01/01/1995 22:45 | 18 | 7.87 | 6.84 | 1.00 | 40 | 3 |
| 27 | 06/01/2014 15:00 | 18 | 4.99 | 4.21 | 0.78 | 22 | 1 |
| 28 | 10/12/1965 13:00 | 18 | 6.92 | 5.60 | 1.33 | 39 | 1 |
| 29 | 10/10/1926 12:00 | 17 | 7.72 | 6.69 | 0.86 | 40 | 1 |
| 30 | 24/10/1961 06:00 | 17 | 8.03 | 7.45 | 0.58 | 13 | 1 |
| 31 | 09/02/1997 15:45 | 16 | 6.54 | 6.02 | 0.52 | 32 | 7 |
| 32 | 06/02/1924 00:00 | 15 | 7.69 | 6.75 | 0.95 | 40 | 1 |
| 33 | 27/02/1990 12:00 | 15 | 2.93 | 2.54 | 0.39 | 29 | 7 |
| 34 | 14/11/1993 23:15 | 14 | 7.82 | 6.92 | 0.90 | 40 | 2 |
| 35 | 23/12/1995 07:30 | 14 | 2.93 | 2.69 | 0.24 | 4 | 3 |
| 36 | 14/12/1973 00:00 | 14 | 3.97 | 2.70 | 1.27 | 37 | 3 |
| 37 | 23/03/1928 06:00 | 14 | 6.20 | 5.82 | 0.38 | 6 | 1 |
| 38 | 11/11/1977 11:00 | 13 | 4.31 | 3.72 | 0.59 | 30 | 1 |
| 39 | 09/02/1988 16:00 | 12 | 4.68 | 3.32 | 1.35 | 23 | 1 |
| 40 | 06/03/1954 19:00 | 12 | 7.97 | 7.54 | 0.43 | 13 | 1 |
| 41 | 12/12/2000 23:45 | 12 | 4.34 | 3.69 | 0.63 | 30 | 3 |
| 42 | 29/08/1992 20:00 | 11 | 5.72 | 5.40 | 0.32 | 14 | 5 |
| 43 | 14/02/2014 19:45 | 11 | 2.93 | 1.90 | 0.94 | 4 | 2 |
| 44 | 02/03/2014 07:45 | 11 | 13.72 | 13.35 | 0.38 | 11 | 5 |
| 45 | 08/10/2006 18:45 | 10 | 8.11 | 7.94 | 0.16 | 13 | 5 |
| 46 | 03/01/1998 14:45 | 10 | 4.89 | 4.12 | 0.77 | 22 | 1 |
| 47 | 28/02/1967 16:00 | 10 | 5.10 | 4.61 | 0.49 | 31 | 2 |
| 48 | 09/03/1989 07:00 | 10 | 10.38 | 10.24 | 0.14 | 8 | 3 |
| 49 | 24/03/1936 05:00 | 10 | 6.19 | 6.07 | 0.12 | 6 | 1 |
| 50 | 30/03/2006 07:45 | 10 | 5.74 | 5.42 | 0.33 | 14 | 8 |
| 51 | 06/10/1979 17:00 | 9 | 6.26 | 5.99 | 0.27 | 6 | 1 |
| 52 | 23/11/1984 23:00 | 9 | 2.90 | 2.53 | 0.38 | 29 | 2 |
| 53 | 17/10/2012 05:15 | 9 | 6.32 | 6.06 | 0.26 | 6 | 3 |
| 54 | 27/11/2011 16:30 | 9 | 6.05 | 5.31 | 0.75 | 33 | 2 |
| 55 | 18/01/1995 07:30 | 9 | 6.21 | 5.21 | 0.99 | 27 | 4 |
| 56 | 28/01/1994 09:30 | 9 | 3.91 | 2.39 | 1.47 | 37 | 2 |
| 57 | 14/02/1941 06:00 | 9 | 6.18 | 5.88 | 0.31 | 6 | 1 |
| 58 | 03/12/2006 05:45 | 9 | 2.90 | 2.17 | 0.68 | 4 | 2 |
| 59 | 11/11/2010 15:15 | 9 | 4.65 | 3.44 | 1.21 | 23 | 2 |
| 60 | 08/03/1981 08:00 | 8 | 5.77 | 5.37 | 0.40 | 26 | 2 |
| 61 | 04/01/1999 13:30 | 8 | 4.62 | 3.81 | 0.81 | 23 | 2 |
| 62 | 07/10/1990 12:00 | 8 | 7.73 | 7.12 | 0.62 | 40 | 2 |
| 63 | 08/02/1966 09:00 | 8 | 5.62 | 5.29 | 0.33 | 14 | 1 |
| 64 | 30/01/1975 09:00 | 8 | 5.64 | 5.41 | 0.23 | 14 | 1 |
| 65 | 06/03/1931 06:00 | 8 | 6.16 | 5.91 | 0.24 | 6 | 1 |
| 66 | 01/02/1988 11:00 | 8 | 4.60 | 3.26 | 1.33 | 23 | 2 |
| 67 | 05/10/1967 00:00 | 8 | 4.97 | 4.37 | 0.59 | 38 | 3 |
| 68 | 04/12/1994 08:00 | 8 | 14.97 | 14.25 | 0.69 | 10 | 2 |
| 69 | 02/01/1991 12:00 | 8 | 4.29 | 3.80 | 0.49 | 30 | 1 |
| 70 | 07/12/1994 02:00 | 8 | 5.48 | 4.78 | 0.68 | 2 | 1 |
| 71 | 16/02/1941 08:00 | 7 | 6.17 | 5.58 | 0.56 | 6 | 1 |
| 72 | 26/12/1998 20:00 | 7 | 1.96 | 0.80 | 1.14 | 24 | 1 |
| 73 | 28/01/1937 06:00 | 7 | 6.16 | 5.63 | 0.53 | 6 | 1 |
| 74 | 10/03/2001 07:15 | 7 | 2.87 | 2.65 | 0.23 | 4 | 2 |
| 75 | 01/02/1995 14:00 | 7 | 5.10 | 4.66 | 0.43 | 31 | 1 |
| 76 | 22/12/1968 13:00 | 7 | 4.77 | 4.16 | 0.61 | 22 | 1 |
| 77 | 07/10/1987 18:00 | 7 | 6.31 | 5.93 | 0.38 | 5 | 1 |
| 78 | 16/09/1978 19:00 | 6 | 5.74 | 5.12 | 0.61 | 26 | 1 |
| 79 | 17/03/1957 14:00 | 6 | 5.04 | 4.74 | 0.29 | 31 | 1 |
| 80 | 26/09/1984 19:00 | 6 | 10.32 | 10.29 | 0.03 | 8 | 1 |
| 81 | 09/01/1962 13:00 | 6 | 2.87 | 2.43 | 0.43 | 29 | 1 |
| 82 | 21/12/1926 00:00 | 6 | 7.57 | 6.63 | 0.94 | 40 | 1 |
| 83 | 11/01/1974 13:00 | 6 | 4.24 | 3.89 | 0.33 | 30 | 1 |
| 84 | 14/12/1993 00:15 | 6 | 6.77 | 5.72 | 1.03 | 39 | 1 |
| 85 | 21/11/1971 23:00 | 6 | 3.77 | 2.48 | 1.29 | 37 | 1 |
| 86 | 24/10/1995 23:15 | 6 | 4.81 | 4.06 | 0.74 | 22 | 2 |
| 87 | 13/10/1939 17:00 | 6 | 6.14 | 5.87 | 0.28 | 6 | 1 |
| 88 | 22/01/1996 05:45 | 6 | 6.25 | 6.03 | 0.22 | 6 | 1 |
| 89 | 14/02/1989 06:00 | 6 | 3.81 | 1.94 | 1.41 | 37 | 1 |
| 90 | 11/02/1974 16:00 | 6 | 5.05 | 4.39 | 0.67 | 31 | 1 |
| 91 | 29/10/1996 13:30 | 6 | 6.77 | 5.76 | 0.87 | 39 | 2 |
| 92 | 08/10/2002 18:00 | 6 | 6.26 | 6.10 | 0.14 | 6 | 1 |
| 93 | 09/02/1974 08:00 | 5 | 7.95 | 7.72 | 0.22 | 13 | 1 |
| 94 | 16/12/1989 07:00 | 5 | 6.23 | 5.66 | 0.57 | 6 | 1 |
| 95 | 25/11/2000 20:15 | 5 | 6.15 | 5.16 | 0.90 | 15 | 1 |
| 96 | 07/04/1962 08:00 | 5 | 7.91 | 7.68 | 0.23 | 13 | 1 |

**Supplementary Table 3:** Extreme skew surge events

| **Event Number** | **Date and Time** | **Return Period (years)** | **Skew Surge (m)** | **Water level**  **(m CD)** | **Astronomical Tide (m)** | **Site ID** | **Number of Sites** |
| --- | --- | --- | --- | --- | --- | --- | --- |
| 1 | 31/01/1953 17:00 | 516 | 1.35 | 6.16 | 4.75 | 33 | 2 |
| 2 | 11/01/1993 00:15 | 495 | 0.70 | 3.03 | 2.32 | 29 | 4 |
| 3 | 14/02/2014 23:00 | 349 | 1.20 | 5.54 | 4.28 | 2 | 7 |
| 4 | 16/10/1987 05:00 | 180 | 1.14 | 5.77 | 4.57 | 1 | 2 |
| 5 | 08/12/2011 17:30 | 179 | 1.16 | 5.84 | 4.67 | 27 | 1 |
| 6 | 06/12/2013 00:45 | 103 | 1.64 | 8.45 | 6.81 | 40 | 5 |
| 7 | 11/01/2005 19:00 | 100 | 1.52 | 6.06 | 4.54 | 25 | 7 |
| 8 | 02/02/2002 02:15 | 84 | 0.65 | 2.73 | 2.07 | 29 | 3 |
| 9 | 15/03/1977 15:00 | 73 | 1.21 | 6.43 | 5.22 | 13 | 1 |
| 10 | 06/11/1996 07:15 | 72 | 1.61 | 4.15 | 2.40 | 23 | 5 |
| 11 | 04/02/2014 20:00 | 71 | 0.73 | 6.03 | 5.30 | 7 | 4 |
| 12 | 01/02/1926 07:00 | 69 | 0.82 | 5.97 | 5.15 | 6 | 1 |
| 13 | 25/10/2008 21:00 | 64 | 0.82 | 4.06 | 3.23 | 30 | 2 |
| 14 | 21/02/1993 06:15 | 61 | 1.69 | 6.25 | 4.51 | 36 | 7 |
| 15 | 26/11/1954 17:00 | 54 | 0.80 | 5.86 | 5.05 | 6 | 1 |
| 16 | 13/12/1981 21:00 | 50 | 2.08 | 15.43 | 13.35 | 10 | 1 |
| 17 | 10/11/1963 13:00 | 46 | 0.79 | 5.21 | 4.40 | 6 | 2 |
| 18 | 08/01/2005 08:30 | 46 | 1.59 | 9.97 | 8.34 | 18 | 6 |
| 19 | 15/01/1952 15:00 | 46 | 0.90 | 4.85 | 3.95 | 31 | 1 |
| 20 | 27/12/2013 06:00 | 45 | 1.19 | 4.62 | 3.42 | 22 | 9 |
| 21 | 29/11/1954 19:00 | 43 | 0.78 | 5.61 | 4.83 | 6 | 1 |
| 22 | 01/02/1983 01:00 | 42 | 1.74 | 11.56 | 9.82 | 19 | 5 |
| 23 | 24/02/1997 20:15 | 40 | 1.49 | 12.94 | 11.45 | 9 | 4 |
| 24 | 27/10/2004 16:15 | 39 | 0.78 | 6.42 | 5.64 | 6 | 2 |
| 25 | 17/01/1969 17:00 | 38 | 0.95 | 9.75 | 8.81 | 8 | 2 |
| 26 | 16/01/1993 07:15 | 37 | 0.89 | 4.52 | 3.63 | 31 | 3 |
| 27 | 01/03/2008 11:00 | 37 | 1.45 | 6.82 | 5.31 | 35 | 5 |
| 28 | 12/02/2014 18:30 | 34 | 1.41 | 5.59 | 4.08 | 15 | 1 |
| 29 | 24/12/2013 14:45 | 34 | 0.62 | 2.65 | 2.02 | 29 | 7 |
| 30 | 24/03/1986 05:00 | 33 | 0.93 | 9.30 | 8.37 | 8 | 1 |
| 31 | 24/12/1997 19:45 | 31 | 1.41 | 8.06 | 6.64 | 20 | 5 |
| 32 | 03/01/2012 08:15 | 29 | 1.46 | 4.32 | 2.67 | 23 | 3 |
| 33 | 01/02/1988 11:00 | 26 | 1.13 | 4.80 | 3.67 | 22 | 3 |
| 34 | 04/01/1998 10:15 | 26 | 1.26 | 10.37 | 9.11 | 12 | 5 |
| 35 | 08/02/2014 14:00 | 23 | 0.93 | 4.36 | 3.43 | 14 | 3 |
| 36 | 01/01/1992 11:00 | 22 | 0.85 | 4.58 | 3.73 | 31 | 4 |
| 37 | 18/01/1995 07:30 | 22 | 0.99 | 6.21 | 5.21 | 27 | 3 |
| 38 | 02/01/1984 22:00 | 21 | 0.91 | 6.21 | 5.31 | 16 | 2 |
| 39 | 02/01/2001 05:00 | 21 | 0.76 | 2.74 | 1.96 | 3 | 1 |
| 40 | 04/01/1976 00:00 | 20 | 1.32 | 7.80 | 6.48 | 40 | 4 |
| 41 | 09/02/1988 15:00 | 20 | 1.58 | 10.15 | 8.57 | 19 | 4 |
| 42 | 16/02/1962 22:00 | 20 | 1.31 | 7.23 | 5.92 | 40 | 2 |
| 43 | 26/12/1998 20:00 | 20 | 1.14 | 1.96 | 0.80 | 24 | 1 |
| 44 | 05/01/1936 14:00 | 20 | 0.73 | 5.50 | 4.77 | 6 | 1 |
| 45 | 05/01/1991 15:00 | 19 | 1.10 | 5.16 | 4.06 | 22 | 2 |
| 46 | 16/12/1989 21:00 | 19 | 1.19 | 9.84 | 8.65 | 12 | 6 |
| 47 | 19/02/1997 18:30 | 18 | 1.33 | 5.47 | 4.12 | 15 | 6 |
| 48 | 22/12/1954 09:00 | 17 | 1.36 | 4.54 | 3.16 | 38 | 1 |
| 49 | 06/12/1973 21:00 | 16 | 1.41 | 6.30 | 4.89 | 39 | 1 |
| 50 | 18/12/1983 15:00 | 16 | 0.71 | 5.87 | 5.16 | 6 | 1 |
| 51 | 13/01/1984 07:00 | 15 | 1.49 | 8.83 | 7.18 | 19 | 2 |
| 52 | 11/11/2010 16:45 | 14 | 1.10 | 1.99 | 0.89 | 24 | 2 |
| 53 | 29/01/2000 22:30 | 14 | 0.92 | 5.08 | 4.13 | 33 | 4 |
| 54 | 17/01/1993 06:15 | 14 | 1.06 | 4.43 | 3.33 | 22 | 3 |
| 55 | 14/12/2011 01:00 | 13 | 0.59 | 2.57 | 1.98 | 29 | 2 |
| 56 | 12/12/1990 13:00 | 13 | 1.09 | 5.62 | 4.50 | 34 | 5 |
| 57 | 24/01/1993 00:15 | 12 | 1.24 | 9.32 | 8.09 | 20 | 4 |
| 58 | 03/01/2000 05:30 | 12 | 1.04 | 5.17 | 4.12 | 28 | 1 |
| 59 | 26/10/1967 04:00 | 12 | 0.73 | 3.51 | 2.77 | 30 | 2 |
| 60 | 01/12/1966 12:00 | 11 | 0.85 | 6.18 | 5.33 | 16 | 1 |
| 61 | 09/11/2007 08:15 | 11 | 1.56 | 4.13 | 2.55 | 37 | 2 |
| 62 | 10/12/1965 13:00 | 11 | 1.33 | 6.92 | 5.60 | 39 | 1 |
| 63 | 05/01/2012 20:45 | 11 | 1.20 | 6.70 | 5.50 | 40 | 3 |
| 64 | 18/01/1983 09:00 | 10 | 1.19 | 7.66 | 6.38 | 35 | 1 |
| 65 | 06/03/1986 07:00 | 10 | 0.58 | 2.14 | 1.56 | 29 | 1 |
| 66 | 02/11/1918 03:00 | 9 | 0.67 | 5.61 | 4.92 | 6 | 1 |
| 67 | 03/03/1982 05:00 | 9 | 1.28 | 4.39 | 3.11 | 23 | 2 |
| 68 | 19/11/1996 07:00 | 9 | 0.70 | 2.79 | 2.08 | 3 | 1 |
| 69 | 11/01/2007 12:00 | 9 | 0.91 | 5.21 | 4.30 | 27 | 2 |
| 70 | 05/01/1993 23:00 | 9 | 0.78 | 4.40 | 3.62 | 31 | 2 |
| 71 | 15/12/1979 04:00 | 9 | 1.54 | 11.40 | 9.87 | 10 | 1 |
| 72 | 17/01/2009 21:45 | 9 | 1.11 | 5.01 | 3.85 | 25 | 1 |
| 73 | 14/11/2002 06:45 | 9 | 0.80 | 6.23 | 5.41 | 1 | 1 |
| 74 | 03/12/1999 08:45 | 9 | 1.27 | 9.33 | 7.80 | 18 | 2 |
| 75 | 29/09/1969 07:00 | 9 | 1.16 | 8.56 | 7.34 | 35 | 3 |
| 76 | 10/12/1957 19:00 | 8 | 0.66 | 5.84 | 5.18 | 6 | 1 |
| 77 | 30/12/1981 07:00 | 8 | 0.66 | 5.83 | 5.17 | 6 | 1 |
| 78 | 28/01/1994 09:30 | 8 | 1.47 | 3.91 | 2.39 | 37 | 1 |
| 79 | 19/11/1973 20:00 | 7 | 1.23 | 6.30 | 5.06 | 39 | 2 |
| 80 | 19/03/2004 10:15 | 7 | 1.33 | 10.77 | 9.43 | 19 | 1 |
| 81 | 14/02/1989 07:00 | 7 | 1.22 | 6.33 | 4.97 | 39 | 2 |
| 82 | 11/02/2014 04:30 | 7 | 1.32 | 10.38 | 9.06 | 11 | 1 |
| 83 | 19/12/1958 11:00 | 7 | 0.64 | 5.15 | 4.50 | 6 | 1 |
| 84 | 02/03/1997 12:15 | 7 | 0.97 | 4.90 | 3.92 | 28 | 1 |
| 85 | 17/01/1962 04:00 | 7 | 0.93 | 6.63 | 5.67 | 13 | 1 |
| 86 | 23/03/1955 04:00 | 7 | 0.64 | 5.93 | 5.29 | 6 | 1 |
| 87 | 03/12/2011 16:45 | 7 | 0.56 | 2.33 | 1.77 | 29 | 1 |
| 88 | 01/11/2006 07:45 | 6 | 1.16 | 4.54 | 3.25 | 38 | 1 |
| 89 | 11/11/2005 14:30 | 6 | 1.06 | 5.17 | 4.11 | 25 | 1 |
| 90 | 18/01/1939 03:00 | 6 | 0.64 | 5.49 | 4.85 | 6 | 1 |
| 91 | 12/11/1991 15:00 | 6 | 0.97 | 4.52 | 3.57 | 22 | 1 |
| 92 | 21/03/1961 02:00 | 6 | 1.10 | 7.90 | 6.81 | 40 | 1 |
| 93 | 31/12/1987 21:00 | 6 | 0.56 | 2.33 | 1.77 | 29 | 1 |
| 94 | 11/02/1995 15:45 | 6 | 0.76 | 7.86 | 7.04 | 8 | 1 |
| 95 | 08/12/1964 01:00 | 6 | 1.31 | 9.67 | 8.36 | 19 | 1 |
| 96 | 27/10/1936 11:00 | 6 | 0.74 | 4.46 | 3.71 | 31 | 1 |
| 97 | 19/01/2009 06:30 | 6 | 0.67 | 2.69 | 2.00 | 3 | 1 |
| 98 | 15/02/1995 07:00 | 6 | 0.87 | 5.94 | 5.06 | 27 | 1 |
| 99 | 15/01/1975 12:00 | 6 | 0.56 | 2.78 | 2.22 | 29 | 1 |
| 100 | 01/11/1960 15:00 | 6 | 0.64 | 5.88 | 5.24 | 6 | 1 |
| 101 | 10/03/2008 10:30 | 6 | 0.66 | 3.09 | 2.43 | 3 | 1 |
| 102 | 13/01/2007 18:30 | 6 | 1.19 | 8.66 | 7.42 | 18 | 1 |
| 103 | 17/12/2004 15:30 | 6 | 0.75 | 6.89 | 6.08 | 1 | 1 |
| 104 | 24/10/1998 21:00 | 6 | 1.41 | 13.37 | 11.90 | 10 | 1 |
| 105 | 05/11/1916 01:00 | 5 | 0.63 | 5.15 | 4.53 | 6 | 1 |
| 106 | 28/10/2013 05:45 | 5 | 0.77 | 4.70 | 3.93 | 2 | 1 |
| 107 | 01/03/1949 04:00 | 5 | 0.81 | 5.37 | 4.55 | 33 | 1 |
| 108 | 06/01/2014 14:00 | 5 | 0.78 | 6.56 | 5.78 | 16 | 1 |
| 109 | 06/01/1996 17:30 | 5 | 0.58 | 5.92 | 5.32 | 7 | 1 |
| 110 | 21/11/1981 02:00 | 5 | 1.07 | 7.22 | 6.16 | 35 | 1 |
| 111 | 25/12/1997 15:45 | 5 | 1.39 | 11.68 | 10.29 | 10 | 2 |

**Supplementary Table 4:** Largest recorded return level sea levels at each site.

| **Site Number** | **Site Name** | **Date and Time** | **Return Period (years)** | **Water level (m CD)** | **Astronomical Tide (m)** | **Skew Surge (m)** |
| --- | --- | --- | --- | --- | --- | --- |
| 1 | [Newhaven](#Newhaven) | 06/12/2013 01:15 | 39 | 7.79 | 7.00 | 0.73 |
| 2 | [Portsmouth](#Portsmouth) | 06/12/2013 01:00 | 12 | 5.56 | 4.87 | 0.69 |
| 3 | [Bournemouth](#Bournemouth) | 10/03/2008 10:30 | 23 | 3.09 | 2.43 | 0.66 |
| 4 | [Weymouth](#Weymouth) | 10/03/2008 09:00 | 40 | 3.04 | 2.48 | 0.53 |
| 5 | [Devonport](#Devonport) | 03/02/2014 08:15 | 8 | 6.37 | 5.91 | 0.46 |
| 6 | [Newlyn](#Newlyn) | 29/01/1948 07:00 | 39 | 6.32 | 5.87 | 0.45 |
| 7 | [St. Mary’s](#StMarys) | 30/03/2006 05:15 | 6 | 6.45 | 6.17 | 0.28 |
| 8 | [Ilfracombe](#Ilfracombe) | 07/04/1985 07:00 | 23 | 10.46 | 10.07 | 0.39 |
| 9 | [Hinkley](#HinkleyPoint) | 03/01/2014 08:00 | 13 | 13.35 | 12.71 | 0.65 |
| 10 | [Avonmouth](#Avonmouth) | 13/12/1981 21:00 | 102 | 15.43 | 13.35 | 2.08 |
| 11 | [Newport](#Newport) | 02/03/2014 07:45 | 11 | 13.72 | 13.35 | 0.38 |
| 12 | [Mumbles](#Mumbles) | 03/01/2014 07:15 | 9 | 10.73 | 10.21 | 0.50 |
| 13 | [Milford Haven](#MilfordHaven) | 23/09/1953 18:00 | 34 | 8.09 | 7.83 | 0.26 |
| 14 | [Fishguard](#Fishguard) | 10/02/1997 09:15 | 20 | 5.78 | 5.36 | 0.42 |
| 15 | [Barmouth](#Barmouth) | 10/02/1997 10:00 | 25 | 6.36 | 5.68 | 0.68 |
| 16 | [Holyhead](#Holyhead) | 01/02/2002 12:45 | 92 | 6.86 | 6.12 | 0.74 |
| 17 | [Llandudno](#Llandudno) | 10/02/1997 12:30 | 28 | 8.95 | 8.49 | 0.46 |
| 18 | [Liverpool](#Liverpool) | 05/12/2013 12:30 | 43 | 11.15 | 9.96 | 1.17 |
| 19 | [Heysham](#Heysham) | 01/02/1983 01:00 | 100 | 11.56 | 9.82 | 1.74 |
| 20 | [Workington](#Workington) | 10/02/1997 13:15 | 86 | 9.96 | 9.21 | 0.75 |
| 21 | [Port Erin](#PortErin) | 03/01/2014 12:30 | 68 | 6.62 | 5.82 | 0.79 |
| 22 | [Portpatrick](#Portpatrick) | 03/01/2014 12:30 | 244 | 5.28 | 4.38 | 0.89 |
| 23 | [Millport](#Millport) | 05/01/1991 15:00 | 81 | 5.07 | 3.76 | 1.31 |
| 24 | [Port Ellen](#PortEllen) | 11/11/2010 16:45 | 8 | 1.99 | 0.89 | 1.10 |
| 25 | [Tobermory](#Tobermory) | 11/01/2005 19:00 | 71 | 6.06 | 4.54 | 1.52 |
| 26 | [Stornoway](#Stornoway) | 01/02/2014 07:30 | 35 | 5.97 | 5.49 | 0.48 |
| 27 | [Ullapool](#Ullapool) | 12/01/2005 08:15 | 57 | 6.44 | 5.51 | 0.93 |
| 28 | [Kinlochbervie](#Kinlochbervie) | 12/01/2005 08:30 | 70 | 6.28 | 5.21 | 1.06 |
| 29 | [Lerwick](#Lerwick) | 11/01/1993 12:30 | 89 | 3.04 | 2.49 | 0.55 |
| 30 | [Wick](#Wick) | 12/01/2005 12:30 | 65 | 4.50 | 3.74 | 0.76 |
| 31 | [Aberdeen](#Aberdeen) | 12/01/2005 14:30 | 57 | 5.31 | 4.55 | 0.76 |
| 32 | [Leith](#Leith) | 05/12/2013 15:15 | 21 | 6.61 | 5.74 | 0.70 |
| 33 | [North Shields](#NorthShields) | 05/12/2013 16:15 | 405 | 6.58 | 5.42 | 1.08 |
| 34 | [Whitby](#Whitby) | 05/12/2013 17:15 | 568 | 7.32 | 6.08 | 1.24 |
| 35 | [Immingham](#Immingham) | 05/12/2013 19:15 | 787 | 9.12 | 7.50 | 1.62 |
| 36 | [Cromer](#Cromer) | 21/02/1993 06:15 | 9 | 6.25 | 4.51 | 1.69 |
| 37 | [Lowestoft](#Lowestoft) | 05/12/2013 22:30 | 188 | 4.76 | 2.79 | 1.93 |
| 38 | [Harwich](#Harwich) | 05/10/1967 00:00 | 8 | 4.97 | 4.37 | 0.59 |
| 39 | [Sheerness](#Sheerness) | 10/12/1965 13:00 | 18 | 6.92 | 5.60 | 1.33 |
| 40 | [Dover](#Dover) | 06/12/2013 00:45 | 843 | 8.45 | 6.81 | 1.64 |

**Supplementary Table 5:** Largest skew surges at each site.

| **Site Number** | **Site Name** | **Date and Time** | **Return Period (years)** | **Skew Surge (m)** | **Water level (m CD)** | **Astronomial Tide (m)** |
| --- | --- | --- | --- | --- | --- | --- |
| 1 | [Newhaven](#Newhaven) | 16/10/1987 05:00 | 180 | 1.135 | 5.77 | 4.573 |
| 2 | [Portsmouth](#Portsmouth) | 14/02/2014 23:00 | 349 | 1.196 | 5.537 | 4.283 |
| 3 | [Bournemouth](#Bournemouth) | 02/01/2001 05:00 | 21 | 0.763 | 2.735 | 1.96 |
| 4 | [Weymouth](#Weymouth) | 14/02/2014 19:45 | 44 | 0.944 | 2.934 | 1.895 |
| 5 | [Devonport](#Devonport) | 14/02/2014 17:45 | 22 | 0.924 | 6.225 | 5.301 |
| 6 | [Newlyn](#Newlyn) | 01/02/1926 07:00 | 69 | 0.82 | 5.971 | 5.151 |
| 7 | [St. Mary’s](#StMarys) | 04/02/2014 20:00 | 71 | 0.734 | 6.029 | 5.295 |
| 8 | [Ilfracombe](#Ilfracombe) | 17/01/1969 17:00 | 38 | 0.949 | 9.754 | 8.805 |
| 9 | [Hinkley](#HinkleyPoint) | 24/02/1997 20:15 | 40 | 1.488 | 12.939 | 11.447 |
| 10 | [Avonmouth](#Avonmouth) | 13/12/1981 21:00 | 50 | 2.084 | 15.43 | 13.346 |
| 11 | [Newport](#Newport) | 24/02/1997 20:45 | 33 | 1.698 | 13.416 | 11.722 |
| 12 | [Mumbles](#Mumbles) | 04/01/1998 10:15 | 26 | 1.259 | 10.371 | 9.112 |
| 13 | [Milford Haven](#MilfordHaven) | 15/03/1977 15:00 | 73 | 1.211 | 6.427 | 5.216 |
| 14 | [Fishguard](#Fishguard) | 14/02/2014 19:30 | 87 | 1.086 | 5.295 | 4.209 |
| 15 | [Barmouth](#Barmouth) | 12/02/2014 18:30 | 34 | 1.412 | 5.586 | 4.076 |
| 16 | [Holyhead](#Holyhead) | 27/12/2013 05:15 | 36 | 0.943 | 5.596 | 4.651 |
| 17 | [Llandudno](#Llandudno) | 19/02/1997 21:00 | 14 | 0.908 | 7.596 | 6.585 |
| 18 | [Liverpool](#Liverpool) | 08/01/2005 08:30 | 46 | 1.585 | 9.967 | 8.336 |
| 19 | [Heysham](#Heysham) | 01/02/1983 01:00 | 42 | 1.744 | 11.56 | 9.822 |
| 20 | [Workington](#Workington) | 27/12/2013 06:00 | 31 | 1.408 | 8.136 | 6.736 |
| 21 | [Port Erin](#PortErin) | 27/12/2013 05:45 | 34 | 1.027 | 5.402 | 4.358 |
| 22 | [Portpatrick](#Portpatrick) | 27/12/2013 06:00 | 45 | 1.188 | 4.624 | 3.417 |
| 23 | [Millport](#Millport) | 06/11/1996 07:15 | 72 | 1.605 | 4.154 | 2.402 |
| 24 | [Port Ellen](#PortEllen) | 26/12/1998 20:00 | 20 | 1.138 | 1.959 | 0.796 |
| 25 | [Tobermory](#Tobermory) | 11/01/2005 19:00 | 100 | 1.515 | 6.062 | 4.542 |
| 26 | [Stornoway](#Stornoway) | 24/12/2013 23:15 | 17 | 0.788 | 4.739 | 3.942 |
| 27 | [Ullapool](#Ullapool) | 08/12/2011 17:30 | 179 | 1.16 | 5.842 | 4.672 |
| 28 | [Kinlochbervie](#Kinlochbervie) | 11/01/2005 20:15 | 20 | 1.095 | 5.956 | 4.861 |
| 29 | [Lerwick](#Lerwick) | 11/01/1993 00:15 | 495 | 0.699 | 3.03 | 2.318 |
| 30 | [Wick](#Wick) | 25/10/2008 21:00 | 64 | 0.824 | 4.059 | 3.226 |
| 31 | [Aberdeen](#Aberdeen) | 15/01/1952 15:00 | 46 | 0.904 | 4.852 | 3.948 |
| 32 | [Leith](#Leith) | 16/01/1993 08:15 | 16 | 0.939 | 5.753 | 4.784 |
| 33 | [North Shields](#NorthShields) | 31/01/1953 17:00 | 516 | 1.353 | 6.163 | 4.749 |
| 34 | [Whitby](#Whitby) | 05/12/2013 17:15 | 31 | 1.237 | 7.319 | 6.08 |
| 35 | [Immingham](#Immingham) | 05/12/2013 19:15 | 75 | 1.617 | 9.116 | 7.499 |
| 36 | [Cromer](#Cromer) | 21/02/1993 06:15 | 61 | 1.69 | 6.25 | 4.509 |
| 37 | [Lowestoft](#Lowestoft) | 05/12/2013 22:30 | 56 | 1.933 | 4.764 | 2.794 |
| 38 | [Harwich](#Harwich) | 22/12/1954 09:00 | 17 | 1.358 | 4.535 | 3.155 |
| 39 | [Sheerness](#Sheerness) | 06/12/1973 21:00 | 16 | 1.408 | 6.303 | 4.889 |
| 40 | [Dover](#Dover) | 06/12/2013 00:45 | 103 | 1.637 | 8.449 | 6.812 |

**Supplementary Table 6:** Skew surge return level estimates for each of the 40 study sites for different return periods.

| **Site Number** | **Site Name** | **Return Period (years)** | | | | | | | | | | |
| --- | --- | --- | --- | --- | --- | --- | --- | --- | --- | --- | --- | --- |
|  |  | **1** | **2** | **5** | **10** | **25** | **50** | **75** | **100** | **250** | **500** | **1000** |
| 1 | [Newhaven](#Newhaven) | 0.57 | 0.65 | 0.74 | 0.82 | 0.92 | 0.99 | 1.04 | 1.07 | 1.17 | 1.25 | 1.33 |
| 2 | [Portsmouth](#Portsmouth) | 0.60 | 0.67 | 0.77 | 0.84 | 0.93 | 1.00 | 1.04 | 1.07 | 1.16 | 1.24 | 1.31 |
| 3 | [Bournemouth](#Bournemouth) | 0.53 | 0.58 | 0.66 | 0.71 | 0.78 | 0.83 | 0.86 | 0.88 | 0.94 | 0.99 | 1.04 |
| 4 | [Weymouth](#Weymouth) | 0.53 | 0.60 | 0.70 | 0.77 | 0.88 | 0.96 | 1.02 | 1.05 | 1.18 | 1.28 | 1.38 |
| 5 | [Devonport](#Devonport) | 0.56 | 0.63 | 0.74 | 0.82 | 0.94 | 1.03 | 1.09 | 1.14 | 1.28 | 1.39 | 1.51 |
| 6 | [Newlyn](#Newlyn) | 0.50 | 0.56 | 0.63 | 0.68 | 0.75 | 0.80 | 0.83 | 0.85 | 0.91 | 0.96 | 1.01 |
| 7 | [St. Mary’s](#StMarys) | 0.48 | 0.53 | 0.58 | 0.63 | 0.68 | 0.72 | 0.74 | 0.75 | 0.80 | 0.83 | 0.86 |
| 8 | [Ilfracombe](#Ilfracombe) | 0.58 | 0.65 | 0.74 | 0.81 | 0.91 | 0.98 | 1.03 | 1.06 | 1.16 | 1.24 | 1.32 |
| 9 | [Hinkley](#HinkleyPoint) | 0.78 | 0.89 | 1.06 | 1.19 | 1.39 | 1.56 | 1.66 | 1.74 | 2.01 | 2.23 | 2.48 |
| 10 | [Avonmouth](#Avonmouth) | 1.02 | 1.17 | 1.39 | 1.58 | 1.85 | 2.08 | 2.23 | 2.34 | 2.73 | 3.05 | 3.41 |
| 11 | [Newport](#Newport) | 0.95 | 1.08 | 1.27 | 1.42 | 1.64 | 1.82 | 1.93 | 2.01 | 2.29 | 2.51 | 2.75 |
| 12 | [Mumbles](#Mumbles) | 0.70 | 0.80 | 0.95 | 1.07 | 1.25 | 1.40 | 1.50 | 1.57 | 1.81 | 2.01 | 2.23 |
| 13 | [Milford Haven](#MilfordHaven) | 0.71 | 0.79 | 0.90 | 0.98 | 1.09 | 1.17 | 1.22 | 1.25 | 1.35 | 1.43 | 1.51 |
| 14 | [Fishguard](#Fishguard) | 0.59 | 0.67 | 0.76 | 0.84 | 0.94 | 1.02 | 1.07 | 1.10 | 1.22 | 1.30 | 1.39 |
| 15 | [Barmouth](#Barmouth) | 0.90 | 1.00 | 1.14 | 1.24 | 1.38 | 1.48 | 1.53 | 1.57 | 1.70 | 1.79 | 1.89 |
| 16 | [Holyhead](#Holyhead) | 0.63 | 0.69 | 0.78 | 0.84 | 0.92 | 0.98 | 1.01 | 1.03 | 1.11 | 1.16 | 1.21 |
| 17 | [Llandudno](#Llandudno) | 0.65 | 0.73 | 0.82 | 0.88 | 0.97 | 1.03 | 1.06 | 1.08 | 1.16 | 1.21 | 1.26 |
| 18 | [Liverpool](#Liverpool) | 0.89 | 1.01 | 1.17 | 1.30 | 1.47 | 1.61 | 1.69 | 1.75 | 1.94 | 2.09 | 2.25 |
| 19 | [Heysham](#Heysham) | 0.95 | 1.08 | 1.27 | 1.42 | 1.63 | 1.80 | 1.91 | 1.98 | 2.23 | 2.43 | 2.65 |
| 20 | [Workington](#Workington) | 0.85 | 0.96 | 1.10 | 1.22 | 1.38 | 1.50 | 1.57 | 1.62 | 1.80 | 1.93 | 2.07 |
| 21 | [Port Erin](#PortErin) | 0.69 | 0.76 | 0.85 | 0.92 | 1.01 | 1.07 | 1.10 | 1.13 | 1.20 | 1.26 | 1.31 |
| 22 | [Portpatrick](#Portpatrick) | 0.75 | 0.84 | 0.95 | 1.03 | 1.13 | 1.21 | 1.25 | 1.28 | 1.38 | 1.45 | 1.52 |
| 23 | [Millport](#Millport) | 0.92 | 1.04 | 1.18 | 1.29 | 1.44 | 1.55 | 1.61 | 1.66 | 1.80 | 1.91 | 2.01 |
| 24 | [Port Ellen](#PortEllen) | 0.80 | 0.88 | 0.99 | 1.07 | 1.16 | 1.24 | 1.28 | 1.30 | 1.39 | 1.46 | 1.52 |
| 25 | [Tobermory](#Tobermory) | 0.79 | 0.89 | 1.03 | 1.14 | 1.28 | 1.40 | 1.47 | 1.52 | 1.68 | 1.80 | 1.93 |
| 26 | [Stornoway](#Stornoway) | 0.59 | 0.64 | 0.71 | 0.76 | 0.82 | 0.86 | 0.89 | 0.90 | 0.96 | 0.99 | 1.03 |
| 27 | [Ullapool](#Ullapool) | 0.70 | 0.77 | 0.86 | 0.92 | 1.00 | 1.06 | 1.09 | 1.12 | 1.19 | 1.24 | 1.29 |
| 28 | [Kinlochbervie](#Kinlochbervie) | 0.77 | 0.85 | 0.95 | 1.02 | 1.12 | 1.19 | 1.23 | 1.26 | 1.34 | 1.41 | 1.47 |
| 29 | [Lerwick](#Lerwick) | 0.47 | 0.51 | 0.55 | 0.58 | 0.61 | 0.64 | 0.65 | 0.66 | 0.68 | 0.70 | 0.71 |
| 30 | [Wick](#Wick) | 0.58 | 0.63 | 0.69 | 0.73 | 0.78 | 0.81 | 0.83 | 0.85 | 0.88 | 0.91 | 0.94 |
| 31 | [Aberdeen](#Aberdeen) | 0.60 | 0.66 | 0.73 | 0.79 | 0.86 | 0.91 | 0.94 | 0.96 | 1.03 | 1.08 | 1.12 |
| 32 | [Leith](#Leith) | 0.60 | 0.68 | 0.79 | 0.88 | 1.01 | 1.11 | 1.18 | 1.23 | 1.39 | 1.52 | 1.66 |
| 33 | [North Shields](#NorthShields) | 0.63 | 0.70 | 0.81 | 0.89 | 0.99 | 1.07 | 1.12 | 1.16 | 1.27 | 1.35 | 1.44 |
| 34 | [Whitby](#Whitby) | 0.71 | 0.81 | 0.95 | 1.06 | 1.21 | 1.33 | 1.40 | 1.45 | 1.63 | 1.76 | 1.91 |
| 35 | [Immingham](#Immingham) | 0.79 | 0.91 | 1.07 | 1.20 | 1.38 | 1.53 | 1.62 | 1.68 | 1.90 | 2.08 | 2.26 |
| 36 | [Cromer](#Cromer) | 0.87 | 0.99 | 1.17 | 1.31 | 1.50 | 1.65 | 1.74 | 1.81 | 2.03 | 2.20 | 2.38 |
| 37 | [Lowestoft](#Lowestoft) | 1.05 | 1.19 | 1.39 | 1.54 | 1.75 | 1.91 | 2.00 | 2.07 | 2.29 | 2.46 | 2.63 |
| 38 | [Harwich](#Harwich) | 0.83 | 0.95 | 1.12 | 1.26 | 1.46 | 1.62 | 1.72 | 1.79 | 2.04 | 2.24 | 2.46 |
| 39 | [Sheerness](#Sheerness) | 0.87 | 1.00 | 1.17 | 1.31 | 1.52 | 1.68 | 1.78 | 1.86 | 2.11 | 2.32 | 2.53 |
| 40 | [Dover](#Dover) | 0.79 | 0.91 | 1.06 | 1.19 | 1.36 | 1.49 | 1.57 | 1.63 | 1.82 | 1.98 | 2.13 |

| 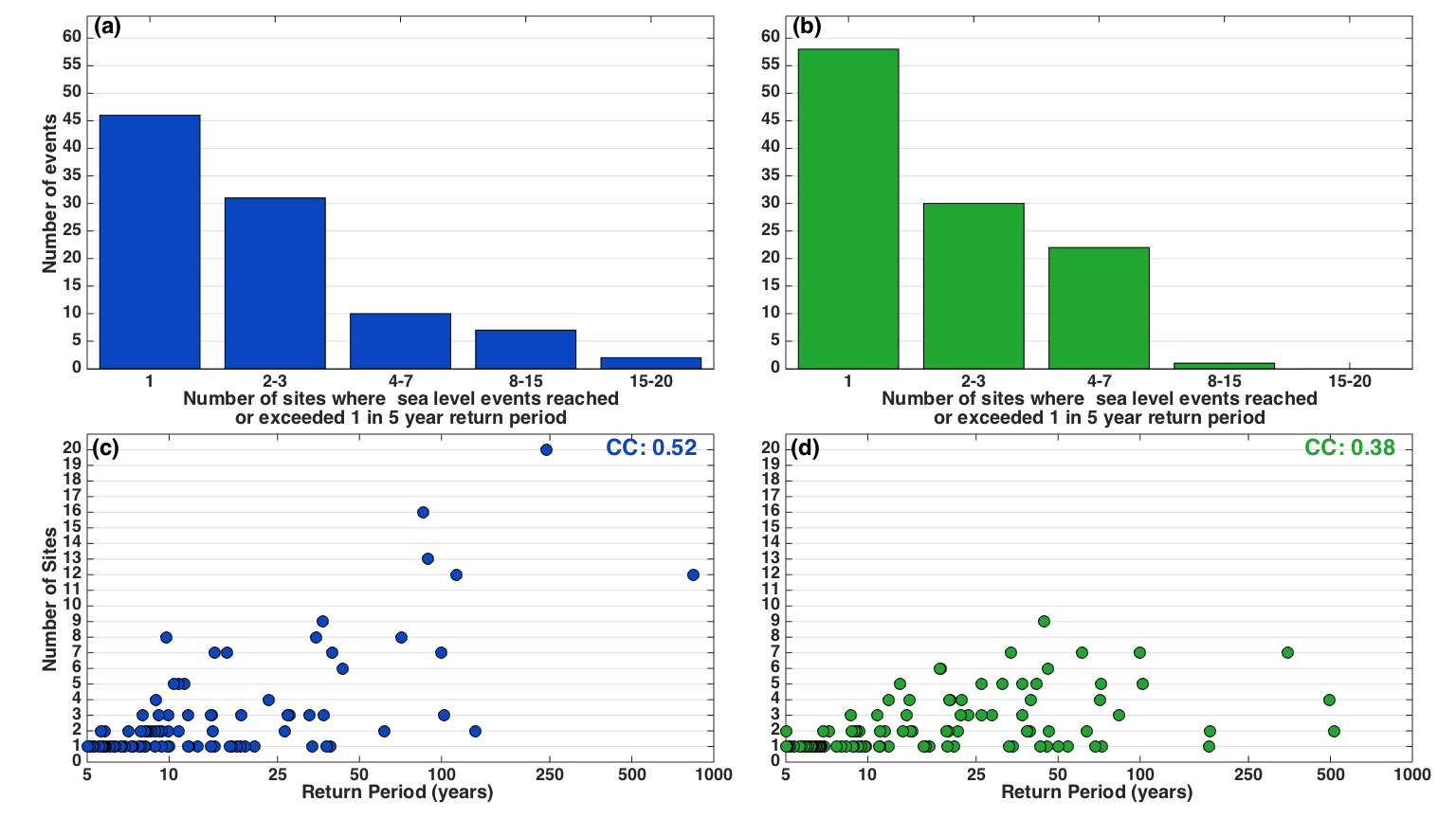 |
| --- |
| **Supplementary Figure 1:** Histograms showing the number of sites where the 1 in 5 year return level was reached or exceed for (a) sea level events and (b) skew surge events. The offset return period of the (c) highest sea levels in each of the 96 sea level events, and (d) the highest skew surge return periods for each of 111 skew surge events, plotted against the number of sites where the 1 in 5 year return period was reached or exceeded during an event. |

| 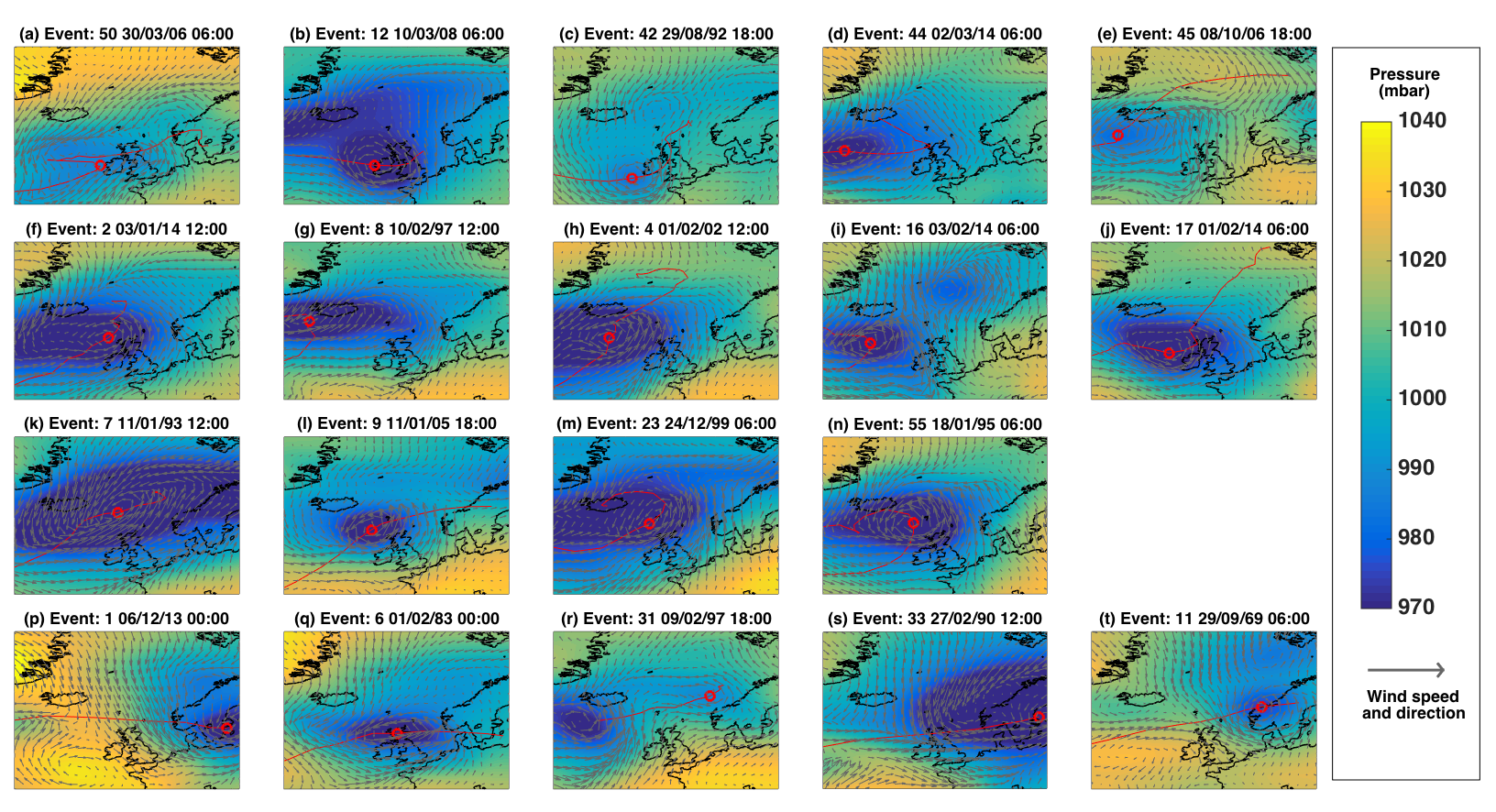 |
| --- |
| **Supplementary Figure 2:** Atmospheric pressure and wind speed at time of highest sea level for the corresponding sea level events shown in Supplementary Fig. 3 that impacted at least four tide gauge sites. The red line shows the storm track and the red circle indicates the location of the storm centre at time of highest sea level return period. |

| 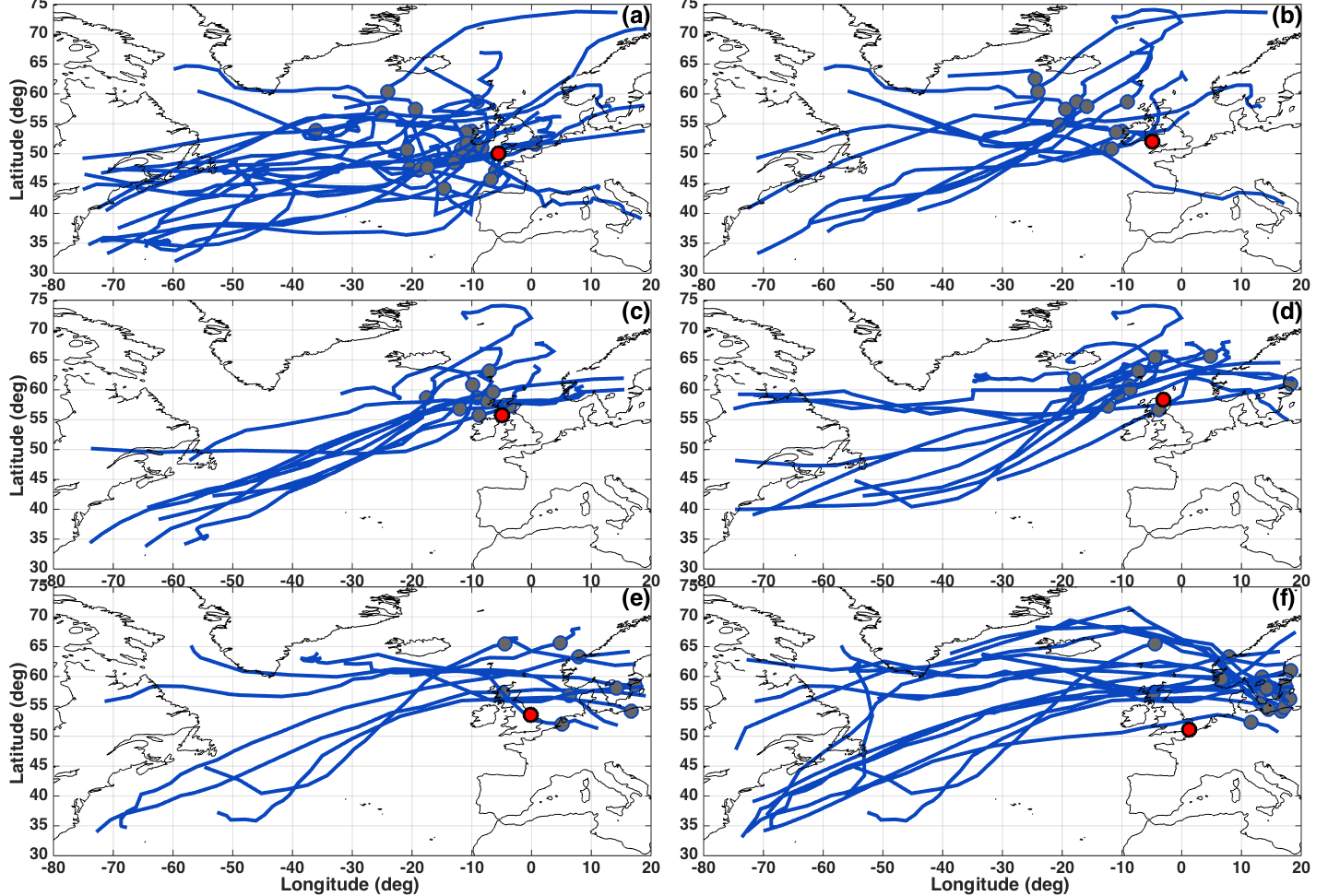 |
| --- |
| **Supplementary Figure 3:** The tracks of the storms that generated sea level events that reached or exceed the 1 in 5 year return level at: (a) Newlyn; (b) Fishguard; (c) Millport; (d) Wick; (e) Immingham; and (f) Dover. The dots indicate the location of storm centres at time of highest sea level, for each storm. The red dots show the location of the tide gauge in question. |

| 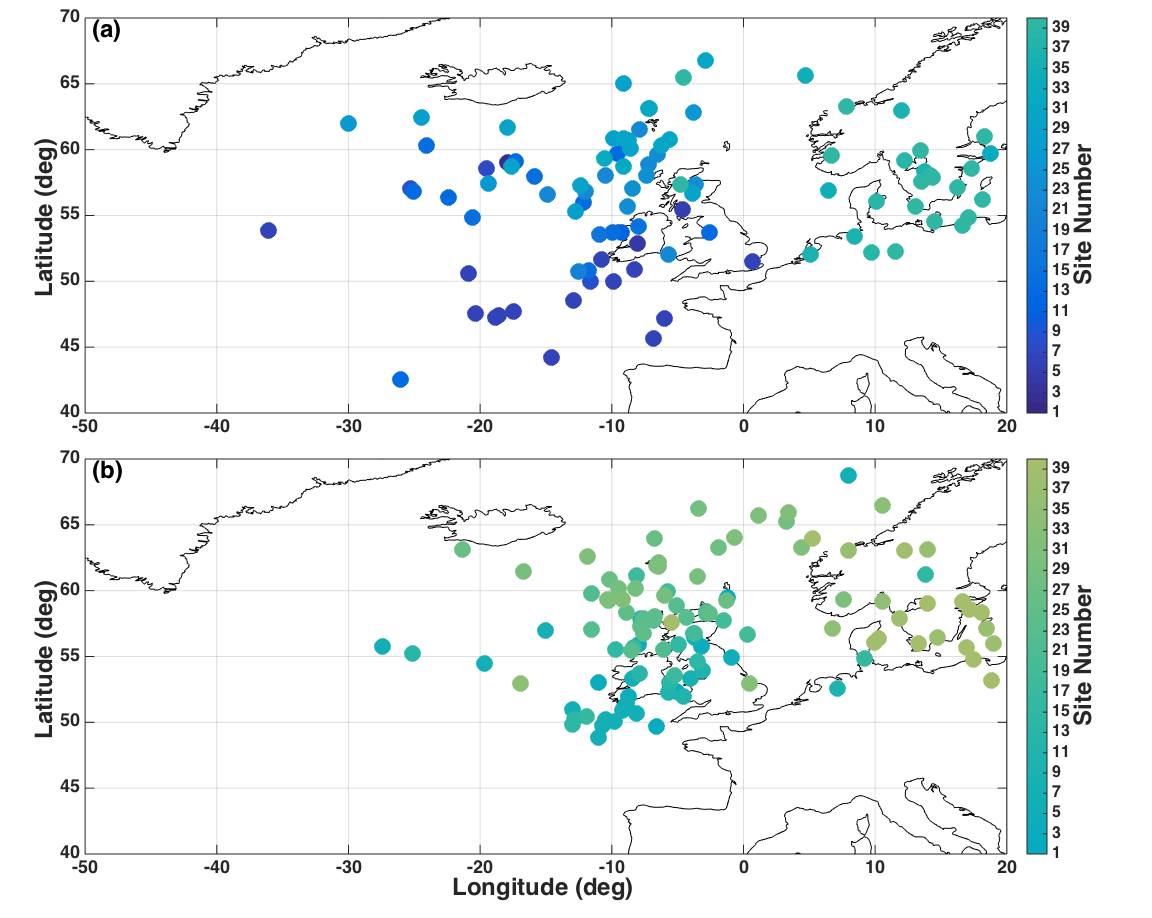 |
| --- |
| **Supplementary Figure 4:** The location of the storm centres at time of highest (a) sea level return period and (b) skew surge, for each of the 96 sea level and 111 skew surge events, respectively. The blue/green shading corresponds to the site number, for each of the 40 sites. |

| 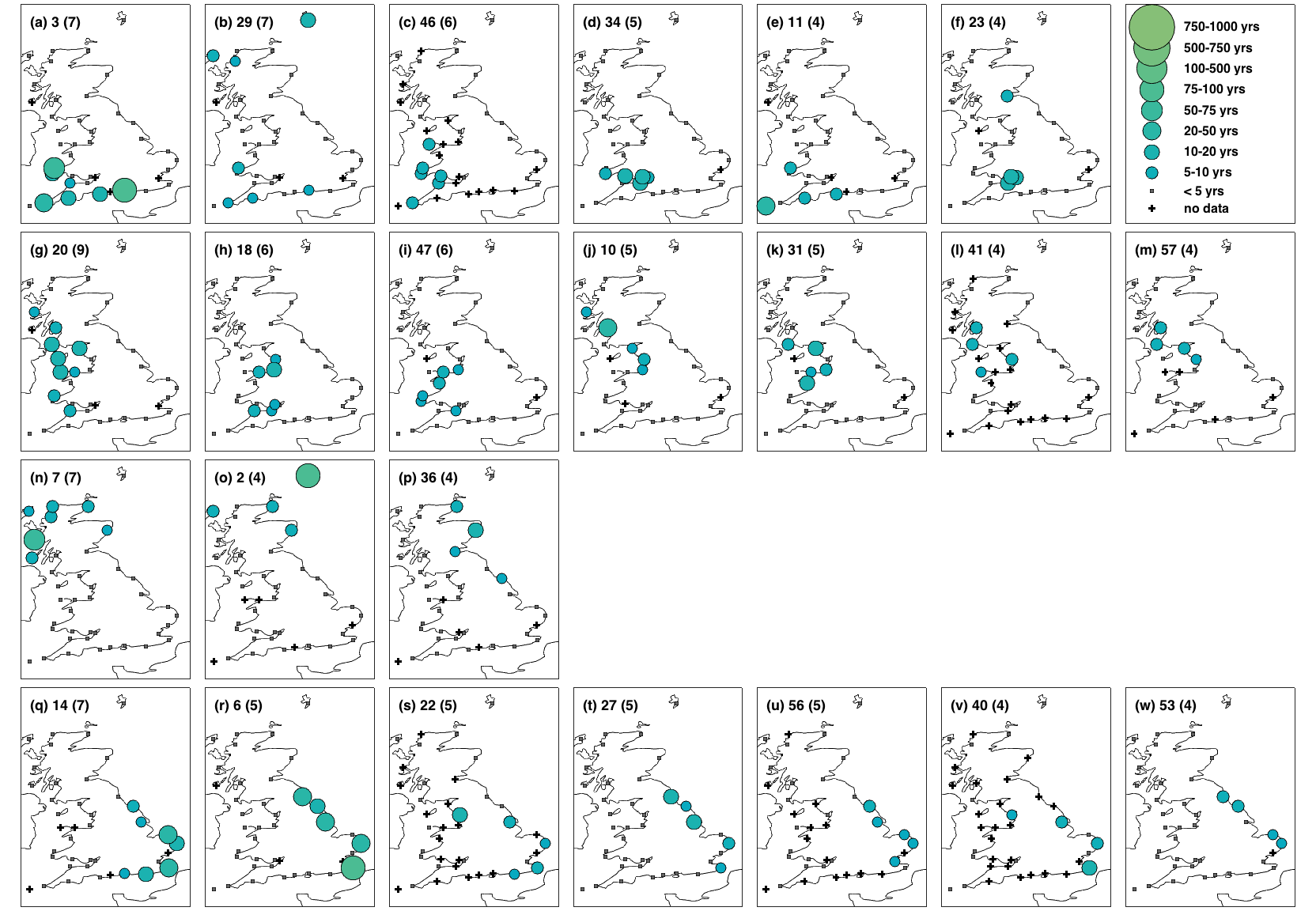 |
| --- |
| **Supplementary Figure 5:** The spatial footprints of all the skew surge events that impacted at least four tide gauge sites. The first number in the top left hand corner indicates the event ranking and the number in the bracket is the number of sites where the 1 in 5 year return level was reached or exceeded. The category one, two, three and four footprint events are shown in the first, second, third and fourth row of panels, respectively. |

| 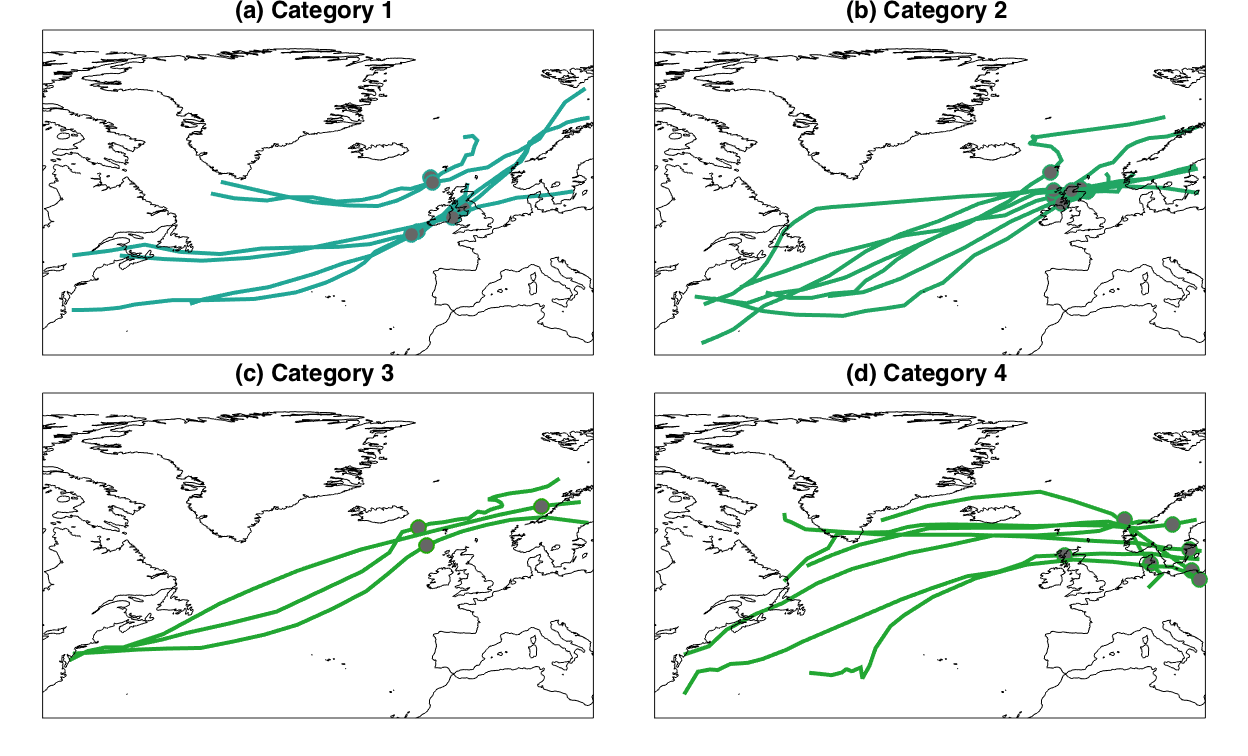 |
| --- |
| **Supplementary Figure 6:** The tracks of the storms that generated the skew surge level events shown in Supplementary Figure 5 that impacted at least four tide gauge sites, for the four regions identified. The dots indicate where the storms centres were at time of highest skew surge. |

| 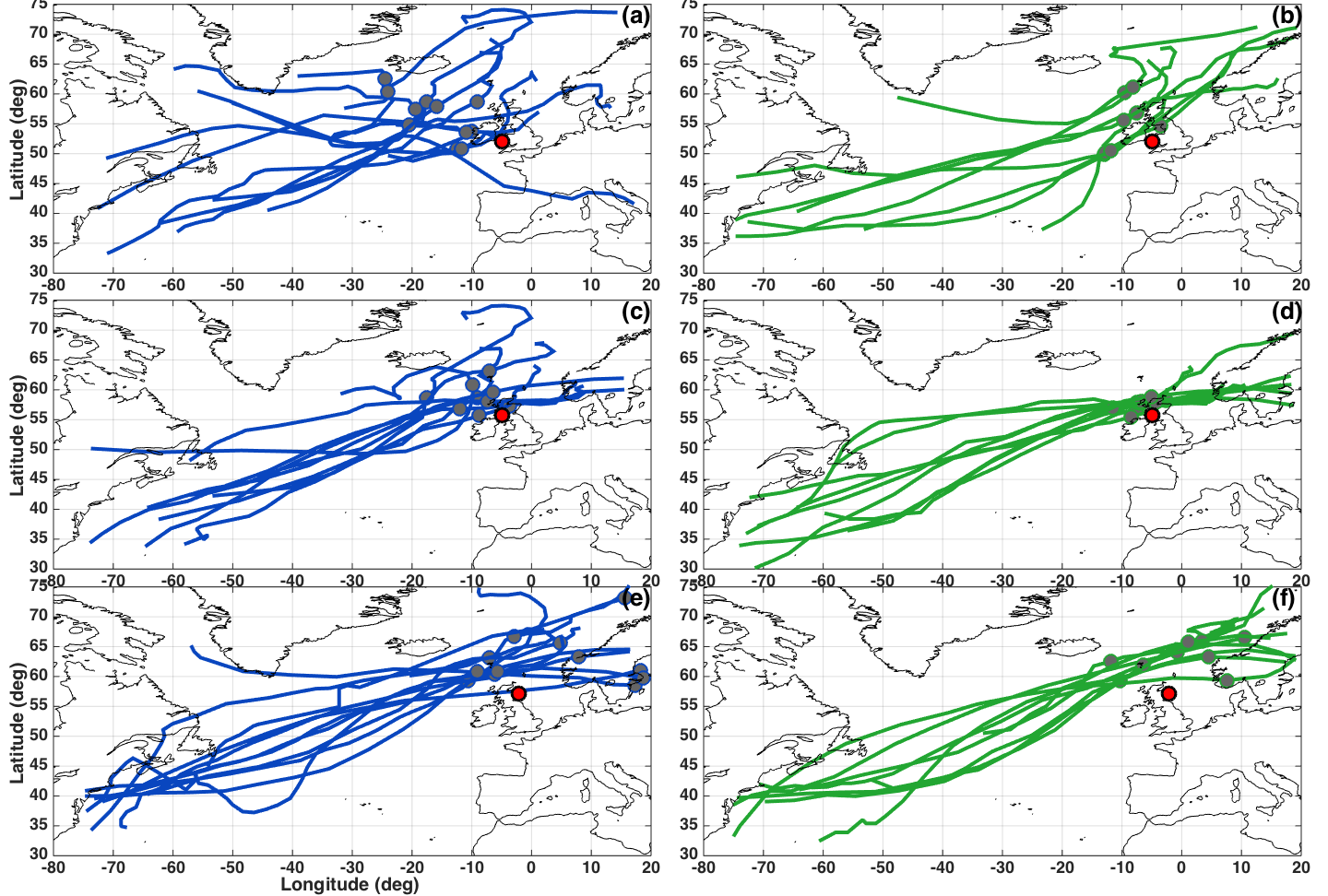 |
| --- |
| **Supplementary Figure 7:** The tracks of the storms that generated (a,c,e) sea level and (b,d,f) skew surge events that reached or exceed the 1 in 5 year return level at: (a,b) Fishguard; (c,d) Millport; (d,e) Aberdeen. The grey dots indicate the location of the storm centre at time of highest sea level or skew surge, for each storm. The red dots show the location of the tide gauge in question. |

| 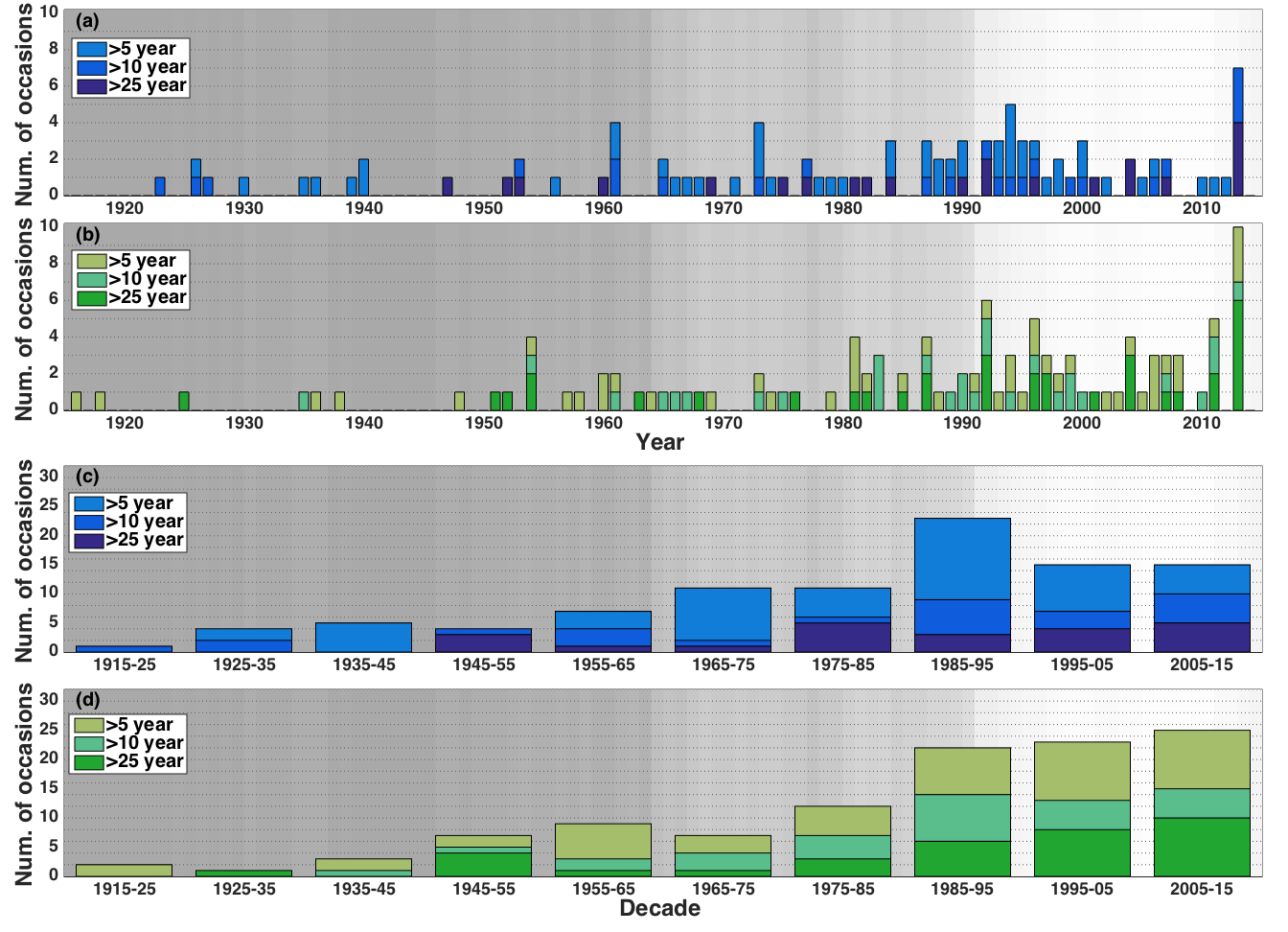 |
| --- |
| **Supplementary Figure 8:** Number of (a) sea level and (b) skew surge events per season (July to July) for different return level thresholds. Number of (c) sea level and (d) skew surge events per decade for different return level thresholds. The grey shading indicates the number of sites for which data is available for each year – the lighter the grey the more sites for which data is available. |

| 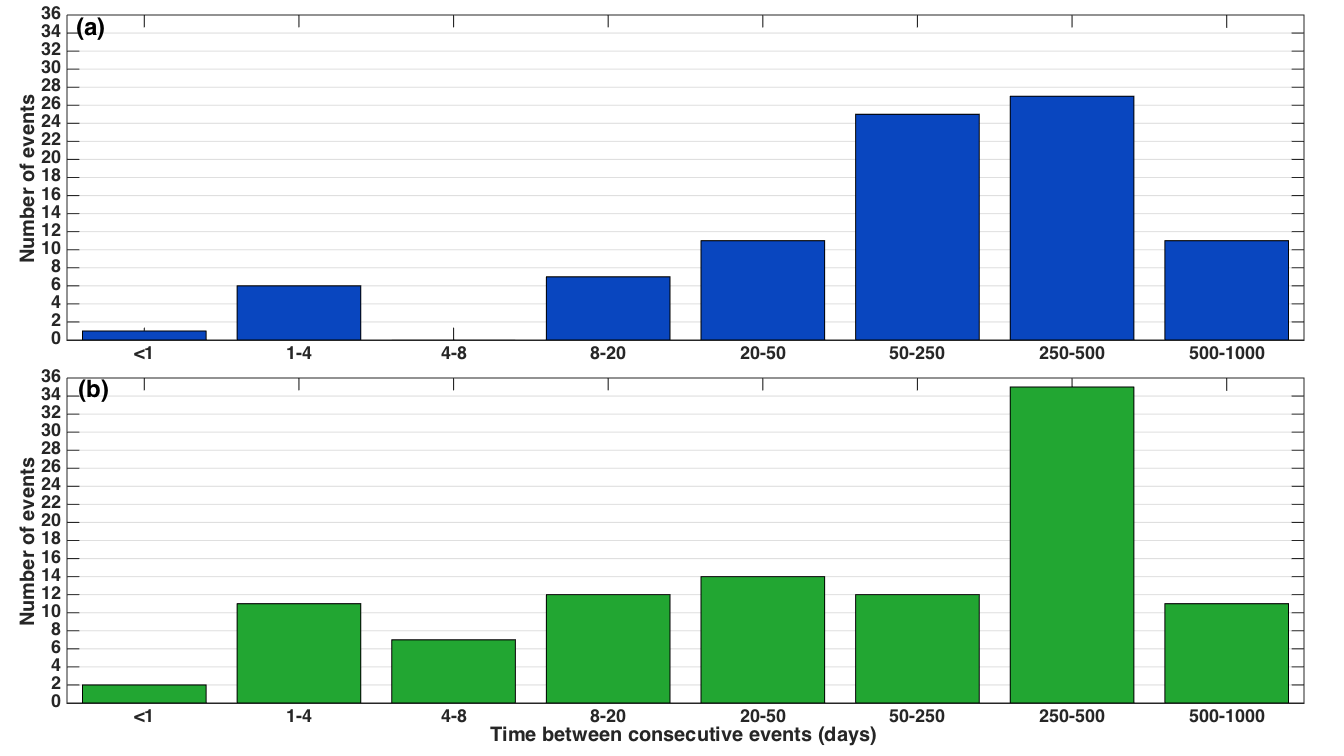 |
| --- |
| **Supplementary Figure 9:** The number of events with specific cluster time intervals for (a) sea level and (b) skew surge events. |

| 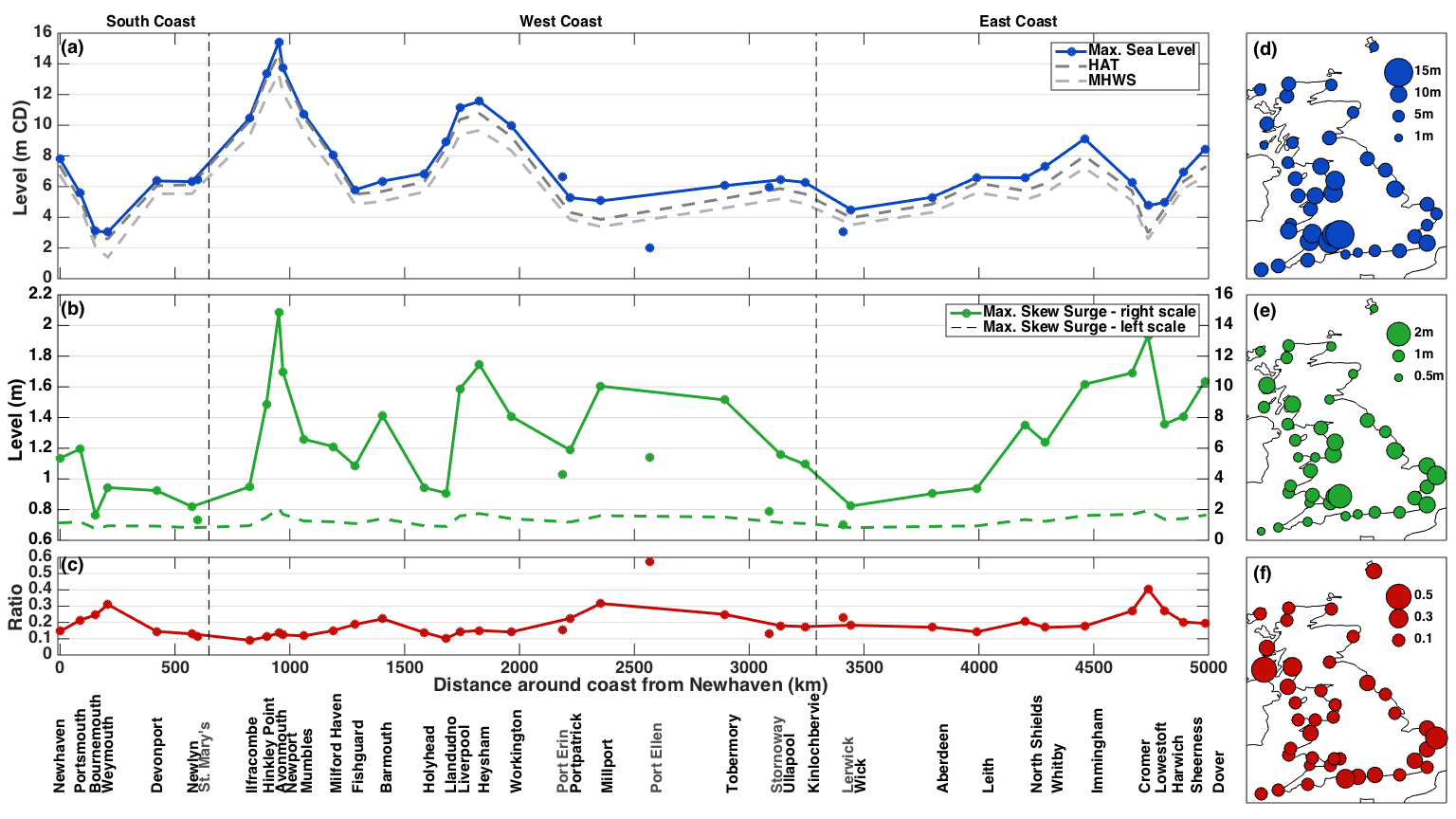 |
| --- |
| **Supplementary Figure 10:** (a,d) Maximum sea level; (b,e) maximum skew surge; and (c,f) the ratio of maximum skew surge to sea level; plotted relative to the distance around the coast of the UK from Newhaven (a,b,c) and on a map of the UK (d,e,f). The circles not lying on the line in (a), (b) and (c) show the results for the tide gauge locations that are located on islands away from the main land coastline. |

| 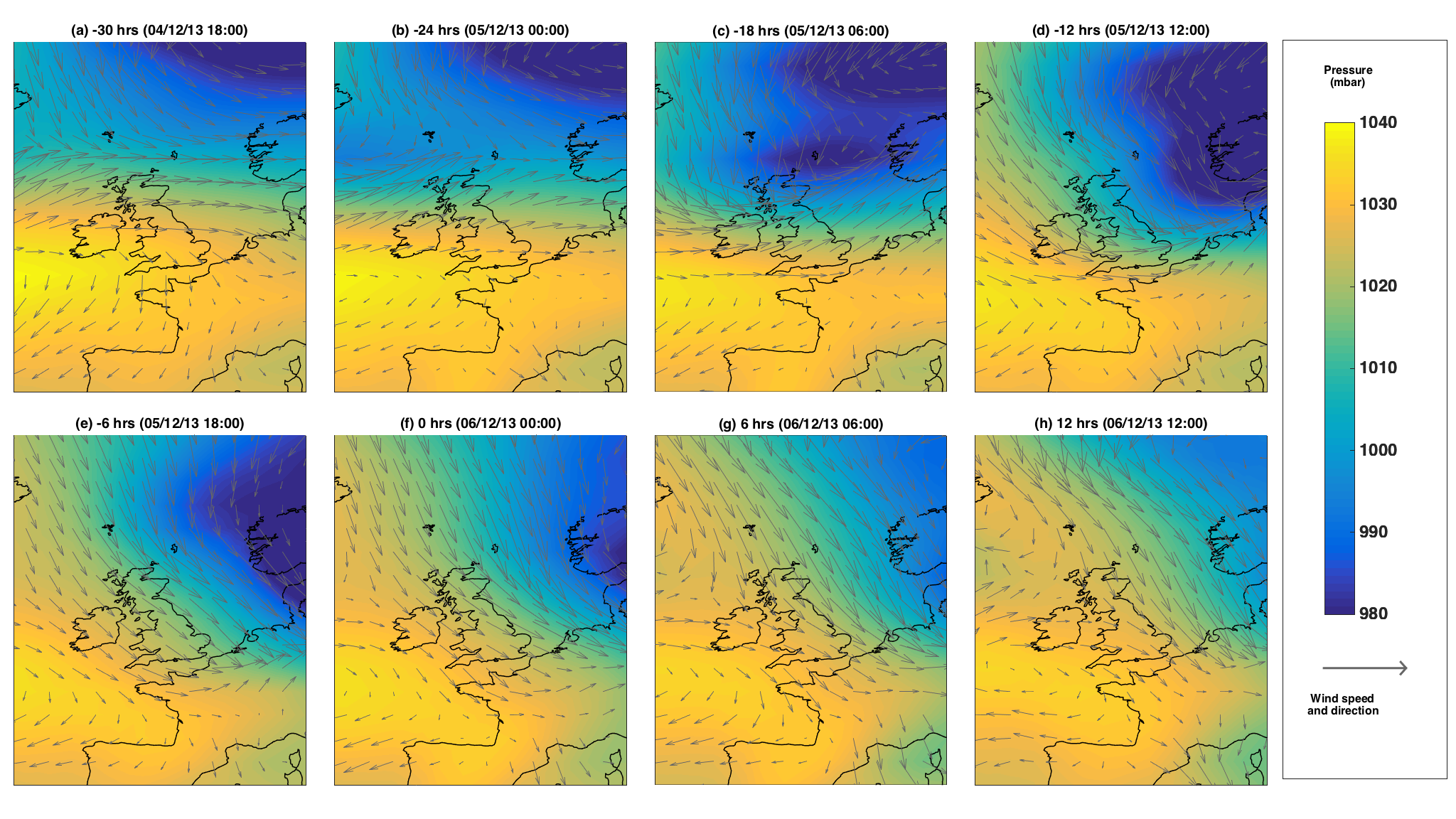 |
| --- |
| **Supplementary Figure 11:** Atmospheric pressure and wind speed every 6 hours during the 5-6 December 2013 extreme sea level event. As the low-pressure system crosses north of Scotland, strong winds are directed into the Irish Sea through the northern channel, elevating surge levels in the Irish Sea. Typically, the North Sea experiences a negative surge at this time, due to the southerly winds at the front of the storm. Once the storm crosses into Scandinavia, the wind direction changes, and strong northerly winds generate a large storm surge, which propagates into the North Sea. |

**Appendix A: Matlab Script**

%Ivan Haigh

%July 2016

%

%This matlab script produces the main results figures in the paper 'Spatial footprint

%and temporal clustering analysis of extreme sea level and storm surge events

%around the coastline of the UK'. Note: I use a higher resolution land

%boundary for the figures in the actual paper.

clear, clc

%-------------------------

%% 1.) Load in data

%load in sea level data

[d m y h mi s HW_all(:,2) HW_all(:,3) HW_all(:,4) HW_all(:,5) HW_all(:,6) HW_all(:,7)] = ...

textread('water_levels.csv','%d/%d/%d %d:%d:%d,%f,%f,%f, %f,%d,%d','headerlines',6);

HW_all(:,1) = datenum(y,m,d,h,mi,s);

clear d m y h mi s

%load in sea level storm tracks

[d m y h mi s HW_ST(:,2) HW_ST(:,3) HW_ST(:,4) HW_ST(:,5)] = ...

textread('water_level_storm_tracks.csv','%d/%d/%d %d:%d:%d,%f,%f,%f,%d','headerlines',6);

HW_ST(:,1) = datenum(y,m,d,h,mi,s);

%load in skew surge data

[d m y h mi s SK_all(:,2) SK_all(:,3) SK_all(:,4) SK_all(:,5) SK_all(:,6) SK_all(:,7)] = ...

textread('skew_surges.csv','%d/%d/%d %d:%d:%d,%f,%f,%f, %f,%d,%d','headerlines',6);

SK_all(:,1) = datenum(y,m,d,h,mi,s);

clear d m y h mi s

%load in skew surge storm tracks

[d m y h mi s SK_ST(:,2) SK_ST(:,3) SK_ST(:,4) SK_ST(:,5)] = ...

textread('skew_surge_storm_tracks.csv','%d/%d/%d %d:%d:%d,%f,%f,%f,%d','headerlines',6);

SK_ST(:,1) = datenum(y,m,d,h,mi,s);

%load site information

[N,X,Y,I] = textread('sites.csv','%3s,%f,%f,%d','headerlines',6);

FN = {'Newhaven' ;'Portsmouth' ;'Bournemouth' ;'Weymouth' ;'Devonport' ;...

'Newlyn' ;'St. Mary''s';'Ilfracombe' ;'Hinkley Point';'Avonmouth' ;...

'Newport' ;'Mumbles' ;'Milford Haven';'Fishguard' ;'Barmouth' ;...

'Holyhead' ;'Llandudno' ;'Liverpool' ;'Heysham' ;'Workington';...

'Port Erin ';'Portpatrick';'Millport' ;'Port Ellen' ;'Tobermory' ;...

'Stornoway' ;'Ullapool' ;'Kinlochbervie';'Lerwick' ;'Wick' ;...

'Aberdeen' ;'Leith' ;'North Shields';'Whitby' ;'Immingham' ;...

'Cromer' ;'Lowestoft' ;'Harwich' ;'Sheerness' ;'Dover' };

%load data availability information

[DQ] = csvread('data_availability.csv',6,0);

Yr = DQ(:,1);

DQ(:,1) = [];

i = find(DQ==0);

DQ(i) = NaN;

%load land

load coast;

L(:,1) = long;

L(:,2) = lat;

%-------------------------

%% 2.) Extract events - i.e. highest return period for each event

%Sea level

n = max(HW_all(:,7));

for i = 1:n

j = find(HW_all(:,7)==i);

[k,m]= max(HW_all(j,2));

HW_events(i,:) = HW_all(j(m),:);

clear j k m

end

clear i n

%Skew surge

n = max(SK_all(:,7));

for i = 1:n

j = find(SK_all(:,7)==i);

[k,m]= max(SK_all(j,2));

SK_events(i,:) = SK_all(j(m),:);

clear j k m

end

clear i n

%-------------------------

%% 3.) Figure 1 in paper

%work out max and minimum years and data lengths

for i = 1:length(X)

j = find(~isnan(DQ(:,i)));

YR(i,1) = Yr(j(1));

YR(i,2) = Yr(j(end));

YR(i,3) = length(j);

YR(i,4) = YR(i,2)- YR(i,1)+1;

clear j

end

% dot sizes for data length

DL = 100:-10:10;

sz = (length(DL):-1:1)*3+10;

figure('unit','normalized','position',[0.1 0.2 0.7 0.7])

axes('unit','normalized','position',[-0.01 0.07 0.51 0.91])

hold on

cm = colormap(parula(length(DL)+5));

cm = cm(end:-1:1,:);

colormap(cm);

plot(L(:,1),L(:,2),'k','linewidth',1.0);

for i = 1:length(DL)-1;

j = find(YR(:,3)<= DL(i) & YR(:,3)> DL(i+1));

plot(X(j),Y(j),'or','markersize',sz(i),'markerfacecolor',cm(i,:),'color','k');

plot(1,59.8-(i*0.6),'or','markersize',sz(i),'markerfacecolor',cm(i,:),'color','k');

text(1.5,59.8-(i*0.6),[num2str(DL(i+1)),'-',num2str(DL(i)),' '],'fontweight','bold','fontsize',18);

end

for i = 1:length(FN);

text(X(i)-0.1,Y(i),[num2str(i)],'fontweight','bold','fontsize',18,'color','b');

end

text(-0.1,60,'Length of data (years) ','fontweight','bold','fontsize',16,'color','k');

set(gca,'fontweight','bold','fontsize',18);

axis equal

set(gca,'xlim',[-7 3],'ylim',[49 61]);

box on

xlabel('Longitude (deg)','fontweight','bold','fontsize',20);

ylabel('Latitude (deg)','fontweight','bold','fontsize',20);

text(-6.7,60.6,'(a) ','fontweight','bold','fontsize',28,'color','k');

axes('unit','normalized','position',[0.47 0.07 0.43 0.91])

hold on

plot(Yr,DQ,'s','color',[0.3 0.3 0.3],'markerfacecolor',[0.3 0.3 0.3],'markersize',15)

set(gca,'fontweight','bold','fontsize',18);

set(gca,'xlim',[1914 2015],'xtick',[1915:10:2015],'ylim',[0.5 40.5],'ytick',[]);

box on

xlabel('Year ','fontweight','bold','fontsize',24);

for i = 1:length(FN)

if i <10

text(2016,i,[' ',num2str(i),'. ',FN{i}],'fontweight','bold','fontsize',18);

else

text(2016,i,[num2str(i),'. ',FN{i}],'fontweight','bold','fontsize',18);

end

end

plot([min(Y)-1 max(Y)+10],[7.5 7.4],'--k');

plot([min(Y)-1 max(Y)+10],[28.5 28.4],'--k');

text(1916,39,'(b) ','fontweight','bold','fontsize',28,'color','k');

%-----------------------

%% 4.) Figure 2 in paper

%creat axis for figure

co = 0;

for i = 1915:10:2015

co = co+1;

xt(co,1) = datenum(i,1,1,0,0,0);

end

clear co i

%calulate number of sites for which data is available each year

i = find(DQ>=1);

DQ(i) = 1;

i = find(isnan(DQ));

DQ(i)= 0;

dq = sum(DQ')';

clear i

%find numnber of skew surge events that are the same as sea level events

for i = 1:length(HW_events(:,1));

j = find(HW_events(i,1)==SK_events(:,1));

D(i,1) = i;

if isempty(j)

D(i,2) = NaN;

D(i,3) = NaN;

else

D(i,2) = j;

D(i,3) = 1;

end

clear j

end

le1 = nansum(D(:,3));

le2 = length(SK_events(:,1))-le1;

XX = [le1,le2];

explode = [1 0];

figure('units','normalized','position',[0.1 0.2 0.7 0.7]);

axes('units','normalized','position',[0.05 0.7 0.93 0.28]);

hold on

bar(HW_events(:,1),HW_events(:,2),300,'edgecolor','k','facecolor',[9 70 191]/255)

set(gca,'xlim',[datenum(1915,1,1,0,0,0) datenum(2015,1,1,0,0,0)],'xtick',xt,'xticklabel',datestr(xt,10));

set(gca,'YScale','log');

set(gca,'ylim',[5 1000],'ytick',[5 10 50 100 500 1000],'yticklabel',[5 10 50 100 500 1000])

set(gca,'fontweight','bold','fontsize',18,'ygrid','on');

ylabel('Return period (years) ','fontweight','bold','fontsize',20)

box on

text(datenum(1915,3,1,0,0,0),800,['(a) '],'fontweight','bold','fontsize',20);

axes('units','normalized','position',[0.05 0.38 0.93 0.28]);

hold on

bar(SK_events(:,1),SK_events(:,2),300,'edgecolor','k','facecolor',[34 166 50]/255)

set(gca,'xlim',[datenum(1915,1,1,0,0,0) datenum(2015,1,1,0,0,0)],'xtick',xt,'xticklabel',datestr(xt,10));

set(gca,'YScale','log');

set(gca,'ylim',[5 1000],'ytick',[5 10 50 100 500 1000],'yticklabel',[5 10 50 100 500 1000])

set(gca,'fontweight','bold','fontsize',18,'ygrid','on');

ylabel('Return period (years) ','fontweight','bold','fontsize',20)

box on

text(datenum(1915,3,1,0,0,0),800,['(b) '],'fontweight','bold','fontsize',20);

axes('units','normalized','position',[0.05 0.06 0.93 0.28]);

hold on

bar(Yr,dq,1,'facecolor',[0.3 0.3 0.3],'edgecolor',[0.7 0.7 0.7])

set(gca,'xlim',[1915 2015],'xtick',[1915:10:2015]);

set(gca,'ylim',[0 40],'ytick',[0:5:40])

set(gca,'fontweight','bold','fontsize',18,'ygrid','on');

xlabel('Year','fontweight','bold','fontsize',20)

ylabel('Number of sites ','fontweight','bold','fontsize',20)

box on

text(1915.3,38,['(c) '],'fontweight','bold','fontsize',20);

fill([1922 1940 1940 1922 1922],[8 8 38 38 8],'w');

plot([1922 1940 1940 1922 1922],[8 8 38 38 8],'k');

text(1922.3,36,['(d) '],'fontweight','bold','fontsize',20);

axes('units','normalized','position',[0.07 0.08 0.26 0.3]);

hold on

P = pie3(XX,explode);

axis equal

axis off

view([-101 48])

colormap([[9 70 191]/255; [34 166 50]/255]);

set(P(4),'fontweight','bold','fontsize',20,'position',[-0.59 1.4 0.3500],'color',[9 70 191]/255)

set(P(8),'fontweight','bold','fontsize',20,'color',[34 166 50]/255)

clear XX

%-------------------------

%% 5.) Figure 3 in paper

%calculate number of sites impacted for Sea level

n = max(HW_all(:,7));

for i = 1:n

j = find(HW_all(:,7)==i);

u= unique( HW_all(j,6));

HW_events(i,8) = length(u);

clear ju

end

clear i n

%lables for figures

T = {'a';'b';'c';'d';'e';'f';'g';'h';'i';'j';'k';'l';'m';'n';'o';'p';'q';'r';'s';'t';'u'};

%event numbers

E2 = [50 12 42 44 45 ...

02 08 04 16 17 ...

07 09 23 55 55 ....

01 06 31 33 11];

%size of dots

sz = [17 20 25 30 35 40 50 60 75];

rp = [5 10 20 50 75 100 500 750 1000];

sz = sz./1.3;

%location of figures

AX(1 ,:) = [-0.33 0.755 0.86 0.24];

AX(2 ,:) = [-0.13 0.755 0.86 0.24];

AX(3 ,:) = [ 0.07 0.755 0.86 0.24];

AX(4 ,:) = [ 0.27 0.755 0.86 0.24];

AX(5 ,:) = [ 0.47 0.755 0.86 0.24];

AX(6 ,:) = [-0.33 0.505 0.86 0.24];

AX(7 ,:) = [-0.13 0.505 0.86 0.24];

AX(8 ,:) = [ 0.07 0.505 0.86 0.24];

AX(9 ,:) = [ 0.27 0.505 0.86 0.24];

AX(10,:) = [ 0.47 0.505 0.86 0.24];

AX(11,:) = [-0.33 0.255 0.86 0.24];

AX(12,:) = [-0.13 0.255 0.86 0.24];

AX(13,:) = [ 0.07 0.255 0.86 0.24];

AX(14,:) = [ 0.27 0.255 0.86 0.24];

AX(15,:) = [ 0.47 0.255 0.86 0.24];

AX(16,:) = [-0.33 0.005 0.86 0.24];

AX(17,:) = [-0.13 0.005 0.86 0.24];

AX(18,:) = [ 0.07 0.005 0.86 0.24];

AX(19,:) = [ 0.27 0.005 0.86 0.24];

AX(20,:) = [ 0.47 0.005 0.86 0.24];

col(1,:) = [9 100 175]/255;

col(2,:) = [9 70 190]/255;

col(3,:) = [9 70 225]/255;

col(4,:) = [9 90 255]/255;

figure('units','normalized','position',[0.1 0.2 0.6 0.8]);

for e = 1:20;

ee = E2(e);

i = find(HW_all(:,7)==ee);

D = HW_all(i,:);

co = 0;

for i = 1:40

j = find(D(:,6)== i);

if isempty(j)

DD(i,1:7) = NaN;

DD(i,7) = i;

else

DD(i,:) = D(j(1),:);

end

end

D =DD;

clear DD i j co

axes('units','normalized','position',AX(e,:));

hold on

plot(L(:,1), L(:,2),'k','linewidth',1);

j = find(D(:,3)==0.9);

plot(X(j),Y(j),'sk','markersize',6,'markerfacecolor',[0.4 0.4 0.4],'linewidth',1,'color','k');

j = find(isnan(D(:,3)));

plot(X(j),Y(j),'+k','markersize',7,'markerfacecolor',[0.4 0.4 0.4],'linewidth',3,'color','k');

for i = 1:length(rp)-1;

j = find(D(:,3)>= rp(i) & D(:,3)<rp(i+1));

if e<6

plot(X(j),Y(j),'ok','markersize',sz(i),'markerfacecolor',col(1,:),'linewidth',1,'color','k');

elseif e>5 & e<11

plot(X(j),Y(j),'ok','markersize',sz(i),'markerfacecolor',col(2,:),'linewidth',1,'color','k');

elseif e>5 & e<11

plot(X(j),Y(j),'ok','markersize',sz(i),'markerfacecolor',col(3,:),'linewidth',1,'color','k');

else

plot(X(j),Y(j),'ok','markersize',sz(i),'markerfacecolor',col(4,:),'linewidth',1,'color','k');

end

clear j

end

axis equal

axis([-8.7 2.8 49.5 60.8])

set(gca,'xtick',[],'ytick',[]);

box on

set(gca,'fontweight','bold','fontsize',16);

text(-8.5,60,['(',T{e},') ',num2str(ee),' (',num2str(HW_events(ee,8)),') ' ],'fontweight','bold','fontsize',18)

clear D D2 ee i

end

%key

axes('units','normalized','position',AX(15,:));

hold on

of = 1.4;

for i = 2:length(rp);

plot(0.9,53.8+(i./of),'ok','markersize',sz(i),'markerfacecolor',[9 70 191]/255,'linewidth',1,'color','k');

if i==1

text(2,53.8+(i./of),['<',num2str(rp(i)),' yrs'],'fontweight','bold','fontsize',16);

else

text(2,53.8+(i./of),[num2str(rp(i-1)),'-',num2str(rp(i)),' yrs'],'fontweight','bold','fontsize',16);

end

end

plot(0.9,54.6,'sk','markersize',6,'markerfacecolor',[0.4 0.4 0.4],'linewidth',1);

text(2,54.6,['< 5 yrs'],'fontweight','bold','fontsize',16);

plot(0.9,54.0,'+k','markersize',7,'markerfacecolor','k','linewidth',3);

text(2,54.0,['no data'],'fontweight','bold','fontsize',16);

axis equal

axis([-1.15 6.35 53.7 61])

set(gca,'xtick',[],'ytick',[]);

box on

%-------------------------

%% 6.) Figure 4 in paper

%define axis limits for figure

lat = [28 87];

long =[-80 20];

figure('units','normalized','position',[0.1 0.2 0.7 0.7]);

axes('position',[-0.22 0.52 0.95 0.43])

hold on

for e = 1:5

ee = E2(e);

j = find(HW_ST(:,5)==ee);

plot(HW_ST(j,2),HW_ST(j,3),'-k','linewidth',4,'color',col(1,:));

diff = abs(HW_ST(j,1)-HW_events(ee,1));

[a,b] = min(diff);

plot(HW_ST(j(b),2),HW_ST(j(b),3),'or','linewidth',2,'color',col(1,:),...

'markerfacecolor',[0.4 0.4 0.4],'markersize',15);

clear j a b diff

end

plot(L(:,1),L(:,2),'-k','linewidth',1);

%plot(x(s),y(s),'ok','linewidth',2,'markersize',15,'color','g','markerfacecolor',[0.8 0.8 0.8]);

grid

axis equal

axis([long(1) long(2) lat(1) lat(2)]);

set(gca,'xtick',[],'ytick',[])

set(gca,'fontweight','bold','fontsize',18);

box on

title('(a) Category 1 ','fontweight','bold','fontsize',24);

axes('position',[0.27 0.52 0.95 0.43])

hold on

for e = 6:10

ee = E2(e);

j = find(HW_ST(:,5)==ee);

plot(HW_ST(j,2),HW_ST(j,3),'-k','linewidth',4,'color',col(2,:));

diff = abs(HW_ST(j,1)-HW_events(ee,1));

[a,b] = min(diff);

plot(HW_ST(j(b),2),HW_ST(j(b),3),'or','linewidth',2,'color',col(2,:),...

'markerfacecolor',[0.4 0.4 0.4],'markersize',15);

clear j a b diff

end

plot(L(:,1),L(:,2),'-k','linewidth',1);

%plot(x(s),y(s),'ok','linewidth',2,'markersize',15,'color','g','markerfacecolor',[0.8 0.8 0.8]);

grid

axis equal

axis([long(1) long(2) lat(1) lat(2)]);

set(gca,'xtick',[],'ytick',[])

set(gca,'fontweight','bold','fontsize',18);

box on

title('(b) Category 2 ','fontweight','bold','fontsize',24);

axes('position',[-0.22 0.03 0.95 0.43])

hold on

for e = 11:14

ee = E2(e);

j = find(HW_ST(:,5)==ee);

plot(HW_ST(j,2),HW_ST(j,3),'-k','linewidth',4,'color',col(3,:));

diff = abs(HW_ST(j,1)-HW_events(ee,1));

[a,b] = min(diff);

plot(HW_ST(j(b),2),HW_ST(j(b),3),'or','linewidth',2,'color',col(3,:),...

'markerfacecolor',[0.4 0.4 0.4],'markersize',15);

clear j a b diff

end

plot(L(:,1),L(:,2),'-k','linewidth',1);

%plot(x(s),y(s),'ok','linewidth',2,'markersize',15,'color','g','markerfacecolor',[0.8 0.8 0.8]);

grid

axis equal

axis([long(1) long(2) lat(1) lat(2)]);

set(gca,'xtick',[],'ytick',[])

set(gca,'fontweight','bold','fontsize',18);

box on

title('(c) Category 3 ','fontweight','bold','fontsize',24);

axes('position',[0.27 0.03 0.95 0.43])

hold on

for e = 15:20

ee = E2(e);

j = find(HW_ST(:,5)==ee);

plot(HW_ST(j,2),HW_ST(j,3),'-k','linewidth',4,'color',col(4,:));

diff = abs(HW_ST(j,1)-HW_events(ee,1));

[a,b] = min(diff);

plot(HW_ST(j(b),2),HW_ST(j(b),3),'or','linewidth',2,'color',col(4,:),...

'markerfacecolor',[0.4 0.4 0.4],'markersize',15);

clear j a b diff

end

plot(L(:,1),L(:,2),'-k','linewidth',1);

%plot(x(s),y(s),'ok','linewidth',2,'markersize',15,'color','g','markerfacecolor',[0.8 0.8 0.8]);

grid

axis equal

axis([long(1) long(2) lat(1) lat(2)]);

set(gca,'xtick',[],'ytick',[])

set(gca,'fontweight','bold','fontsize',18);

box on

title('(d) Category 4 ','fontweight','bold','fontsize',24);

%-------------------------

%% 7.) Figure 5 in paper

%events above certain threshold

k1 = find(HW_events(:,3)>10);

k2 = find(HW_events(:,3)>25);

m1 = find(SK_events(:,2)>10);

m2 = find(SK_events(:,2)>25);

%Get out months

[yy,mm,dd,hh,mii,ss] = datevec(HW_events(:,1));

[yysk,mmsk,ddsk,hhsk,miisk,sssk] = datevec(SK_events(:,1));

figure('units','normalized','position',[0.1 0.2 0.7 0.7]);

%Sea Level

axes('units','normalized','position',[0.05 0.54 0.93 0.43]);

hold on

h1 = hist(mm ,0.5:1:12.5);

h2 = hist(mm(k1),0.5:1:12.5);

h3 = hist(mm(k2),0.5:1:12.5);

bar([0.5:1:12.5],[h1],'facecolor',[0.0723 0.4887 0.8467]);

bar([0.5:1:12.5],[h2],'facecolor',[0.0591 0.3598 0.8683]);

bar([0.5:1:12.5],[h3],'facecolor',[0.2081 0.1663 0.5292]);

set(gca,'xlim',[0 12],'xtick',[0.5:1:11.5],'xticklabel',{'Jan','Feb','Mar','Apr','May','Jun','Jul','Aug','Sep','Oct','Nov','Dec'});

set(gca,'fontweight','bold','fontsize',18,'ygrid','on');

set(gca,'ylim',[0 38],'ytick',[0:5:50]);

%xlabel('Month ','fontweight','bold','fontsize',20)

ylabel('Number of occasions ','fontweight','bold','fontsize',20)

box on

LL = legend('>5 year','>10 year','>25 year','location','northeast');

set(LL,'fontweight','bold','fontsize',20);

text(0.05,36.5,['(a) '],'fontweight','bold','fontsize',20);

%Skew surge

axes('units','normalized','position',[0.05 0.06 0.93 0.43]);

hold on

h1 = hist(mmsk ,0.5:1:12.5);

h2 = hist(mmsk(m1),0.5:1:12.5);

h3 = hist(mmsk(m2),0.5:1:12.5);

bar([0.5:1:12.5],[h1],'facecolor',[0.6473 0.7456 0.4188]);

bar([0.5:1:12.5],[h2],'facecolor',[0.3482 0.7424 0.5473]);

bar([0.5:1:12.5],[h3],'facecolor',[34 166 50]/255);

set(gca,'xlim',[0 12],'xtick',[0.5:1:11.5],'xticklabel',{'Jan','Feb','Mar','Apr','May','Jun','Jul','Aug','Sep','Oct','Nov','Dec'});

set(gca,'fontweight','bold','fontsize',18,'ygrid','on');

set(gca,'ylim',[0 38],'ytick',[0:5:50]);

xlabel('Month ','fontweight','bold','fontsize',20)

ylabel('Number of occasions ','fontweight','bold','fontsize',20)

box on

LL = legend('>5 year','>10 year','>25 year','location','northeast');

set(LL,'fontweight','bold','fontsize',20);

text(0.05,36.5,['(b) '],'fontweight','bold','fontsize',20);

clear LL

%-------------------------

%% 8.) Figure 6 in paper

%Calculate time between sucessive events - Sea level

[a,b] = sort(HW_events(:,1));

HW_events = HW_events(b,:);

clear a b

HW_events(2:end,9) = abs(diff(HW_events(:,1)));

HW_events(1,9) = 1000;

clear xx

xx = [0 1.1 4.1 7.9 20 50 250 500 1000]';

for i = 1:length(xx)-1

j = find(HW_events(:,9)>=xx(i) & HW_events(:,9)<xx(i+1));

H(i) = length(j);

clear j

end

clear i

%Calculate time between sucessive events - Skew surges

[a,b] = sort(SK_events(:,1));

SK_events = SK_events(b,:);

clear a b

SK_events(2:end,9) = abs(diff(SK_events(:,1)));

SK_events(1,9) = 1000;

clear xxsk

xxsk = [0 1.1 4.1 7.9 20 50 250 500 1000]';

for i = 1:length(xxsk)-1

j = find(SK_events(:,9)>=xxsk(i) & SK_events(:,9)<xxsk(i+1));

Hsk(i) = length(j);

clear j

end

clear i

%labels for axis

co = 0;

for i = 1915:10:2015

co = co+1;

xt(co,1) = datenum(i,1,1,0,0,0);

end

clear co i

figure('units','normalized','position',[0.1 0.2 0.7 0.7]);

%Sea level

axes('units','normalized','position',[0.05 0.54 0.93 0.43]);

semilogy(HW_events(:,1),HW_events(:,9),'or','markersize',12,'markerfacecolor',[9 70 191]/255,'color',[9 70 191]/255);

hold on

for i = [0.5 1 2 3 5 8 10 100 250 500 1000]

plot([datenum(1914,1,1,0,0,0) datenum(2016,1,1,0,0,0)],[i i],':','color',[0.4 0.4 0.4]);

end

semilogy(HW_events(:,1),HW_events(:,9),'or','markersize',12,'markerfacecolor',[9 70 191]/255,'color',[9 70 191]/255);

set(gca,'xlim',[datenum(1915,1,1,0,0,0) datenum(2015,1,1,0,0,0)],'xtick',xt,'xticklabel',datestr(xt,10));

set(gca,'ylim',[0.5 1000],'ytick',[0.5 1 2 3 5 7 10 100 250 1000],'yticklabel',[0.5 1 2 3 5 7 10 100 250 1000],'ygrid','on');

%xlabel('Date ','fontweight','bold','fontsize',20);

ylabel('Time between consecutive events (days) ','fontweight','bold','fontsize',20);

box on

set(gca,'fontweight','bold','fontsize',18);

text(datenum(1915,3,1,0,0,0),700,['(a) '],'fontweight','bold','fontsize',20);

%Skew surge

axes('units','normalized','position',[0.05 0.06 0.93 0.43]);

semilogy(SK_events(:,1),SK_events(:,9),'or','markersize',12,'markerfacecolor',[34 166 50]/255,'color',[34 166 50]/255);

hold on

for i = [0.5 1 2 3 5 8 10 100 250 500 1000]

plot([datenum(1914,1,1,0,0,0) datenum(2016,1,1,0,0,0)],[i i],':','color',[0.4 0.4 0.4]);

end

semilogy(SK_events(:,1),SK_events(:,9),'or','markersize',9,'markerfacecolor',[34 166 50]/255,'color',[34 166 50]/255);

set(gca,'xlim',[datenum(1915,1,1,0,0,0) datenum(2015,1,1,0,0,0)],'xtick',xt,'xticklabel',datestr(xt,10));

set(gca,'ylim',[0.5 1000],'ytick',[0.5 1 2 3 5 7 10 100 250 1000],'yticklabel',[0.5 1 2 3 5 7 10 100 250 1000],'ygrid','on');

xlabel('Year ','fontweight','bold','fontsize',20);

ylabel('Time between consecutive events (days) ','fontweight','bold','fontsize',20);

box on

set(gca,'fontweight','bold','fontsize',18);

text(datenum(1915,3,1,0,0,0),700,['(b) '],'fontweight','bold','fontsize',20);

%-------------------------

%% 9.) Figure 7 in paper

%sort back by return period

[a,b] = sort(HW_events(:,1));

HW_events = HW_events(b,:);

clear a b

%Find events less than four days apart

E = find(HW_events(:,9)<4);

[a,b] = sort(HW_events(E,9));

E = E(b);

E(:,2) = HW_events(E(:,1)-1,7);

E(:,1) = HW_events(E(:,1),7);

clear a b;

[a,b] = sort(HW_events(:,7),'ascend');

HW_events = HW_events(b,:);

clear a b

%Labels

T = {'1a';'1b';'2a';'2b';'3a';'3b';'4a';'4b';'5a';'5b';'6a';'6b';'7a';'7b';'8a';'8b';'9a';'9b';'10a';'10b'};

%dot sizes

sz = [17 20 25 30 35 40 50 60 75];

rp = [5 10 20 50 75 100 500 750 1000];

sz = sz./1.3;

%axis locations

AX(1 ,:) = [-0.32 0.755 0.86 0.24];

AX(2 ,:) = [-0.07 0.755 0.86 0.24];

AX(3 ,:) = [-0.32 0.505 0.86 0.24];

AX(4 ,:) = [-0.07 0.505 0.86 0.24];

AX(5 ,:) = [-0.32 0.255 0.86 0.24];

AX(6 ,:) = [-0.07 0.255 0.86 0.24];

AX(7 ,:) = [-0.32 0.005 0.86 0.24];

AX(8 ,:) = [-0.07 0.005 0.86 0.24];

AX(9 ,:) = [ 0.20 0.755 0.86 0.24];

AX(10,:) = [ 0.45 0.755 0.86 0.24];

AX(11,:) = [ 0.20 0.505 0.86 0.24];

AX(12,:) = [ 0.45 0.505 0.86 0.24];

AX(13,:) = [ 0.20 0.255 0.86 0.24];

AX(14,:) = [ 0.45 0.255 0.86 0.24];

AX(15,:) = [ 0.20 0.005 0.86 0.24];

AX(16,:) = [ 0.45 0.005 0.86 0.24];

cooo = 0;

coo = 0;

figure('units','normalized','position',[0.1 0.1 0.6 0.8]);

for e = 1:length(E(:,1));

E(e,2);

m = find(HW_events(:,7)==E(e,2));

datestr(HW_events(m,1));

E(e,1);

n = find(HW_events(:,7)==E(e,1));

datestr(HW_events(n,1));

ee = E(e,2);

i = find(HW_all(:,7)==ee);

D = HW_all(i,:);

co = 0;

for i = 1:40

j = find(D(:,6)== i);

if isempty(j)

DD(i,1:7) = NaN;

DD(i,7) = i;

else

DD(i,:) = D(j(1),:);

end

end

coo= coo+1;

axes('units','normalized','position',AX(coo,:));

hold on

plot(L(:,1), L(:,2),'k','linewidth',1);

j = find(D(:,2)==0.9);

plot(X(j),Y(j),'sk','markersize',6,'markerfacecolor',[0.4 0.4 0.4],'linewidth',1,'color','k');

j = find(isnan(DD(:,2)));

plot(X(j),Y(j),'+k','markersize',7,'markerfacecolor',[0.4 0.4 0.4],'linewidth',3,'color','k');

for i = 1:length(rp)-1;

j = find(DD(:,2)>= rp(i) & DD(:,2)<rp(i+1));

plot(X(j),Y(j),'ok','markersize',sz(i),'markerfacecolor',[9 70 191]/255,'linewidth',1,'color','k');

clear j

end

axis equal

axis([-8.7 2.8 49.5 60.8])

set(gca,'xtick',[],'ytick',[]);

box on

set(gca,'fontweight','bold','fontsize',16);

cooo = cooo+1;

text(-8.5,60,['(',T{cooo},') ',num2str(ee),' (',num2str(HW_events(ee,8)),') ' ],'fontweight','bold','fontsize',18)

text(3.5,51,[num2str(ee),'. ',datestr(HW_events(ee,1),'dd/mm/yyyy HH:MM')],'rotation',90,'fontweight','bold','fontsize',16);

ee1 = ee;

ee = E(e,1);

text(4.5,51,[num2str(ee),'. ',datestr(HW_events(ee,1),'dd/mm/yyyy HH:MM')],'rotation',90,'fontweight','bold','fontsize',16);

text(5.5,51,[num2str(round((HW_events(ee,1)-HW_events(ee1,1)).*100)./100),' days'],'rotation',90,'fontweight','bold','fontsize',16);

i = find(HW_all(:,7)==ee);

D = HW_all(i,:);

co = 0;

for i = 1:40

j = find(D(:,6)== i);

if isempty(j)

DD(i,1:7) = NaN;

DD(i,7) = i;

else

DD(i,:) = D(j(1),:);

end

end

D =DD;

coo= coo+1;

axes('units','normalized','position',AX(coo,:));

hold on

plot(L(:,1), L(:,2),'k','linewidth',1);

j = find(DD(:,2)==0.9);

plot(X(j),Y(j),'sk','markersize',6,'markerfacecolor',[0.4 0.4 0.4],'linewidth',1,'color','k');

j = find(isnan(DD(:,2)));

plot(X(j),Y(j),'+k','markersize',7,'markerfacecolor',[0.4 0.4 0.4],'linewidth',3,'color','k');

for i = 1:length(rp)-1;

j = find(DD(:,2)>= rp(i) & DD(:,2)<rp(i+1));

plot(X(j),Y(j),'ok','markersize',sz(i),'markerfacecolor',[9 70 191]/255,'linewidth',1,'color','k');

clear j

end

axis equal

axis([-8.7 2.8 49.5 60.8])

set(gca,'xtick',[],'ytick',[]);

box on

set(gca,'fontweight','bold','fontsize',16);

cooo = cooo+1;

text(-8.5,60,['(',T{cooo},') ',num2str(ee),' (',num2str(HW_events(ee,8)),') ' ],'fontweight','bold','fontsize',18)

end

%key

axes('units','normalized','position',AX(16,:));

hold on

of = 1.4;

for i = 2:length(rp);

plot(0.9,53.8+(i./of),'ok','markersize',sz(i),'markerfacecolor',[9 70 191]/255,'linewidth',1,'color','k');

if i==1

text(2,53.8+(i./of),['<',num2str(rp(i)),' yrs'],'fontweight','bold','fontsize',16);

else

text(2,53.8+(i./of),[num2str(rp(i-1)),'-',num2str(rp(i)),' yrs'],'fontweight','bold','fontsize',16);

end

end

plot(0.9,54.6,'sk','markersize',6,'markerfacecolor',[0.4 0.4 0.4],'linewidth',1);

text(2,54.6,['< 5 yrs'],'fontweight','bold','fontsize',16);

plot(0.9,54.0,'+k','markersize',7,'markerfacecolor','k','linewidth',3);

text(2,54.0,['no data'],'fontweight','bold','fontsize',16);

axis equal

axis([-1.15 6.35 53.7 61])

set(gca,'xtick',[],'ytick',[]);

box on

%-------------------------

**Appendix B: Locations**


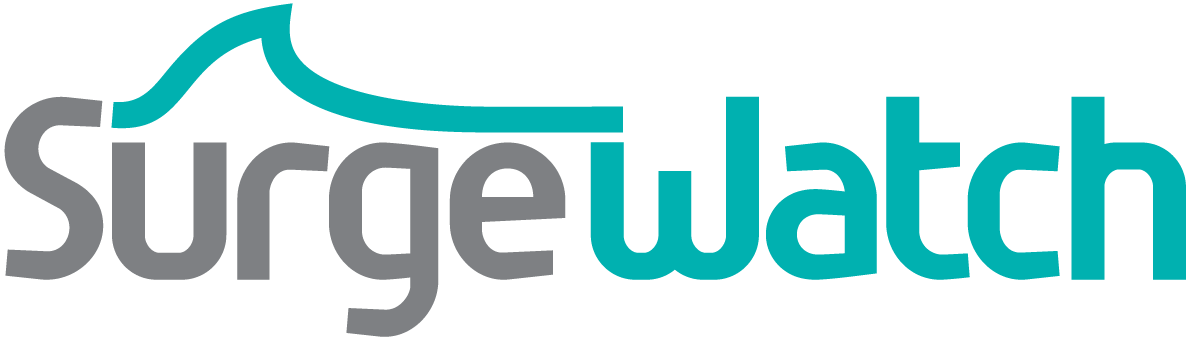
**LOCATION**

**** **B1. Newhaven**

*Chart Datum (CD) is 3.52m below Ordnance Datum Newlyn (ODN).*

| *Observational Period* |  | *Latitude & Longitude* |
| --- | --- | --- |
| ***1982-1987,1991-present*** |  | ***50° 46′ 54.4″ N, 00° 03′ 25.3″ E*** |

**Supplementary Table B1a:** High water levels (m CD) that reached or exceeded a 1 in 5 year return level at this site.

| **Date and time (GMT)** | **Return period (years)** | **Water**  **level (m CD)** | **Astronomical tide (m CD)** | **Skew surge (m)** |
| --- | --- | --- | --- | --- |
| 06/12/2013 01:15 | 39 | 7.79 | 7.00 | 0.73 |
| 02/02/1983 02:00 | 25 | 7.69 | 6.92 | 0.76 |
| 08/04/1985 01:00 | 11 | 7.61 | 7.24 | 0.38 |
| 31/01/1983 13:00 | 6 | 7.52 | 7.08 | 0.44 |

**SupplementaryTable B1b:** Skew surges (m) that reached or exceeded a 1 in 5 year return level at this site.

| **Date and time (GMT)** | **Return period (years)** | **Skew surge (m)** | **Water**  **level (m CD)** | **Astronomical tide (m CD)** |
| --- | --- | --- | --- | --- |
| 16/10/1987 05:00 | 180 | 1.14 | 5.77 | 4.57 |
| 21/02/1993 11:15 | 33 | 0.94 | 7.31 | 6.36 |
| 14/11/2002 06:45 | 9 | 0.80 | 6.23 | 5.41 |
| 02/02/1983 02:00 | 6 | 0.76 | 7.69 | 6.92 |
| 17/12/2004 15:30 | 6 | 0.75 | 6.89 | 6.08 |

| **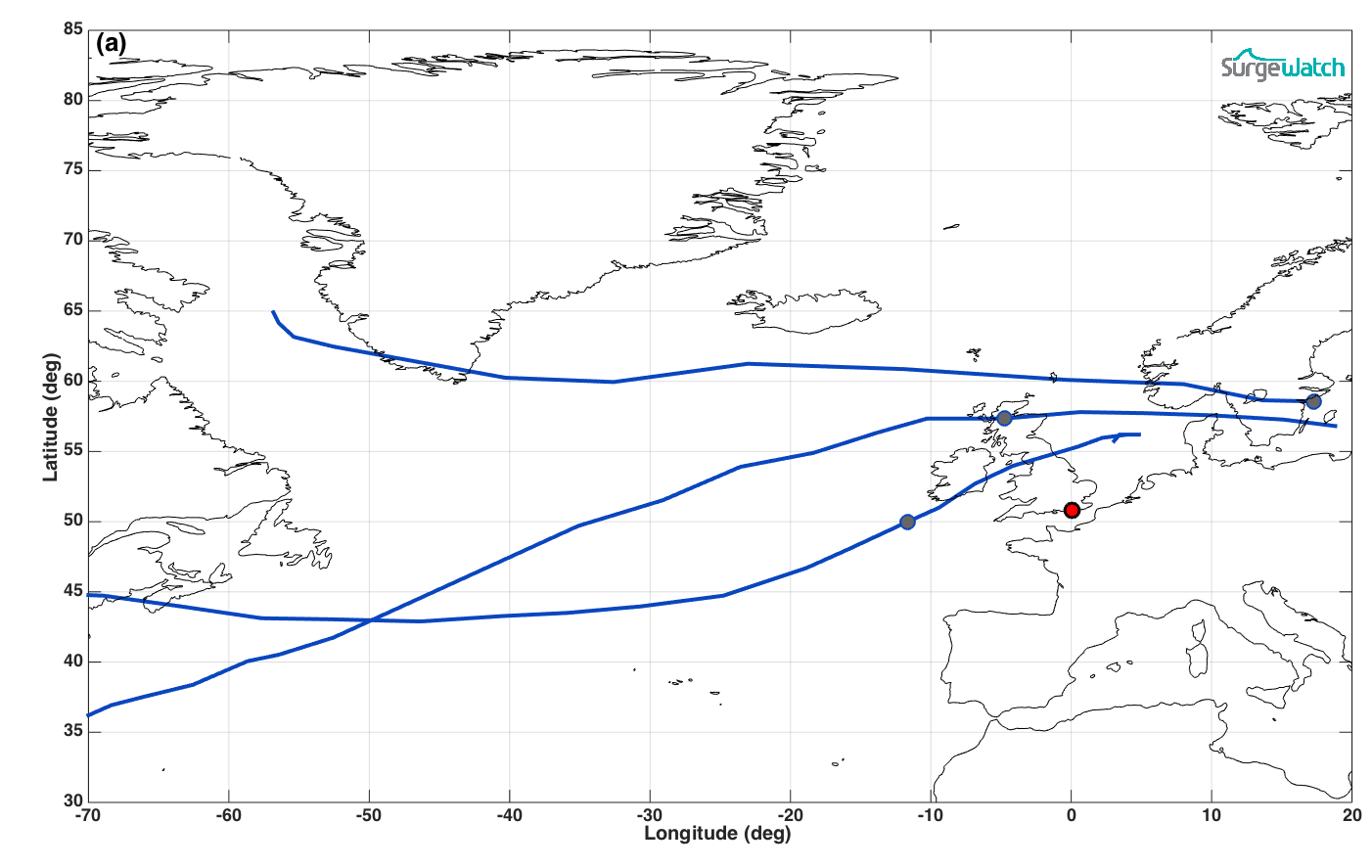** |
| --- |
| **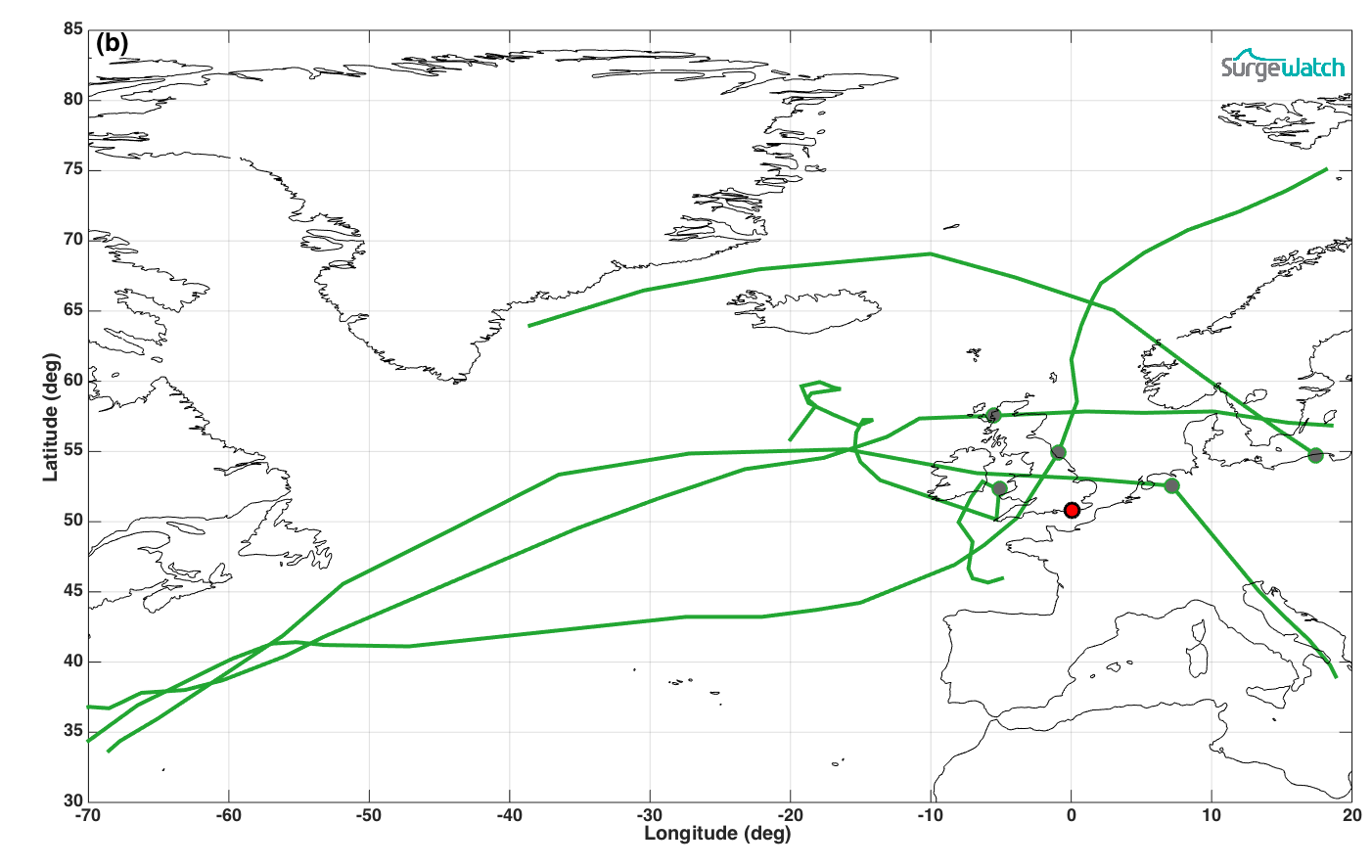** |
| **SupplementaryFigure B1:** Tracks of the storms that generated (a) high water levels and (b) skew surges that reached or exceeded the 1 in 5 year return level at Newhaven (location shown with the blue dot). The red dot indicates the location of the storm centre at the time of highest water level or skew surge. |


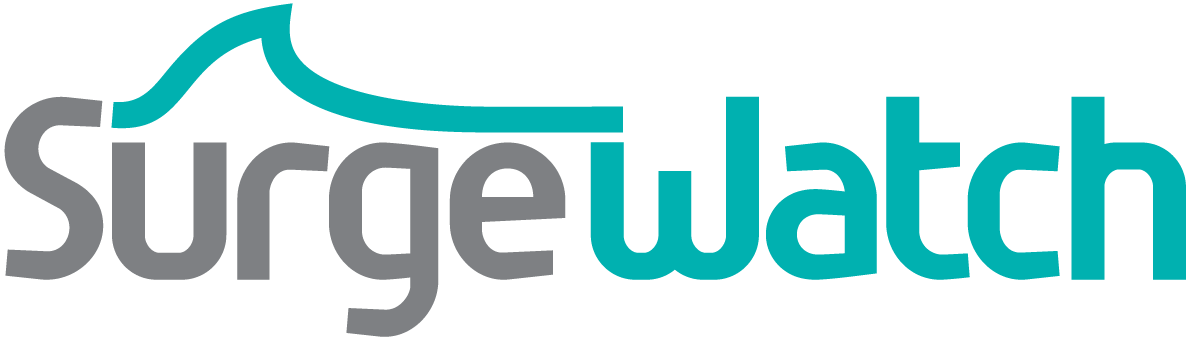
**LOCATION**

** B2.** **Portsmouth**

*Chart Datum (CD) is 2.73m below Ordnance Datum Newlyn (ODN).*

| *Observational Period* |  | *Latitude & Longitude* |
| --- | --- | --- |
| ***1991-present*** |  | **50° 48′ 07.9″ N, 01° 06′ 40.5″ W** |

**SupplementaryTable B2a:** High water levels (m CD) that reached or exceeded a 1 in 5 year return level at this site.

| **Date and time (GMT)** | **Return period (years)** | **Water**  **level (m CD)** | **Astronomical tide (m CD)** | **Skew surge (m)** |
| --- | --- | --- | --- | --- |
| 06/12/2013 01:00 | 12 | 5.56 | 4.87 | 0.69 |
| 14/02/2014 23:00 | 9 | 5.54 | 4.28 | 1.20 |
| 23/12/1995 11:45 | 9 | 5.51 | 5.08 | 0.43 |
| 11/01/1993 13:15 | 8 | 5.48 | 4.82 | 0.66 |
| 07/12/1994 02:00 | 8 | 5.48 | 4.78 | 0.68 |
| 10/01/1993 12:30 | 8 | 5.48 | 4.82 | 0.66 |
| 10/03/2008 13:00 | 7 | 5.50 | 4.91 | 0.58 |
| 03/01/2014 12:30 | 7 | 5.49 | 4.91 | 0.58 |

**SupplementaryTable B2b:** Skew surges (m) that reached or exceeded a 1 in 5 year return level at this site.

| **Date and time (GMT)** | **Return period (years)** | **Skew surge (m)** | **Water**  **level (m CD)** | **Astronomical tide (m CD)** |
| --- | --- | --- | --- | --- |
| 14/02/2014 23:00 | 349 | 1.20 | 5.54 | 4.28 |
| 23/12/2013 14:45 | 7 | 0.80 | 4.88 | 4.08 |
| 21/02/1993 11:30 | 6 | 0.78 | 5.15 | 4.36 |
| 28/10/2013 05:45 | 5 | 0.77 | 4.70 | 3.93 |

| **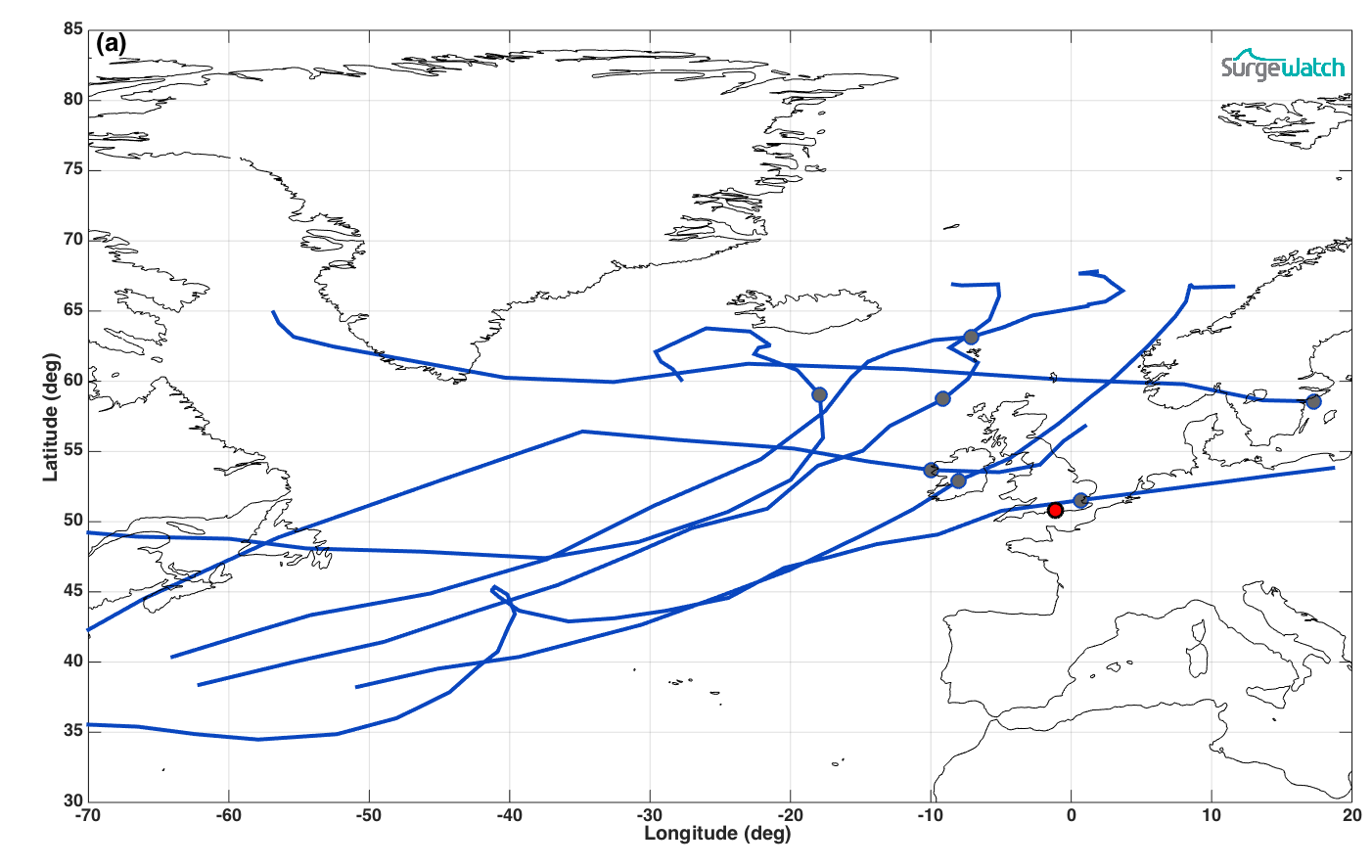**  **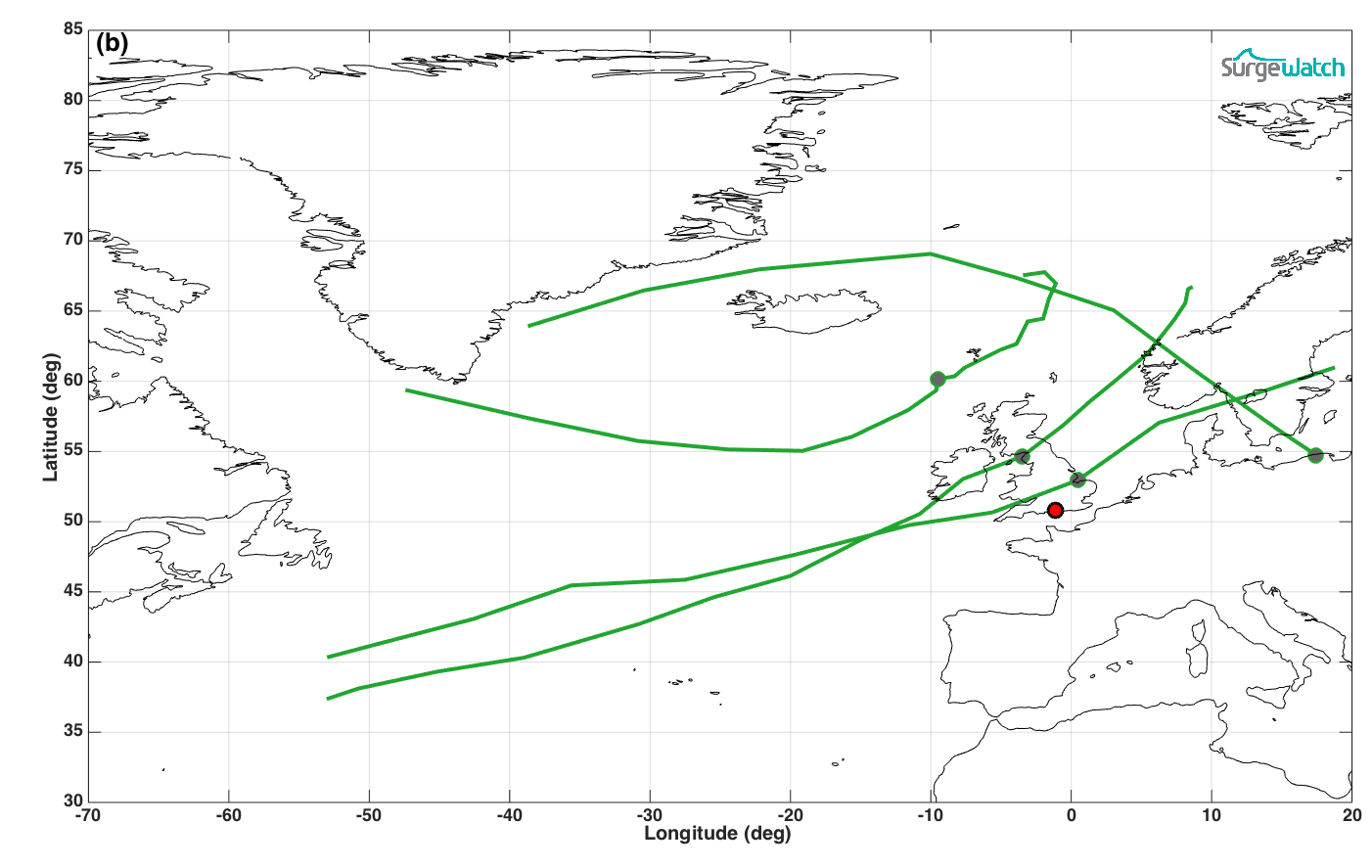** |
| --- |
| **Supplementary Figure B2:** Tracks of the storms that generated (a) high water levels and (b) skew surges that reached or exceeded the 1 in 5 year return level at Portsmouth (location shown with the blue dot). The red dot indicates the location of the storm centre at the time of highest water level or skew surge. |


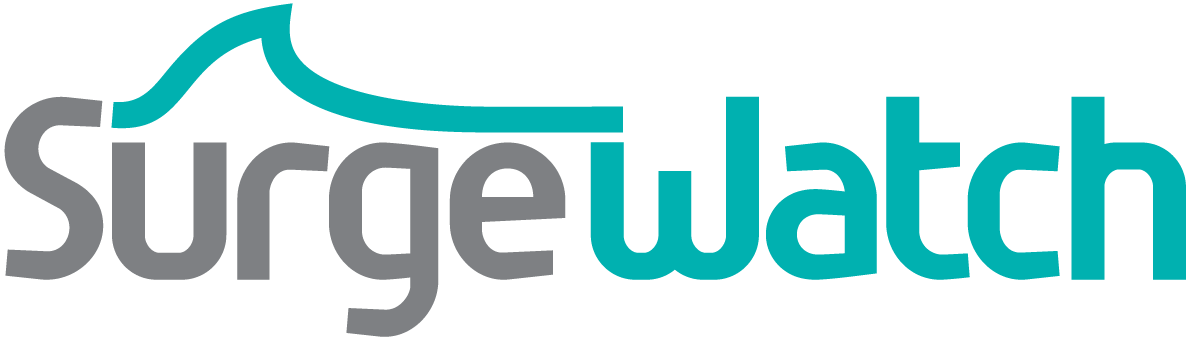
**LOCATION**

** B3.** **Bournemouth**

*Chart Datum (CD) is 1.4m below Ordnance Datum Newlyn (ODN).*

| *Observational Period* |  | *Latitude & Longitude* |
| --- | --- | --- |
| ***1996-present*** |  | ***50° 42′ 51.6″N, 01° 52′ 29.5″W*** |

**Supplementary Table B3a:** High water levels (m CD) that reached or exceeded a 1 in 5 year return level at this site.

| **Date and time (GMT)** | **Return period (years)** | **Water**  **level (m CD)** | **Astronomical tide (m CD)** | **Skew surge (m)** |
| --- | --- | --- | --- | --- |
| 10/03/2008 10:30 | 23 | 3.09 | 2.43 | 0.66 |

**Supplementary Table B3b:** Skew surges (m) that reached or exceeded a 1 in 5 year return level at this site.

| **Date and time (GMT)** | **Return period (years)** | **Skew surge (m)** | **Water**  **level (m CD)** | **Astronomical tide (m CD)** |
| --- | --- | --- | --- | --- |
| 02/01/2001 05:00 | 21 | 0.76 | 2.73 | 1.96 |
| 19/11/1996 07:00 | 9 | 0.70 | 2.79 | 2.08 |
| 19/01/2009 06:30 | 6 | 0.67 | 2.69 | 2.00 |
| 10/03/2008 10:30 | 6 | 0.66 | 3.09 | 2.43 |

| **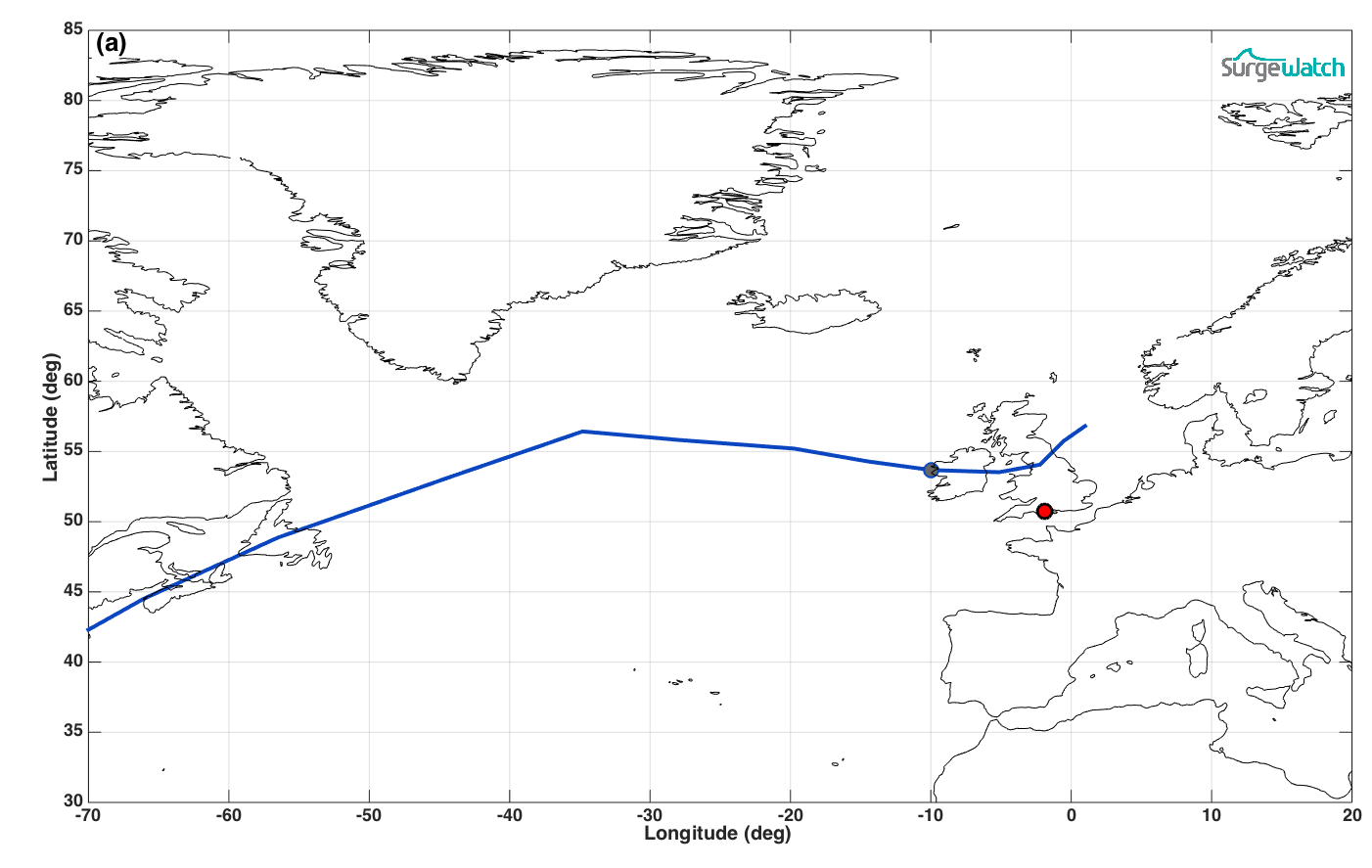** |
| --- |
| **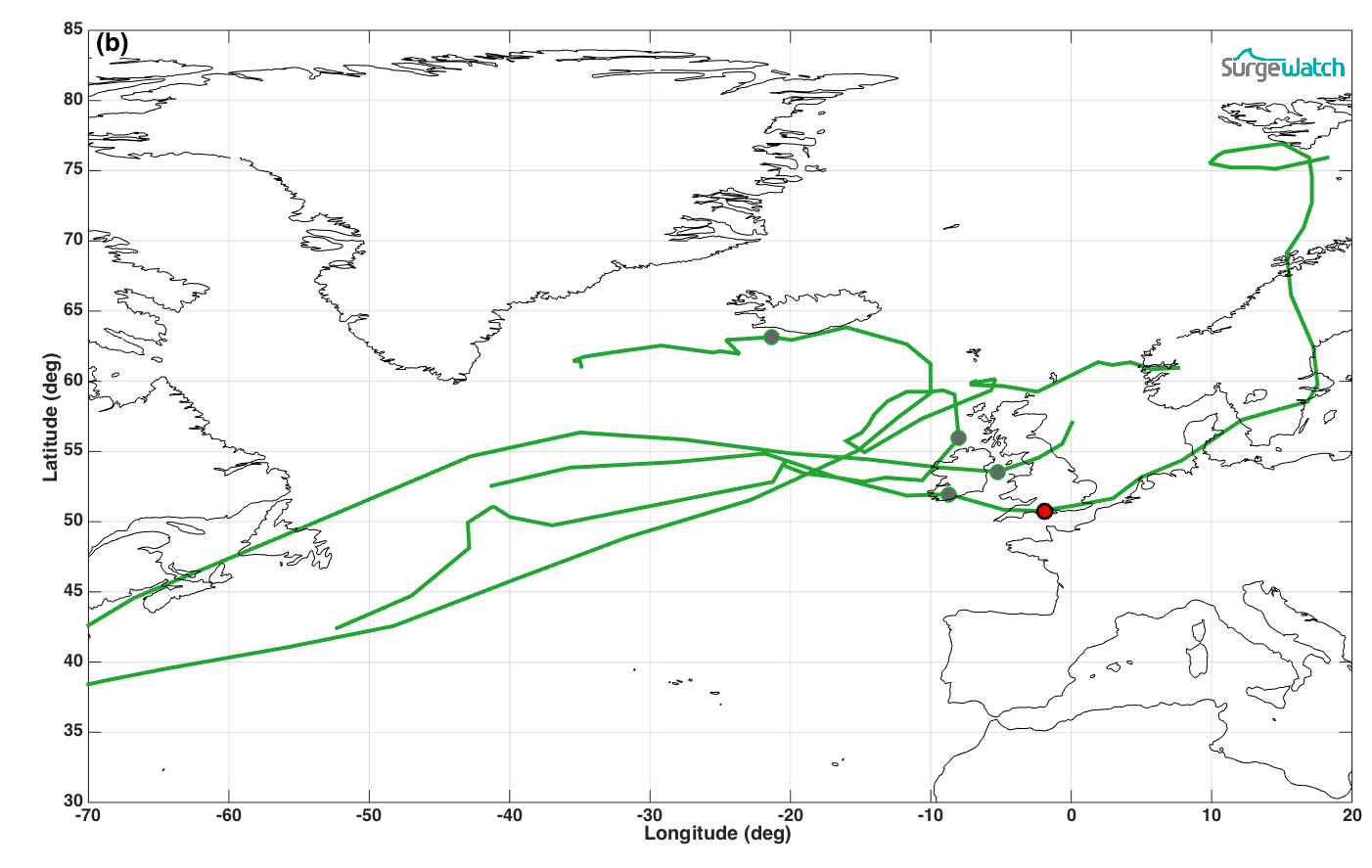** |
| **Supplementary Figure B3:** Tracks of the storms that generated (a) high water levels and (b) skew surges that reached or exceeded the 1 in 5 year return level at Bournemouth (location shown with the blue dot). The red dot indicates the location of the storm centre at the time of highest water level or skew surge. |


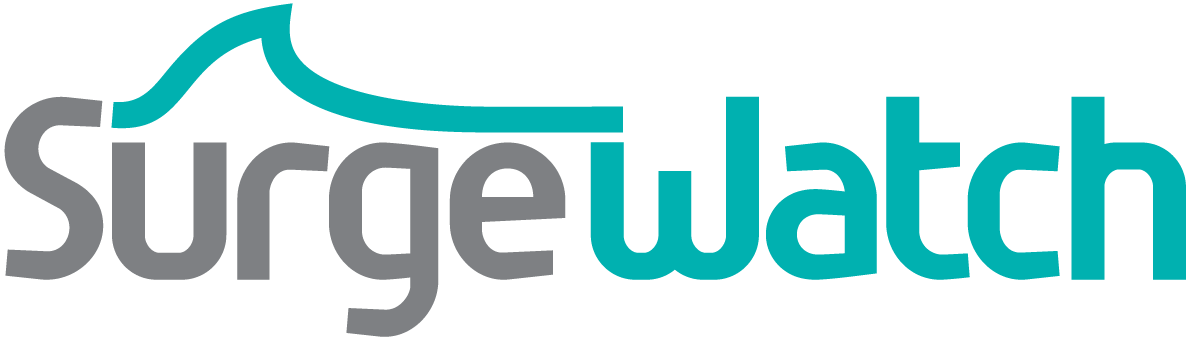
**LOCATION**

** B4.** **Weymouth**

*Chart Datum (CD) is 0.93m below Ordnance Datum Newlyn (ODN).*

| *Observational Period* |  | *Latitude & Longitude* |
| --- | --- | --- |
| ***1991-present*** |  | ***50° 36′ 30.6″ N, 02° 26′ 52.6″ W*** |

**Supplementary Table B4a:** High water levels (m CD) that reached or exceeded a 1 in 5 year return level at this site.

| **Date and time (GMT)** | **Return period (years)** | **Water**  **level (m CD)** | **Astronomical tide (m CD)** | **Skew surge (m)** |
| --- | --- | --- | --- | --- |
| 10/03/2008 09:00 | 40 | 3.04 | 2.48 | 0.53 |
| 27/10/2004 18:00 | 17 | 2.95 | 2.35 | 0.60 |
| 23/12/1995 07:30 | 14 | 2.93 | 2.69 | 0.24 |
| 03/01/2014 08:00 | 13 | 2.94 | 2.53 | 0.41 |
| 14/02/2014 19:45 | 11 | 2.93 | 1.90 | 0.94 |
| 03/12/2006 05:45 | 9 | 2.90 | 2.17 | 0.68 |
| 10/01/1993 08:00 | 8 | 2.87 | 2.48 | 0.38 |
| 12/12/2000 19:15 | 7 | 2.88 | 2.48 | 0.39 |
| 10/03/2001 07:15 | 7 | 2.87 | 2.65 | 0.23 |
| 10/02/1997 09:15 | 7 | 2.86 | 2.53 | 0.32 |
| 30/08/1992 08:00 | 6 | 2.85 | 2.46 | 0.39 |
| 24/10/1995 19:45 | 5 | 2.84 | 2.34 | 0.38 |

**Supplementary Table B4b:** Skew surges (m) that reached or exceeded a 1 in 5 year return level at this site.

|  | **Date and time (GMT)** | **Return period (years)** | **Skew surge (m)** | **Water**  **level (m CD)** | **Astronomical tide (m CD)** |  |
| --- | --- | --- | --- | --- | --- | --- |
|  | 14/02/2014 19:45 | 44 | 0.94 | 2.93 | 1.90 |  |
|  | 05/02/2014 10:30 | 15 | 0.82 | 2.84 | 2.02 |  |
|  | 04/02/2014 21:45 | 8 | 0.75 | 2.72 | 1.94 |  |
| **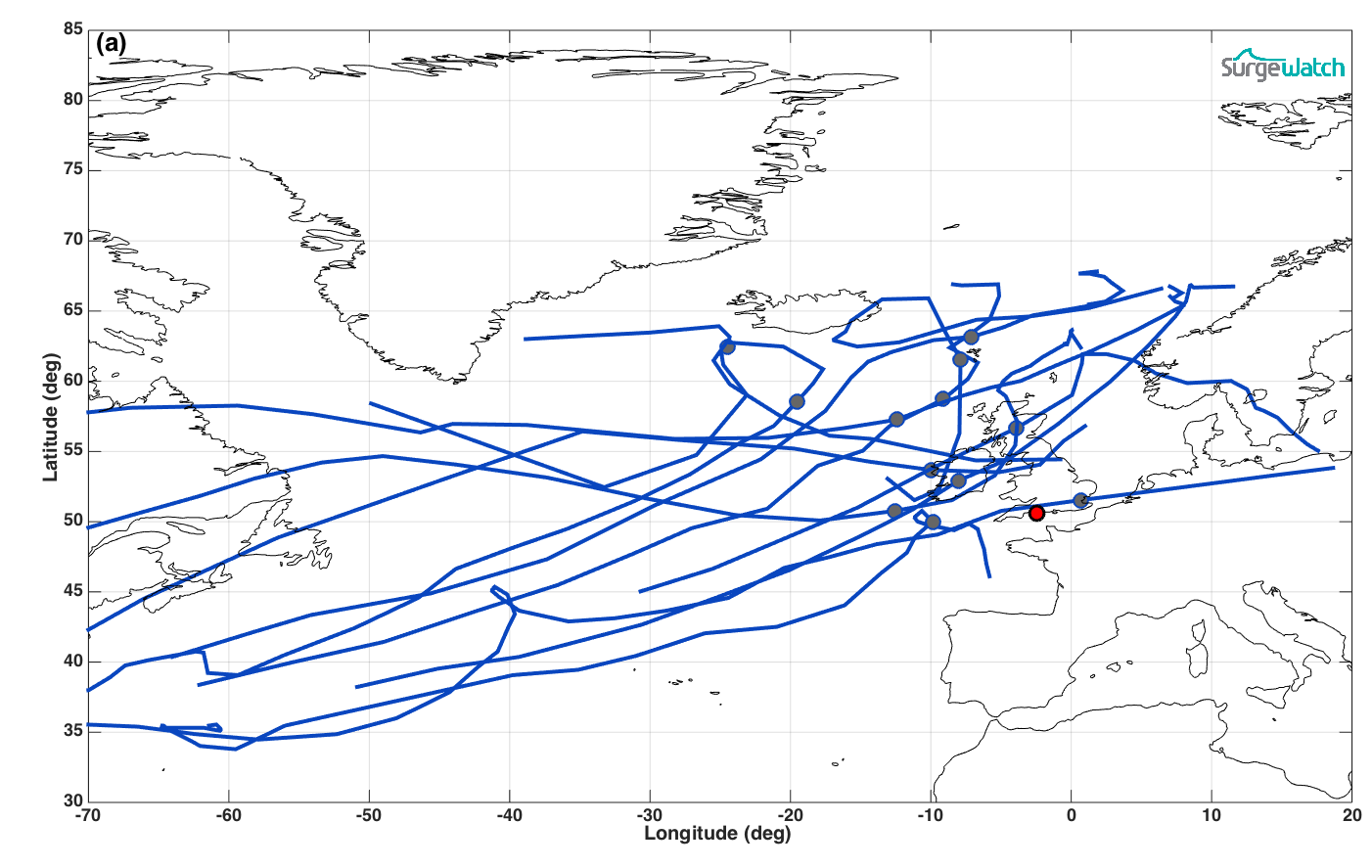**  **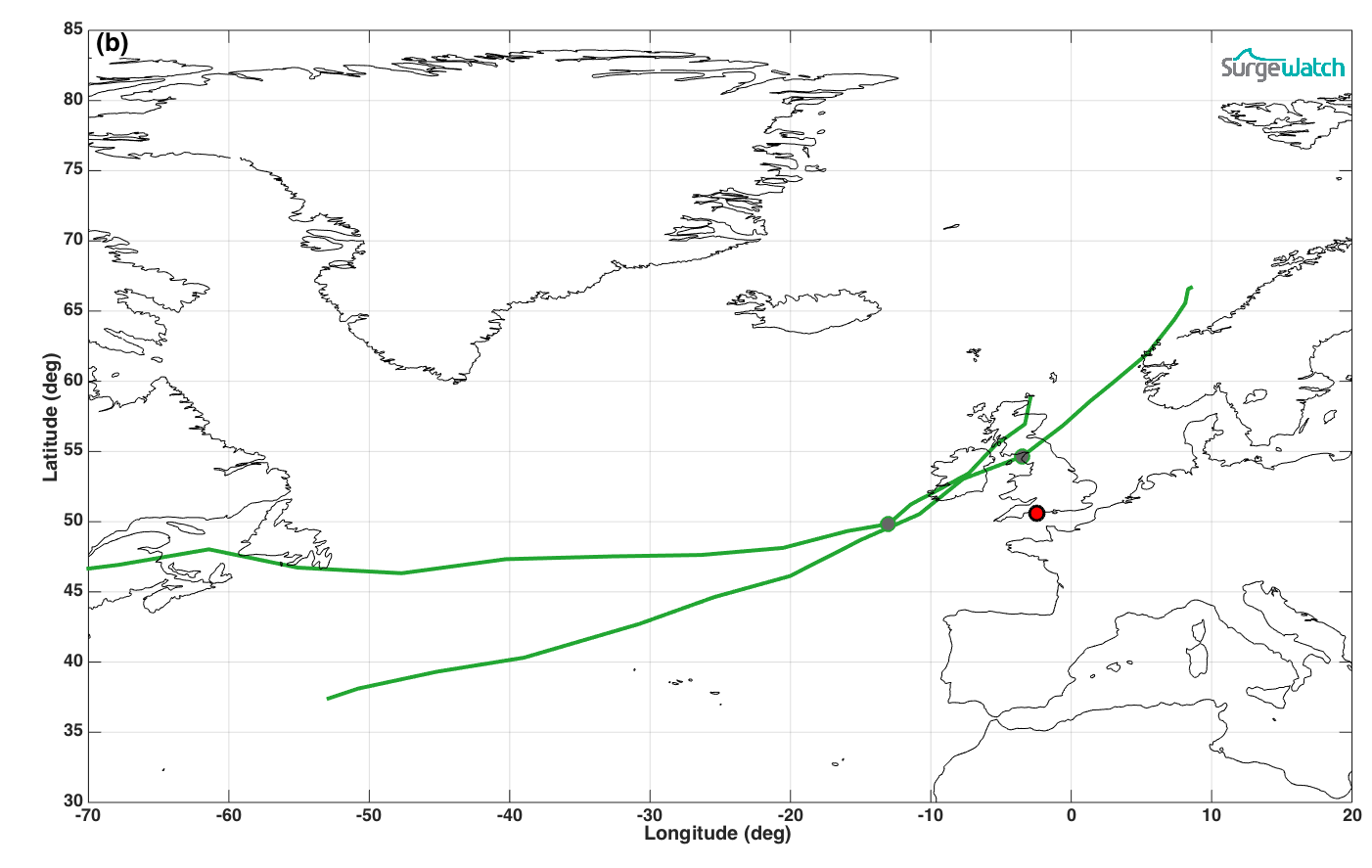** | | | | | | |
| **Supplementary Figure B4:** Tracks of the storms that generated (a) high water levels and (b) skew surges that reached or exceeded the 1 in 5 year return level at Weymouth (location shown with the blue dot). The red dot indicates the location of the storm centre at the time of highest water level or skew surge. | | | | | | |


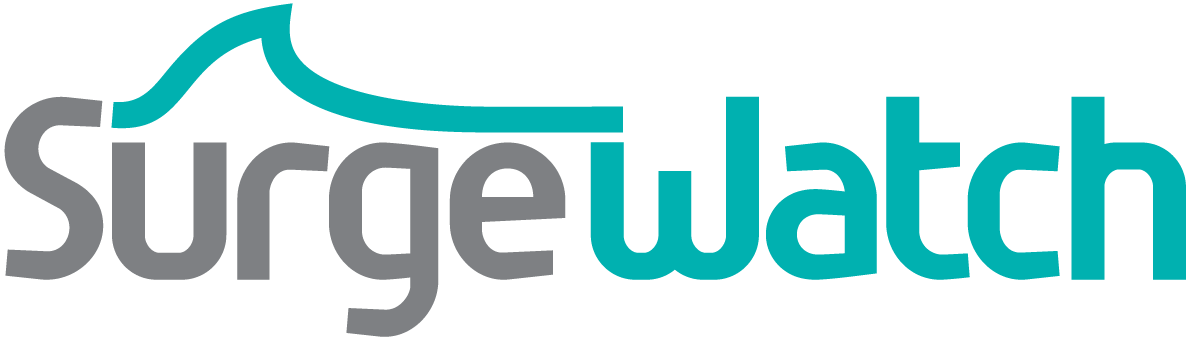
**LOCATION**

** B5.** **Devonport**

*Chart Datum (CD) is 3.22m below Ordnance Datum Newlyn (ODN).*

| *Observational Period* |  | *Latitude & Longitude* |
| --- | --- | --- |
| ***1987,1991-present*** |  | ***50° 22′ 06.2″ N, 04° 11′ 06.9″ W*** |

**Supplementary Table B5a:** High water levels (m CD) that reached or exceeded a 1 in 5 year return level at this site.

| **Date and time (GMT)** | **Return period (years)** | **Water**  **level (m CD)** | **Astronomical tide (m CD)** | **Skew surge (m)** |
| --- | --- | --- | --- | --- |
| 03/02/2014 08:15 | 8 | 6.37 | 5.91 | 0.46 |
| 27/10/2004 17:15 | 8 | 6.35 | 5.68 | 0.67 |
| 10/03/2008 07:30 | 7 | 6.34 | 5.73 | 0.61 |
| 07/10/1987 18:00 | 7 | 6.31 | 5.93 | 0.38 |

**Supplementary Table B5b:** Skew surges (m) that reached or exceeded a 1 in 5 year return level at this site.

| **Date and time (GMT)** | **Return period (years)** | **Skew surge (m)** | **Water**  **level (m CD)** | **Astronomical tide (m CD)** |
| --- | --- | --- | --- | --- |
| 14/02/2014 17:45 | 22 | 0.92 | 6.22 | 5.30 |
| 04/02/2014 21:15 | 15 | 0.87 | 6.15 | 5.28 |
| 16/10/1987 00:00 | 14 | 0.85 | 4.92 | 4.04 |
| 23/12/2013 21:15 | 9 | 0.81 | 5.64 | 4.83 |

| **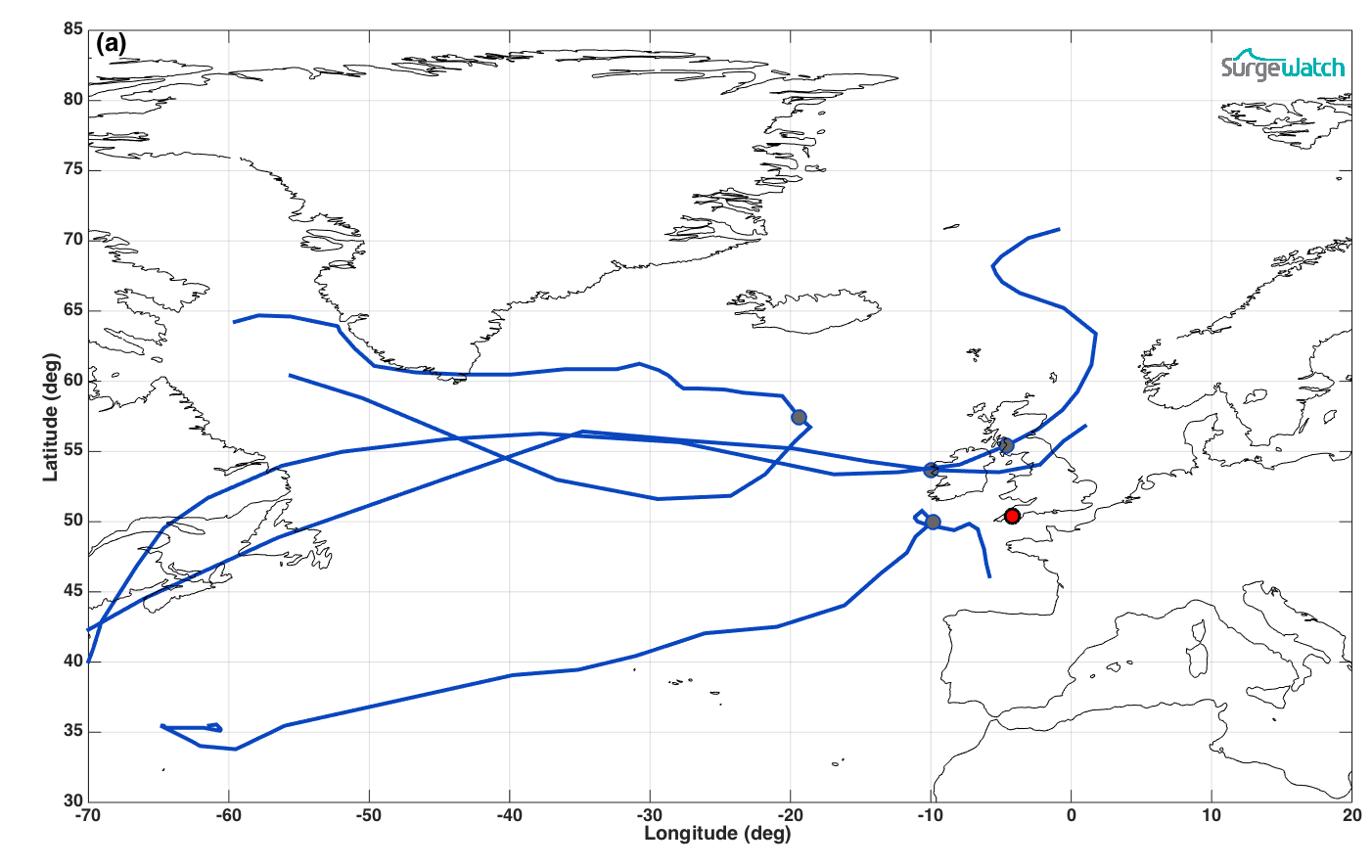** |
| --- |
| **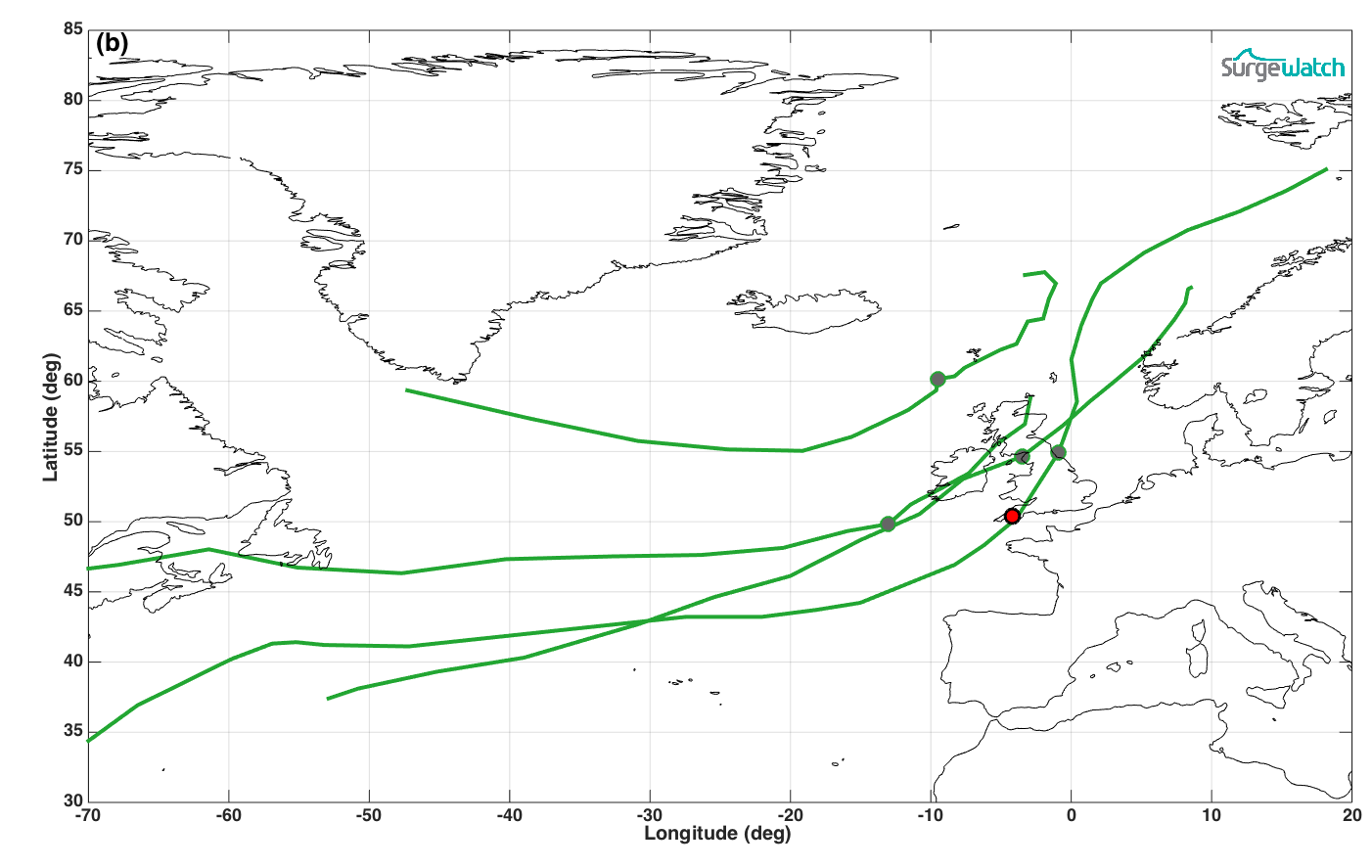** |
| **Supplementary Figure B5:** Tracks of the storms that generated (a) high water levels and (b) skew surges that reached or exceeded the 1 in 5 year return level at Devonport (location shown with the blue dot). The red dot indicates the location of the storm centre at the time of highest water level or skew surge. |


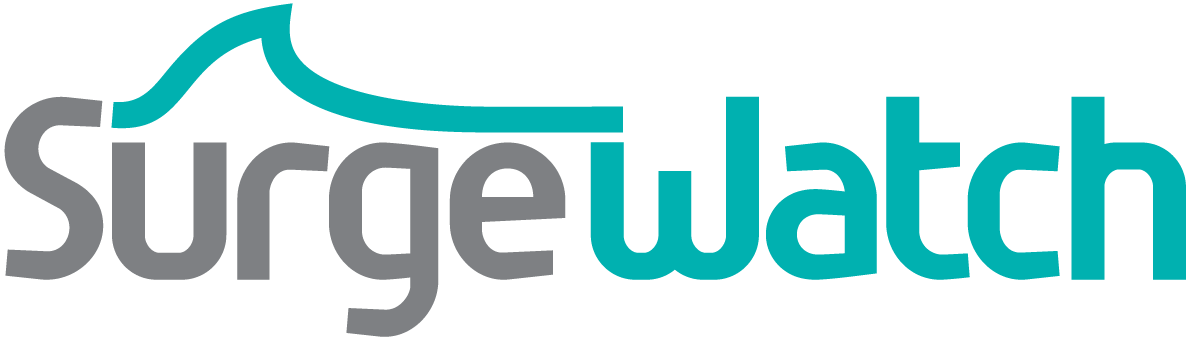
**LOCATION**

** B6.** **Newlyn**

*Chart Datum (CD) is 3.05m below Ordnance Datum Newlyn (ODN).*

| *Observational Period* |  | *Latitude & Longitude* |
| --- | --- | --- |
| ***1915-present*** |  | ***50° 06′ 10.8″ N, 05° 32′ 33.9″ W*** |

**Supplementary Table B6a:** High water levels (m CD) that reached or exceeded a 1 in 5 year return level at this site.

| **Date and time (GMT)** | **Return period (years)** | **Water**  **level (m CD)** | **Astronomical tide (m CD)** | **Skew surge (m)** |
| --- | --- | --- | --- | --- |
| 29/01/1948 07:00 | 39 | 6.32 | 5.87 | 0.45 |
| 27/10/2004 16:15 | 37 | 6.42 | 5.64 | 0.78 |
| 03/02/2014 07:00 | 37 | 6.44 | 5.97 | 0.47 |
| 07/04/1985 06:00 | 28 | 6.36 | 5.97 | 0.39 |
| 07/04/1985 18:00 | 16 | 6.32 | 5.85 | 0.47 |
| 10/03/2008 06:15 | 15 | 6.35 | 5.89 | 0.46 |
| 23/03/1928 06:00 | 14 | 6.20 | 5.82 | 0.38 |
| 24/03/1936 05:00 | 10 | 6.19 | 6.07 | 0.12 |
| 25/03/1936 06:00 | 9 | 6.18 | 6.01 | 0.17 |
| 06/10/1979 17:00 | 9 | 6.26 | 5.99 | 0.27 |
| 23/12/1995 05:00 | 9 | 6.29 | 6.07 | 0.22 |
| 17/10/2012 05:15 | 9 | 6.32 | 6.06 | 0.26 |
| 03/01/2014 06:00 | 9 | 6.32 | 5.96 | 0.32 |
| 14/02/1941 06:00 | 9 | 6.18 | 5.88 | 0.31 |
| 02/03/2014 05:00 | 8 | 6.31 | 6.11 | 0.19 |
| 06/03/1931 06:00 | 8 | 6.16 | 5.91 | 0.24 |
| 16/02/1941 08:00 | 7 | 6.17 | 5.58 | 0.56 |
| 28/01/1937 06:00 | 7 | 6.16 | 5.63 | 0.53 |
| 30/03/2006 05:00 | 6 | 6.27 | 5.99 | 0.28 |
| 13/10/1939 17:00 | 6 | 6.14 | 5.87 | 0.28 |
| 24/12/1995 06:00 | 6 | 6.25 | 6.11 | 0.14 |
| 22/01/1996 05:45 | 6 | 6.25 | 6.03 | 0.22 |
| 08/10/2002 18:00 | 6 | 6.26 | 6.10 | 0.14 |
| 08/10/2006 17:30 | 5 | 6.26 | 6.15 | 0.09 |
| 16/12/1989 07:00 | 5 | 6.23 | 5.66 | 0.57 |

**Supplementary Table B6b:** Skew surges (m) that reached or exceeded a 1 in 5 year return level at this site.

| **Date and time (GMT)** | **Return period (years)** | **Skew surge (m)** | **Water**  **level (m CD)** | **Astronomical tide (m CD)** |
| --- | --- | --- | --- | --- |
| 01/02/1926 07:00 | 69 | 0.82 | 5.97 | 5.15 |
| 14/02/2014 16:45 | 65 | 0.82 | 6.08 | 5.26 |
| 26/11/1954 17:00 | 54 | 0.80 | 5.86 | 5.05 |
| 10/11/1963 13:00 | 46 | 0.79 | 5.21 | 4.40 |
| 29/11/1954 19:00 | 43 | 0.78 | 5.61 | 4.83 |
| 27/10/2004 16:15 | 39 | 0.78 | 6.42 | 5.64 |
| 05/01/1936 14:00 | 20 | 0.73 | 5.50 | 4.77 |
| 16/12/1989 19:00 | 18 | 0.72 | 5.98 | 5.26 |
| 18/12/1983 15:00 | 16 | 0.71 | 5.87 | 5.16 |
| 23/12/2013 20:15 | 10 | 0.67 | 5.47 | 4.80 |
| 02/11/1918 03:00 | 9 | 0.67 | 5.61 | 4.92 |
| 10/12/1957 19:00 | 8 | 0.66 | 5.84 | 5.18 |
| 30/12/1981 07:00 | 8 | 0.66 | 5.83 | 5.17 |
| 19/12/1958 11:00 | 7 | 0.64 | 5.14 | 4.50 |
| 23/03/1955 04:00 | 7 | 0.64 | 5.93 | 5.29 |
| 18/01/1939 03:00 | 6 | 0.64 | 5.49 | 4.85 |
| 01/11/1960 15:00 | 6 | 0.63 | 5.88 | 5.24 |
| 05/11/1916 01:00 | 5 | 0.63 | 5.15 | 4.52 |

| **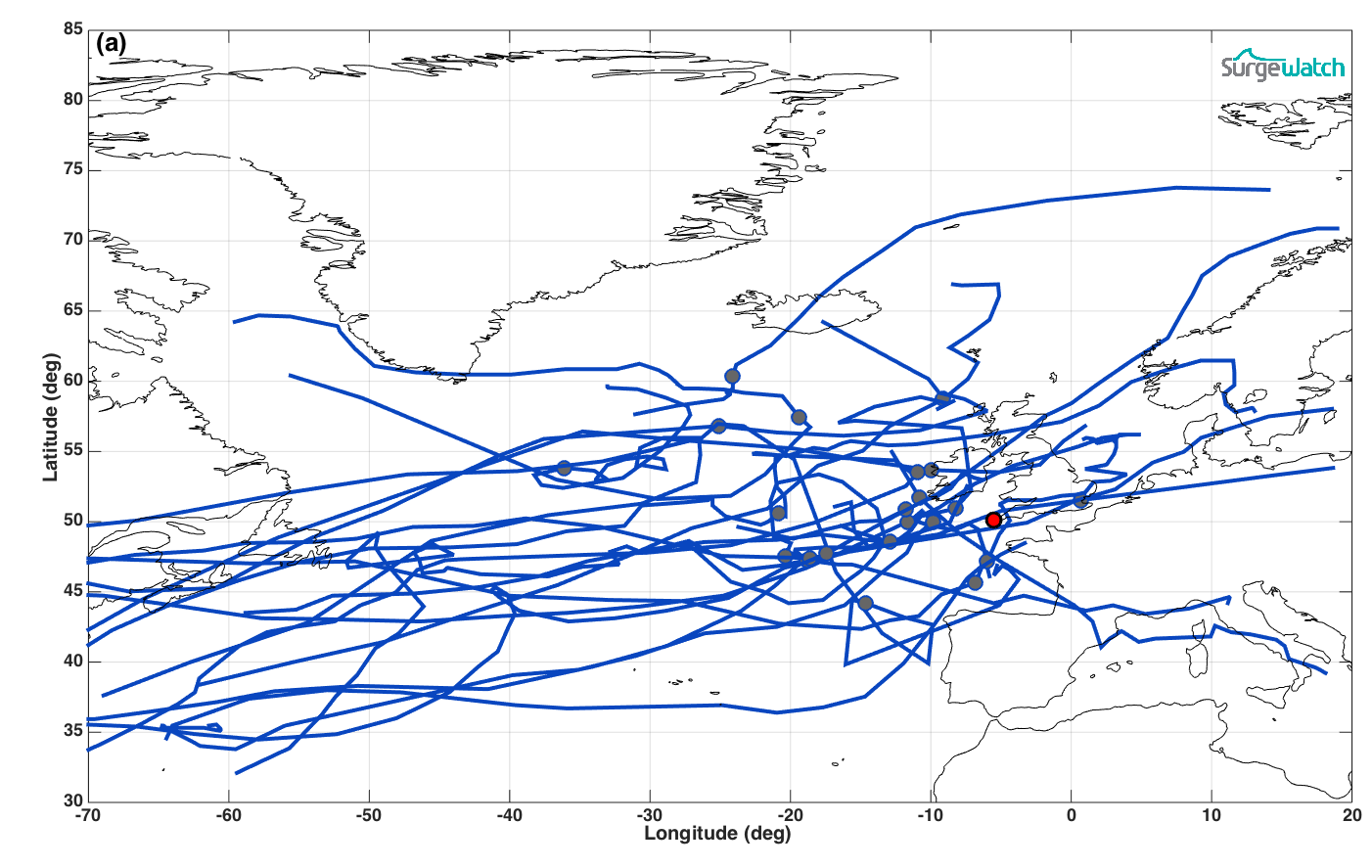** |
| --- |
| **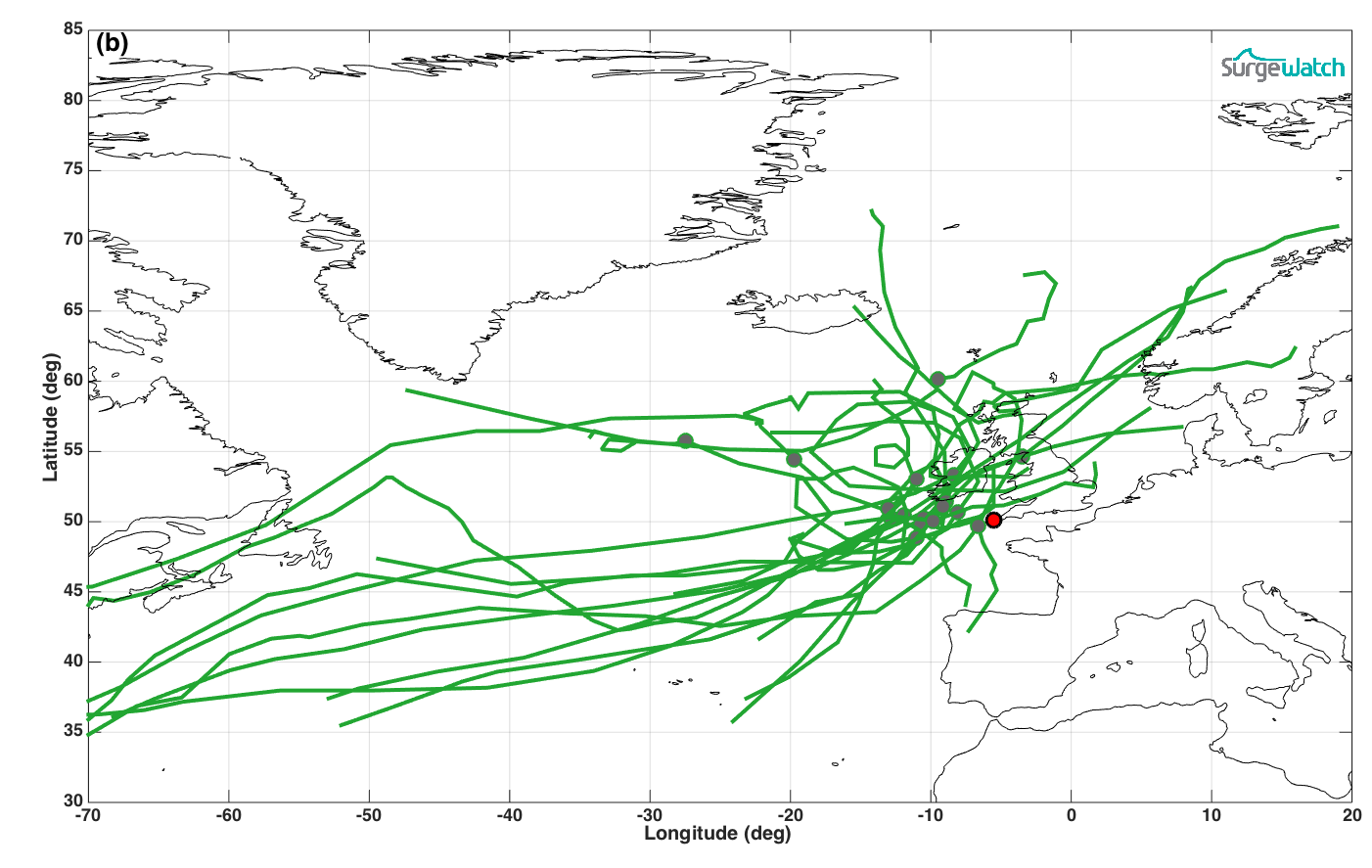** |
| **Supplementary Figure B6:** Tracks of the storms that generated (a) high water levels and (b) skew surges that reached or exceeded the 1 in 5 year return level at Newlyn (location shown with the blue dot). The red dot indicates the location of the storm centre at the time of highest water level or skew surge. |


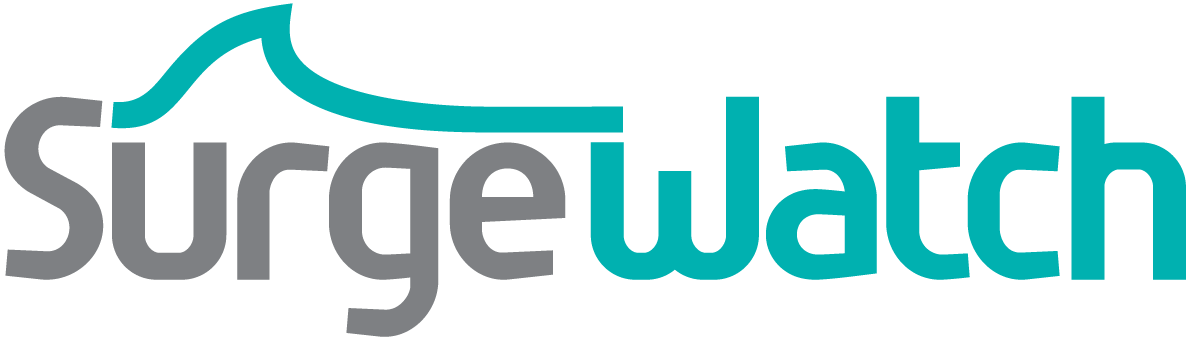
**LOCATION**

** B7.** **St. Mary’s**

*Chart Datum (CD) is 2.9m below Ordnance Datum Newlyn (ODN).*

| *Observational Period* |  | *Latitude & Longitude* |
| --- | --- | --- |
| ***1952,1958,1965-1975,1980-present*** |  | ***51° 26′ 44.3″ N, 00° 44′ 36.1″ E*** |

**Supplementary Table B7a:** High water levels (m CD) that reached or exceeded a 1 in 5 year return level at this site.

| **Date and time (GMT)** | **Return period (years)** | **Water**  **level (m CD)** | **Astronomical tide (m CD)** | **Skew surge (m)** |
| --- | --- | --- | --- | --- |
| 30/03/2006 05:15 | 6 | 6.45 | 6.17 | 0.28 |
| 10/03/2001 05:00 | 5 | 6.44 | 6.20 | 0.23 |
| 11/03/2001 05:45 | 5 | 6.44 | 6.26 | 0.17 |

**Supplementary Table B7b:** Skew surges (m) that reached or exceeded a 1 in 5 year return level at this site.

| **Date and time (GMT)** | **Return period (years)** | **Skew surge (m)** | **Water**  **level (m CD)** | **Astronomical tide (m CD)** |
| --- | --- | --- | --- | --- |
| 04/02/2014 20:00 | 71 | 0.73 | 6.03 | 5.29 |
| 27/10/2004 16:15 | 29 | 0.68 | 6.40 | 5.72 |
| 07/02/2014 22:30 | 9 | 0.61 | 4.90 | 4.28 |
| 06/01/1996 17:30 | 5 | 0.58 | 5.92 | 5.32 |

| **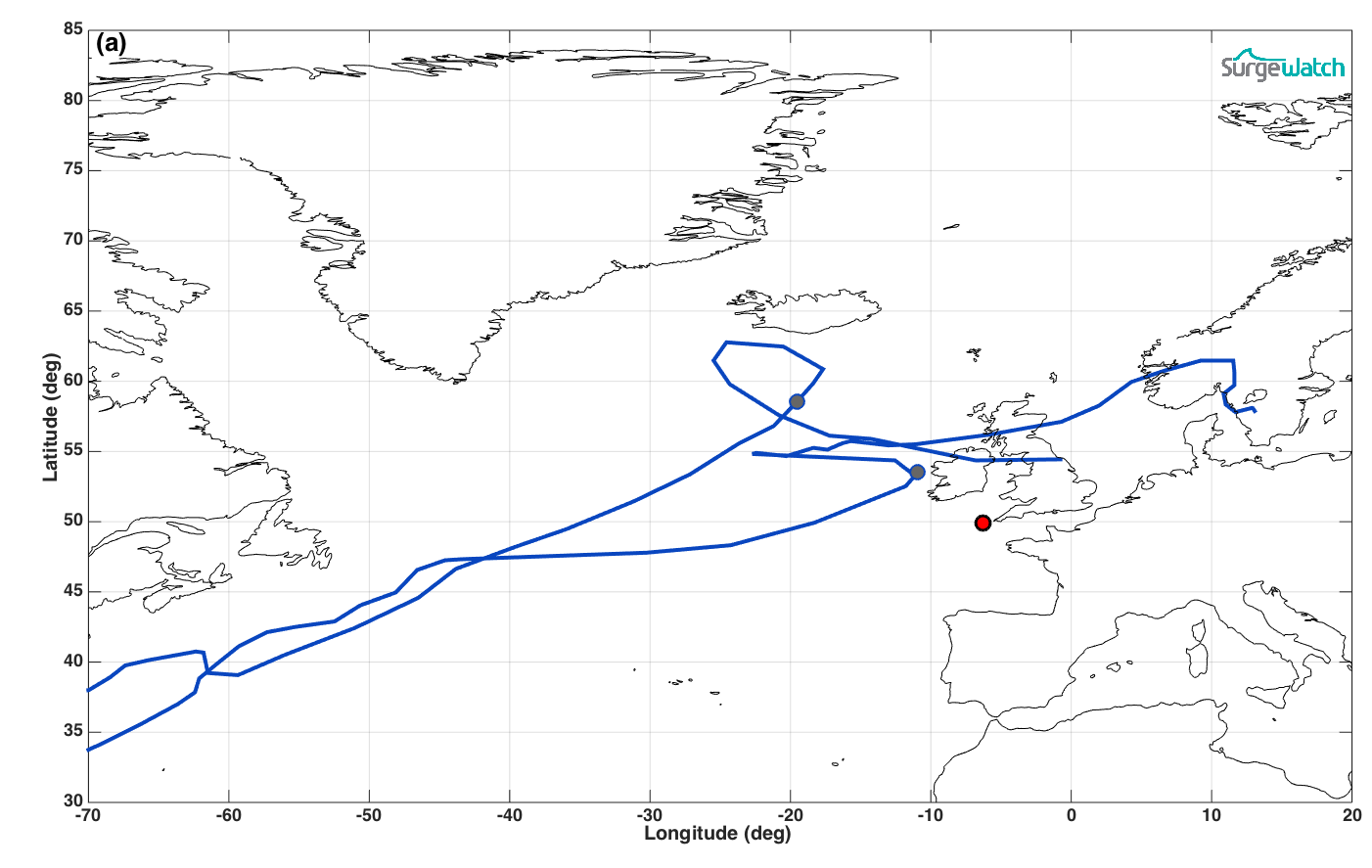**  **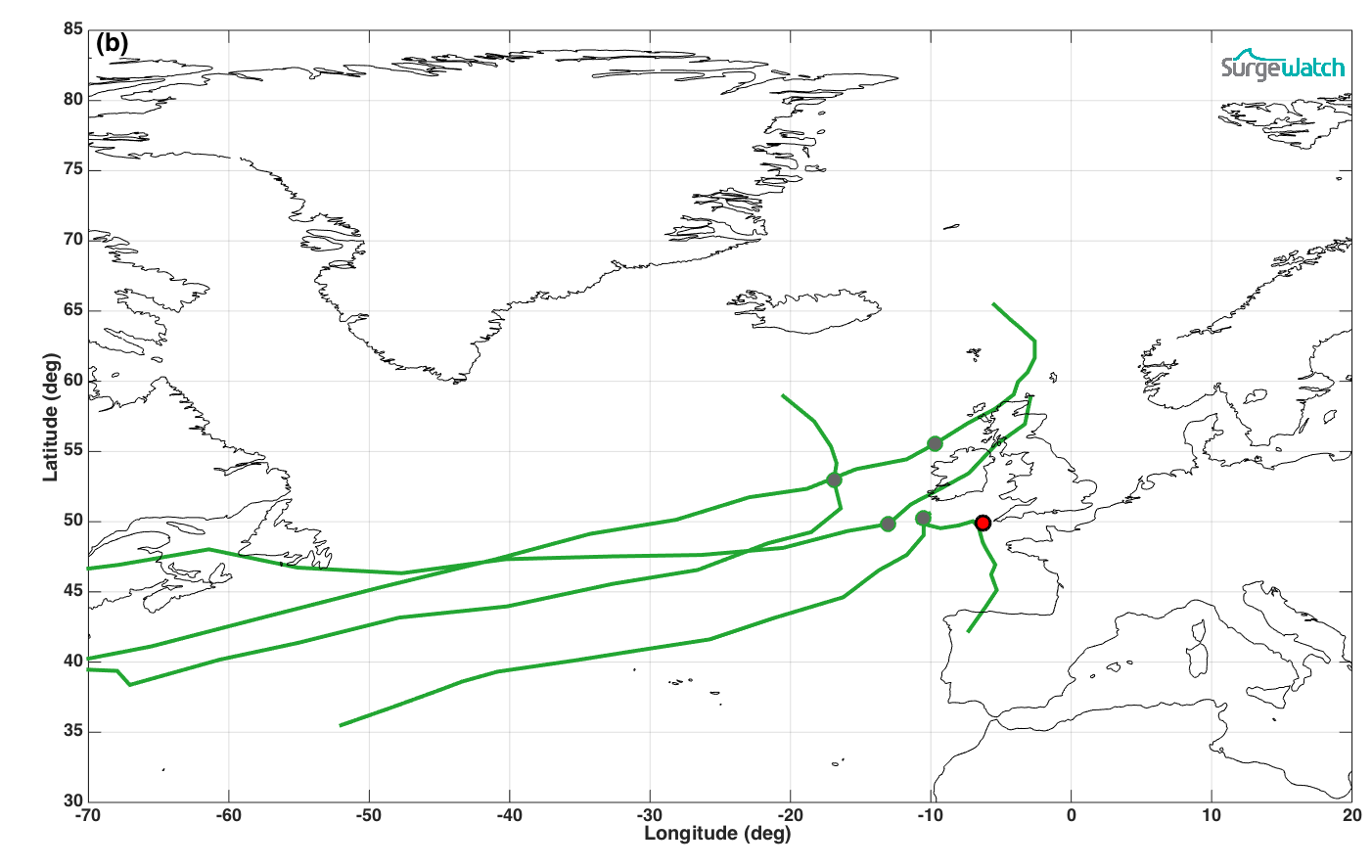** |
| --- |
| **Supplementary Figure B7:** Tracks of the storms that generated (a) high water levels and (b) skew surges that reached or exceeded the 1 in 5 year return level at St. Mary’s (location shown with the blue dot). The red dot indicates the location of the storm centre at the time of highest water level or skew surge. |


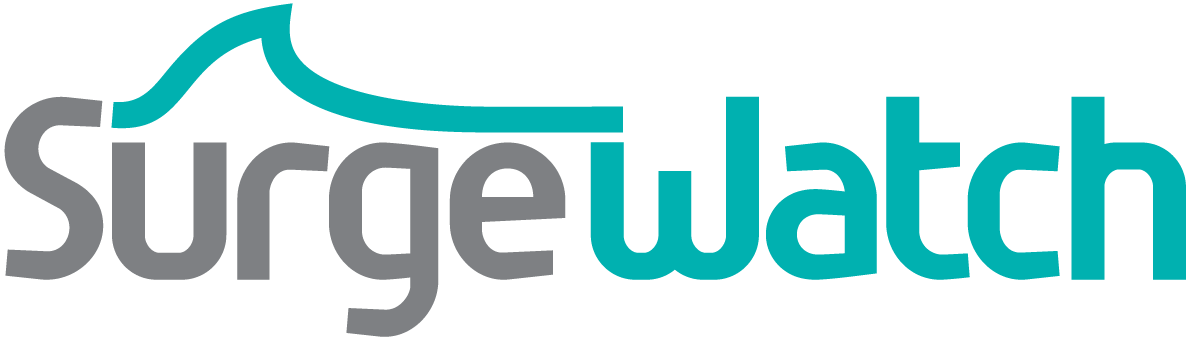
**LOCATION**

** B8.** **Ilfracombe**

*Chart Datum (CD) is 4.80m below Ordnance Datum Newlyn (ODN).*

| *Observational Period* |  | *Latitude & Longitude* |
| --- | --- | --- |
| ***1968-present*** |  | ***51° 12′ 40.1″ N, 04° 06′ 44.6″ W*** |

**Supplementary Table B8a:** High water levels (m CD) that reached or exceeded a 1 in 5 year return level at this site.

| **Date and time (GMT)** | **Return period (years)** | **Water**  **level (m CD)** | **Astronomical tide (m CD)** | **Skew surge (m)** |
| --- | --- | --- | --- | --- |
| 07/04/1985 07:00 | 23 | 10.46 | 10.07 | 0.39 |
| 03/01/2014 07:00 | 20 | 10.50 | 10.02 | 0.48 |
| 10/02/1997 08:00 | 14 | 10.42 | 10.07 | 0.35 |
| 09/03/1989 07:00 | 10 | 10.38 | 10.24 | 0.14 |
| 02/03/2014 06:30 | 9 | 10.42 | 10.21 | 0.21 |
| 03/02/2014 08:15 | 8 | 10.40 | 9.94 | 0.46 |
| 08/10/2006 18:45 | 7 | 10.38 | 10.31 | 0.06 |
| 30/03/2006 06:30 | 6 | 10.36 | 10.16 | 0.21 |
| 26/09/1984 19:00 | 6 | 10.32 | 10.29 | 0.03 |
| 01/02/2014 06:45 | 5 | 10.37 | 10.19 | 0.17 |
| 17/10/2012 06:45 | 5 | 10.36 | 10.06 | 0.30 |

**Supplementary Table B8b:** Skew surges (m) that reached or exceeded a 1 in 5 year return level at this site.

| **Date and time (GMT)** | **Return period (years)** | **Skew surge (m)** | **Water**  **level (m CD)** | **Astronomical tide (m CD)** |
| --- | --- | --- | --- | --- |
| 17/01/1969 17:00 | 38 | 0.95 | 9.75 | 8.80 |
| 24/03/1986 05:00 | 33 | 0.93 | 9.30 | 8.37 |
| 27/12/2013 00:15 | 13 | 0.84 | 8.09 | 7.25 |
| 16/12/1989 21:00 | 13 | 0.83 | 9.44 | 8.61 |
| 08/01/2005 03:30 | 11 | 0.82 | 8.74 | 7.92 |
| 14/02/2014 18:00 | 9 | 0.80 | 9.62 | 8.82 |
| 11/02/1995 15:45 | 6 | 0.76 | 7.86 | 7.04 |

| **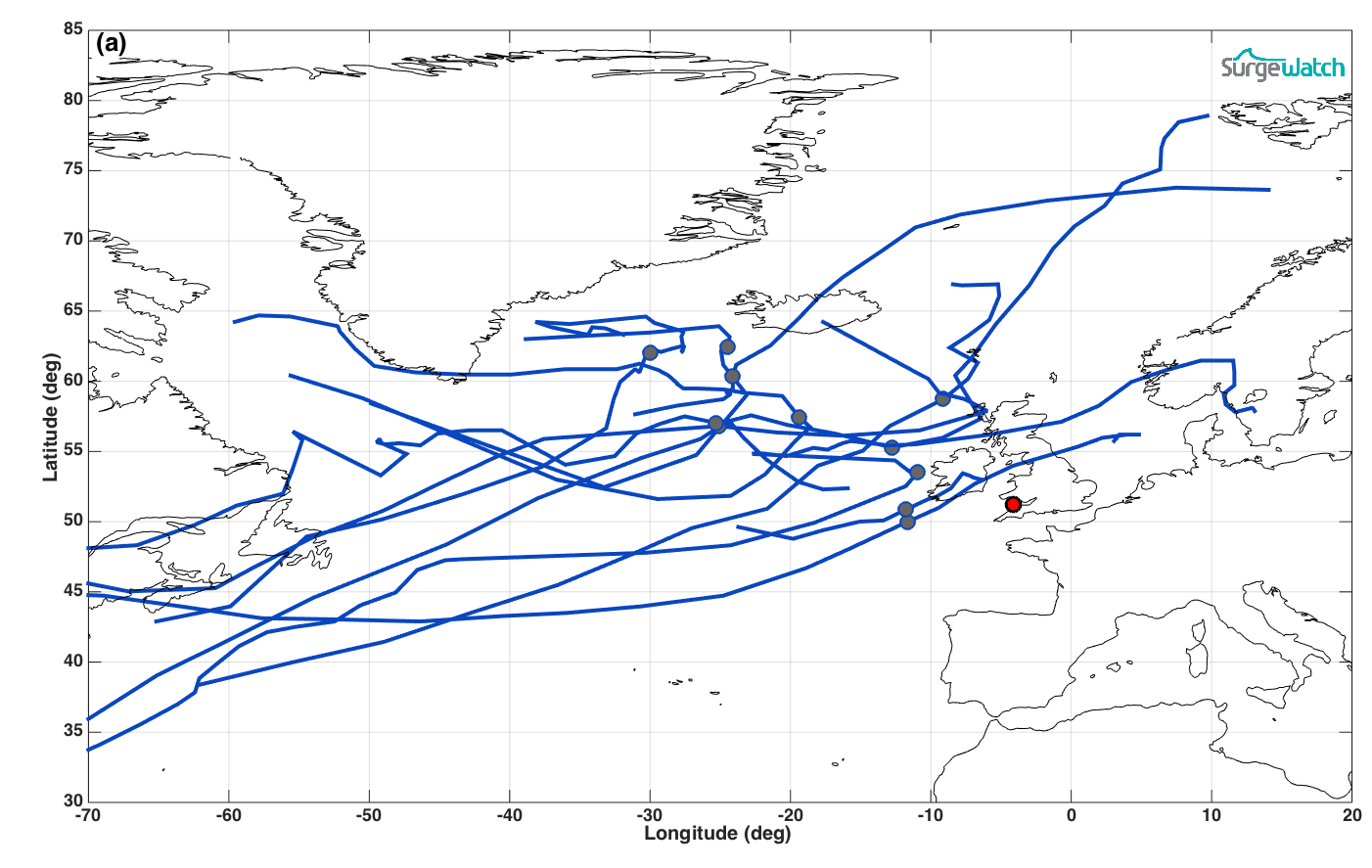**  **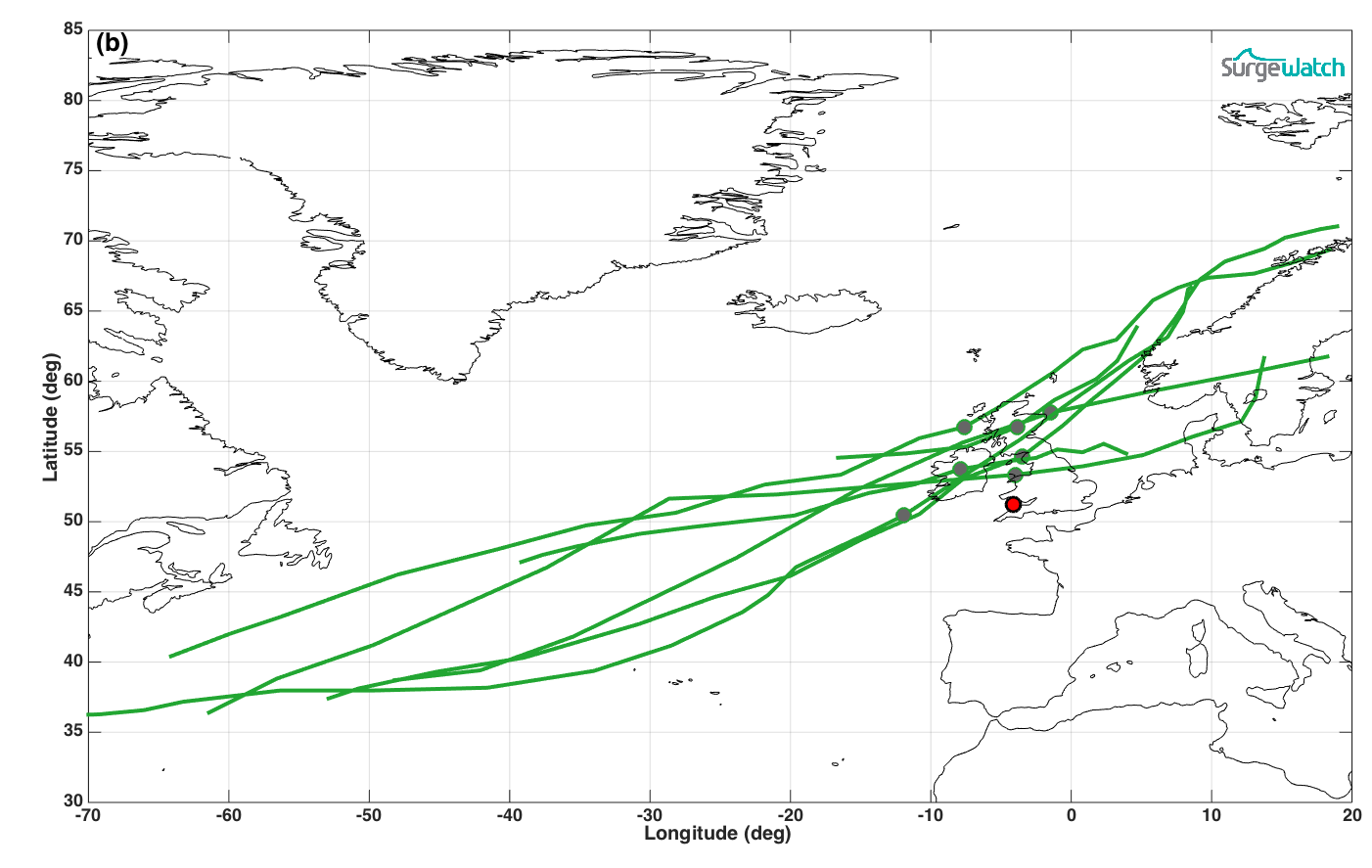** |
| --- |
| **Supplementary Figure B8:** Tracks of the storms that generated (a) high water levels and (b) skew surges that reached or exceeded the 1 in 5 year return level at Ilfracombe (location shown with the blue dot). The red dot indicates the location of the storm centre at the time of highest water level or skew surge. |


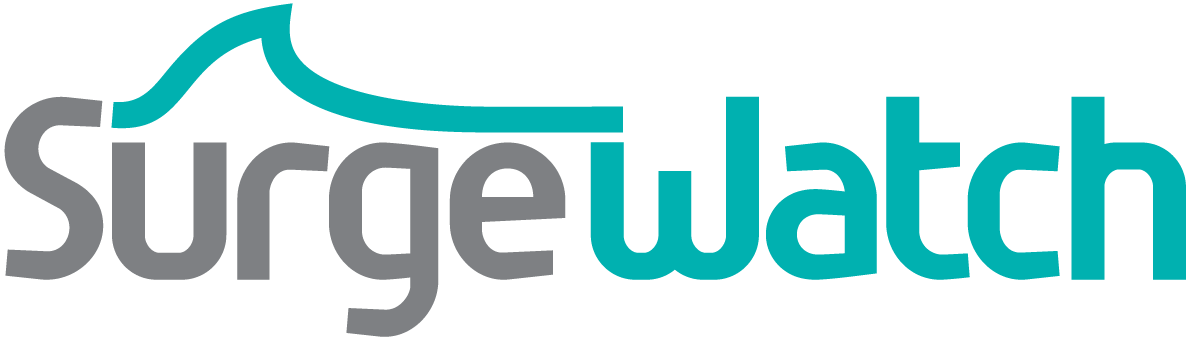
**LOCATION**

** A9.** **Hinkley Point**

*Chart Datum (CD) is 5.90m below Ordnance Datum Newlyn (ODN).*

| *Observational Period* |  | *Latitude & Longitude* |
| --- | --- | --- |
| ***1990-present*** |  | ***51° 12′ 54.9″ N, 03° 08′ 04.1″ W*** |

**Supplementary Table B9a:** High water levels (m CD) that reached or exceeded a 1 in 5 year return level at this site.

| **Date and time (GMT)** | **Return period (years)** | **Water**  **level (m CD)** | **Astronomical tide (m CD)** | **Skew surge (m)** |
| --- | --- | --- | --- | --- |
| 03/01/2014 08:00 | 13 | 13.35 | 12.71 | 0.65 |
| 10/02/1997 08:45 | 8 | 13.26 | 12.74 | 0.52 |
| 02/03/2014 07:15 | 6 | 13.25 | 12.99 | 0.26 |

**Supplementary Table B9b:** High water levels (m CD) that reached or exceeded a 1 in 5 year return level at this site.

| **Date and time (GMT)** | **Return period (years)** | **Skew surge (m)** | **Water**  **level (m CD)** | **Astronomical tide (m CD)** |
| --- | --- | --- | --- | --- |
| 24/02/1997 20:15 | 40 | 1.49 | 12.94 | 11.45 |
| 04/01/1998 10:45 | 21 | 1.35 | 12.45 | 11.09 |
| 08/01/2005 04:15 | 7 | 1.12 | 11.18 | 10.06 |
| 19/02/1997 17:45 | 7 | 1.12 | 11.18 | 9.92 |

| **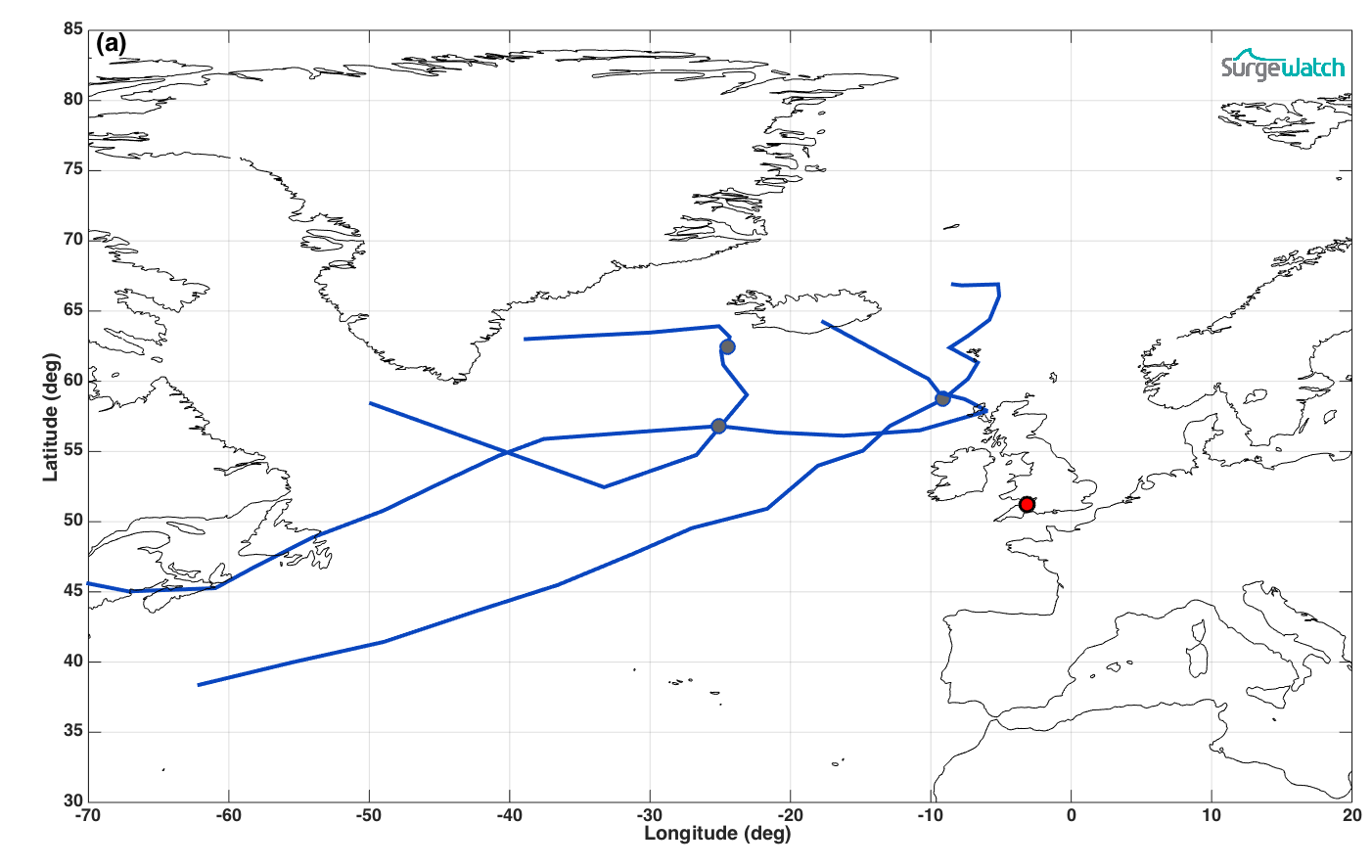** |
| --- |
| **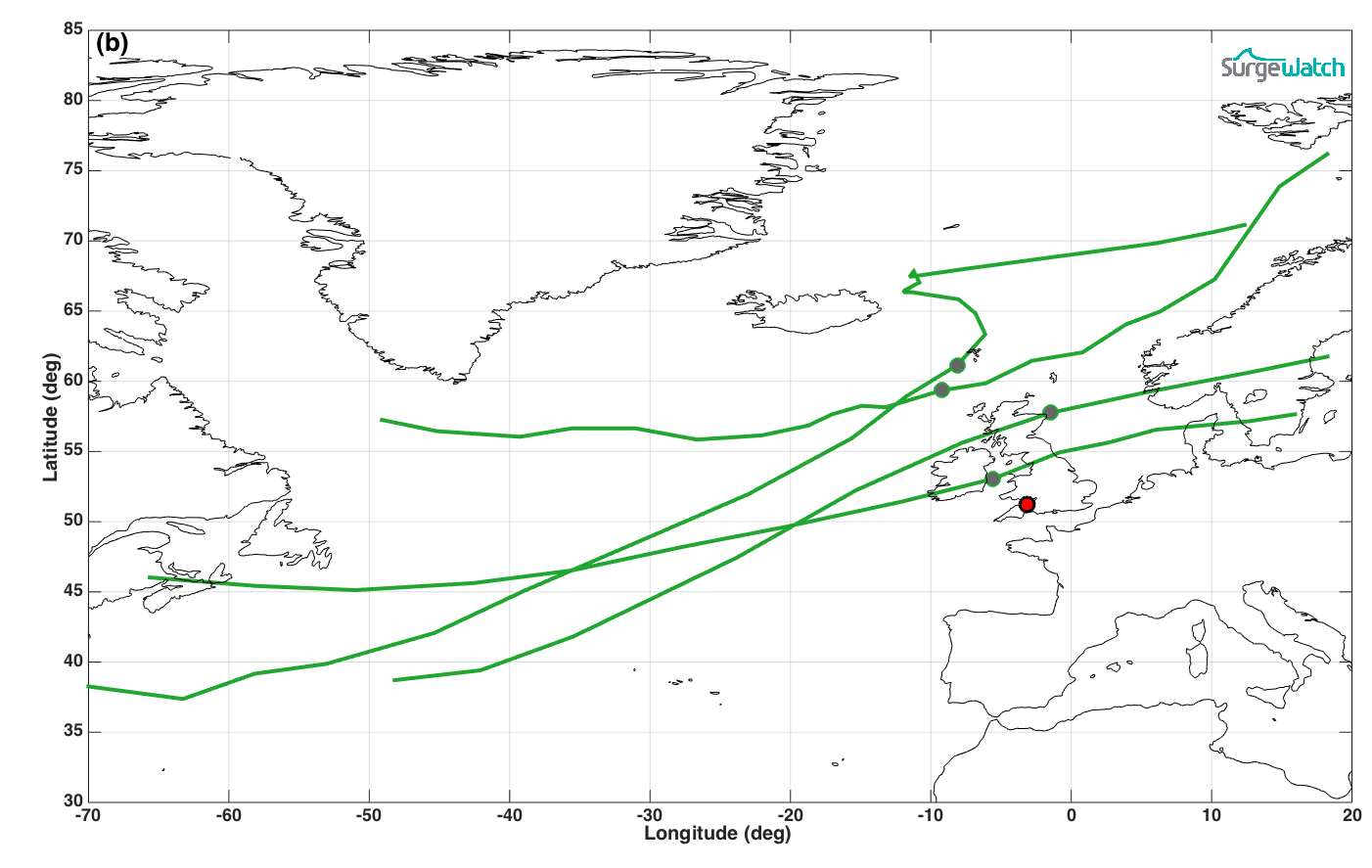** |
| **Supplementary Figure B9:** Tracks of the storms that generated (a) high water levels and (b) skew surges that reached or exceeded the 1 in 5 year return level at Hinkley Point (location shown with the blue dot). The red dot indicates the location of the storm centre at the time of highest water level or skew surge. |


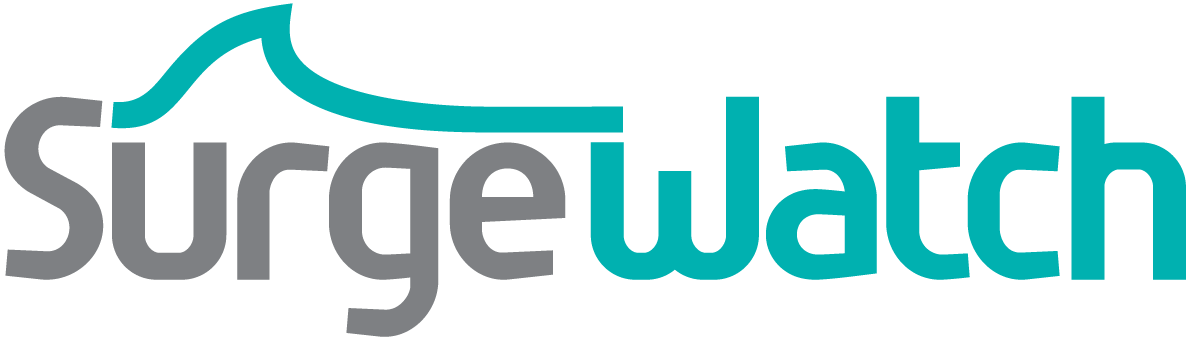
**LOCATION**

** B10.** **Avonmouth**

*Chart Datum (CD) is 6.50m below Ordnance Datum Newlyn (ODN).*

| *Observational Period* |  | *Latitude & Longitude* |
| --- | --- | --- |
| ***1961-1962,1972-1976,1979-1984,1986-2012*** |  | ***51° 30′ 27.9″ N, 02° 42′ 45.9″ W*** |

**Table B10a:** High water levels (m CD) that reached or exceeded a 1 in 5 year return level at this site.

| **Date and time (GMT)** | **Return period (years)** | **Water**  **level (m CD)** | **Astronomical tide (m CD)** | **Skew surge (m)** |
| --- | --- | --- | --- | --- |
| 13/12/1981 21:00 | 102 | 15.43 | 13.35 | 2.08 |
| 26/02/1990 08:00 | 9 | 15.00 | 14.12 | 0.87 |
| 04/12/1994 08:00 | 8 | 14.97 | 14.25 | 0.69 |
| 10/02/1997 09:15 | 6 | 14.93 | 14.24 | 0.68 |

**Table B10b:** High water levels (m CD) that reached or exceeded a 1 in 5 year return level at this site.

| **Date and time (GMT)** | **Return period (years)** | **Skew surge (m)** | **Water**  **level (m CD)** | **Astronomical tide (m CD)** |
| --- | --- | --- | --- | --- |
| 13/12/1981 21:00 | 50 | 2.08 | 15.43 | 13.35 |
| 24/02/1997 20:45 | 36 | 1.95 | 14.79 | 12.85 |
| 04/01/1998 11:15 | 11 | 1.60 | 14.08 | 12.47 |
| 15/12/1979 04:00 | 9 | 1.53 | 11.40 | 9.87 |
| 24/10/1998 21:00 | 6 | 1.41 | 13.37 | 11.90 |
| 25/12/1997 15:45 | 5 | 1.39 | 11.68 | 10.29 |

| **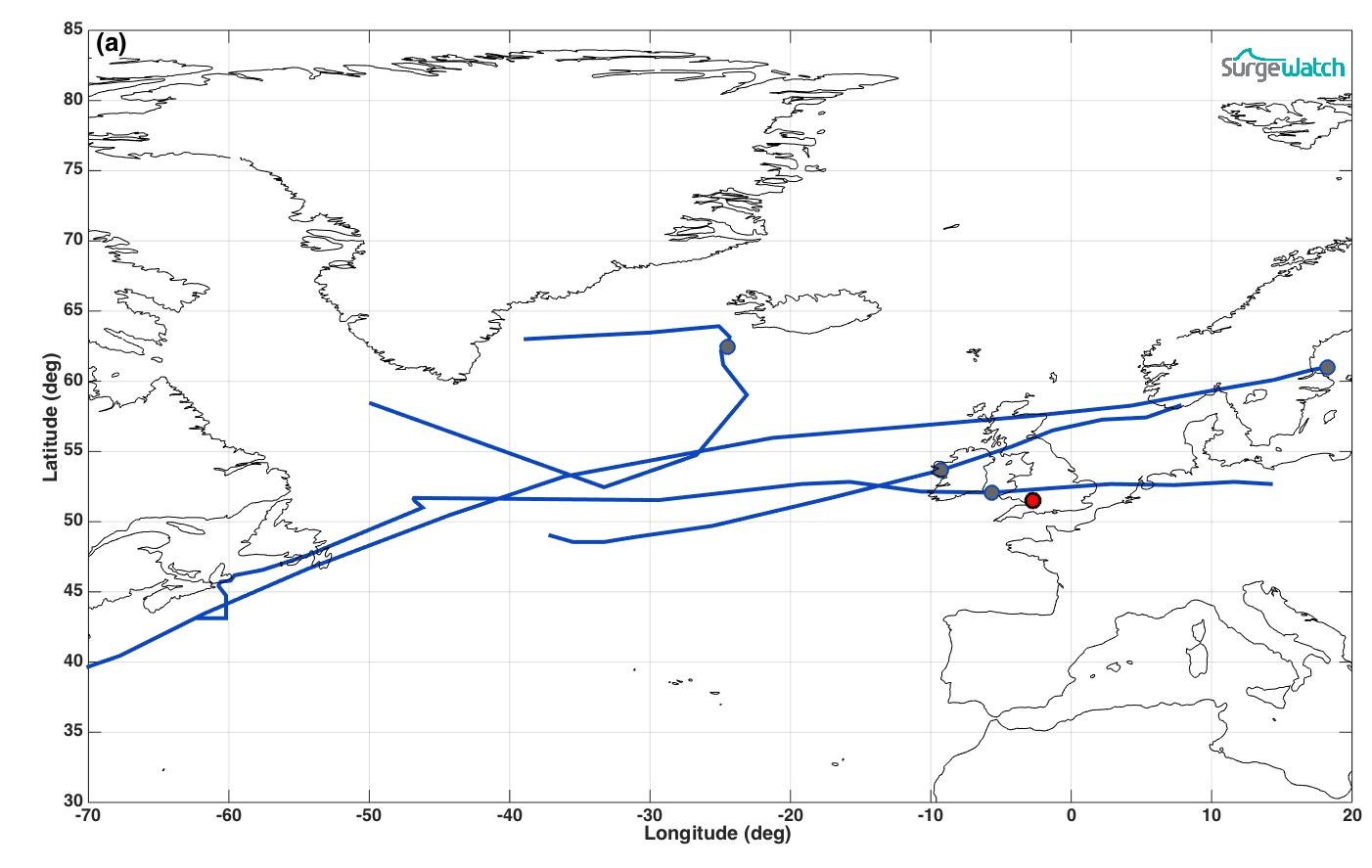**  **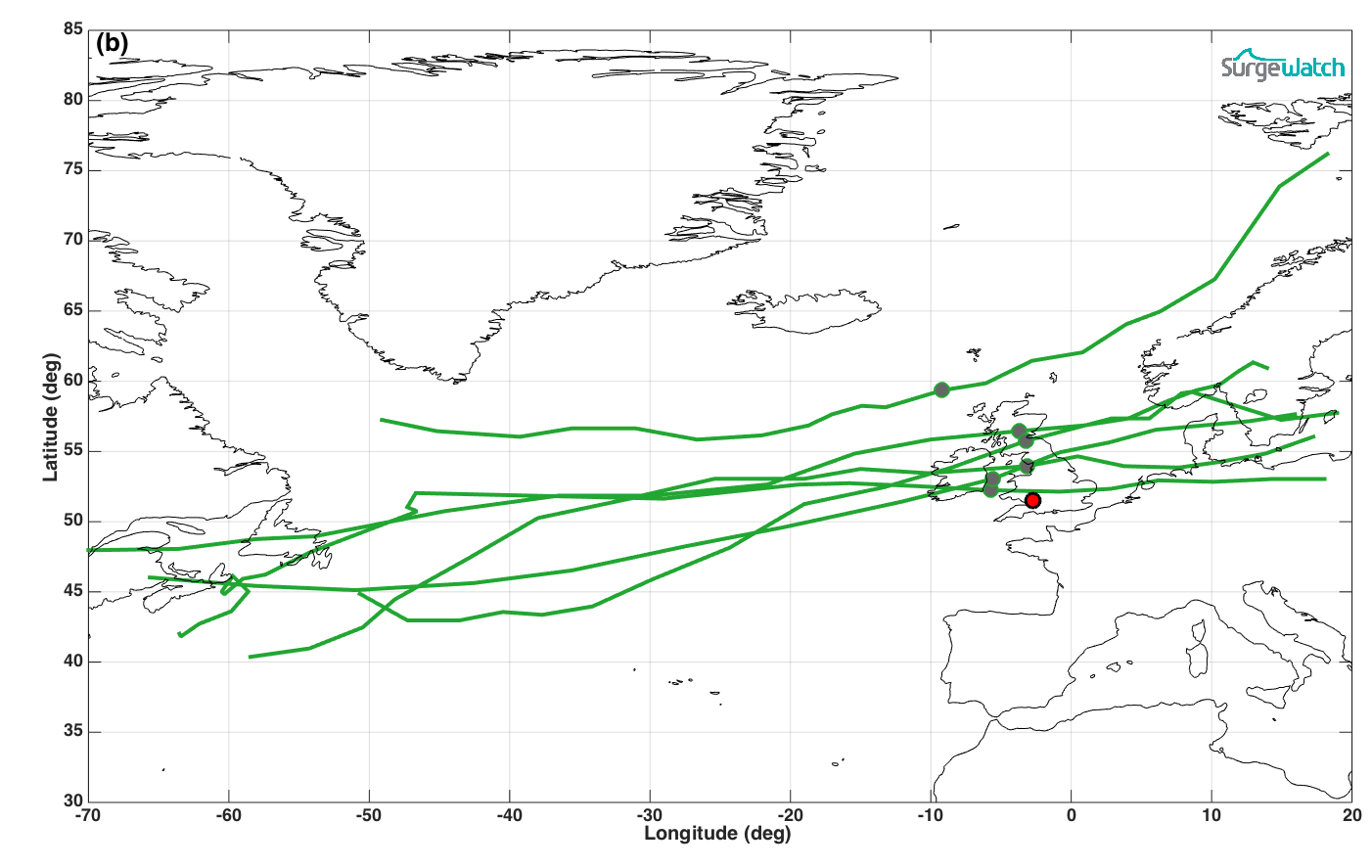** |
| --- |
| **Figure B10:** Tracks of the storms that generated (a) high water levels and (b) skew surges that reached or exceeded the 1 in 5 year return level at Avonmouth (location shown with the blue dot). The red dot indicates the location of the storm centre at the time of highest water level or skew surge. |


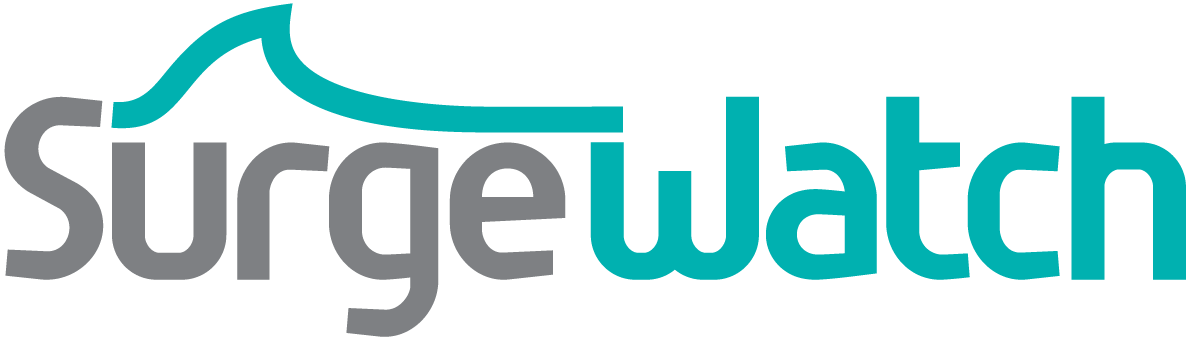
**LOCATION**

** B11.** **Newport**

*Chart Datum (CD) is 2.60m below Ordnance Datum Newlyn (ODN).*

| *Observational Period* |  | *Latitude & Longitude* |
| --- | --- | --- |
| ***1946-present*** |  | ***55° 00′ 26.8″ N, 01° 26′ 23.2″ W*** |

**Table B11a:** High water levels (m CD) that reached or exceeded a 1 in 5 year return level at this site.

| **Date and time (GMT)** | **Return period (years)** | **Water**  **level (m CD)** | **Astronomical tide (m CD)** | **Skew surge (m)** |
| --- | --- | --- | --- | --- |
| 02/03/2014 07:45 | 11 | 13.72 | 13.34 | 0.38 |
| 10/02/1997 09:00 | 8 | 13.63 | 13.01 | 0.62 |
| 08/10/2006 20:00 | 7 | 13.62 | 13.71 | -0.09 |
| 04/12/1994 07:45 | 7 | 13.59 | 13.03 | 0.56 |
| 30/03/2006 07:45 | 6 | 13.60 | 13.43 | 0.17 |

**Table B11b:** High water levels (m CD) that reached or exceeded a 1 in 5 year return level at this site.

| **Date and time (GMT)** | **Return period (years)** | **Skew surge (m)** | **Water**  **level (m CD)** | **Astronomical tide (m CD)** |
| --- | --- | --- | --- | --- |
| 24/02/1997 20:45 | 33 | 1.70 | 13.42 | 11.72 |
| 04/01/1998 11:15 | 26 | 1.65 | 12.96 | 11.26 |
| 11/02/2014 04:30 | 7 | 1.32 | 10.38 | 9.06 |
| 08/01/2005 04:45 | 5 | 1.28 | 11.60 | 10.30 |

| **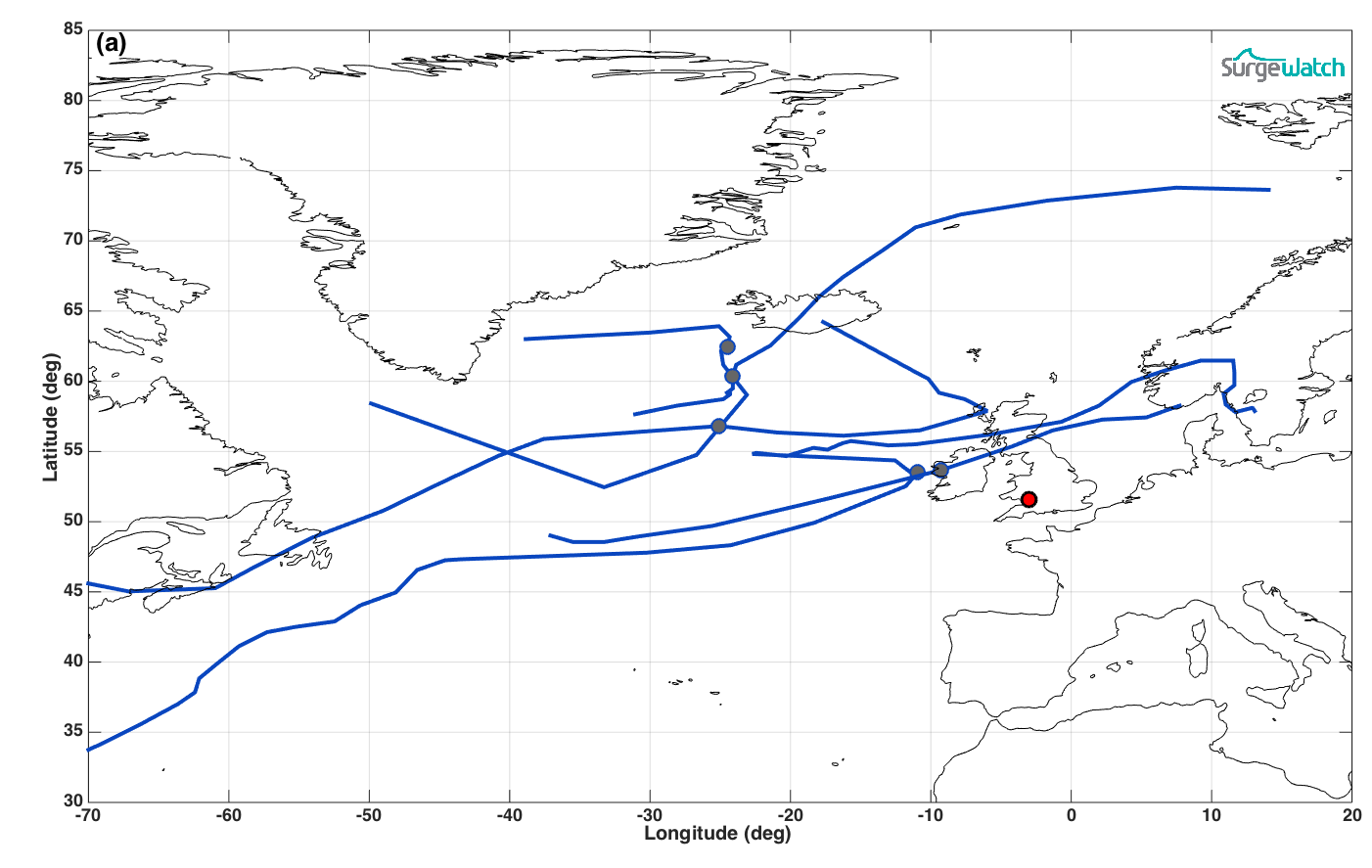**  **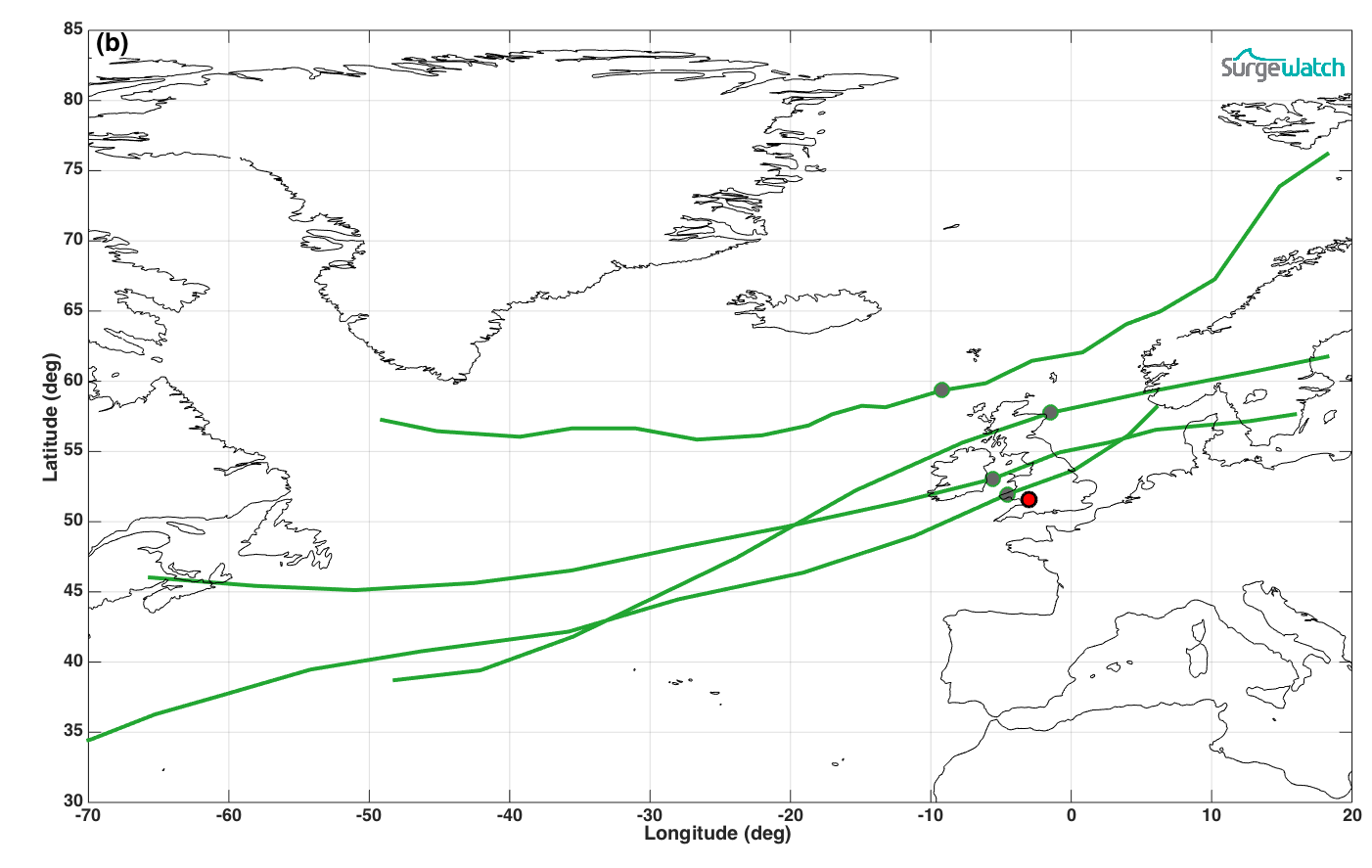** |
| --- |
| **Figure B11:** Tracks of the storms that generated (a) high water levels and (b) skew surges that reached or exceeded the 1 in 5 year return level at Newport (location shown with the blue dot). The red dot indicates the location of the storm centre at the time of highest water level or skew surge. |


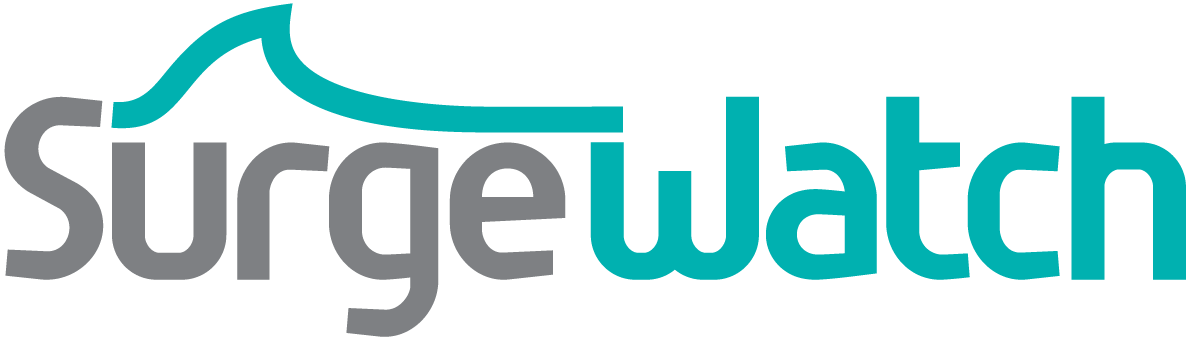
**LOCATION**

** B12.** **Mumbles**

*Chart Datum (CD) is 5.00m below Ordnance Datum Newlyn (ODN).*

| *Observational Period* |  | *Latitude & Longitude* |
| --- | --- | --- |
| ***1988-1993, 1997-present*** |  | ***51° 34′ 12.0″ N, 03° 58′ 31.7″ W*** |

**Table B12a:** High water levels (m CD) that reached or exceeded a 1 in 5 year return level at this site.

| **Date and time (GMT)** | **Return period (years)** | **Water**  **level (m CD)** | **Astronomical tide (m CD)** | **Skew surge (m)** |
| --- | --- | --- | --- | --- |
| 03/01/2014 07:15 | 9 | 10.73 | 10.21 | 0.50 |
| 29/08/1992 19:00 | 6 | 10.63 | 10.41 | 0.22 |
| 09/03/1989 07:00 | 5 | 10.60 | 10.37 | 0.23 |

**Table B12b:** High water levels (m CD) that reached or exceeded a 1 in 5 year return level at this site.

| **Date and time (GMT)** | **Return period (years)** | **Skew surge (m)** | **Water**  **level (m CD)** | **Astronomical tide (m CD)** |
| --- | --- | --- | --- | --- |
| 04/01/1998 10:15 | 26 | 1.26 | 10.37 | 9.11 |
| 16/12/1989 21:00 | 19 | 1.19 | 9.84 | 8.65 |
| 16/12/1989 09:00 | 6 | 0.98 | 9.99 | 9.02 |

| **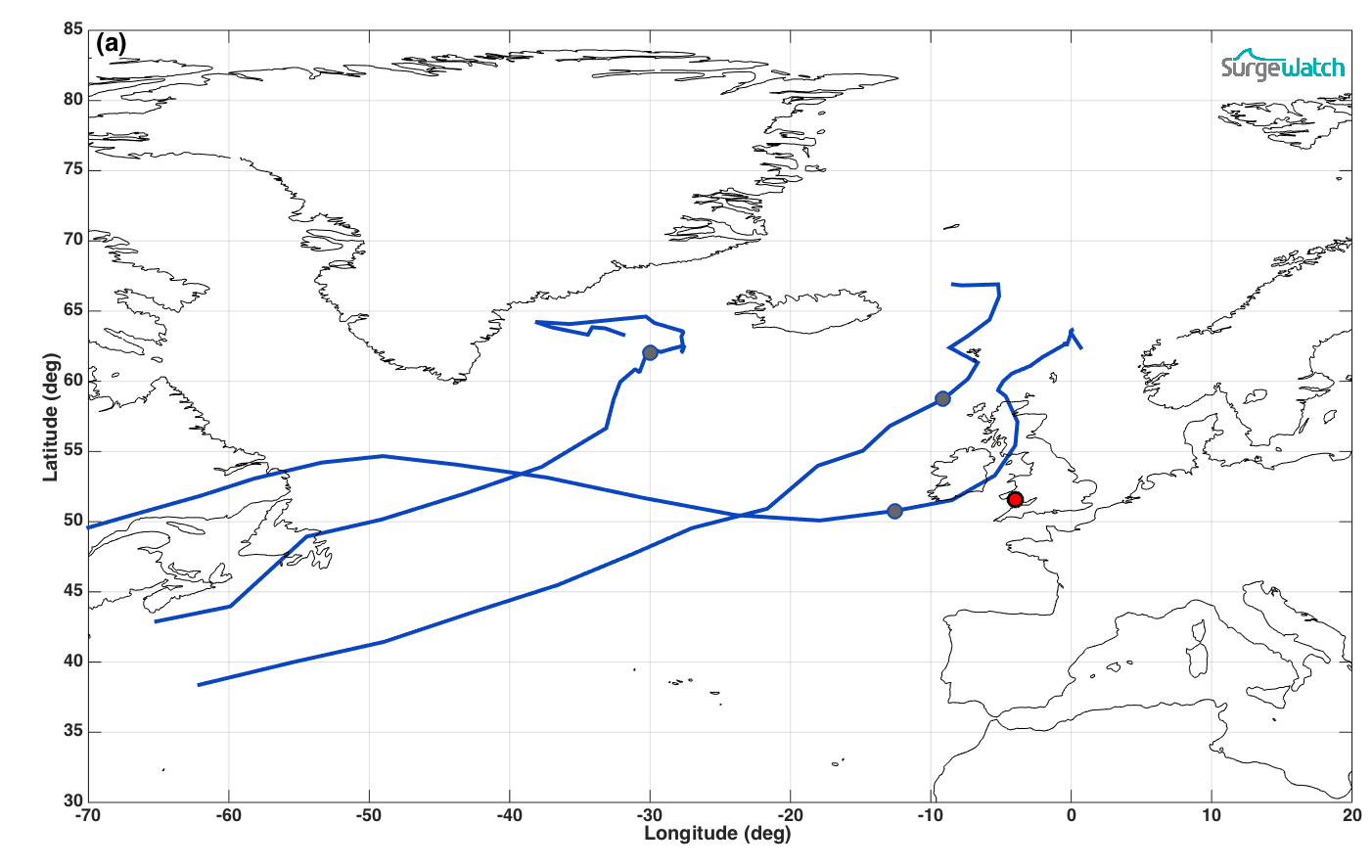**  **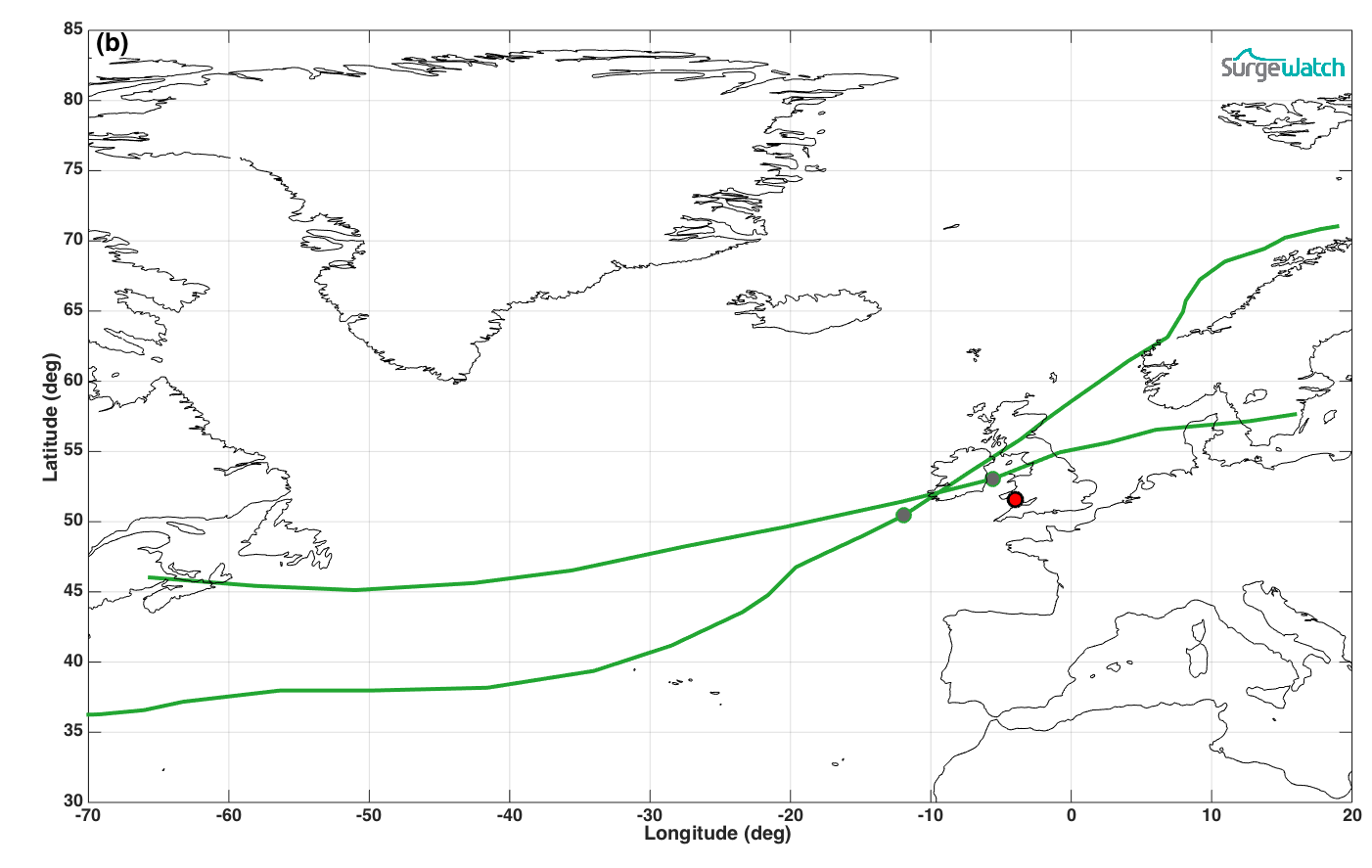** |
| --- |
| **Figure B12:** Tracks of the storms that generated (a) high water levels and (b) skew surges that reached or exceeded the 1 in 5 year return level at Mumbles (location shown with the blue dot). The red dot indicates the location of the storm centre at the time of highest water level or skew surge. |


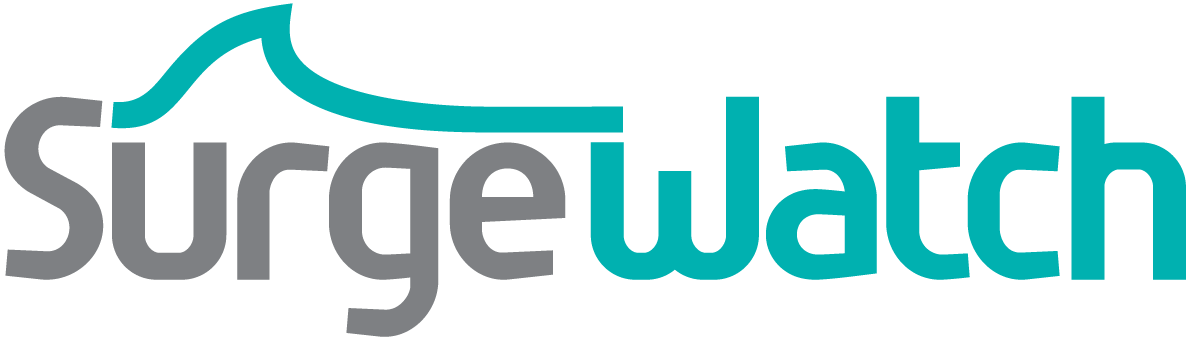
**LOCATION**

** B13.** **Milford Haven**

*Chart Datum (CD) is 3.71m below Ordnance Datum Newlyn (ODN).*

| *Observational Period* |  | *Latitude & Longitude* |
| --- | --- | --- |
| ***1961-1962,1964-1965,1967-present*** |  | ***51° 42′ 26.6″ N, 05° 03′ 06.4″ W*** |

**Table B13a:** High water levels (m CD) that reached or exceeded a 1 in 5 year return level at this site.

| **Date and time (GMT)** | **Return period (years)** | **Water**  **level (m CD)** | **Astronomical tide (m CD)** | **Skew surge (m)** |
| --- | --- | --- | --- | --- |
| 23/09/1953 18:00 | 34 | 8.09 | 7.83 | 0.26 |
| 24/09/1953 19:00 | 24 | 8.06 | 7.98 | 0.08 |
| 07/03/1962 07:00 | 21 | 8.06 | 7.83 | 0.23 |
| 10/03/2008 07:45 | 19 | 8.18 | 7.57 | 0.60 |
| 24/10/1961 06:00 | 17 | 8.03 | 7.45 | 0.58 |
| 10/02/1997 08:15 | 15 | 8.12 | 7.69 | 0.42 |
| 03/02/2014 08:30 | 12 | 8.14 | 7.64 | 0.50 |
| 06/03/1954 19:00 | 12 | 7.97 | 7.54 | 0.43 |
| 02/03/2014 06:45 | 10 | 8.13 | 7.79 | 0.34 |
| 08/10/2006 18:45 | 10 | 8.11 | 7.94 | 0.16 |
| 24/10/1961 18:00 | 9 | 7.97 | 7.55 | 0.42 |
| 07/03/1962 19:00 | 9 | 7.97 | 7.68 | 0.29 |
| 29/08/1992 19:00 | 8 | 8.04 | 7.71 | 0.32 |
| 30/03/2006 06:30 | 8 | 8.07 | 7.71 | 0.35 |
| 05/10/1967 19:00 | 7 | 7.96 | 7.83 | 0.12 |
| 07/03/1954 08:00 | 7 | 7.91 | 7.73 | 0.18 |
| 13/12/1981 19:00 | 6 | 7.98 | 7.06 | 0.75 |
| 01/02/2014 07:15 | 6 | 8.07 | 7.79 | 0.26 |
| 09/02/1974 08:00 | 5 | 7.95 | 7.72 | 0.22 |
| 08/02/1974 07:00 | 5 | 7.94 | 7.77 | 0.17 |
| 01/02/2002 08:30 | 5 | 8.02 | 7.49 | 0.53 |
| 07/04/1962 08:00 | 5 | 7.91 | 7.68 | 0.23 |

**Table B13b:** Skew surges (m) that reached or exceeded a 1 in 5 year return level at this site.

| **Date and time (GMT)** | **Return period (years)** | **Skew surge (m)** | **Water**  **level (m CD)** | **Astronomical tide (m CD)** |
| --- | --- | --- | --- | --- |
| 15/03/1977 15:00 | 73 | 1.21 | 6.43 | 5.22 |
| 14/02/2014 18:00 | 38 | 1.13 | 7.59 | 6.42 |
| 16/12/1989 21:00 | 15 | 1.03 | 7.63 | 6.60 |
| 04/01/1998 09:45 | 11 | 0.99 | 7.75 | 6.70 |
| 19/02/1997 16:45 | 7 | 0.94 | 6.87 | 5.93 |
| 17/01/1962 04:00 | 7 | 0.93 | 6.63 | 5.67 |
| 17/01/1969 18:00 | 5 | 0.91 | 7.62 | 6.70 |

| **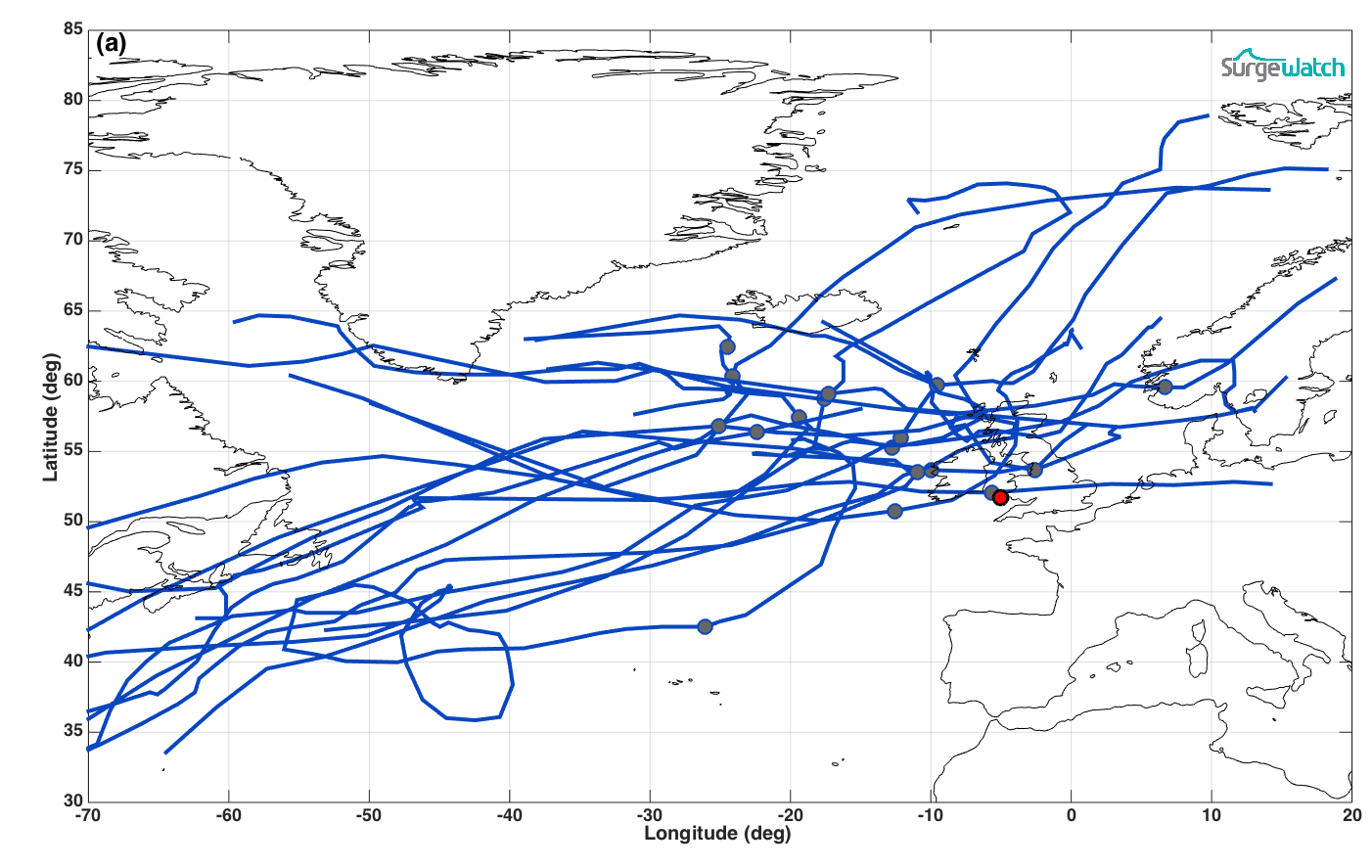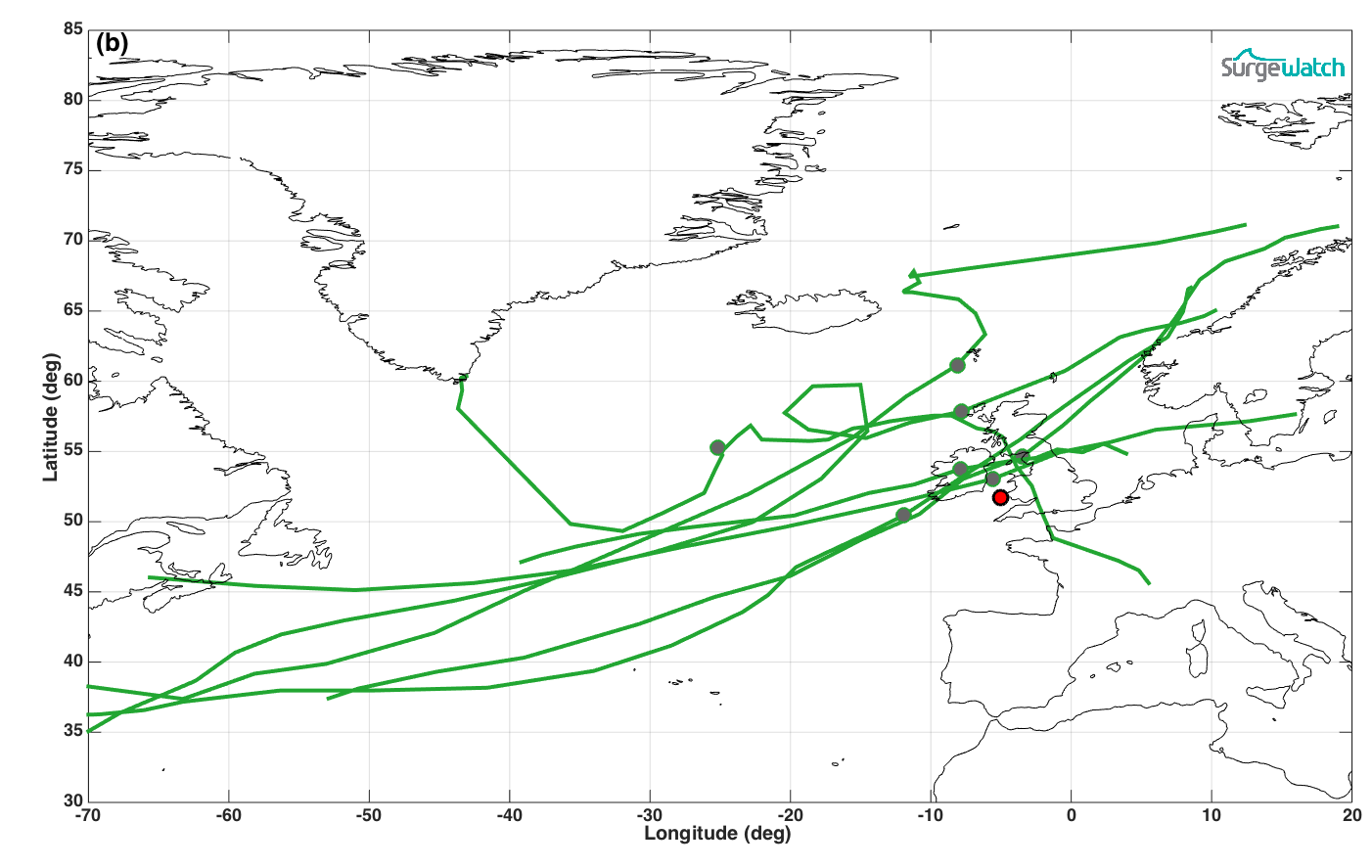** |
| --- |
| **Figure B13:** Tracks of the storms that generated (a) high water levels and (b) skew surges that reached or exceeded the 1 in 5 year return level at Milford Haven (location shown with the blue dot). The red dot indicates the location of the storm centre at the time of highest water level or skew surge. |


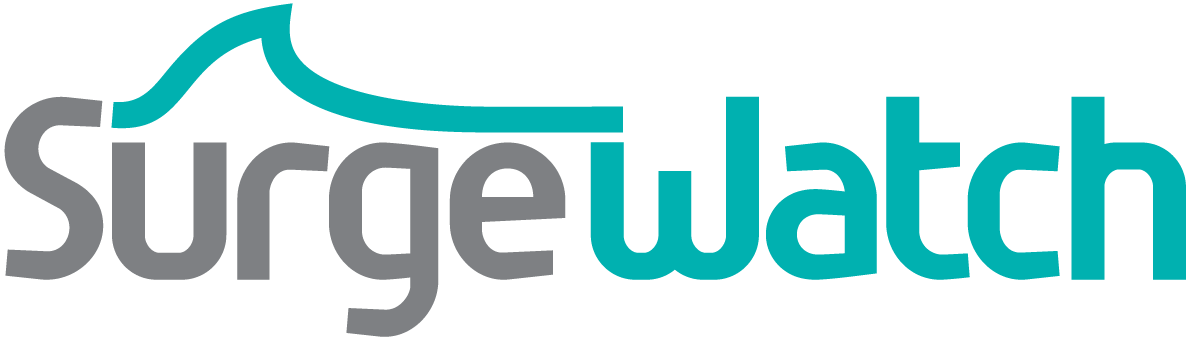
**LOCATION**

** B14.** **Fishguard**

*Chart Datum (CD) is 2.44m below Ordnance Datum Newlyn (ODN).*

| *Observational Period* |  | *Latitude & Longitude* |
| --- | --- | --- |
| ***1963-1971,1973-present*** |  | ***52° 00′ 47.6″ N, 04° 59′ 01.5″ W*** |

**Supplementary Table B14a:** High water levels (m CD) that reached or exceeded a 1 in 5 year return level at this site.

| **Date and time (GMT)** | **Return period (years)** | **Water**  **level (m CD)** | **Astronomical tide (m CD)** | **Skew surge (m)** |
| --- | --- | --- | --- | --- |
| 10/02/1997 09:15 | 20 | 5.78 | 5.36 | 0.42 |
| 03/01/2014 08:00 | 17 | 5.80 | 5.27 | 0.49 |
| 03/02/2014 09:30 | 16 | 5.80 | 5.14 | 0.66 |
| 29/08/1992 20:00 | 11 | 5.72 | 5.40 | 0.32 |
| 30/03/2006 07:45 | 10 | 5.74 | 5.42 | 0.33 |
| 08/02/1966 09:00 | 8 | 5.62 | 5.29 | 0.33 |
| 30/01/1975 09:00 | 8 | 5.64 | 5.41 | 0.23 |
| 10/03/2008 08:45 | 8 | 5.72 | 5.20 | 0.52 |
| 01/02/2002 09:30 | 7 | 5.70 | 5.38 | 0.31 |
| 08/10/2006 20:00 | 7 | 5.71 | 5.62 | 0.09 |
| 17/10/2012 20:15 | 7 | 5.71 | 5.37 | 0.34 |

**Supplementary Table B14b:** Skew surges (m) that reached or exceeded a 1 in 5 year return level at this site.

| **Date and time (GMT)** | **Return period (years)** | **Skew surge (m)** | **Water**  **level (m CD)** | **Astronomical tide (m CD)** |
| --- | --- | --- | --- | --- |
| 14/02/2014 19:30 | 87 | 1.09 | 5.29 | 4.21 |
| 08/02/2014 14:00 | 23 | 0.93 | 4.36 | 3.43 |
| 16/12/1989 22:00 | 18 | 0.90 | 5.33 | 4.44 |
| 23/12/2013 23:15 | 17 | 0.90 | 4.67 | 3.75 |
| 04/02/2014 22:30 | 15 | 0.88 | 5.23 | 4.34 |
| 27/12/2013 01:15 | 14 | 0.87 | 4.39 | 3.48 |
| 10/11/1963 15:00 | 10 | 0.83 | 4.61 | 3.78 |
| 23/12/2013 10:30 | 10 | 0.83 | 4.96 | 4.13 |
| 08/02/2014 01:00 | 8 | 0.80 | 4.24 | 3.43 |
| 19/02/1997 17:45 | 7 | 0.79 | 4.74 | 3.95 |

| **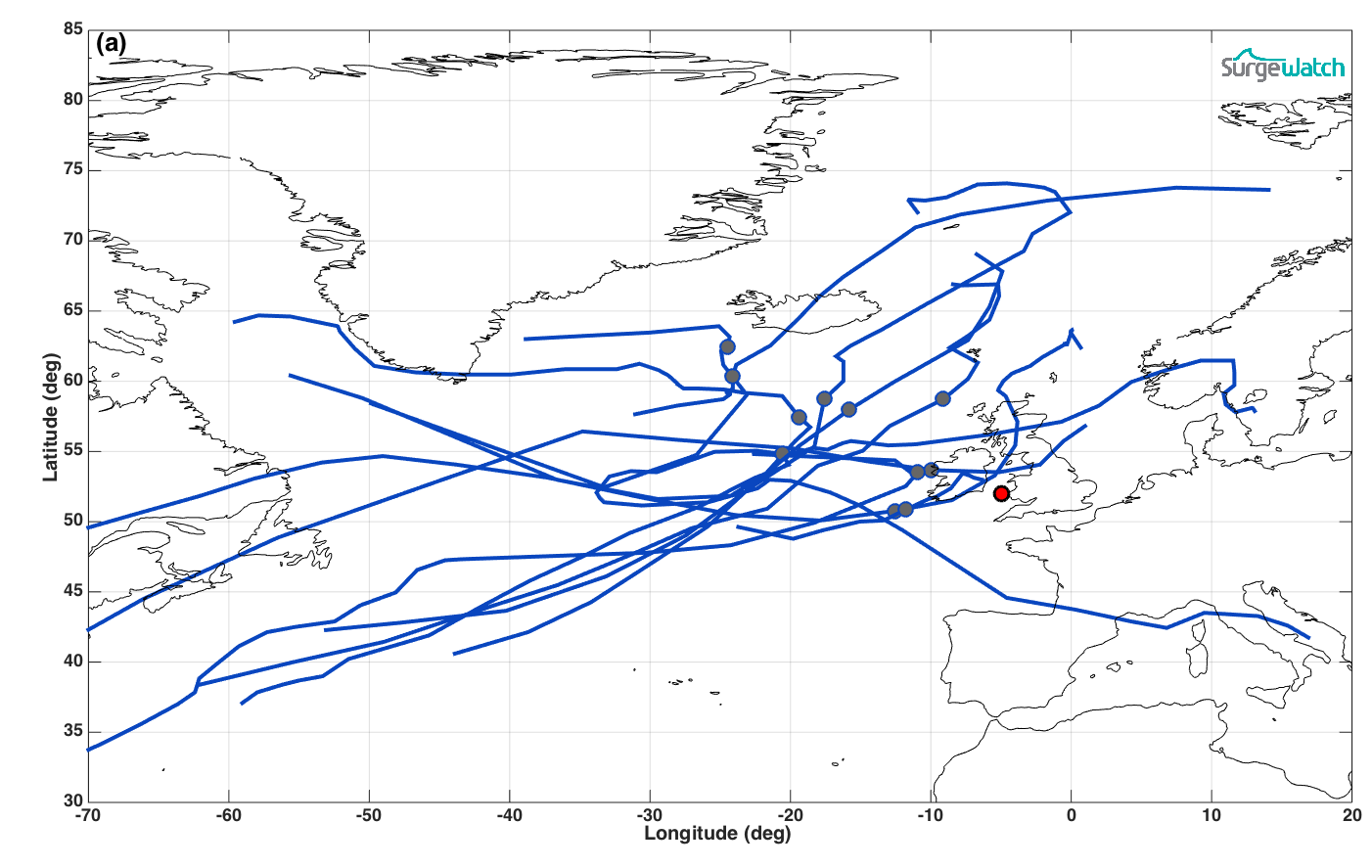**  **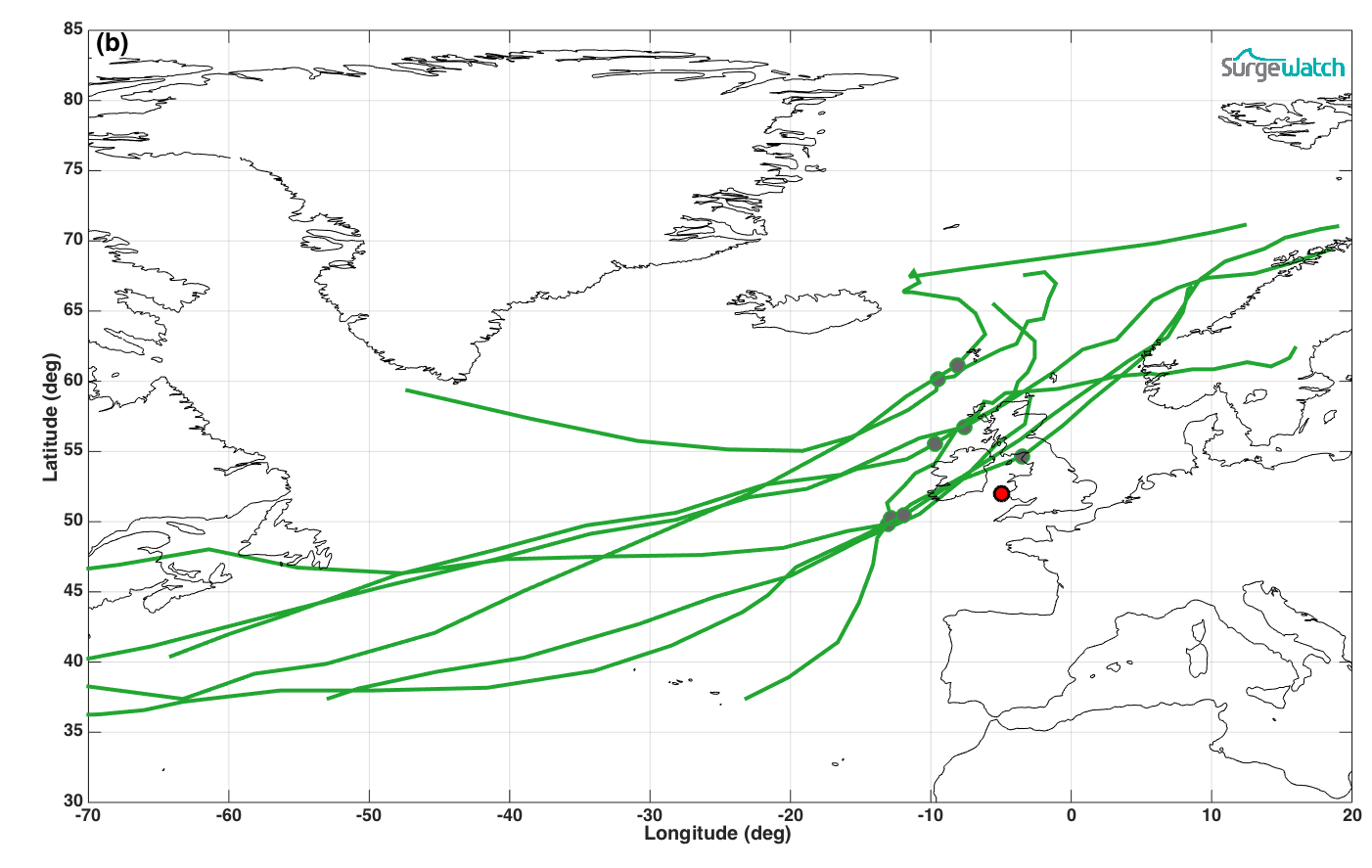** |
| --- |
| **Supplementary Figure B14:** Tracks of the storms that generated (a) high water levels and (b) skew surges that reached or exceeded the 1 in 5 year return level at Fishguard (location shown with the blue dot). The red dot indicates the location of the storm centre at the time of highest water level or skew surge. |


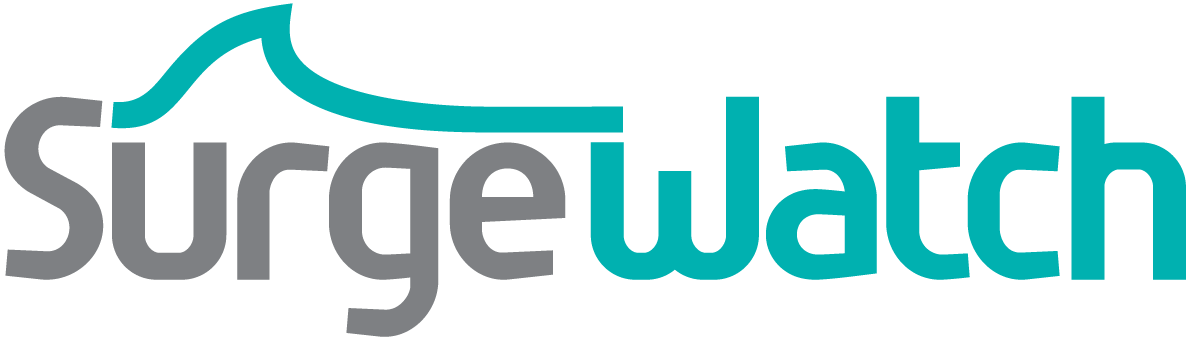
**LOCATION**

** B15.** **Barmouth**

*Chart Datum (CD) is 2.44m below Ordnance Datum Newlyn (ODN).*

| *Observational Period* |  | *Latitude & Longitude* |
| --- | --- | --- |
| ***1991-2003, 2005-present*** |  | ***52° 43′ 09.6″N, 04° 02′ 42.1″W*** |

**Supplementary Table B15a:** High water levels (m CD) that reached or exceeded a 1 in 5 year return level at this site.

| **Date and time (GMT)** | **Return period (years)** | **Water**  **level (m CD)** | **Astronomical tide (m CD)** | **Skew surge (m)** |
| --- | --- | --- | --- | --- |
| 10/02/1997 10:00 | 25 | 6.36 | 5.68 | 0.68 |
| 03/01/2014 09:15 | 18 | 6.36 | 5.62 | 0.74 |
| 30/03/2006 08:45 | 6 | 6.19 | 5.63 | 0.55 |
| 25/11/2000 20:15 | 5 | 6.15 | 5.16 | 0.90 |

**Supplementary Table B15b:** Skew surges (m) that reached or exceeded a 1 in 5 year return level at this site.

| **Date and time (GMT)** | **Return period (years)** | **Skew surge (m)** | **Water**  **level (m CD)** | **Astronomical tide (m CD)** |
| --- | --- | --- | --- | --- |
| 12/02/2014 18:30 | 34 | 1.41 | 5.59 | 4.08 |
| 24/12/1997 18:00 | 31 | 1.40 | 5.22 | 3.41 |
| 19/02/1997 18:30 | 18 | 1.33 | 5.47 | 4.12 |
| 08/02/2014 15:15 | 9 | 1.22 | 4.88 | 3.64 |
| 25/12/1997 17:00 | 7 | 1.19 | 5.16 | 3.96 |

| **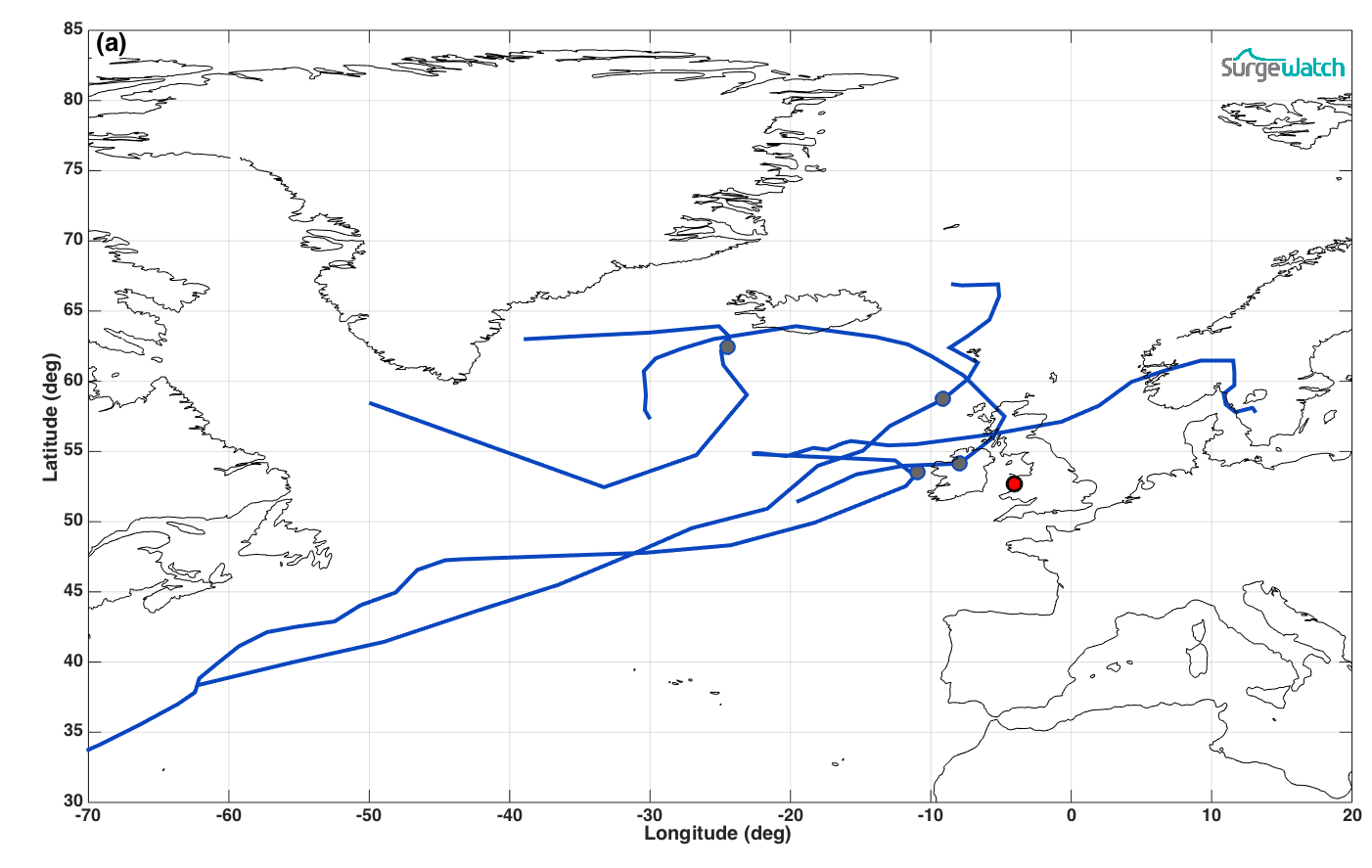**  **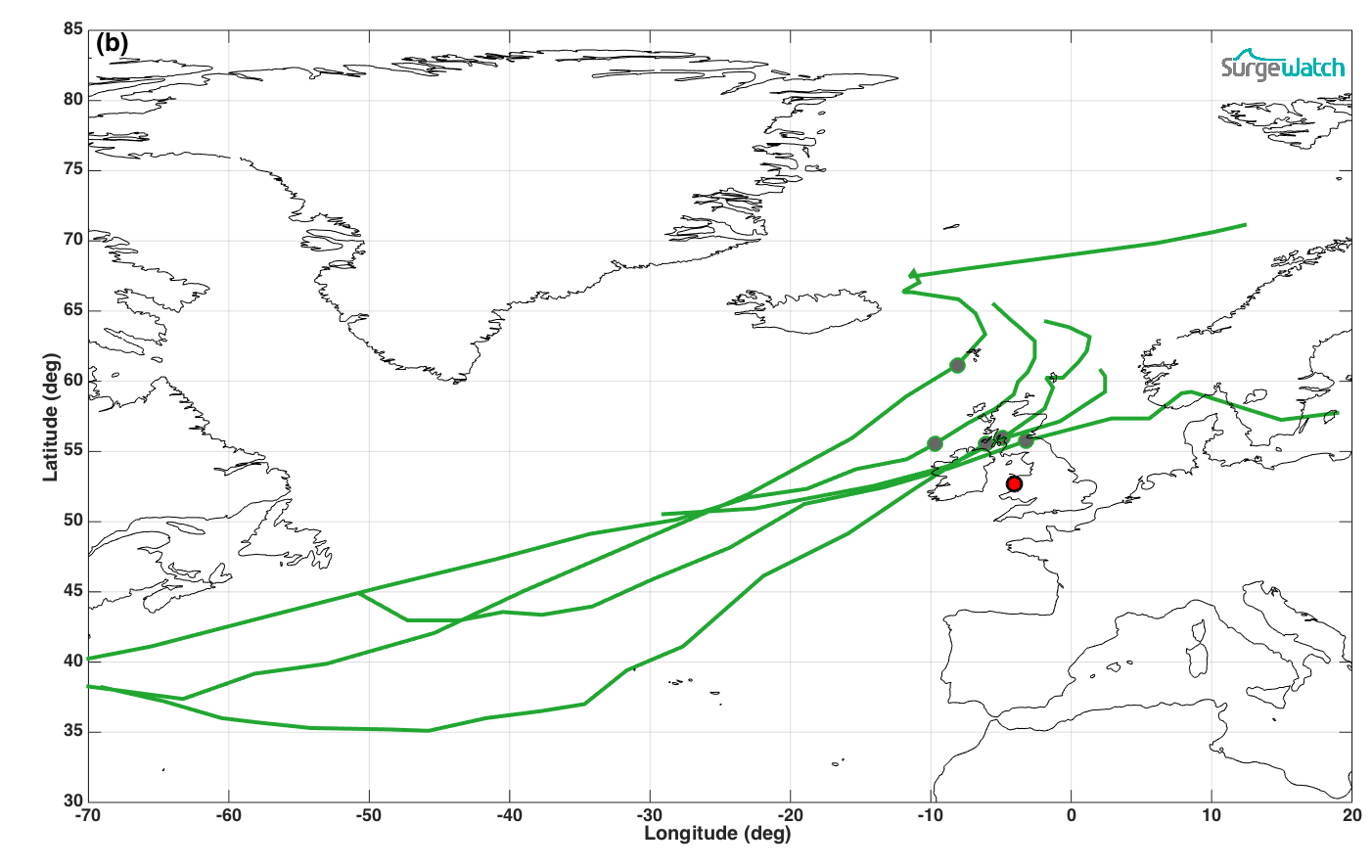** |
| --- |
| **Supplementary Figure B15:** Tracks of the storms that generated (a) high water levels and (b) skew surges that reached or exceeded the 1 in 5 year return level at Barmouth (location shown with the blue dot). The red dot indicates the location of the storm centre at the time of highest water level or skew surge. |


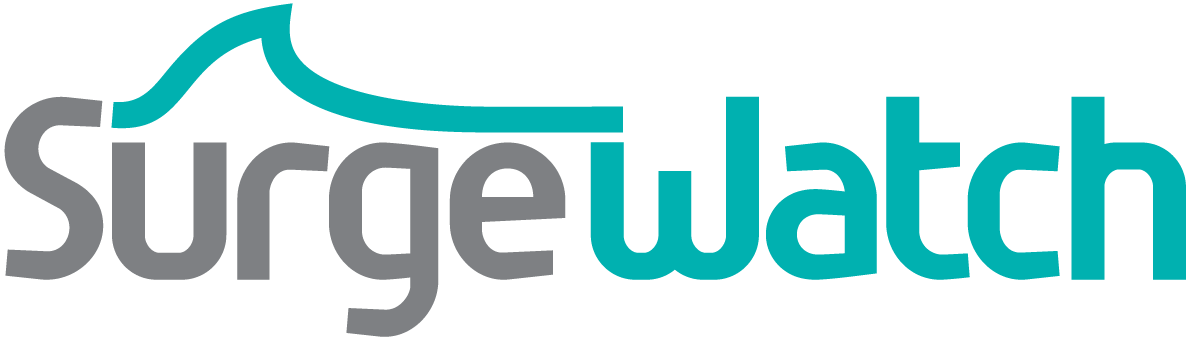
**LOCATION**

** B16.** **Holyhead**

*Chart Datum (CD) is 3.05m below Ordnance Datum Newlyn (ODN).*

| *Observational Period* |  | *Latitude & Longitude* |
| --- | --- | --- |
| ***1964-1969, 1981, 1983, 1985, 1988, 1990-1991, 1995-present*** |  | ***53° 18′ 50.2″ N, 04° 37′ 13.5″ W*** |

**Supplementary Table B16a:** High water levels (m CD) that reached or exceeded a 1 in 5 year return level at this site.

| **Date and time (GMT)** | **Return period (years)** | **Water**  **level (m CD)** | **Astronomical tide (m CD)** | **Skew surge (m)** |
| --- | --- | --- | --- | --- |
| 01/02/2002 12:45 | 92 | 6.86 | 6.12 | 0.74 |
| 03/01/2014 11:00 | 20 | 6.74 | 6.15 | 0.54 |
| 10/02/1997 12:15 | 17 | 6.68 | 6.20 | 0.48 |
| 12/12/2000 23:30 | 10 | 6.64 | 5.85 | 0.71 |
| 23/12/1999 22:45 | 7 | 6.60 | 5.95 | 0.65 |
| 30/03/2006 10:45 | 6 | 6.59 | 6.19 | 0.40 |
| 03/02/2014 12:30 | 5 | 6.61 | 6.11 | 0.48 |

**Supplementary Table B16b:** Skew surges (m) that reached or exceeded a 1 in 5 year return level at this site.

| **Date and time (GMT)** | **Return period (years)** | **Skew surge (m)** | **Water**  **level (m CD)** | **Astronomical tide (m CD)** |
| --- | --- | --- | --- | --- |
| 27/12/2013 05:15 | 36 | 0.94 | 5.60 | 4.65 |
| 02/01/1984 22:00 | 21 | 0.90 | 6.21 | 5.31 |
| 17/12/1989 01:00 | 16 | 0.87 | 6.21 | 5.33 |
| 01/12/1966 12:00 | 11 | 0.85 | 6.18 | 5.33 |
| 01/02/1988 10:00 | 9 | 0.83 | 6.04 | 5.21 |
| 09/02/1988 14:00 | 6 | 0.78 | 5.82 | 5.04 |
| 06/01/2014 14:00 | 5 | 0.78 | 6.56 | 5.78 |

| **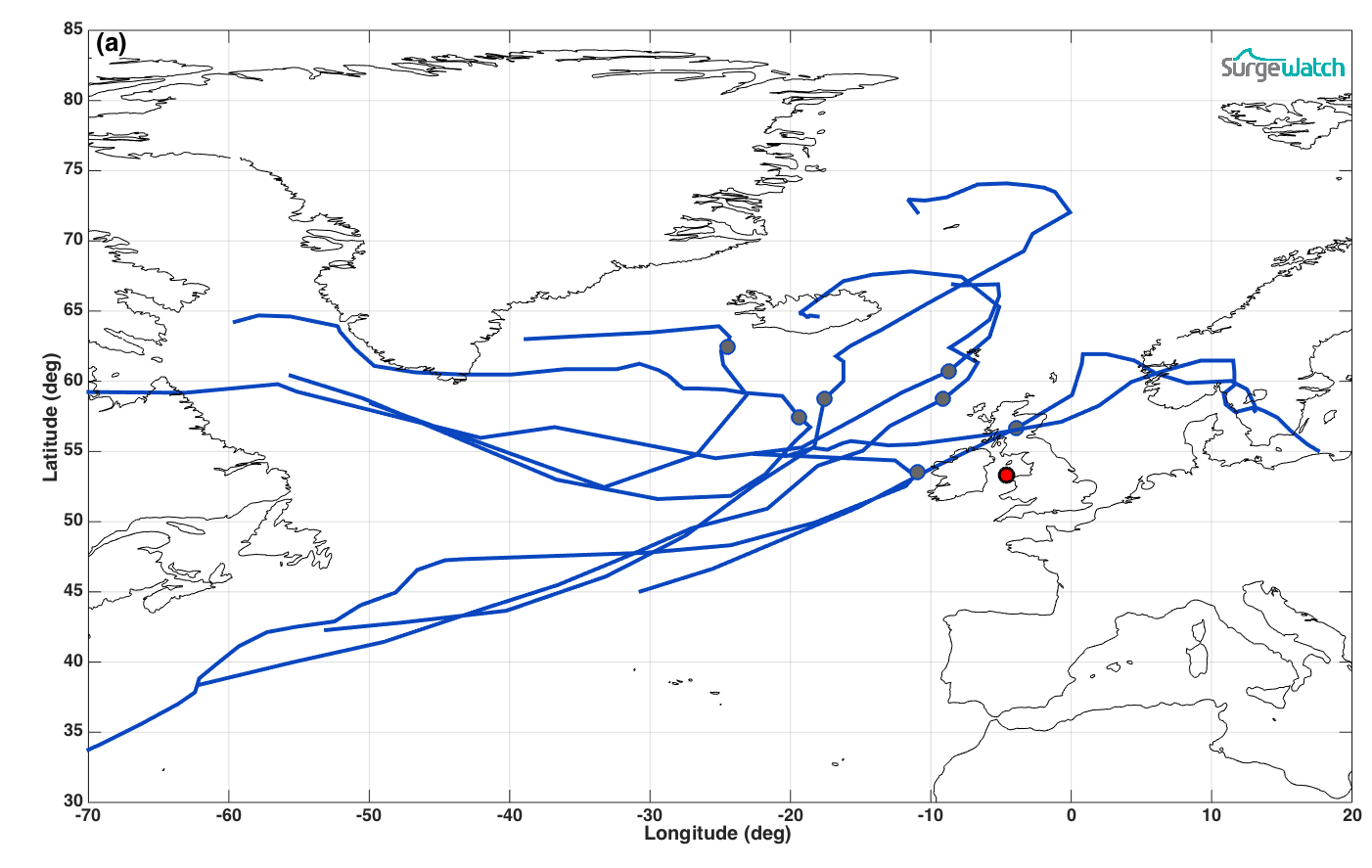**  **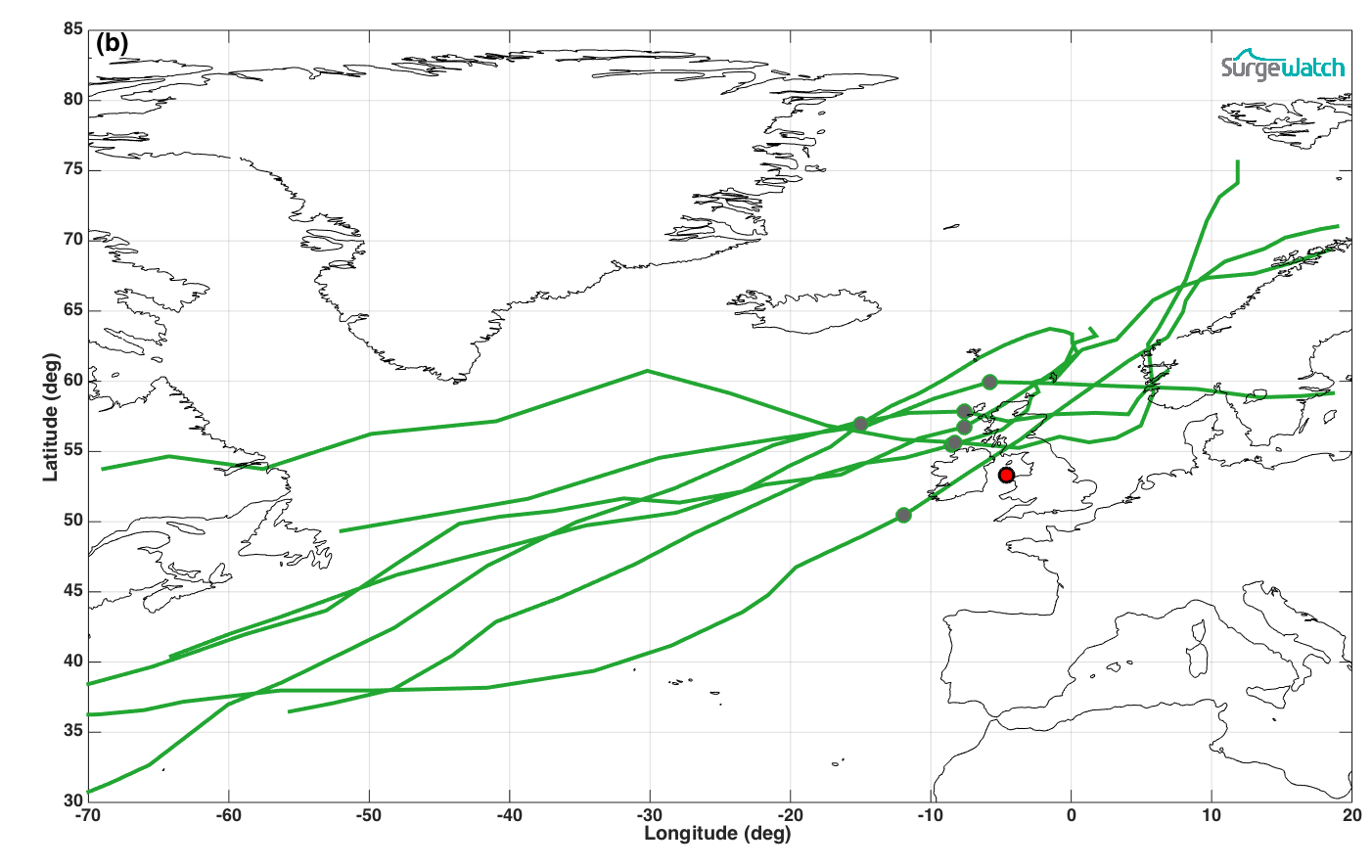** |
| --- |
| **Supplementary Figure B16:** Tracks of the storms that generated (a) high water levels and (b) skew surges that reached or exceeded the 1 in 5 year return level at Holyhead (location shown with the blue dot). The red dot indicates the location of the storm centre at the time of highest water level or skew surge. |


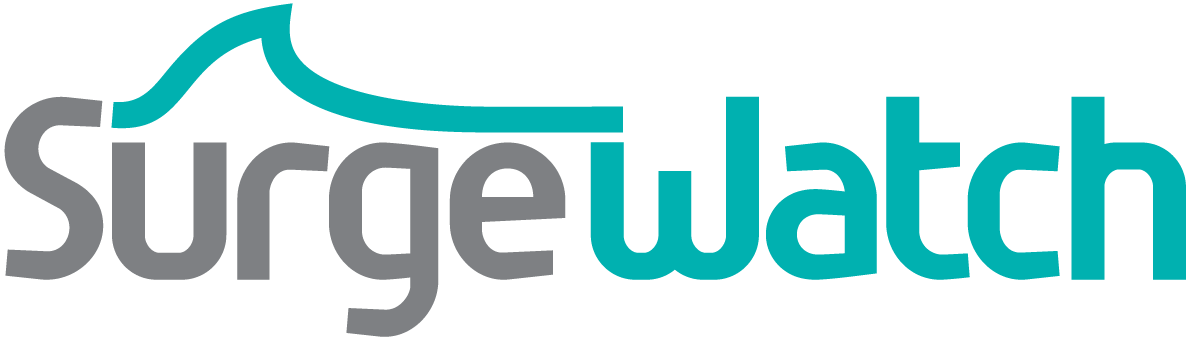
**LOCATION**

** B17.** **Llandudno**

*Chart Datum (CD) is 3.85m below Ordnance Datum Newlyn (ODN).*

| *Observational Period* |  | *Latitude & Longitude* |
| --- | --- | --- |
| ***1971, 1994-present*** |  | ***53° 19′ 54.0″ N, 03° 49′ 30.8″ W*** |

**Supplementary Table B17a:** High water levels (m CD) that reached or exceeded a 1 in 5 year return level at this site.

| **Date and time (GMT)** | **Return period (years)** | **Water**  **level (m CD)** | **Astronomical tide (m CD)** | **Skew surge (m)** |
| --- | --- | --- | --- | --- |
| 10/02/1997 12:30 | 28 | 8.95 | 8.49 | 0.46 |
| 03/01/2014 12:00 | 17 | 8.93 | 8.38 | 0.52 |
| 01/02/2002 13:15 | 17 | 8.90 | 8.24 | 0.66 |
| 05/12/2013 12:00 | 15 | 8.91 | 8.21 | 0.71 |

**Supplementary Table B17b:** Skew surges (m) that reached or exceeded a 1 in 5 year return level at this site.

| **Date and time (GMT)** | **Return period (years)** | **Skew surge (m)** | **Water**  **level (m CD)** | **Astronomical tide (m CD)** |
| --- | --- | --- | --- | --- |
| 19/02/1997 21:00 | 14 | 0.91 | 7.60 | 6.58 |
| 08/01/2005 08:15 | 14 | 0.91 | 7.74 | 6.82 |
| 27/12/2013 05:30 | 7 | 0.84 | 7.00 | 6.15 |
| 24/12/1997 19:30 | 6 | 0.83 | 6.89 | 5.96 |

| **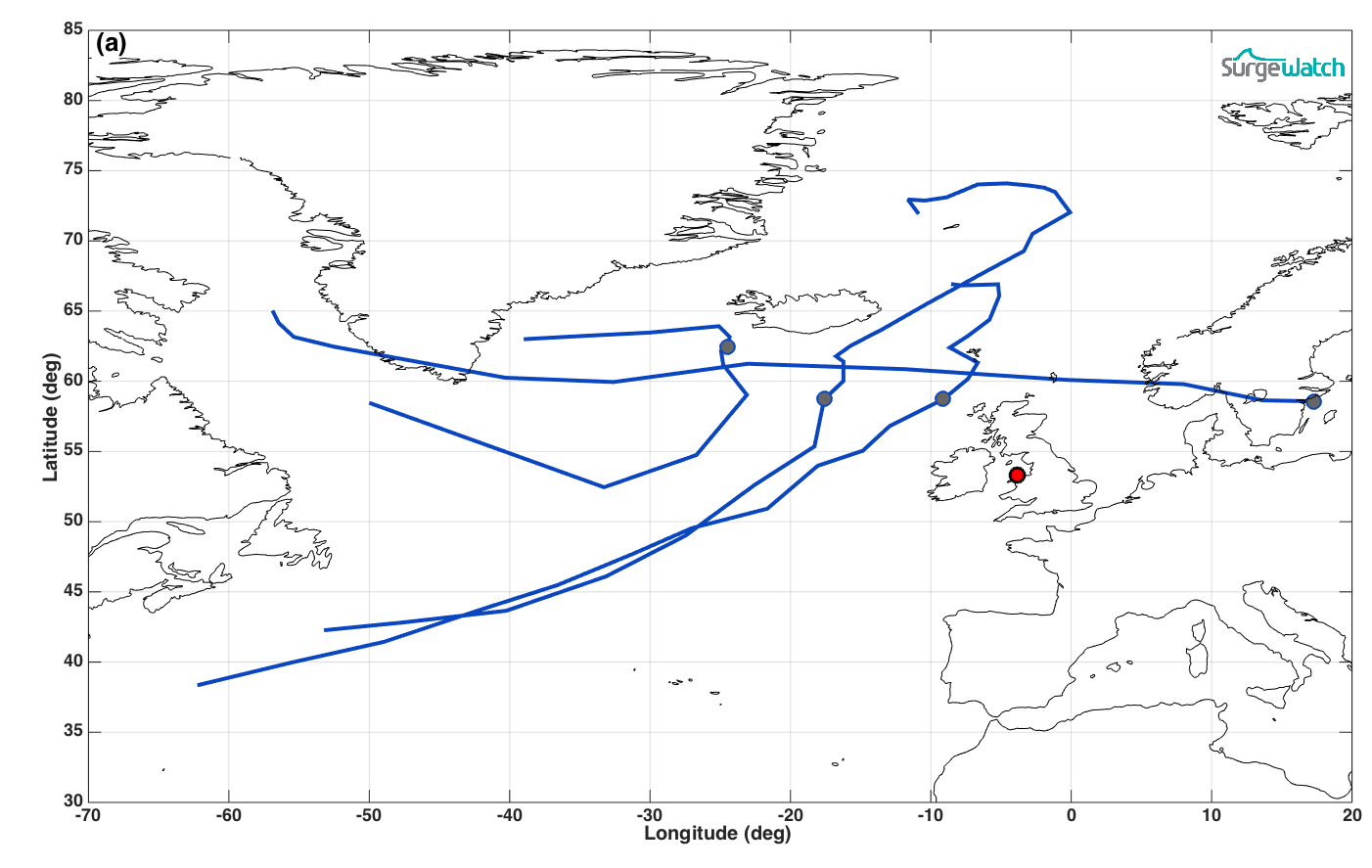**  **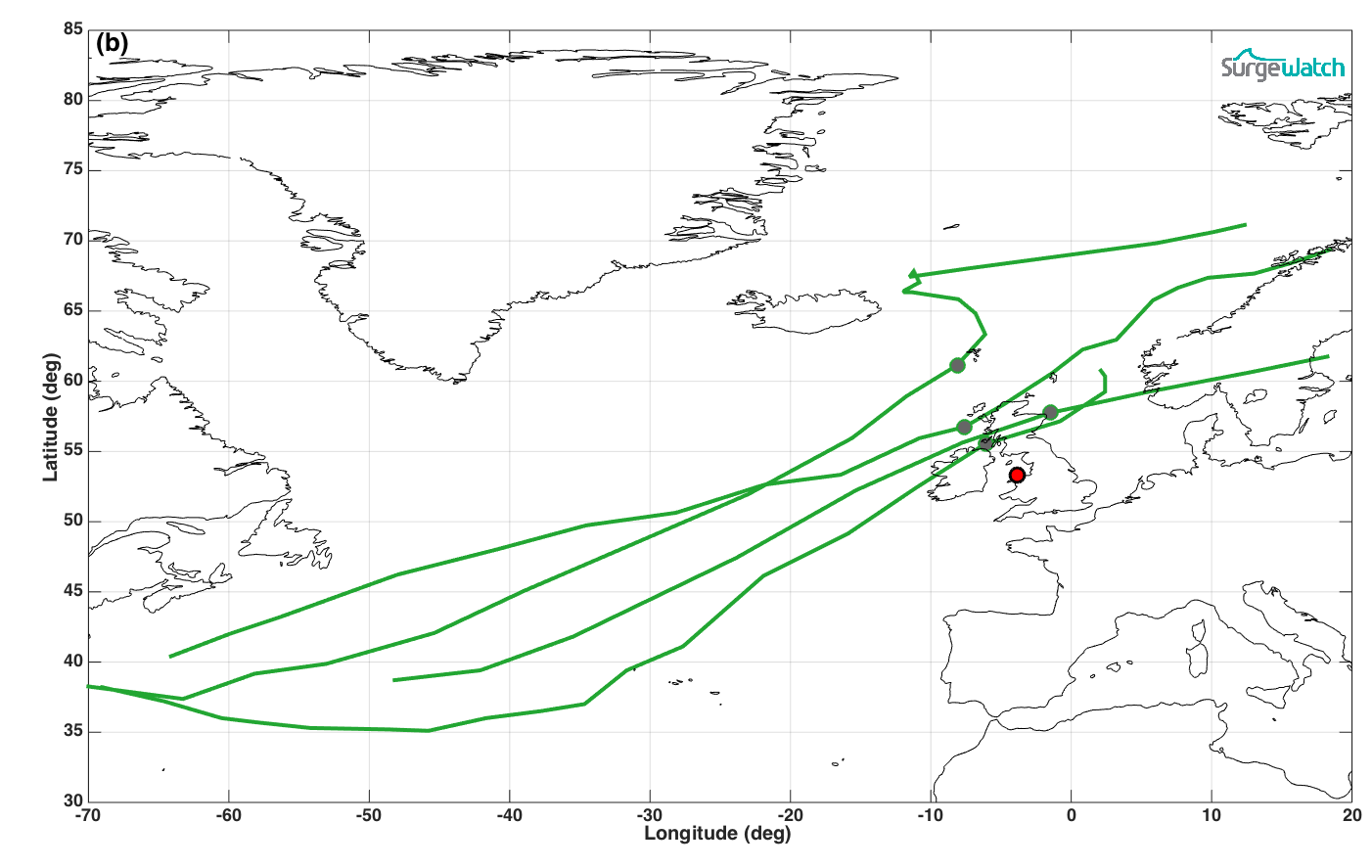** |
| --- |
| **Supplementary Figure B17:** Tracks of the storms that generated (a) high water levels and (b) skew surges that reached or exceeded the 1 in 5 year return level at Llandudno (location shown with the blue dot). The red dot indicates the location of the storm centre at the time of highest water level or skew surge. |


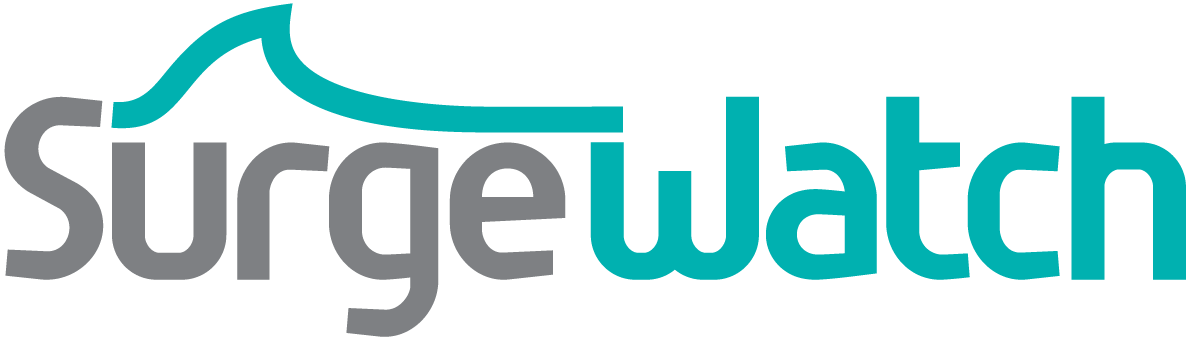
**LOCATION**

** B18.** **Liverpool**

*Chart Datum (CD) is 4.93m below Ordnance Datum Newlyn (ODN).*

| *Observational Period* |  | *Latitude & Longitude* |
| --- | --- | --- |
| ***1991-present*** |  | ***53° 26′ 58.9″ N, 03° 01′ 05.3″ W*** |

**Supplementary Table B18a:** High water levels (m CD) that reached or exceeded a 1 in 5 year return level at this site.

| **Date and time (GMT)** | **Return period (years)** | **Water**  **level (m CD)** | **Astronomical tide (m CD)** | **Skew surge (m)** |
| --- | --- | --- | --- | --- |
| 05/12/2013 12:30 | 43 | 11.15 | 9.96 | 1.17 |
| 10/02/1997 12:45 | 12 | 10.82 | 10.08 | 0.71 |
| 03/01/2014 12:00 | 8 | 10.79 | 10.21 | 0.57 |
| 01/02/2002 13:30 | 5 | 10.69 | 9.74 | 0.94 |

**Supplementary Table B18b:** Skew surges (m) that reached or exceeded a 1 in 5 year return level at this site.

| **Date and time (GMT)** | **Return period (years)** | **Skew surge (m)** | **Water**  **level (m CD)** | **Astronomical tide (m CD)** |
| --- | --- | --- | --- | --- |
| 08/01/2005 08:30 | 46 | 1.59 | 9.97 | 8.34 |
| 24/12/1997 20:00 | 15 | 1.36 | 8.84 | 7.26 |
| 03/12/1999 08:45 | 9 | 1.27 | 9.33 | 7.80 |
| 06/11/1996 07:30 | 6 | 1.19 | 8.63 | 7.44 |
| 13/01/2007 18:30 | 6 | 1.19 | 8.66 | 7.42 |
| 19/02/1997 21:45 | 5 | 1.18 | 9.39 | 8.21 |

| **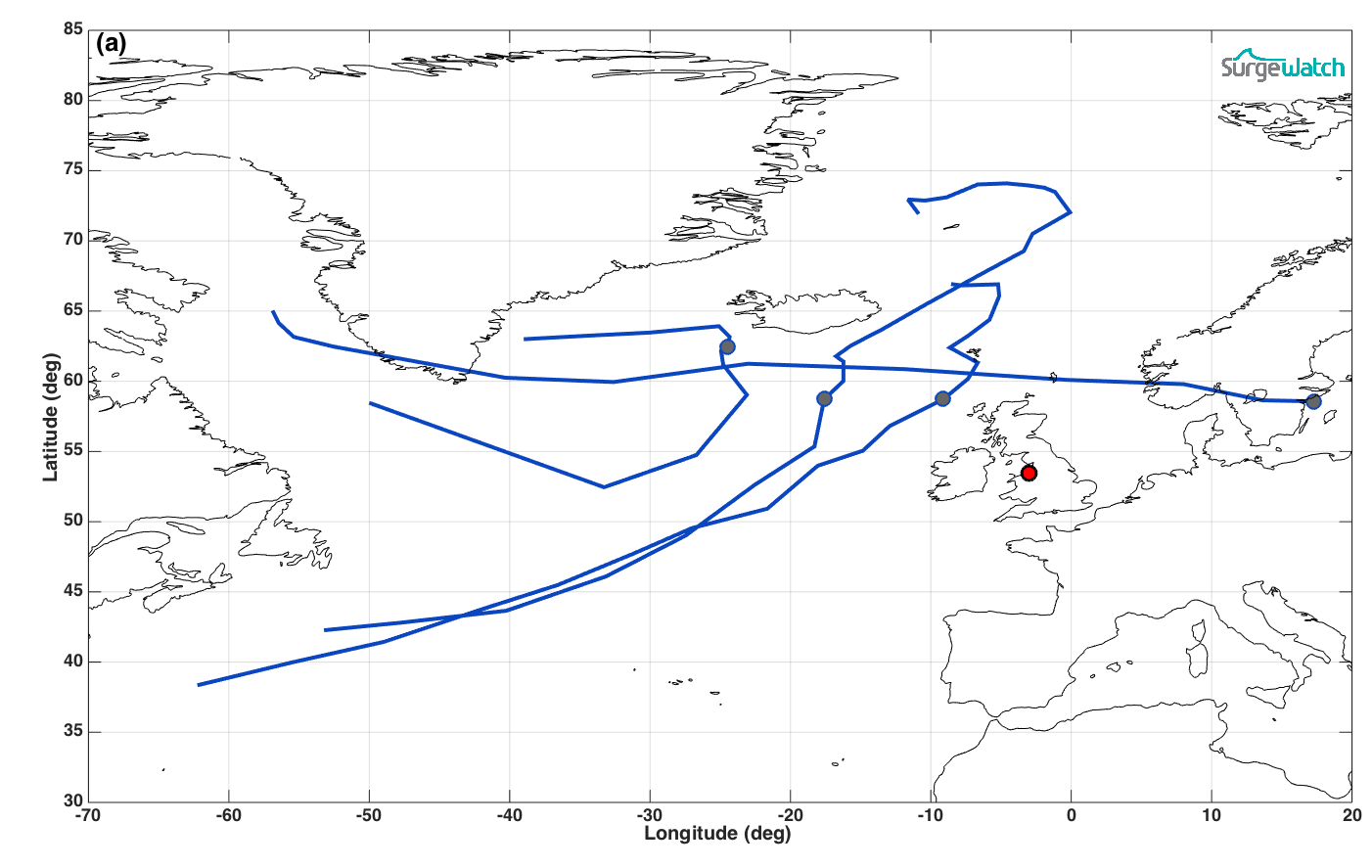**  **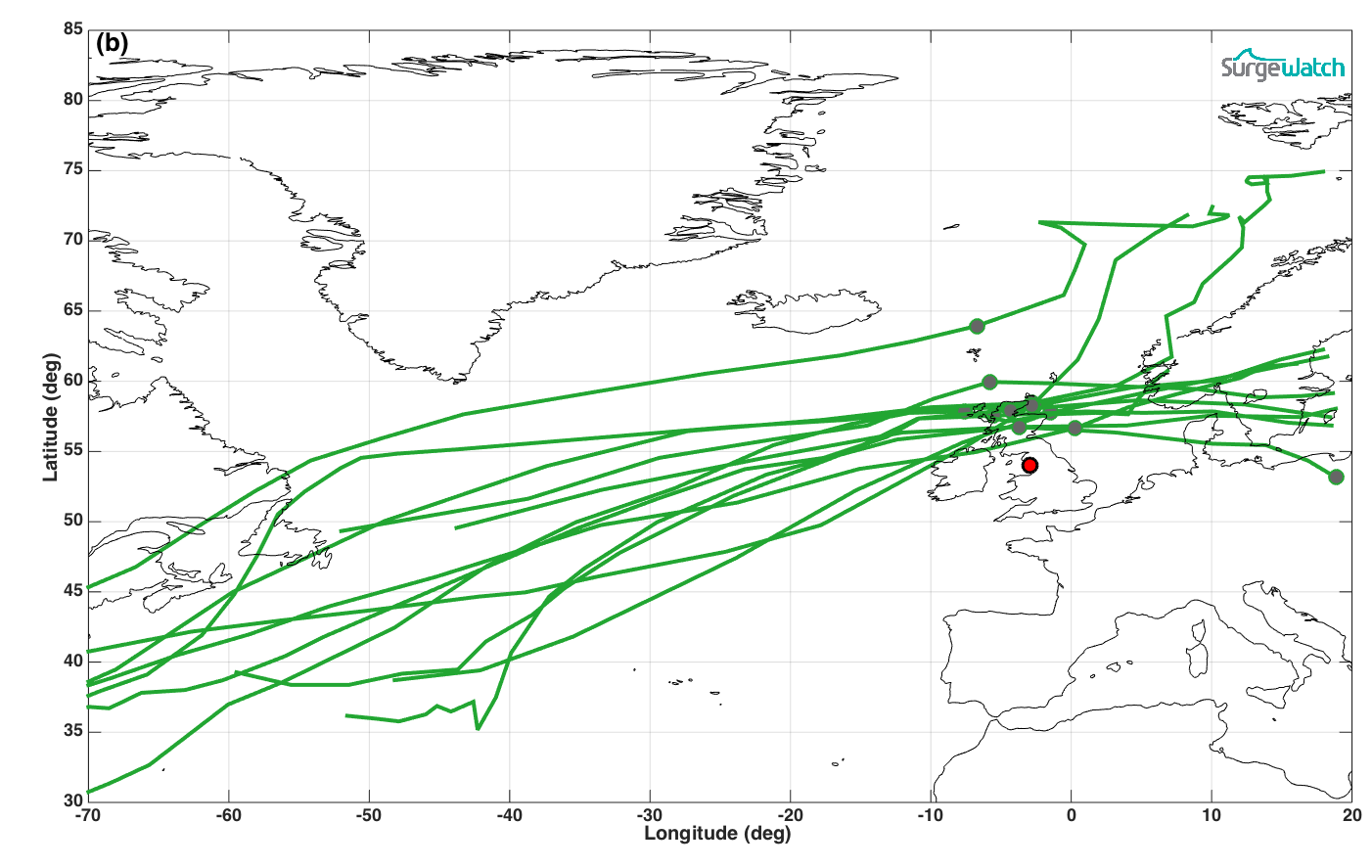** |
| --- |
| **Supplementary Figure B18:** Tracks of the storms that generated (a) high water levels and (b) skew surges that reached or exceeded the 1 in 5 year return level at Liverpool (location shown with the blue dot). The red dot indicates the location of the storm centre at the time of highest water level or skew surge. |


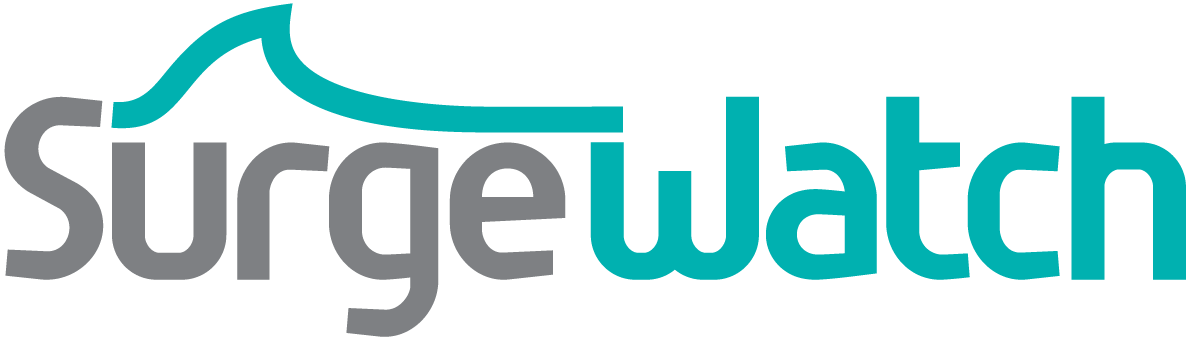
**LOCATION**

** B19.** **Heysham**

*Chart Datum (CD) is 4.90m below Ordnance Datum Newlyn (ODN).*

| *Observational Period* |  | *Latitude & Longitude* |
| --- | --- | --- |
| ***1954-1976, 2004-present*** |  | ***54° 01′ 54.6″ N, 02° 55′ 12.9″ W*** |

**Supplementary Table B19a:** High water levels (m CD) that reached or exceeded a 1 in 5 year return level at this site.

| **Date and time (GMT)** | **Return period (years)** | **Water**  **level (m CD)** | **Astronomical tide (m CD)** | **Skew surge (m)** |
| --- | --- | --- | --- | --- |
| 01/02/1983 01:00 | 100 | 11.56 | 9.82 | 1.74 |
| 01/02/2002 13:45 | 28 | 11.35 | 10.27 | 1.08 |
| 10/02/1997 13:15 | 19 | 11.29 | 10.45 | 0.84 |
| 03/01/2014 12:15 | 9 | 11.18 | 10.47 | 0.71 |

**Supplementary Table B19b:** Skew surges (m) that reached or exceeded a 1 in 5 year return level at this site.

| **Date and time (GMT)** | **Return period (years)** | **Skew surge (m)** | **Water**  **level (m CD)** | **Astronomical tide (m CD)** |
| --- | --- | --- | --- | --- |
| 01/02/1983 01:00 | 42 | 1.74 | 11.56 | 9.82 |
| 09/02/1988 15:00 | 20 | 1.58 | 10.15 | 8.57 |
| 13/01/1984 07:00 | 15 | 1.49 | 8.83 | 7.18 |
| 06/11/1996 07:45 | 12 | 1.45 | 8.98 | 7.53 |
| 03/01/1976 00:00 | 8 | 1.36 | 10.69 | 9.32 |
| 08/01/2005 08:45 | 8 | 1.35 | 10.00 | 8.62 |
| 02/01/1984 23:00 | 7 | 1.34 | 10.34 | 8.99 |
| 19/03/2004 10:15 | 7 | 1.33 | 10.77 | 9.43 |
| 24/01/1993 00:00 | 7 | 1.32 | 10.50 | 9.18 |
| 03/12/1999 08:15 | 6 | 1.31 | 9.53 | 8.19 |
| 08/12/1964 01:00 | 6 | 1.31 | 9.67 | 8.36 |
| 11/01/2007 04:00 | 6 | 1.29 | 9.07 | 7.80 |

| **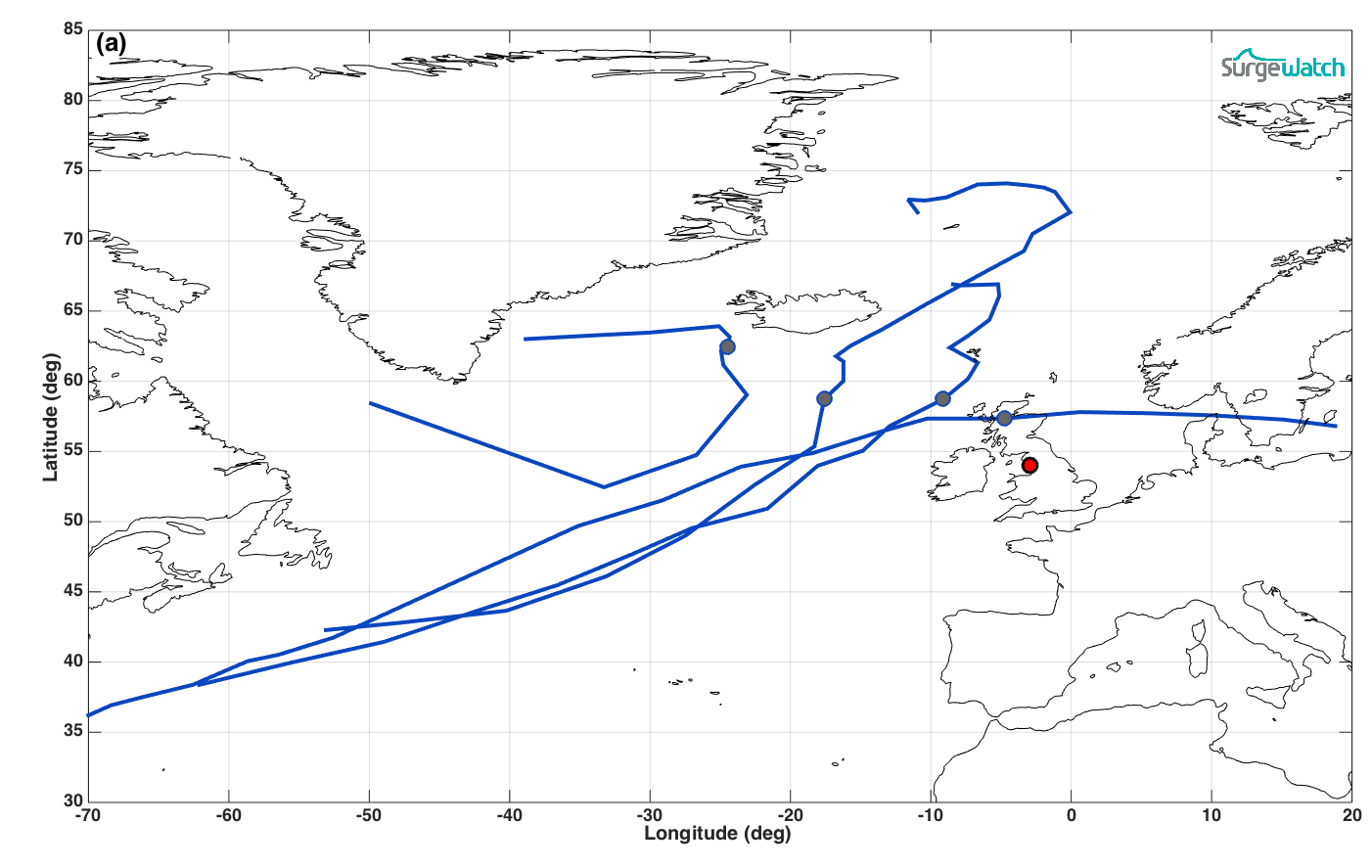**  **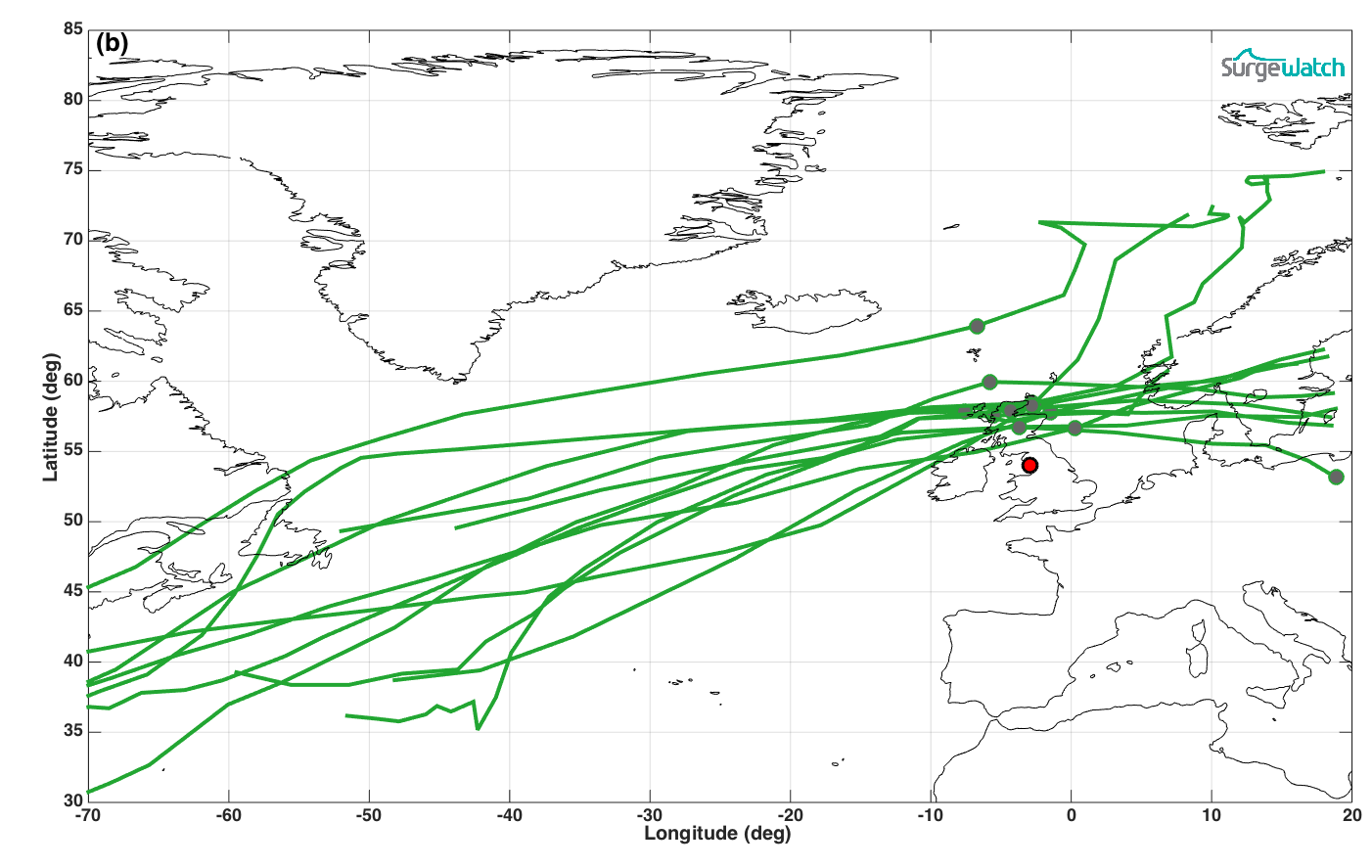** |
| --- |
| **Supplementary Figure B19:** Tracks of the storms that generated (a) high water levels and (b) skew surges that reached or exceeded the 1 in 5 year return level at Heysham (location shown with the blue dot). The red dot indicates the location of the storm centre at the time of highest water level or skew surge. |


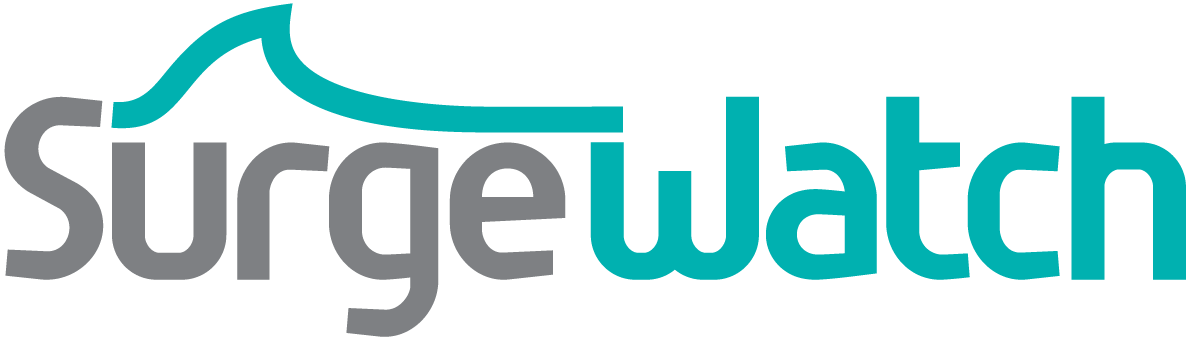
**LOCATION**

** B20.** **Workington**

*Chart Datum (CD) is 4.20m below Ordnance Datum Newlyn (ODN).*

| *Observational Period* |  | *Latitude & Longitude* |
| --- | --- | --- |
| ***1992-present*** |  | ***54° 39′ 02.6″ N, 03° 34′ 01.8″ W*** |

**Supplementary Table B20a:** High water levels (m CD) that reached or exceeded a 1 in 5 year return level at this site.

| **Date and time (GMT)** | **Return period (years)** | **Water**  **level (m CD)** | **Astronomical tide (m CD)** | **Skew surge (m)** |
| --- | --- | --- | --- | --- |
| 10/02/1997 13:15 | 86 | 9.96 | 9.21 | 0.75 |
| 03/01/2014 12:30 | 46 | 9.90 | 9.06 | 0.84 |
| 01/02/2014 12:15 | 20 | 9.77 | 9.16 | 0.62 |
| 01/02/2002 13:45 | 17 | 9.72 | 8.95 | 0.79 |
| 10/01/1993 12:30 | 16 | 9.69 | 9.03 | 0.67 |
| 11/01/1993 13:15 | 15 | 9.69 | 9.10 | 0.59 |
| 12/01/1993 14:15 | 12 | 9.65 | 8.98 | 0.65 |
| 31/08/1992 01:00 | 8 | 9.59 | 9.22 | 0.38 |
| 30/08/1992 00:00 | 5 | 9.53 | 9.22 | 0.31 |

**Supplementary Table B20b:** Skew surges (m) that reached or exceeded a 1 in 5 year return level at this site.

| **Date and time (GMT)** | **Return period (years)** | **Skew surge (m)** | **Water**  **level (m CD)** | **Astronomical tide (m CD)** |
| --- | --- | --- | --- | --- |
| 27/12/2013 06:00 | 31 | 1.41 | 8.14 | 6.74 |
| 24/12/1997 19:45 | 31 | 1.41 | 8.06 | 6.64 |
| 24/01/1993 00:15 | 12 | 1.24 | 9.32 | 8.08 |
| 06/11/1996 08:00 | 9 | 1.20 | 7.76 | 6.56 |
| 03/01/2012 07:15 | 6 | 1.12 | 7.38 | 6.12 |

| **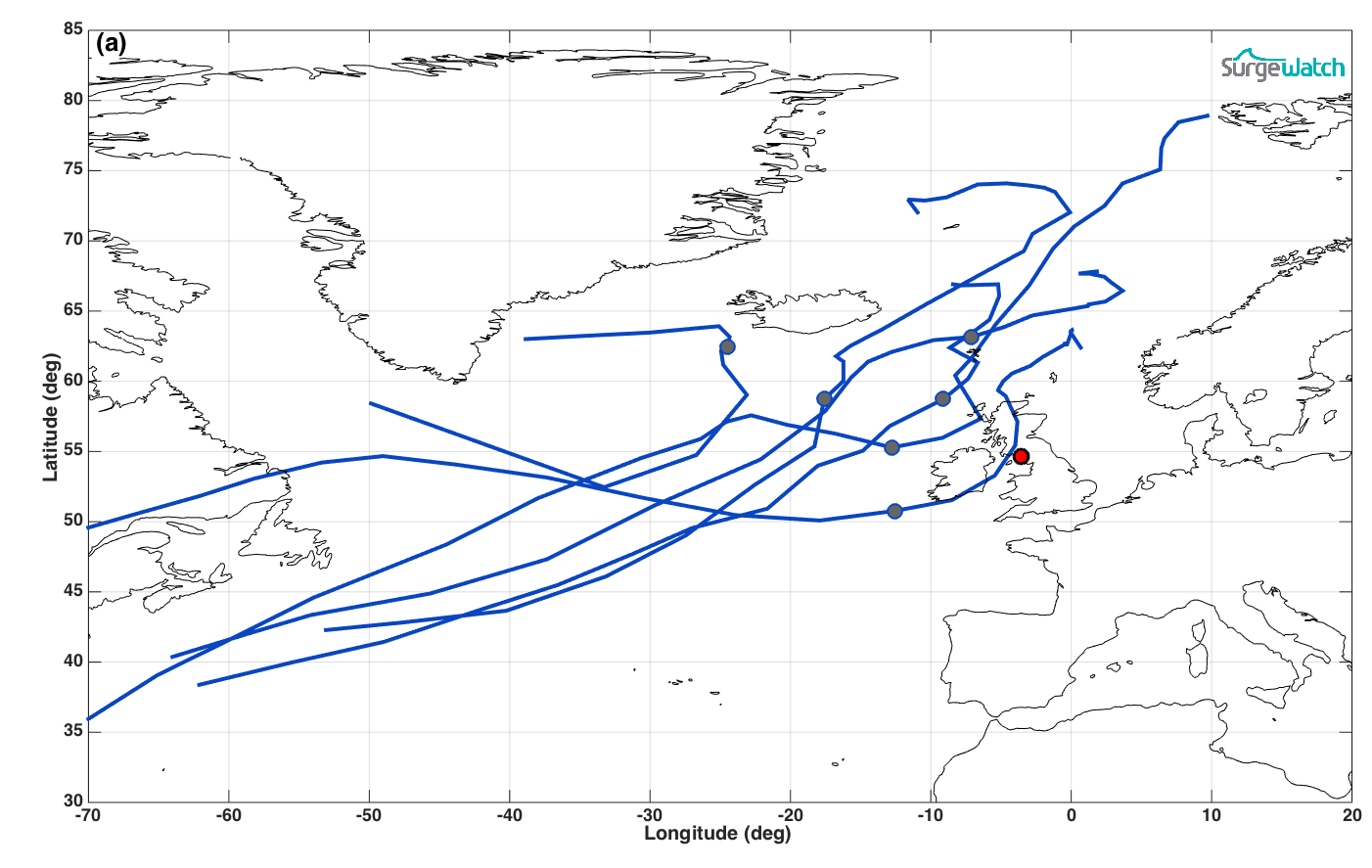**  **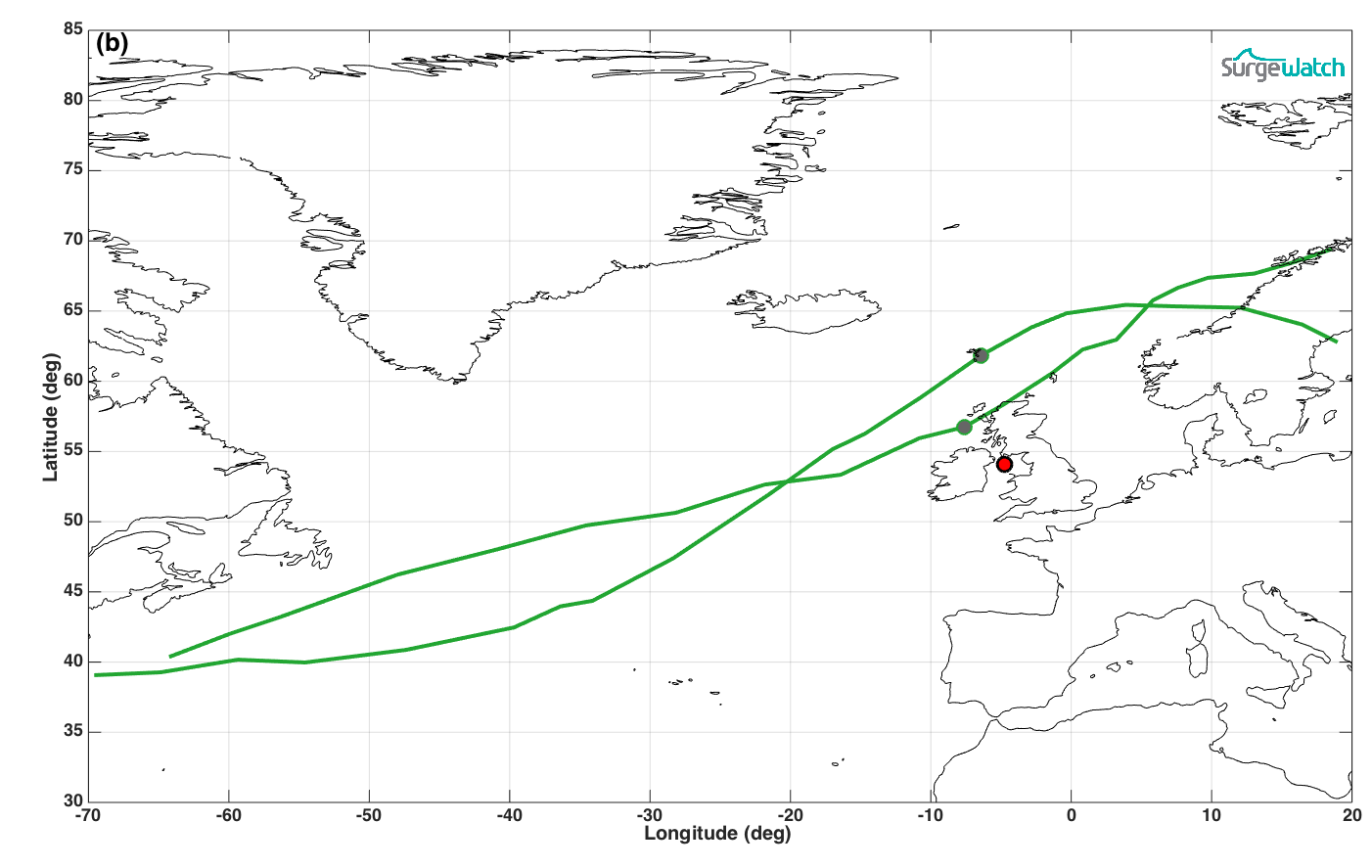** |
| --- |
| **Supplementary Figure B20:** Tracks of the storms that generated (a) high water levels and (b) skew surges that reached or exceeded the 1 in 5 year return level at Workington (location shown with the blue dot). The red dot indicates the location of the storm centre at the time of highest water level or skew surge. |


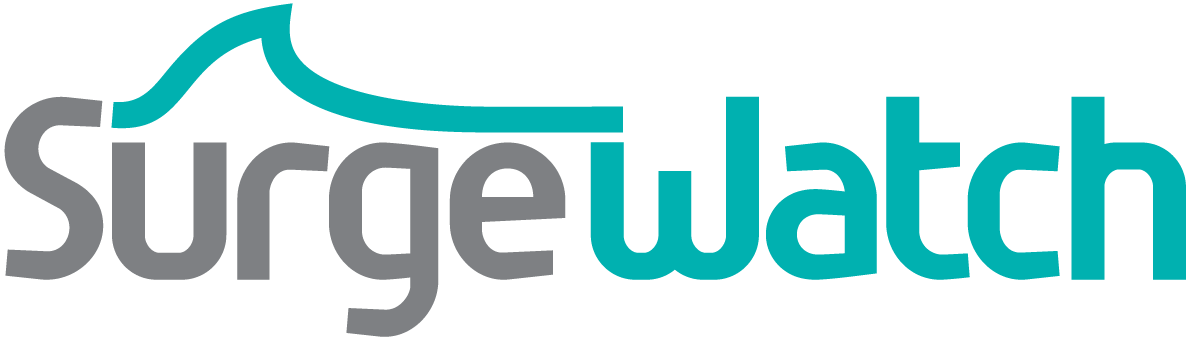
**LOCATION**

** B21.** **Port Erin**

*Chart Datum (CD) is 2.81m below Ordnance Datum Newlyn (ODN).*

| *Observational Period* |  | *Latitude & Longitude* |
| --- | --- | --- |
| ***1992-1995, 1998-present*** |  | ***54° 05′ 06.8″ N, 04° 46′ 05.0″ W*** |

**Supplementary Table B21a:** High water levels (m CD) that reached or exceeded a 1 in 5 year return level at this site.

| **Date and time (GMT)** | **Return period (years)** | **Water**  **level (m CD)** | **Astronomical tide (m CD)** | **Skew surge (m)** |
| --- | --- | --- | --- | --- |
| 03/01/2014 12:30 | 68 | 6.62 | 5.82 | 0.79 |
| 01/02/2002 13:45 | 33 | 6.52 | 5.73 | 0.79 |
| 01/02/2014 12:15 | 7 | 6.37 | 5.88 | 0.48 |
| 10/01/1993 12:30 | 6 | 6.31 | 5.71 | 0.59 |
| 03/02/2014 13:30 | 5 | 6.35 | 5.78 | 0.57 |

**Supplementary Table B21b:** Skew surges (m) that reached or exceeded a 1 in 5 year return level at this site.

| **Date and time (GMT)** | **Return period (years)** | **Skew surge (m)** | **Water**  **level (m CD)** | **Astronomical tide (m CD)** |
| --- | --- | --- | --- | --- |
| 27/12/2013 05:45 | 34 | 1.03 | 5.40 | 4.36 |
| 17/01/1993 06:15 | 6 | 0.87 | 5.39 | 4.51 |

| **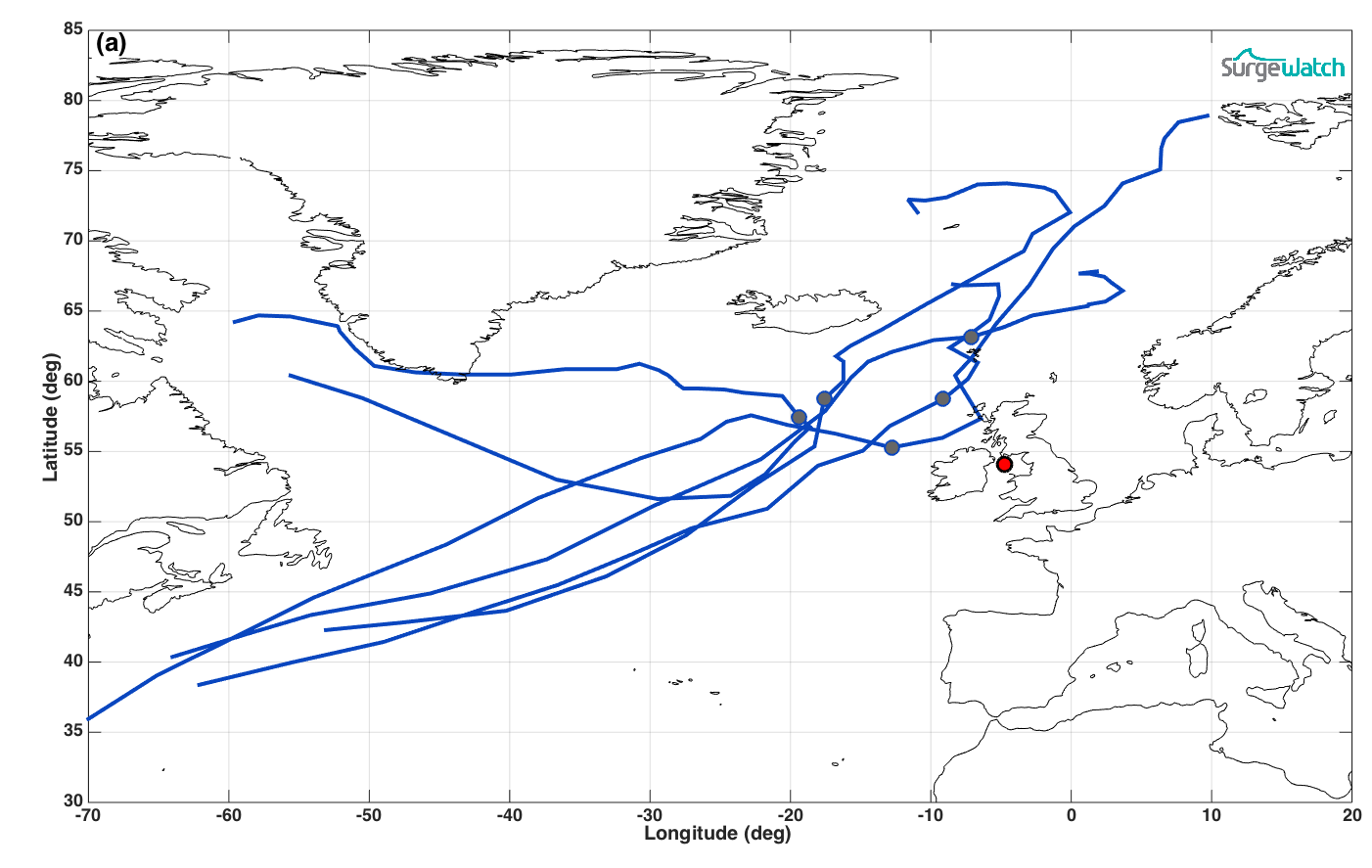**  **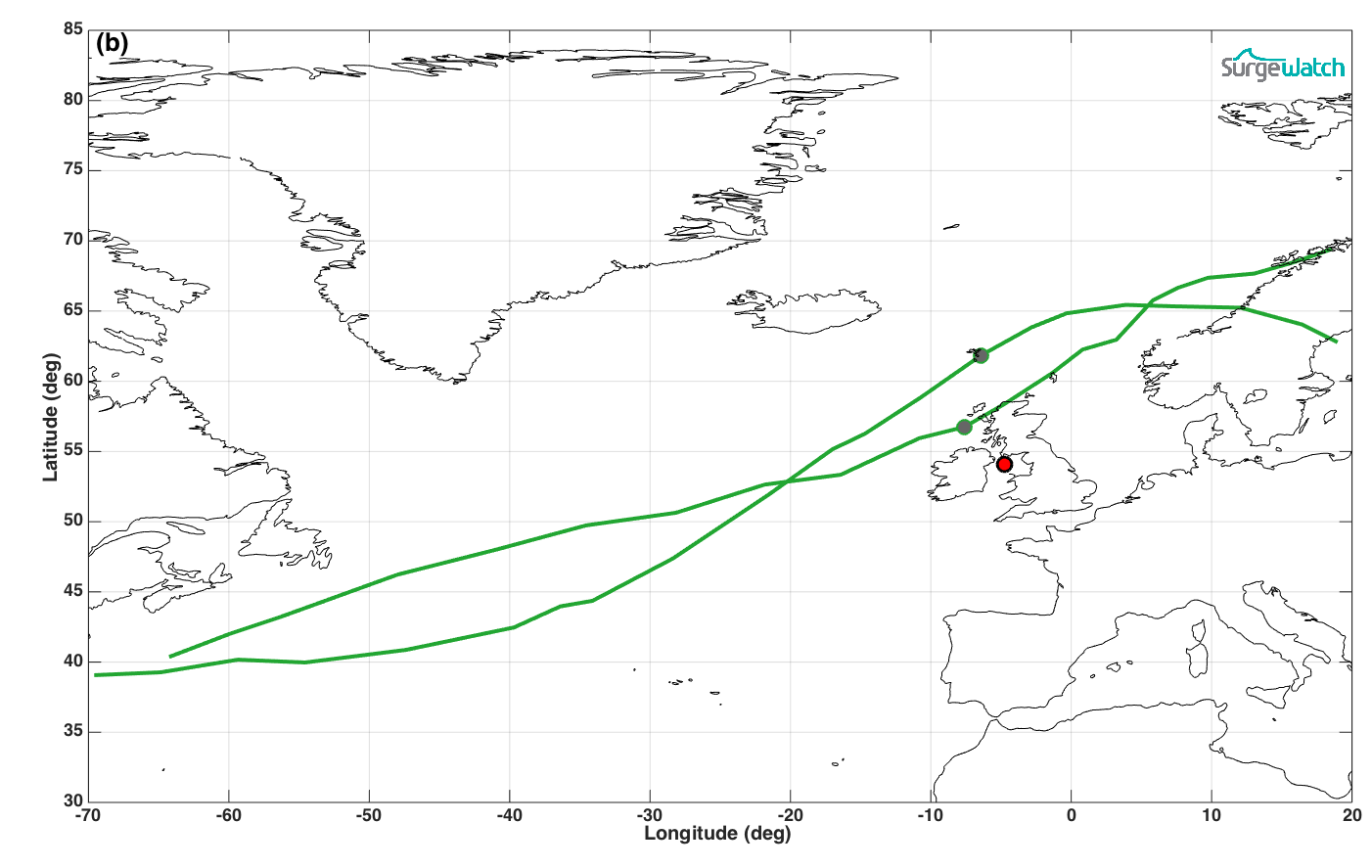** |
| --- |
| **Supplementary Figure B21:** Tracks of the storms that generated (a) high water levels and (b) skew surges that reached or exceeded the 1 in 5 year return level at Port Erin (location shown with the blue dot). The red dot indicates the location of the storm centre at the time of highest water level or skew surge. |


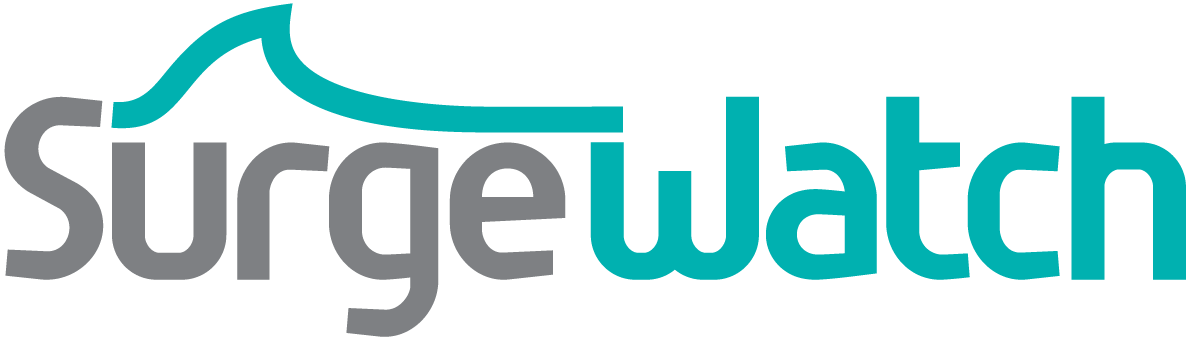
**LOCATION**

** B22.** **Portpatrick**

*Chart Datum (CD) is 1.80m below Ordnance Datum Newlyn (ODN).*

| *Observational Period* |  | *Latitude & Longitude* |
| --- | --- | --- |
| ***1968-present*** |  | ***54° 50′ 33.2″ N, 05° 07′ 12.1″ W*** |

**Supplementary Table B22a:** High water levels (m CD) that reached or exceeded a 1 in 5 year return level at this site.

| **Date and time (GMT)** | **Return period (years)** | **Water**  **level (m CD)** | **Astronomical tide (m CD)** | **Skew surge (m)** |
| --- | --- | --- | --- | --- |
| 03/01/2014 12:30 | 244 | 5.28 | 4.38 | 0.89 |
| 05/01/1991 15:00 | 134 | 5.16 | 4.06 | 1.10 |
| 01/02/2002 14:00 | 113 | 5.17 | 4.41 | 0.76 |
| 10/01/1993 12:45 | 24 | 4.98 | 4.23 | 0.76 |
| 06/01/2014 15:00 | 18 | 4.99 | 4.21 | 0.77 |
| 01/02/2014 12:30 | 15 | 4.96 | 4.43 | 0.53 |
| 03/01/1998 14:45 | 10 | 4.89 | 4.12 | 0.77 |
| 03/02/2014 13:45 | 8 | 4.89 | 4.38 | 0.49 |
| 04/01/1999 13:15 | 8 | 4.86 | 4.25 | 0.60 |
| 10/02/1997 13:30 | 8 | 4.85 | 4.29 | 0.57 |
| 13/12/1981 13:00 | 8 | 4.82 | 4.14 | 0.69 |
| 22/12/1968 13:00 | 7 | 4.77 | 4.16 | 0.61 |
| 01/02/1988 11:00 | 6 | 4.80 | 3.67 | 1.13 |
| 24/10/1995 23:15 | 6 | 4.81 | 4.06 | 0.74 |

**Supplementary Table B22b:** Skew surges (m) that reached or exceeded a 1 in 5 year return level at this site.

| **Date and time (GMT)** | **Return period (years)** | **Skew surge (m)** | **Water**  **level (m CD)** | **Astronomical tide (m CD)** |
| --- | --- | --- | --- | --- |
| 27/12/2013 06:00 | 45 | 1.19 | 4.62 | 3.42 |
| 01/02/1988 11:00 | 26 | 1.13 | 4.80 | 3.67 |
| 05/01/1991 15:00 | 19 | 1.10 | 5.16 | 4.06 |
| 17/01/1993 06:15 | 14 | 1.06 | 4.43 | 3.33 |
| 09/02/1988 15:00 | 13 | 1.05 | 4.65 | 3.60 |
| 24/12/1997 19:30 | 13 | 1.05 | 4.36 | 3.31 |
| 24/01/1993 00:45 | 12 | 1.04 | 4.65 | 3.59 |
| 13/01/1984 07:00 | 8 | 1.00 | 4.05 | 3.07 |
| 18/01/1995 00:00 | 8 | 0.99 | 4.77 | 3.75 |
| 03/01/2012 08:00 | 8 | 0.99 | 4.12 | 2.95 |
| 03/03/1982 04:00 | 6 | 0.97 | 4.34 | 3.28 |
| 12/11/1991 15:00 | 6 | 0.97 | 4.52 | 3.57 |

| **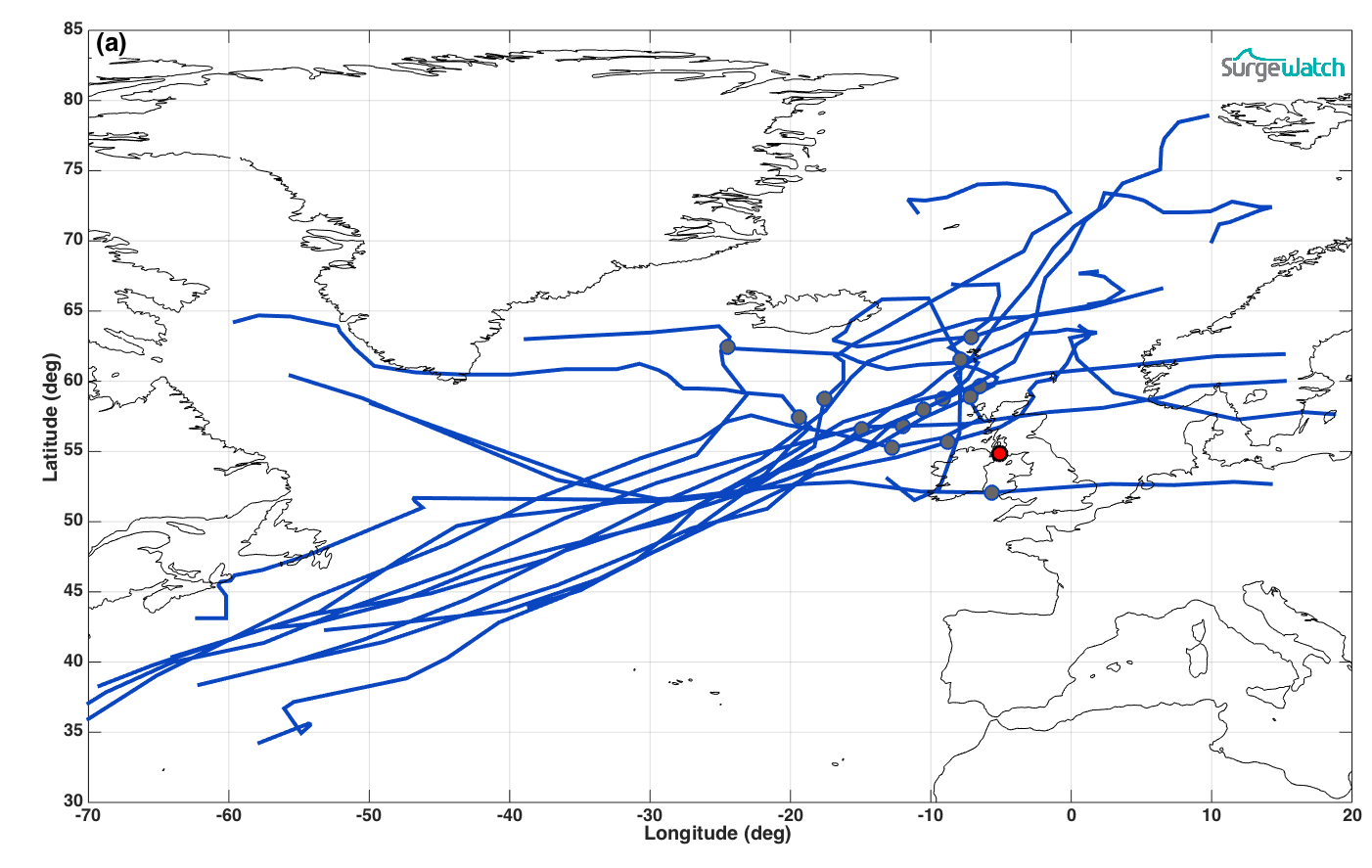**  **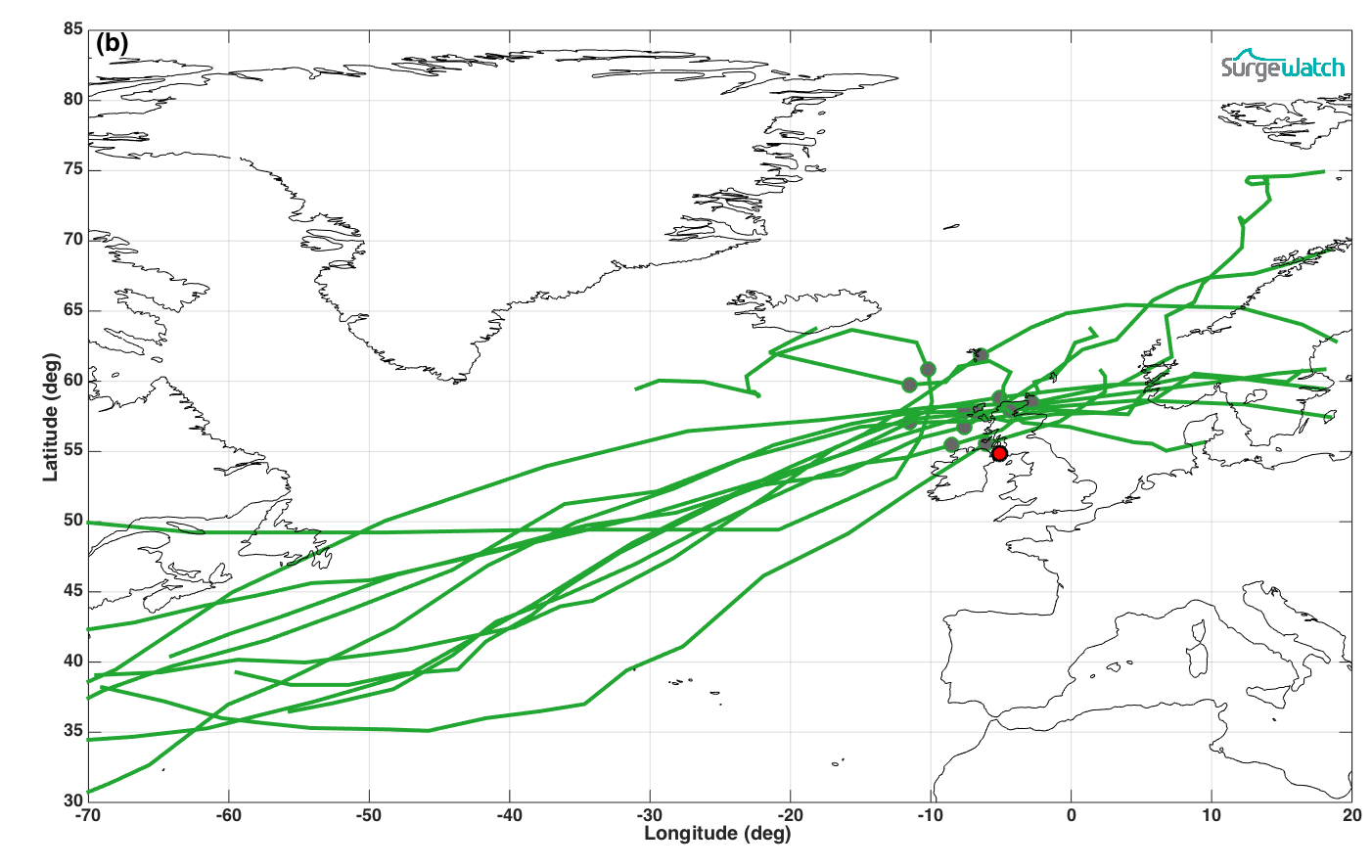** |
| --- |
| **Supplementary Figure B22:** Tracks of the storms that generated (a) high water levels and (b) skew surges that reached or exceeded the 1 in 5 year return level at Portpatrick (location shown with the blue dot). The red dot indicates the location of the storm centre at the time of highest water level or skew surge. |


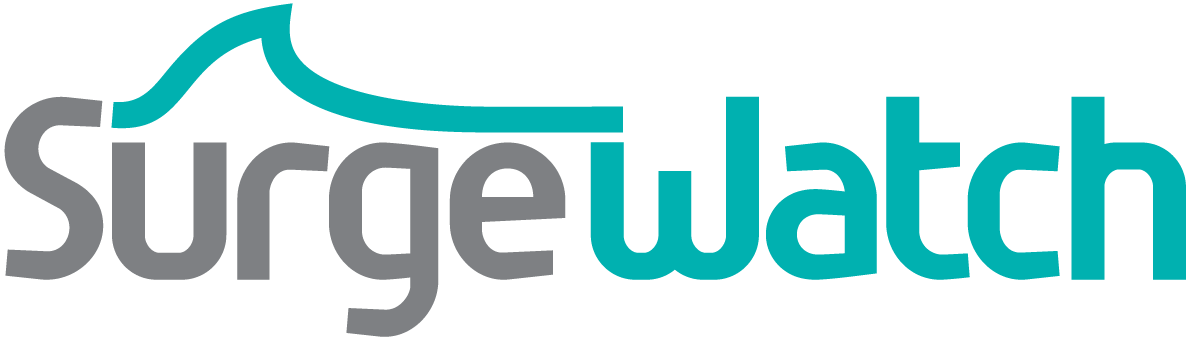
**LOCATION**

** B23.** **Millport**

*Chart Datum (CD) is 1.62m below Ordnance Datum Newlyn (ODN).*

| *Observational Period* |  | *Latitude & Longitude* |
| --- | --- | --- |
| ***1978, 1981-1983, 1985-present*** |  | ***55° 44′ 59.3″ N, 04° 54′ 22.8″ W*** |

**Supplementary Table B23a:** High water levels (m CD) that reached or exceeded a 1 in 5 year return level at this site.

| **Date and time (GMT)** | **Return period (years)** | **Water**  **level (m CD)** | **Astronomical tide (m CD)** | **Skew surge (m)** |
| --- | --- | --- | --- | --- |
| 05/01/1991 15:00 | 81 | 5.07 | 3.76 | 1.31 |
| 03/01/2014 13:15 | 18 | 4.80 | 3.71 | 1.07 |
| 10/01/1993 13:00 | 16 | 4.74 | 3.68 | 1.03 |
| 09/02/1988 16:00 | 12 | 4.67 | 3.32 | 1.35 |
| 11/11/2010 15:15 | 9 | 4.65 | 3.44 | 1.21 |
| 01/02/2002 15:00 | 8 | 4.63 | 3.93 | 0.67 |
| 04/01/1999 13:30 | 8 | 4.62 | 3.81 | 0.81 |
| 01/02/1988 11:00 | 8 | 4.60 | 3.26 | 1.33 |
| 18/01/1995 01:15 | 7 | 4.59 | 3.45 | 1.14 |

**Supplementary Table B23b:** Skew surges (m) that reached or exceeded a 1 in 5 year return level at this site.

| **Date and time (GMT)** | **Return period (years)** | **Skew surge (m)** | **Water**  **level (m CD)** | **Astronomical tide (m CD)** |
| --- | --- | --- | --- | --- |
| 06/11/1996 07:15 | 72 | 1.60 | 4.15 | 2.40 |
| 03/01/2012 08:15 | 29 | 1.46 | 4.32 | 2.67 |
| 09/02/1988 16:00 | 15 | 1.35 | 4.67 | 3.32 |
| 01/02/1988 11:00 | 13 | 1.33 | 4.60 | 3.26 |
| 27/12/2013 06:45 | 11 | 1.31 | 4.28 | 2.97 |
| 05/01/1991 15:00 | 11 | 1.31 | 5.07 | 3.76 |
| 24/01/1993 01:30 | 11 | 1.30 | 4.46 | 3.14 |
| 03/03/1982 05:00 | 9 | 1.28 | 4.39 | 3.11 |
| 11/11/2010 15:15 | 6 | 1.21 | 4.65 | 3.44 |

| **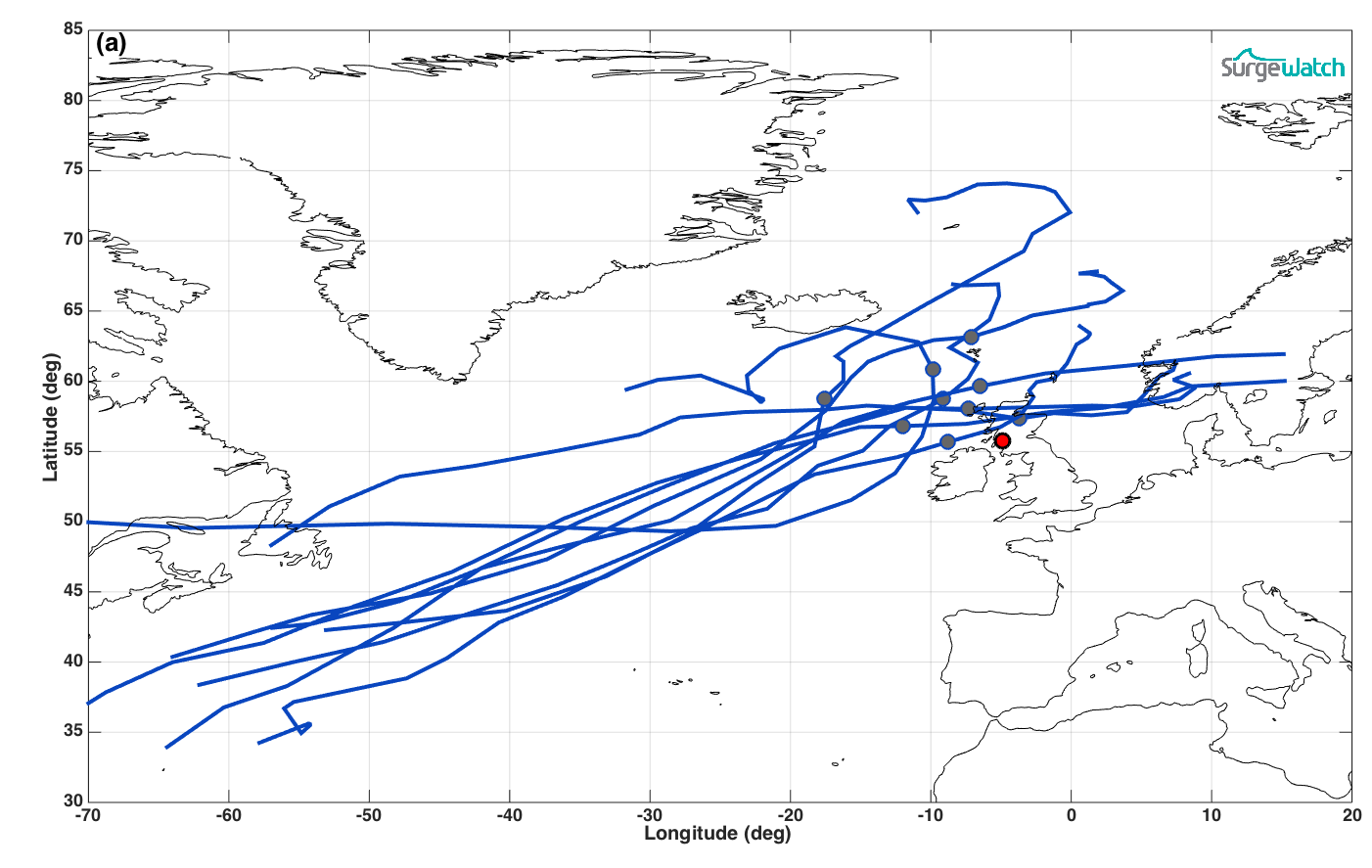**  **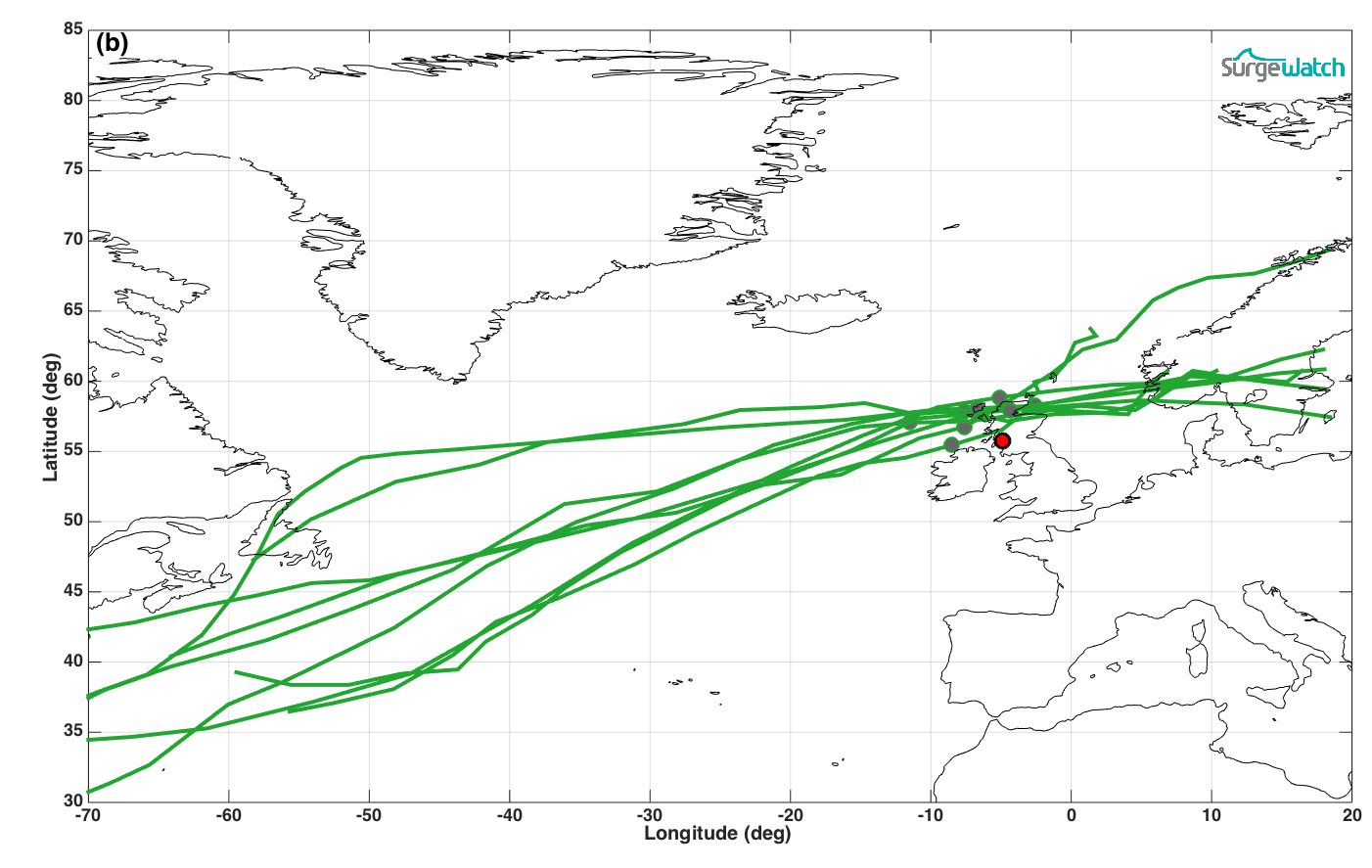** |
| --- |
| **Supplementary Figure B23:** Tracks of the storms that generated (a) high water levels and (b) skew surges that reached or exceeded the 1 in 5 year return level at Millport (location shown with the blue dot). The red dot indicates the location of the storm centre at the time of highest water level or skew surge. |


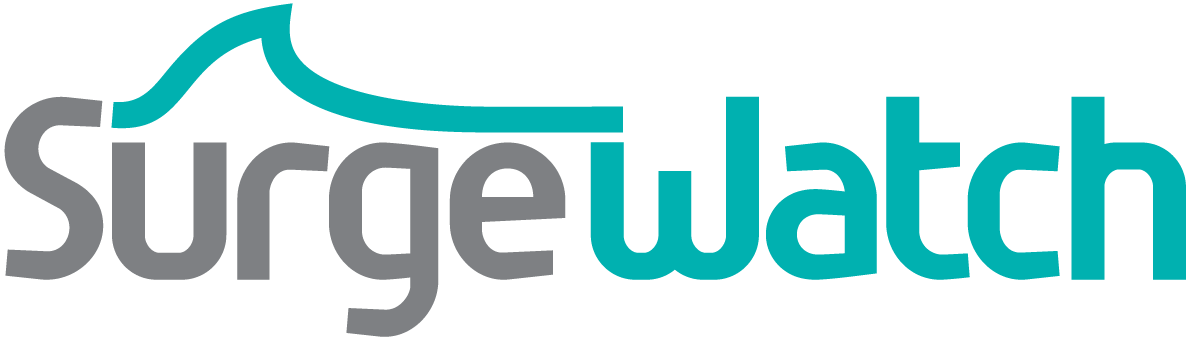
**LOCATION**

** B24.** **Port Ellen**

*Chart Datum (CD) is 0.19m below Ordnance Datum Newlyn (ODN).*

| *Observational Period* |  | *Latitude & Longitude* |
| --- | --- | --- |
| ***1979-1980, 1991-present*** |  | ***55° 37′ 39.3″ N, 06° 11′ 23.7″ W*** |

**Supplementary Table B24a:** High water levels (m CD) that reached or exceeded a 1 in 5 year return level at this site.

| **Date and time (GMT)** | **Return period (years)** | **Water**  **level (m CD)** | **Astronomical tide (m CD)** | **Skew surge (m)** |
| --- | --- | --- | --- | --- |
| 11/11/2010 16:45 | 8 | 1.99 | 0.89 | 1.10 |
| 26/12/1998 20:00 | 7 | 1.96 | 0.80 | 1.14 |
| 18/01/1995 03:00 | 7 | 1.95 | 0.88 | 1.00 |

**Supplementary Table B24b:** Skew surges (m) that reached or exceeded a 1 in 5 year return level at this site.

| **Date and time (GMT)** | **Return period (years)** | **Skew surge (m)** | **Water**  **level (m CD)** | **Astronomical tide (m CD)** |
| --- | --- | --- | --- | --- |
| 26/12/1998 20:00 | 20 | 1.14 | 1.96 | 0.80 |
| 11/01/2005 18:45 | 15 | 1.11 | 1.81 | 0.54 |
| 11/11/2010 16:45 | 14 | 1.10 | 1.99 | 0.89 |
| 18/01/1995 03:00 | 6 | 1.00 | 1.95 | 0.88 |

| **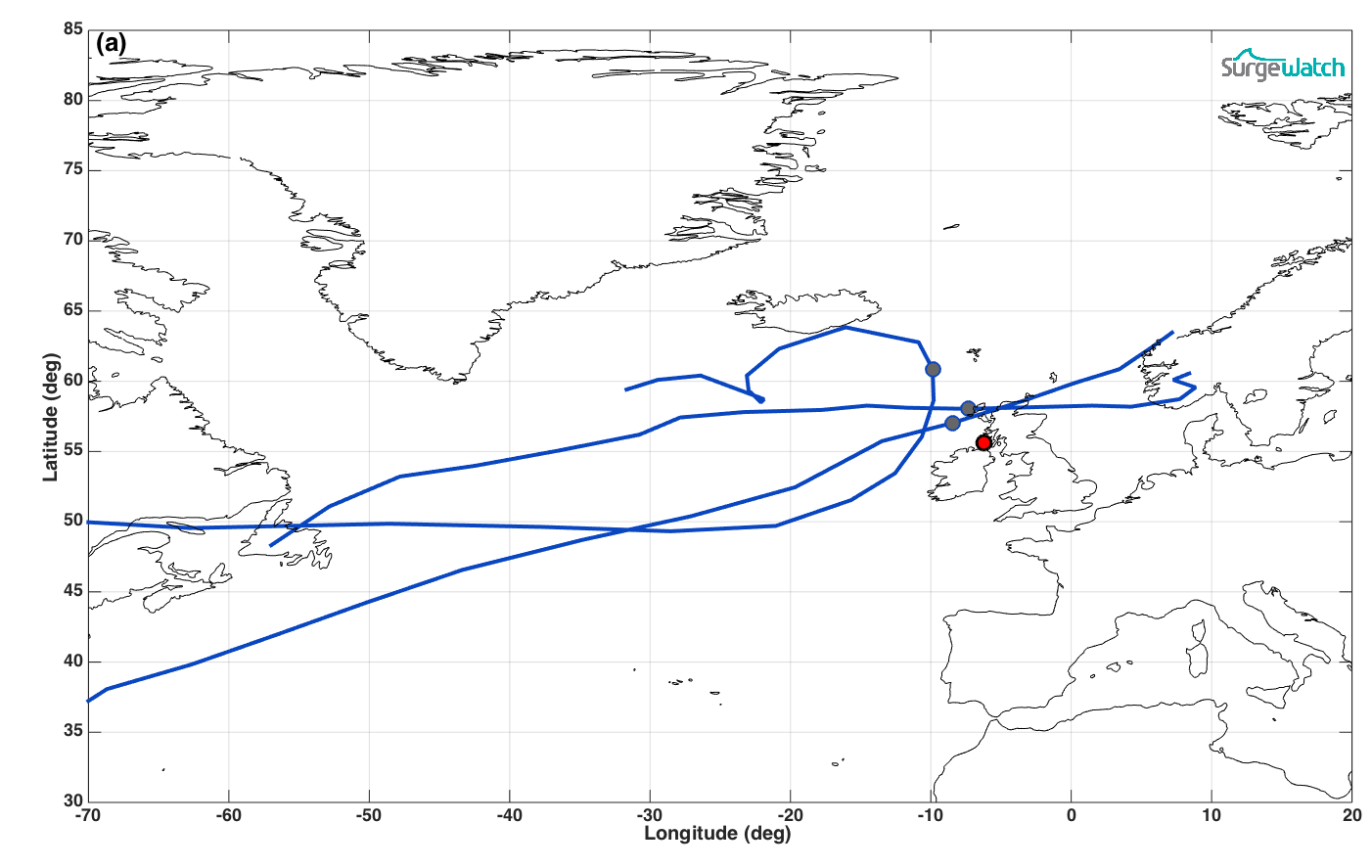**  **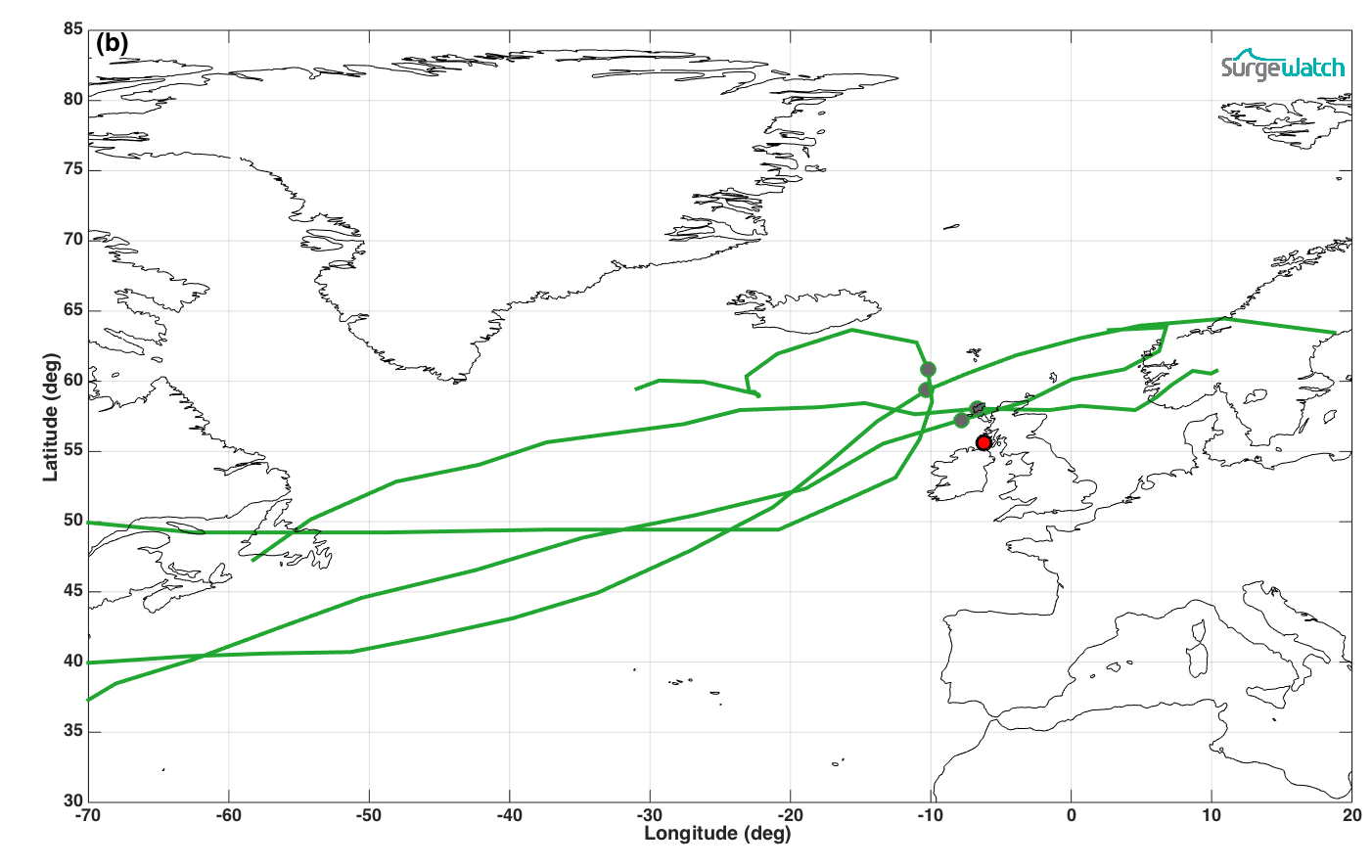** |
| --- |
| **Supplementary Figure B24:** Tracks of the storms that generated (a) high water levels and (b) skew surges that reached or exceeded the 1 in 5 year return level at Port Ellen (location shown with the blue dot). The red dot indicates the location of the storm centre at the time of highest water level or skew surge. |


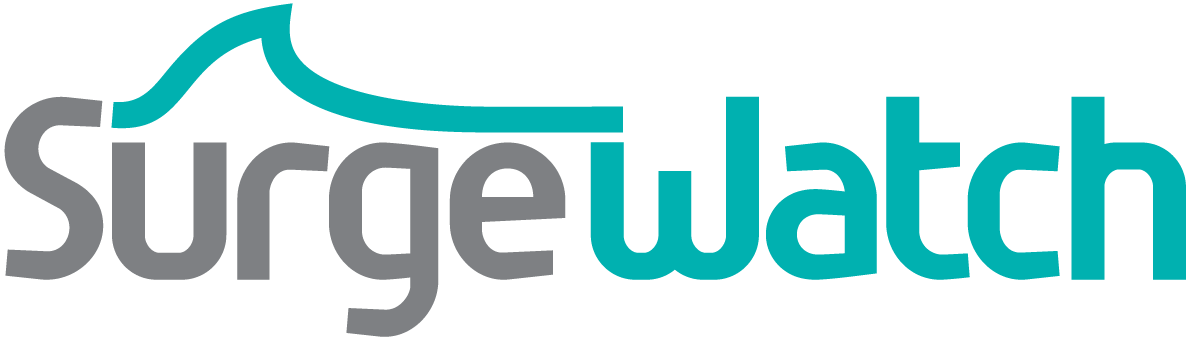
**LOCATION**

** B25.** **Tobermory**

*Chart Datum (CD) is 2.39m below Ordnance Datum Newlyn (ODN).*

| *Observational Period* |  | *Latitude & Longitude* |
| --- | --- | --- |
| ***1987-present*** |  | ***56° 37′ 23.2″ N, 06° 03′ 51.2″ W*** |

**Supplementary Table B25a:** High water levels (m CD) that reached or exceeded a 1 in 5 year return level at this site.

| **Date and time (GMT)** | **Return period (years)** | **Water**  **level (m CD)** | **Astronomical tide (m CD)** | **Skew surge (m)** |
| --- | --- | --- | --- | --- |
| 11/01/2005 19:00 | 71 | 6.06 | 4.54 | 1.51 |
| 03/01/2014 07:15 | 8 | 5.73 | 5.05 | 0.65 |

**Supplementary Table B25b:** Skew surges (m) that reached or exceeded a 1 in 5 year return level at this site.

| **Date and time (GMT)** | **Return period (years)** | **Skew surge (m)** | **Water**  **level (m CD)** | **Astronomical tide (m CD)** |
| --- | --- | --- | --- | --- |
| 11/01/2005 19:00 | 100 | 1.51 | 6.06 | 4.54 |
| 17/01/2009 21:45 | 9 | 1.11 | 5.01 | 3.85 |
| 11/11/2005 14:30 | 6 | 1.06 | 5.17 | 4.11 |
| 06/11/1996 03:15 | 6 | 1.05 | 4.57 | 3.47 |
| 27/12/2013 13:00 | 5 | 1.04 | 4.76 | 3.72 |

| **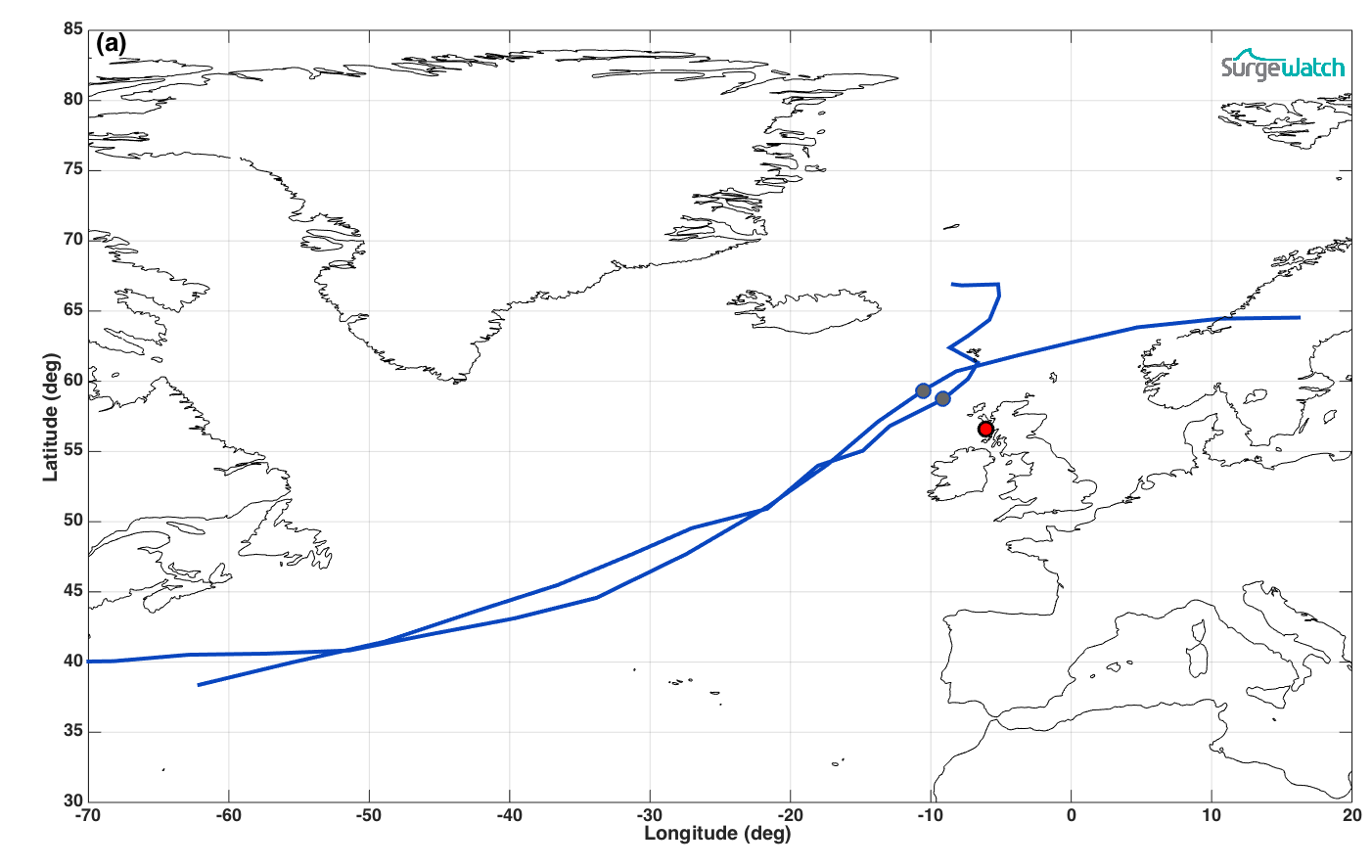**  **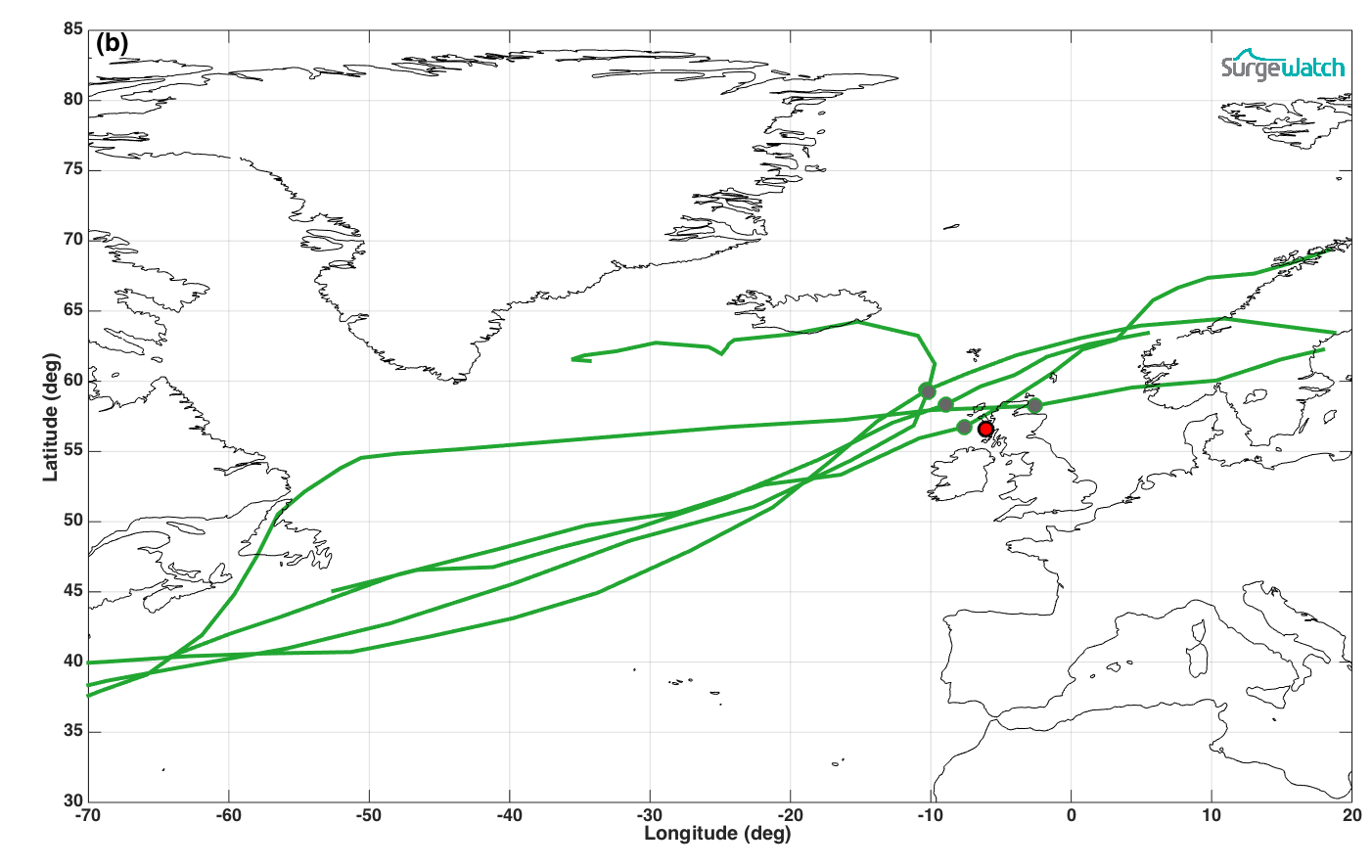** |
| --- |
| **Supplementary Figure B25:** Tracks of the storms that generated (a) high water levels and (b) skew surges that reached or exceeded the 1 in 5 year return level at Tobermory (location shown with the blue dot). The red dot indicates the location of the storm centre at the time of highest water level or skew surge. |


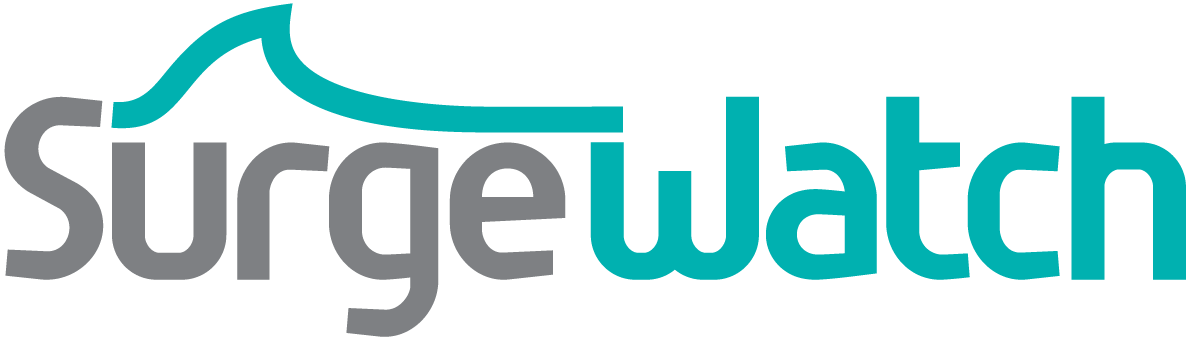
**LOCATION**

** B26.** **Stornoway**

*Chart Datum (CD) is 2.71m below Ordnance Datum Newlyn (ODN).*

| *Observational Period* |  | *Latitude & Longitude* |
| --- | --- | --- |
| ***1976, 1978-1981, 1983, 1985-present*** |  | ***58° 12′ 27.8″ N, 06° 23′ 20.0″ W*** |

**Supplementary Table B26a:** High water levels (m CD) that reached or exceeded a 1 in 5 year return level at this site.

| **Date and time (GMT)** | **Return period (years)** | **Water**  **level (m CD)** | **Astronomical tide (m CD)** | **Skew surge (m)** |
| --- | --- | --- | --- | --- |
| 01/02/2014 07:30 | 35 | 5.97 | 5.49 | 0.48 |
| 10/02/1997 08:30 | 20 | 5.89 | 5.49 | 0.41 |
| 12/01/2005 08:00 | 17 | 5.89 | 5.20 | 0.70 |
| 11/01/1993 08:15 | 15 | 5.85 | 5.29 | 0.54 |
| 03/01/2014 08:15 | 14 | 5.89 | 5.40 | 0.44 |
| 02/02/2014 08:30 | 10 | 5.86 | 5.46 | 0.39 |
| 09/02/1997 08:00 | 9 | 5.82 | 5.55 | 0.26 |
| 08/03/1981 08:00 | 8 | 5.77 | 5.37 | 0.40 |
| 10/01/1993 20:15 | 7 | 5.78 | 4.99 | 0.78 |
| 09/03/1989 08:00 | 7 | 5.77 | 5.62 | 0.15 |
| 16/09/1978 19:00 | 6 | 5.74 | 5.11 | 0.61 |

**Supplementary Table B26b:** Skew surges (m) that reached or exceeded a 1 in 5 year return level at this site.

|  | **Date and time (GMT)** | **Return period (years)** | **Skew surge (m)** | **Water**  **level (m CD)** | **Astronomical tide (m CD)** |  |
| --- | --- | --- | --- | --- | --- | --- |
|  | 24/12/2013 23:15 | 17 | 0.79 | 4.74 | 3.94 |  |
|  | 10/01/1993 20:15 | 16 | 0.78 | 5.78 | 4.99 |  |
|  | 24/12/2013 10:30 | 10 | 0.76 | 5.02 | 4.26 |  |
|  | 01/02/2002 21:45 | 8 | 0.74 | 5.57 | 4.84 |  |
|  | 11/01/2005 19:45 | 7 | 0.73 | 5.48 | 4.75 |  |
| **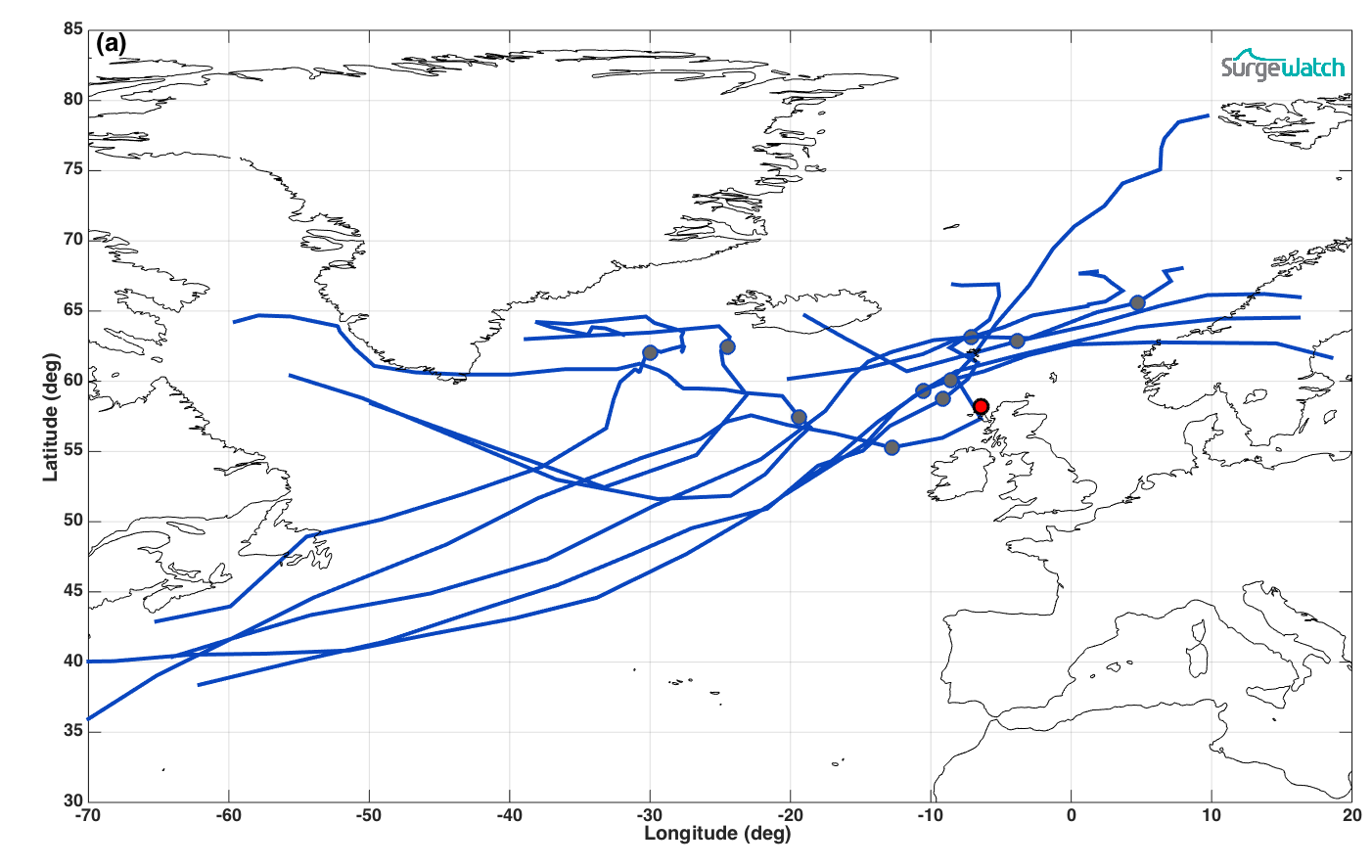**  **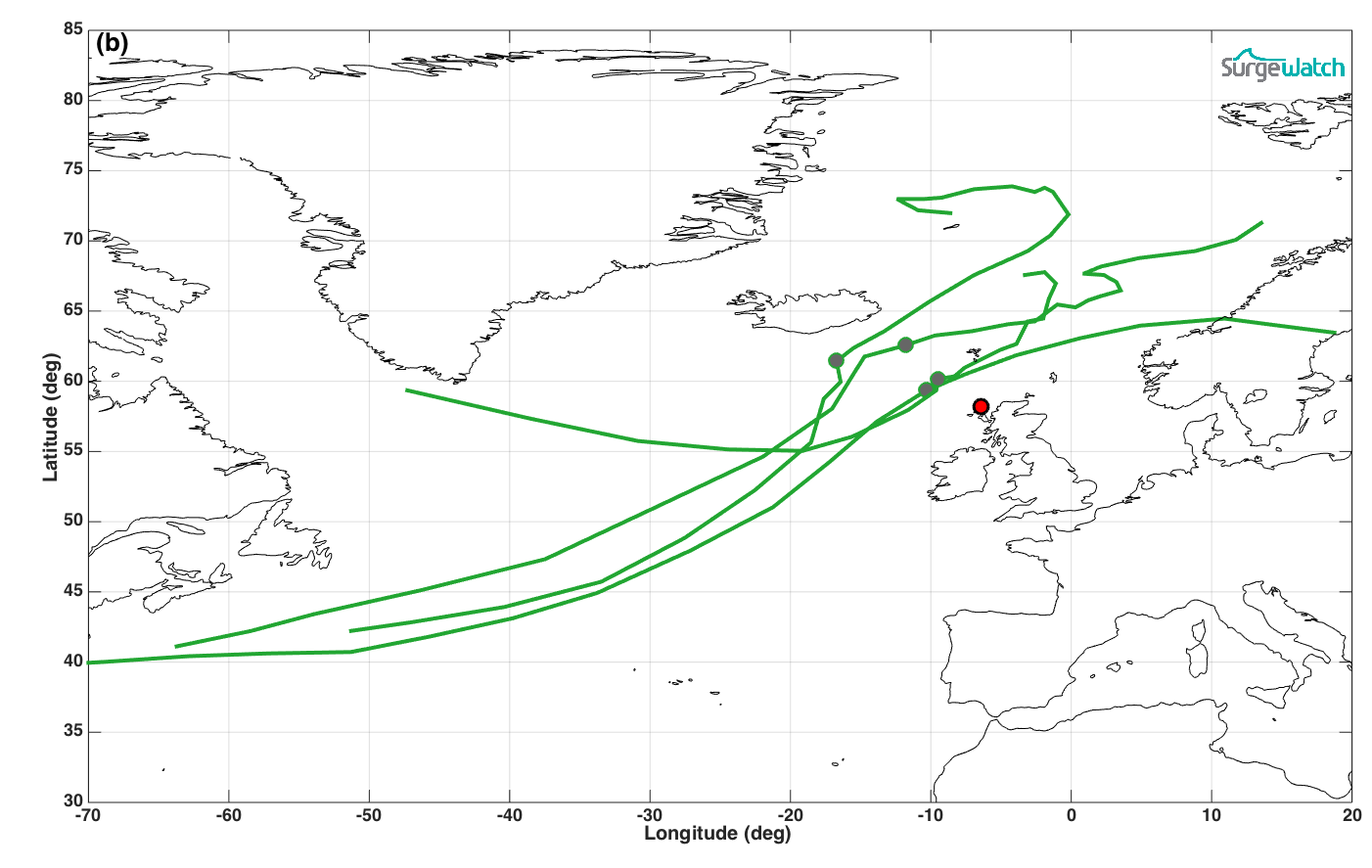** | | | | | | |
| **Supplementary Figure B26:** Tracks of the storms that generated (a) high water levels and (b) skew surges that reached or exceeded the 1 in 5 year return level at Stornoway (location shown with the blue dot). The red dot indicates the location of the storm centre at the time of highest water level or skew surge. | | | | | | |


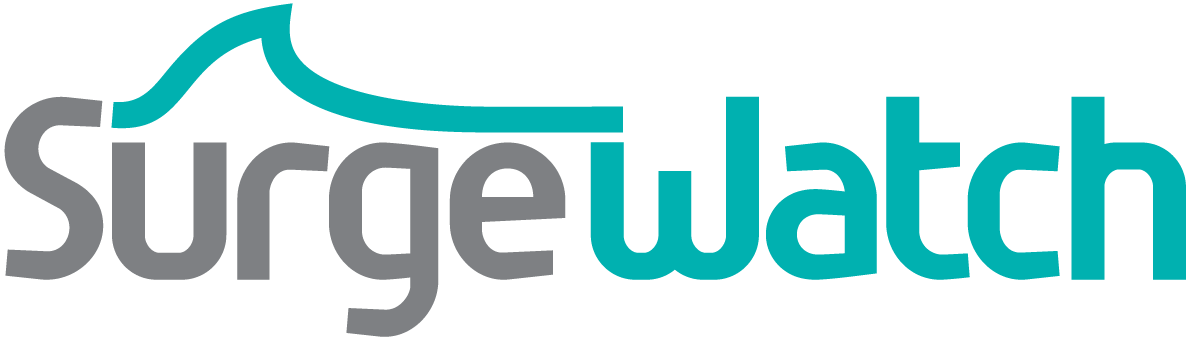
**LOCATION**

** B27.** **Ullapool**

*Chart Datum (CD) is 2.75m below Ordnance Datum Newlyn (ODN).*

| *Observational Period* |  | *Latitude & Longitude* |
| --- | --- | --- |
| ***1966-1968, 1970-1972, 1974-1983, 1985-present*** |  | ***57° 53′ 42.9″ N, 05° 09′ 29.0″ W*** |

**Supplementary Table B27a:** High water levels (m CD) that reached or exceeded a 1 in 5 year return level at this site.

| **Date and time (GMT)** | **Return period (years)** | **Water**  **level (m CD)** | **Astronomical tide (m CD)** | **Skew surge (m)** |
| --- | --- | --- | --- | --- |
| 12/01/2005 08:15 | 57 | 6.44 | 5.51 | 0.93 |
| 10/01/1993 20:45 | 20 | 6.30 | 5.47 | 0.80 |
| 24/12/1999 07:30 | 18 | 6.30 | 5.67 | 0.64 |
| 11/01/1993 08:30 | 15 | 6.26 | 5.69 | 0.57 |
| 18/01/1995 07:30 | 9 | 6.21 | 5.21 | 0.99 |
| 05/12/2013 07:15 | 9 | 6.25 | 5.44 | 0.61 |
| 03/01/2014 08:00 | 9 | 6.25 | 5.77 | 0.48 |
| 01/02/2014 07:45 | 9 | 6.25 | 5.80 | 0.45 |
| 12/01/1993 09:30 | 6 | 6.16 | 5.59 | 0.57 |
| 28/02/1967 09:00 | 6 | 6.10 | 5.48 | 0.62 |

**Supplementary Table B27b:** Skew surges (m) that reached or exceeded a 1 in 5 year return level at this site.

| **Date and time (GMT)** | **Return period (years)** | **Skew surge (m)** | **Water**  **level (m CD)** | **Astronomical tide (m CD)** |
| --- | --- | --- | --- | --- |
| 08/12/2011 17:30 | 179 | 1.16 | 5.84 | 4.67 |
| 18/01/1995 07:30 | 22 | 0.99 | 6.21 | 5.21 |
| 11/01/2005 20:15 | 13 | 0.94 | 6.08 | 5.11 |
| 12/01/2005 08:15 | 11 | 0.93 | 6.44 | 5.51 |
| 11/01/2007 12:00 | 9 | 0.91 | 5.21 | 4.30 |
| 25/12/2013 00:00 | 6 | 0.87 | 5.14 | 4.26 |
| 15/02/1995 07:00 | 6 | 0.87 | 5.94 | 5.06 |
| 26/10/1967 00:00 | 6 | 0.87 | 4.66 | 3.79 |
| 15/01/1993 12:15 | 5 | 0.86 | 5.58 | 4.72 |

| **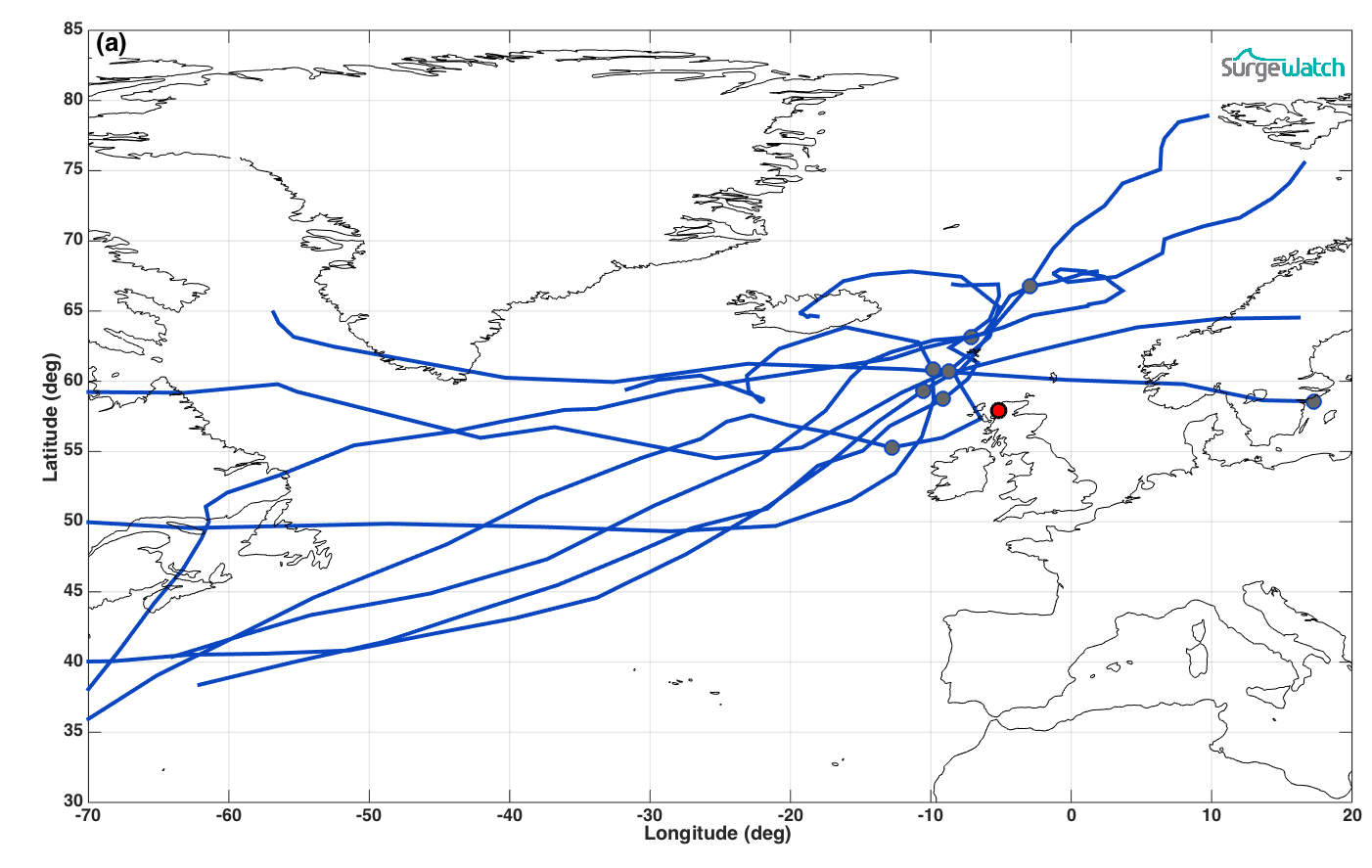**  **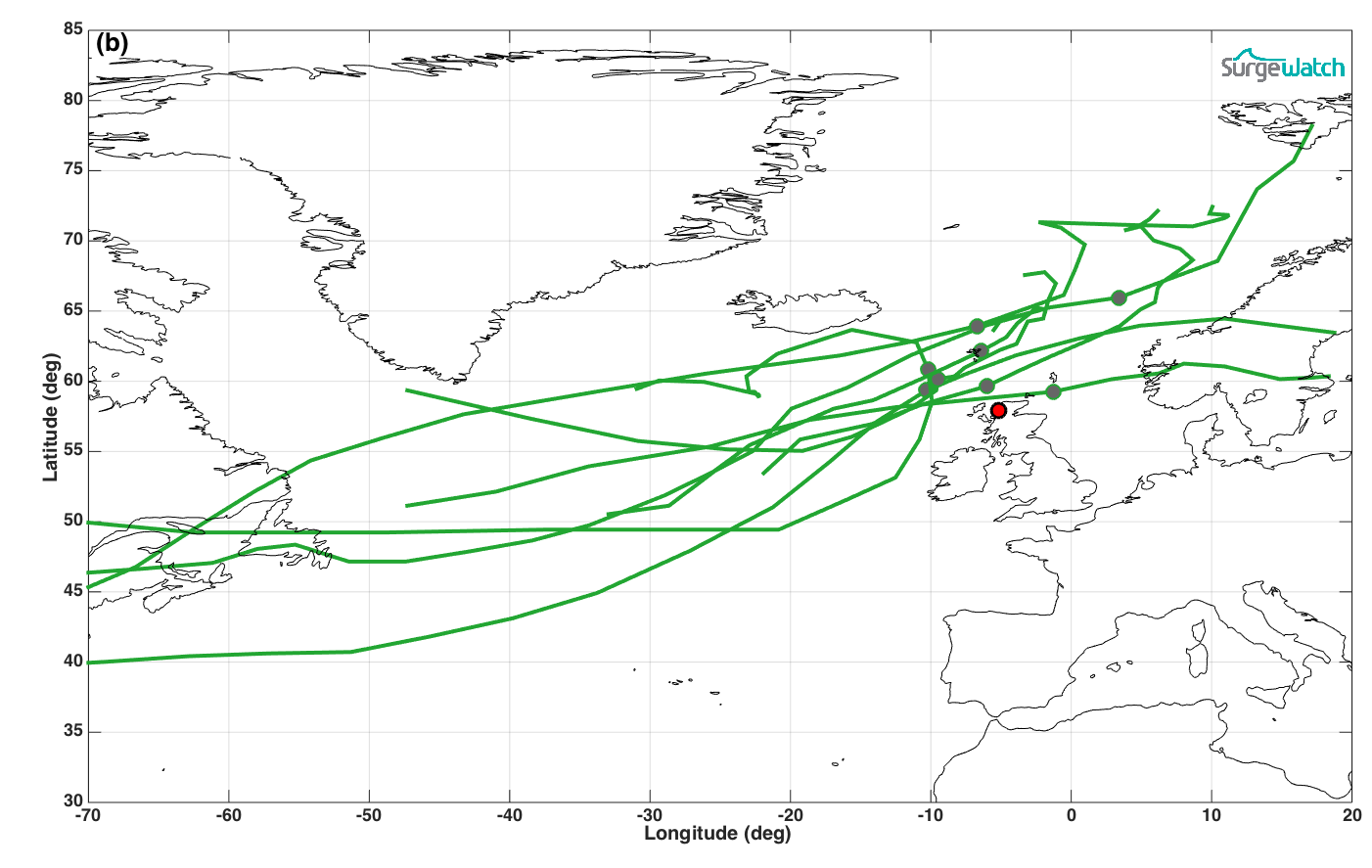** |
| --- |
| **Supplementary Figure B27:** Tracks of the storms that generated (a) high water levels and (b) skew surges that reached or exceeded the 1 in 5 year return level at Ullapool (location shown with the blue dot). The red dot indicates the location of the storm centre at the time of highest water level or skew surge. |


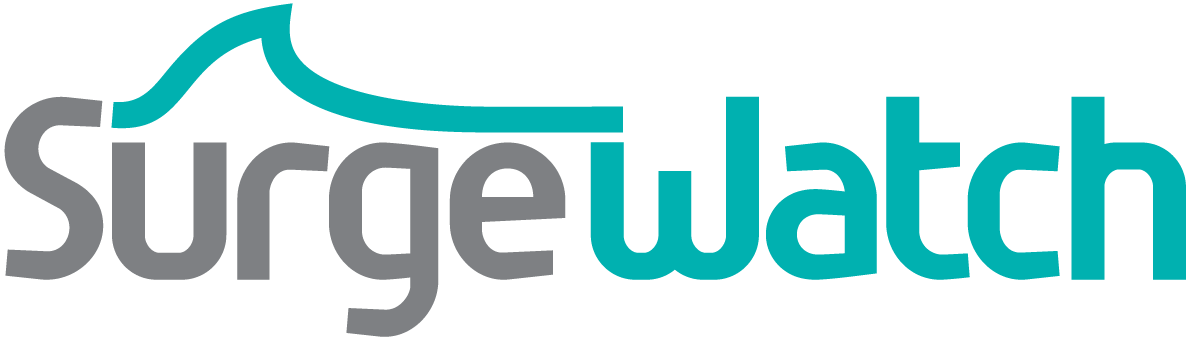
**LOCATION**

** B28.** **Kinlochbervie**

*Chart Datum (CD) is 2.5m below Ordnance Datum Newlyn (ODN).*

| *Observational Period* |  | *Latitude & Longitude* |
| --- | --- | --- |
| ***1991-2001, 2003-present*** |  | ***58° 27′ 24.1″ N, 05° 03′ 00.8″ W*** |

**Supplementary Table B28a:** High water levels (m CD) that reached or exceeded a 1 in 5 year return level at this site.

| **Date and time (GMT)** | **Return period (years)** | **Water**  **level (m CD)** | **Astronomical tide (m CD)** | **Skew surge (m)** |
| --- | --- | --- | --- | --- |
| 12/01/2005 08:30 | 70 | 6.28 | 5.21 | 1.06 |
| 24/12/1999 08:00 | 23 | 6.11 | 5.40 | 0.70 |
| 10/01/1993 21:00 | 20 | 6.07 | 5.12 | 0.93 |
| 11/01/1993 08:30 | 14 | 6.01 | 5.32 | 0.64 |
| 10/02/1997 09:00 | 10 | 5.98 | 5.51 | 0.47 |
| 09/02/1997 08:00 | 9 | 5.95 | 5.55 | 0.38 |
| 11/01/2005 20:15 | 8 | 5.96 | 4.86 | 1.09 |
| 18/01/1995 08:15 | 8 | 5.93 | 5.02 | 0.90 |
| 01/02/2014 08:15 | 5 | 5.93 | 5.44 | 0.47 |

**Supplementary Table B28b:** Skew surges (m) that reached or exceeded a 1 in 5 year return level at this site.

| **Date and time (GMT)** | **Return period (years)** | **Skew surge (m)** | **Water**  **level (m CD)** | **Astronomical tide (m CD)** |
| --- | --- | --- | --- | --- |
| 11/01/2005 20:15 | 20 | 1.09 | 5.96 | 4.86 |
| 12/01/2005 08:30 | 15 | 1.06 | 6.28 | 5.21 |
| 03/01/2000 05:30 | 12 | 1.04 | 5.17 | 4.12 |
| 02/03/1997 12:15 | 7 | 0.97 | 4.90 | 3.92 |

| **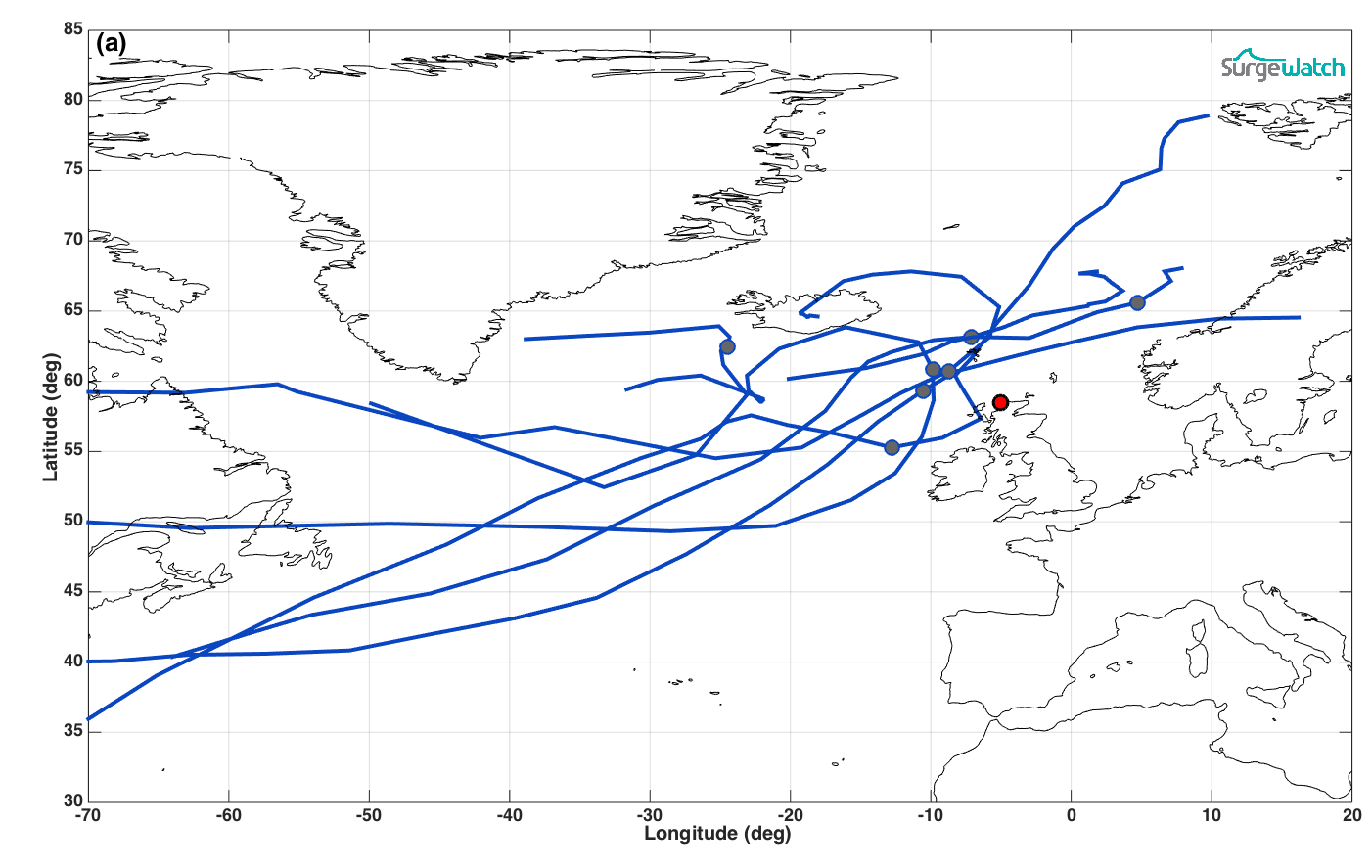**  **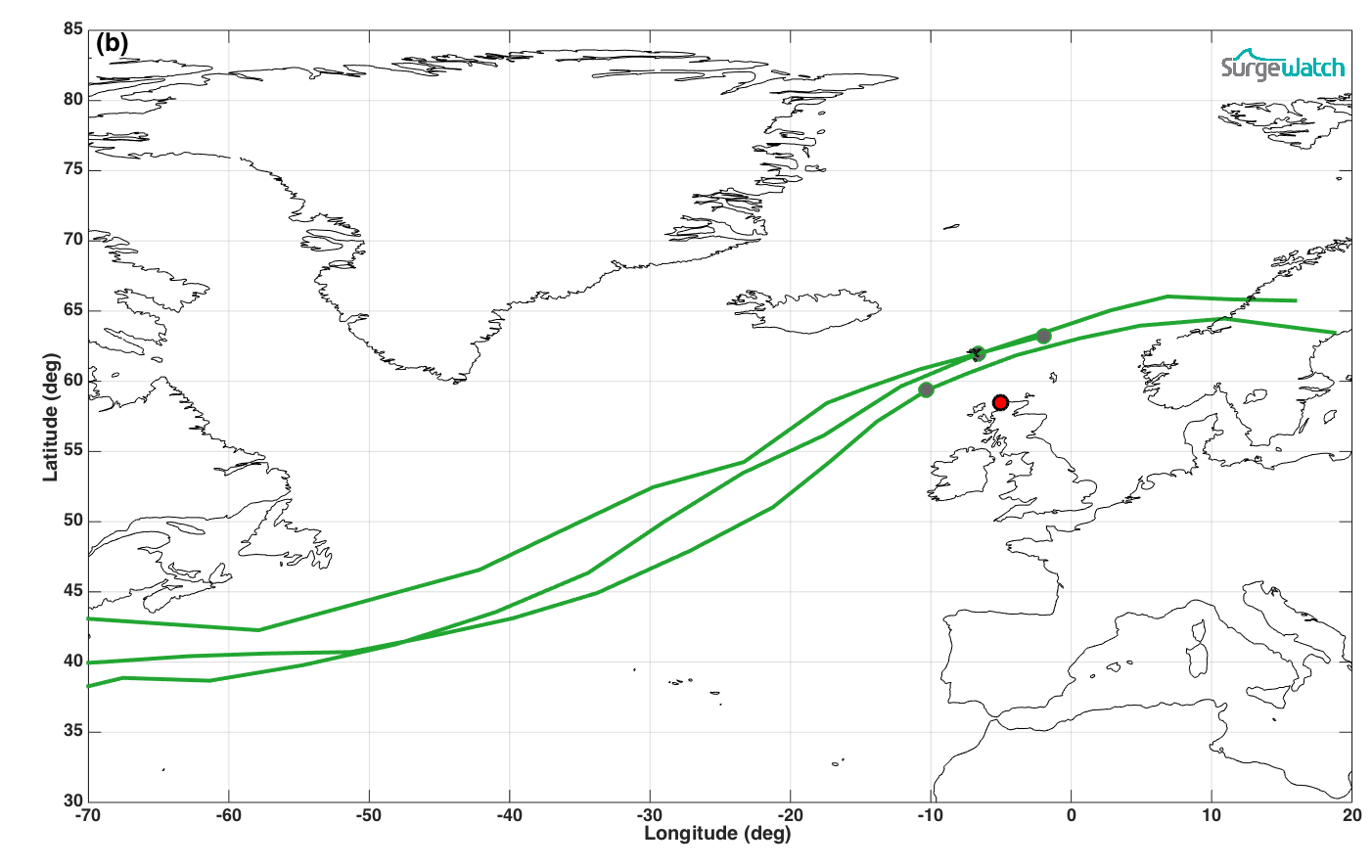** |
| --- |
| **Supplementary Figure B28:** Tracks of the storms that generated (a) high water levels and (b) skew surges that reached or exceeded the 1 in 5 year return level at Kinlochbervie (location shown with the blue dot). The red dot indicates the location of the storm centre at the time of highest water level or skew surge. |


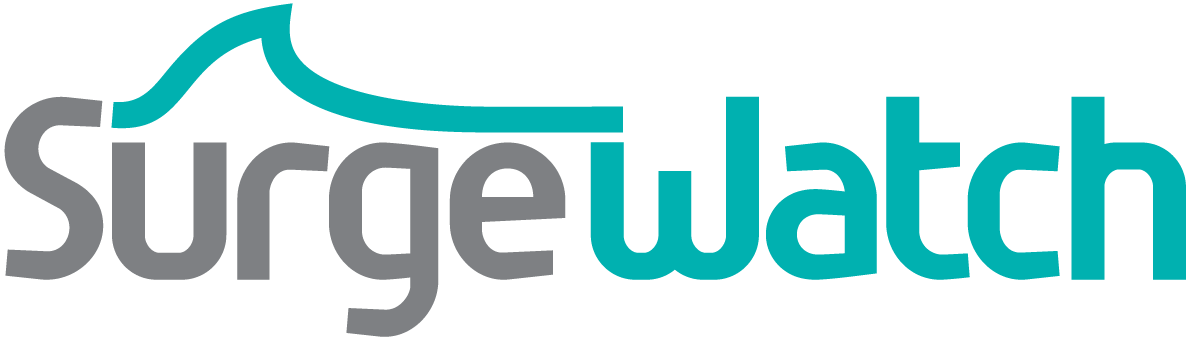
**LOCATION**

** B29.** **Lerwick**

*Chart Datum (CD) is 1.22m below Ordnance Datum Newlyn (ODN).*

| *Observational Period* |  | *Latitude & Longitude* |
| --- | --- | --- |
| ***1959-present*** |  | ***60° 09′ 14.5″ N, 01° 08′ 25.1″ W*** |

**Supplementary Table B29a:** High water levels (m CD) that reached or exceeded a 1 in 5 year return level at this site.

| **Date and time (GMT)** | **Return period (years)** | **Water**  **level (m CD)** | **Astronomical tide (m CD)** | **Skew surge (m)** |
| --- | --- | --- | --- | --- |
| 11/01/1993 12:30 | 89 | 3.04 | 2.49 | 0.55 |
| 11/01/1993 00:15 | 78 | 3.03 | 2.32 | 0.70 |
| 27/02/1990 12:00 | 15 | 2.93 | 2.54 | 0.39 |
| 12/01/1993 13:45 | 10 | 2.90 | 2.44 | 0.45 |
| 23/11/1984 23:00 | 9 | 2.90 | 2.53 | 0.38 |
| 02/02/2002 13:45 | 9 | 2.90 | 2.28 | 0.61 |
| 10/01/1993 12:15 | 7 | 2.88 | 2.47 | 0.40 |
| 09/01/1962 13:00 | 6 | 2.87 | 2.42 | 0.43 |
| 12/01/2005 12:00 | 5 | 2.86 | 2.39 | 0.47 |

**Supplementary Table B29b:** Skew surges (m) that reached or exceeded a 1 in 5 year return level at this site.

| **Date and time (GMT)** | **Return period (years)** | **Skew surge (m)** | **Water**  **level (m CD)** | **Astronomical tide (m CD)** |
| --- | --- | --- | --- | --- |
| 11/01/1993 00:15 | 495 | 0.70 | 3.03 | 2.32 |
| 02/02/2002 02:15 | 84 | 0.65 | 2.73 | 2.07 |
| 24/12/2013 14:45 | 34 | 0.62 | 2.65 | 2.02 |
| 02/02/2002 13:45 | 20 | 0.61 | 2.90 | 2.28 |
| 14/12/2011 01:00 | 13 | 0.59 | 2.57 | 1.98 |
| 06/03/1986 07:00 | 10 | 0.58 | 2.14 | 1.56 |
| 03/02/2002 03:00 | 9 | 0.58 | 2.52 | 1.94 |
| 03/12/2011 16:45 | 7 | 0.56 | 2.33 | 1.77 |
| 31/12/1987 21:00 | 6 | 0.56 | 2.33 | 1.77 |
| 15/01/1975 12:00 | 6 | 0.56 | 2.77 | 2.22 |
| 12/01/1993 01:30 | 5 | 0.55 | 2.85 | 2.29 |

| **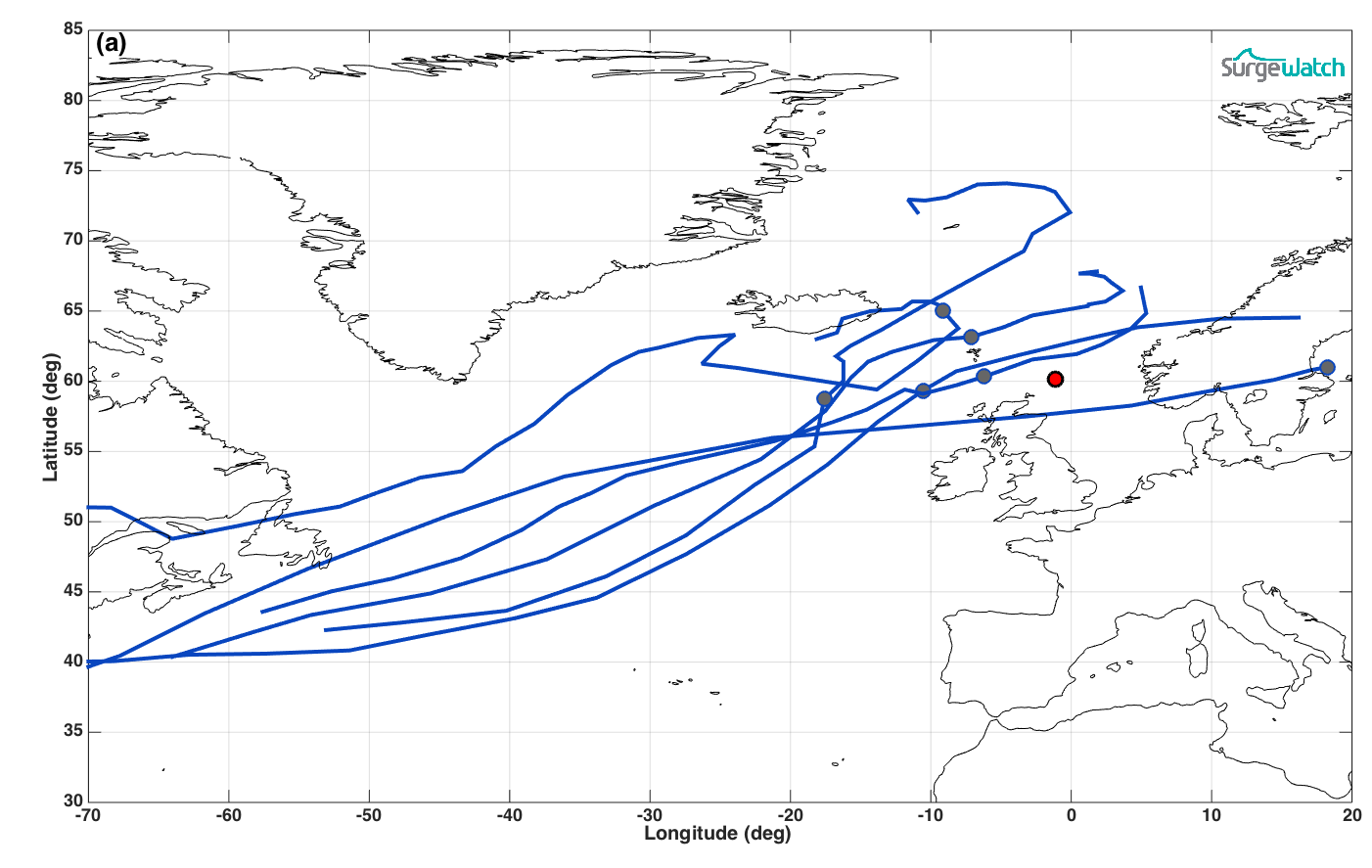**  **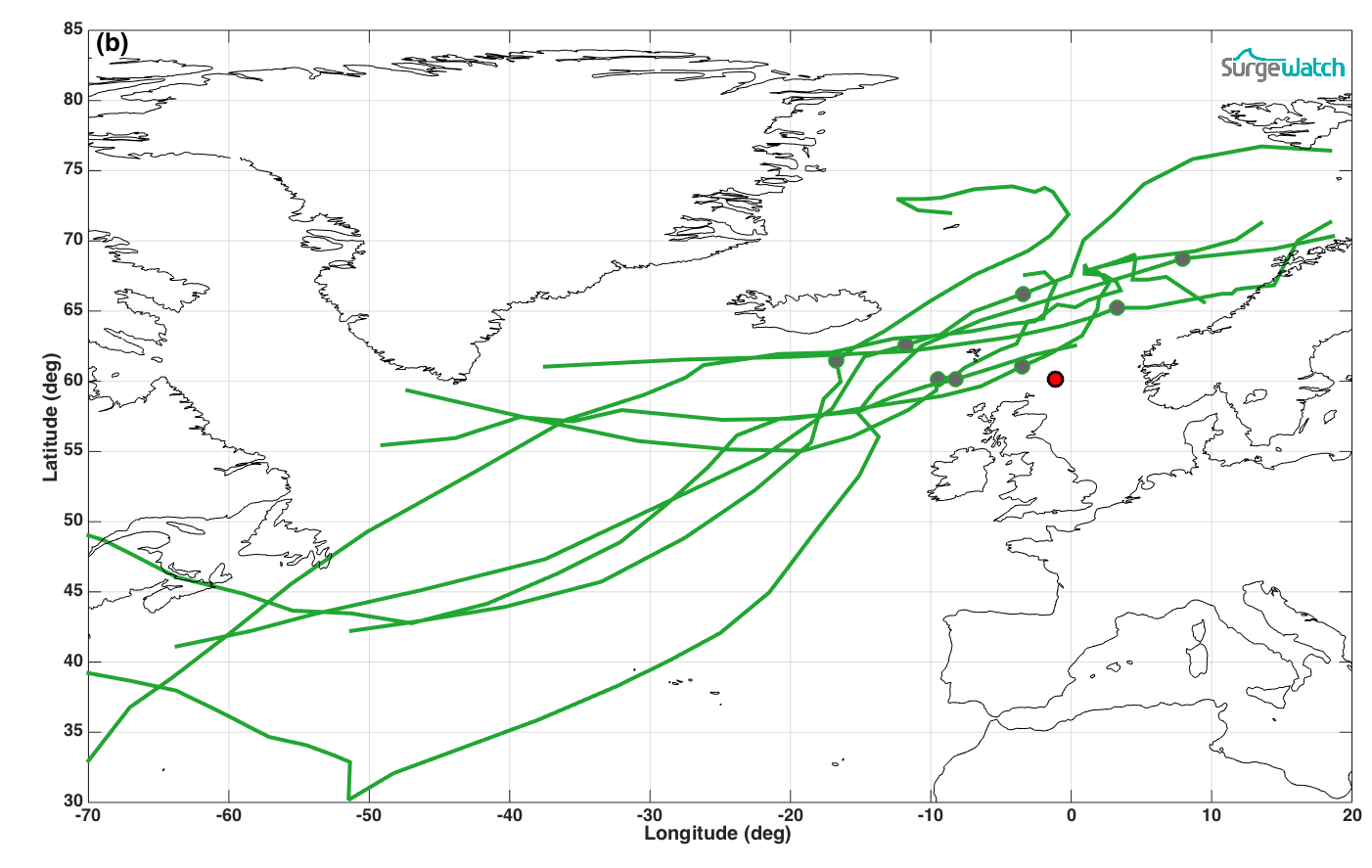** |
| --- |
| **Supplementary Figure B29:** Tracks of the storms that generated (a) high water levels and (b) skew surges that reached or exceeded the 1 in 5 year return level at X (location shown with the blue dot). The red dot indicates the location of the storm centre at the time of highest water level or skew surge. |


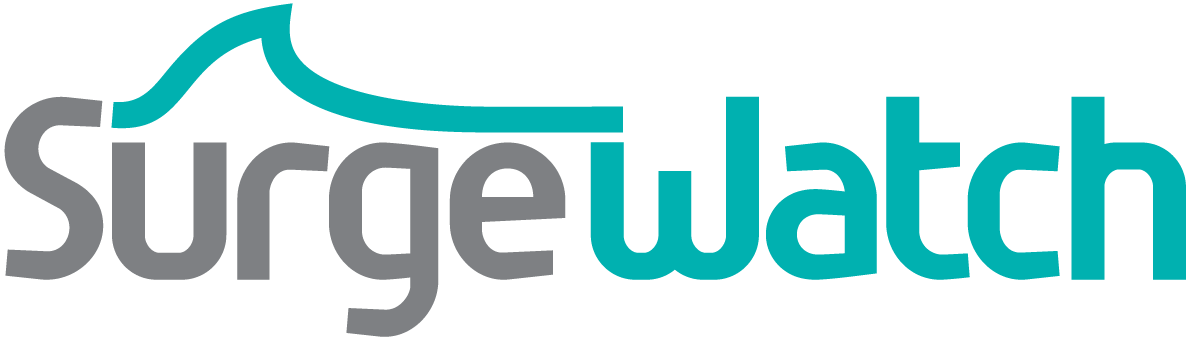
**LOCATION**

** B30.** **Wick**

*Chart Datum (CD) is 1.71m below Ordnance Datum Newlyn (ODN).*

| *Observational Period* |  | *Latitude & Longitude* |
| --- | --- | --- |
| ***1965-1970, 1972-present*** |  | ***58° 26′ 27.5″ N, 03° 05′ 11.0″ W*** |

**Supplementary Table B30a:** High water levels (m CD) that reached or exceeded a 1 in 5 year return level at this site.

| **Date and time (GMT)** | **Return period (years)** | **Water**  **level (m CD)** | **Astronomical tide (m CD)** | **Skew surge (m)** |
| --- | --- | --- | --- | --- |
| 12/01/2005 12:30 | 65 | 4.50 | 3.74 | 0.76 |
| 11/01/1993 00:45 | 32 | 4.42 | 3.64 | 0.77 |
| 25/12/1999 00:45 | 19 | 4.38 | 3.84 | 0.55 |
| 11/11/1977 11:00 | 13 | 4.31 | 3.72 | 0.59 |
| 11/01/1993 13:00 | 12 | 4.33 | 3.84 | 0.49 |
| 12/12/2000 23:45 | 12 | 4.34 | 3.69 | 0.63 |
| 03/12/2006 21:45 | 8 | 4.31 | 3.80 | 0.51 |
| 09/02/1997 12:30 | 8 | 4.29 | 3.96 | 0.33 |
| 02/01/1991 12:00 | 8 | 4.29 | 3.80 | 0.49 |
| 08/03/1981 12:00 | 7 | 4.27 | 3.73 | 0.51 |
| 27/02/1990 13:00 | 7 | 4.27 | 3.90 | 0.36 |
| 24/12/1999 12:15 | 6 | 4.28 | 3.90 | 0.38 |
| 02/02/2002 02:15 | 6 | 4.28 | 3.56 | 0.71 |
| 11/01/1974 13:00 | 6 | 4.24 | 3.89 | 0.33 |

**Supplementary Table B30b:** Skew surges (m) that reached or exceeded a 1 in 5 year return level at this site.

| **Date and time (GMT)** | **Return period (years)** | **Skew surge (m)** | **Water**  **level (m CD)** | **Astronomical tide (m CD)** |
| --- | --- | --- | --- | --- |
| 25/10/2008 21:00 | 64 | 0.82 | 4.06 | 3.23 |
| 11/01/1993 00:45 | 20 | 0.77 | 4.42 | 3.64 |
| 12/01/2005 12:30 | 17 | 0.76 | 4.50 | 3.74 |
| 01/01/1992 09:00 | 16 | 0.75 | 3.76 | 3.02 |
| 26/10/1967 04:00 | 12 | 0.73 | 3.51 | 2.77 |
| 13/12/2011 12:45 | 9 | 0.72 | 4.08 | 3.36 |
| 02/02/2002 02:15 | 8 | 0.71 | 4.28 | 3.56 |
| 05/01/1993 20:45 | 5 | 0.69 | 3.71 | 3.03 |

| **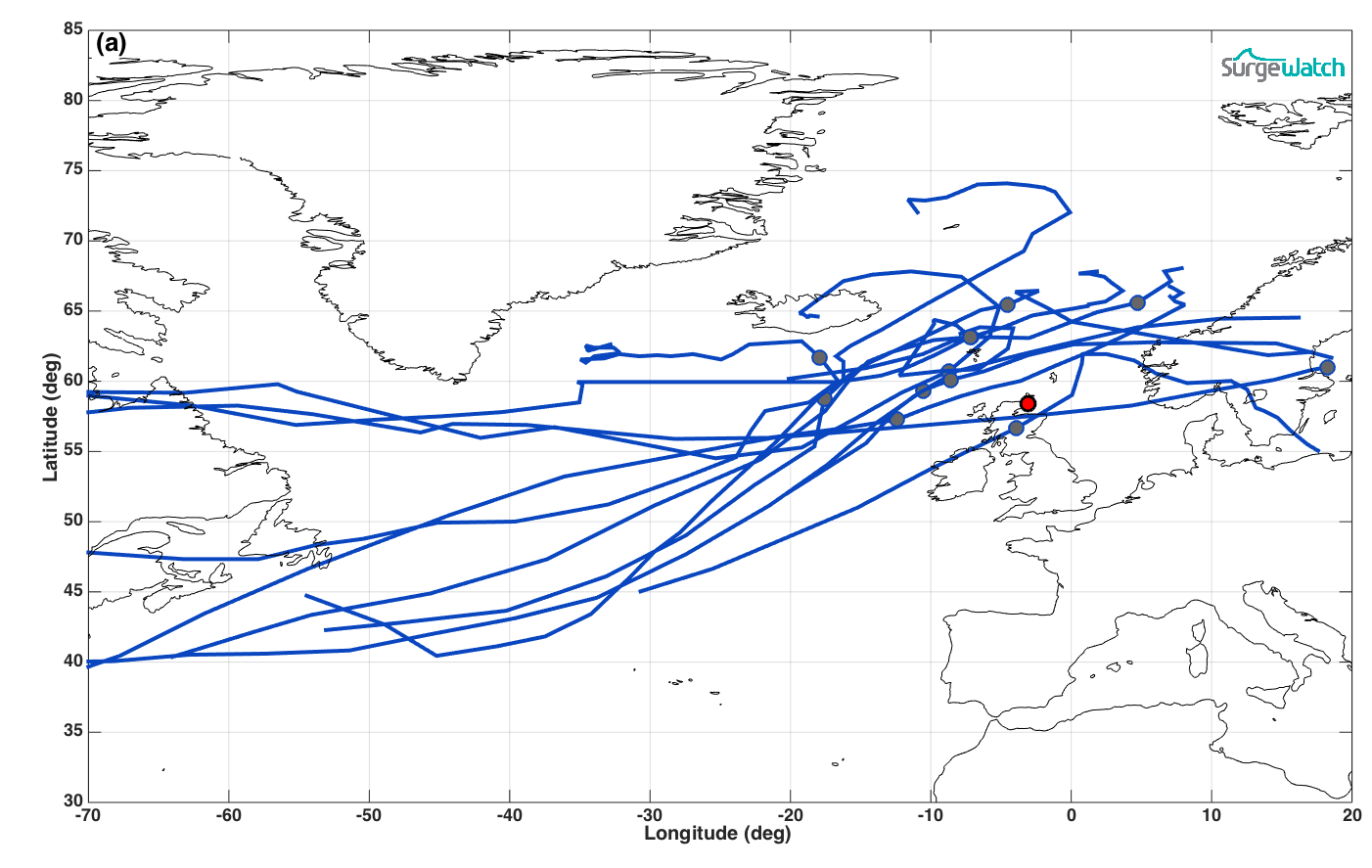** |
| --- |
| **Supplementary Figure B30:** Tracks of the storms that generated (a) high water levels and (b) skew surges that reached or exceeded the 1 in 5 year return level at Wick (location shown with the blue dot). The red dot indicates the location of the storm centre at the time of highest water level or skew surge. |

**LOCATION**

** B31.** **Aberdeen**

*Chart Datum (CD) is 2.25m below Ordnance Datum Newlyn (ODN).*

| *Observational Period* |  | *Latitude & Longitude* |
| --- | --- | --- |
| ***1930-present*** |  | ***57° 08′ 38.5″ N, 02° 04′ 48.8″ W*** |

**Supplementary Table B31a:** High water levels (m CD) that reached or exceeded a 1 in 5 year return level at this site.

| **Date and time (GMT)** | **Return period (years)** | **Water**  **level (m CD)** | **Astronomical tide (m CD)** | **Skew surge (m)** |
| --- | --- | --- | --- | --- |
| 12/01/2005 14:30 | 57 | 5.31 | 4.55 | 0.76 |
| 05/12/2013 15:00 | 20 | 5.23 | 4.61 | 0.60 |
| 11/01/1993 02:45 | 16 | 5.17 | 4.35 | 0.79 |
| 09/02/1997 14:15 | 10 | 5.14 | 4.73 | 0.40 |
| 28/02/1967 16:00 | 10 | 5.10 | 4.61 | 0.49 |
| 27/02/1990 15:00 | 10 | 5.13 | 4.71 | 0.42 |
| 29/09/1969 03:00 | 10 | 5.10 | 4.46 | 0.64 |
| 23/11/1984 01:00 | 9 | 5.11 | 4.71 | 0.41 |
| 27/11/2011 14:15 | 8 | 5.14 | 4.58 | 0.56 |
| 28/02/1967 04:00 | 8 | 5.07 | 4.36 | 0.71 |
| 01/02/1995 14:00 | 7 | 5.10 | 4.66 | 0.43 |
| 17/03/1957 14:00 | 6 | 5.03 | 4.73 | 0.29 |
| 11/02/1974 16:00 | 6 | 5.05 | 4.39 | 0.67 |

**Supplementary Table B31b:** Skew surges (m) that reached or exceeded a 1 in 5 year return level at this site.

| **Date and time (GMT)** | **Return period (years)** | **Skew surge (m)** | **Water**  **level (m CD)** | **Astronomical tide (m CD)** |
| --- | --- | --- | --- | --- |
| 15/01/1952 15:00 | 46 | 0.90 | 4.85 | 3.95 |
| 16/01/1993 07:15 | 37 | 0.89 | 4.52 | 3.62 |
| 25/10/2008 23:00 | 24 | 0.86 | 4.89 | 4.03 |
| 01/01/1992 11:00 | 22 | 0.85 | 4.58 | 3.73 |
| 17/01/1993 21:00 | 11 | 0.79 | 4.37 | 3.57 |
| 11/01/1993 02:45 | 10 | 0.79 | 5.17 | 4.35 |
| 05/01/1993 23:00 | 9 | 0.78 | 4.40 | 3.62 |
| 12/01/2005 14:30 | 8 | 0.76 | 5.31 | 4.55 |
| 27/10/1936 11:00 | 6 | 0.74 | 4.46 | 3.71 |

|  |
| --- |
| **Supplementary Figure B31:** Tracks of the storms that generated (a) high water levels and (b) skew surges that reached or exceeded the 1 in 5 year return level at Aberdeen (location shown with the blue dot). The red dot indicates the location of the storm centre at the time of highest water level or skew surge. |

**LOCATION**

** B32.** **Leith**

*Chart Datum (CD) is 2.9m below Ordnance Datum Newlyn (ODN).*

| *Observational Period* |  | *Latitude & Longitude* |
| --- | --- | --- |
| ***988-present*** |  | ***55° 59′ 23.4″ N, 03° 10′ 54.1″ W*** |

**Supplementary Table B32a:** High water levels (m CD) that reached or exceeded a 1 in 5 year return level at this site.

| **Date and time (GMT)** | **Return period (years)** | **Water**  **level (m CD)** | **Astronomical tide (m CD)** | **Skew surge (m)** |
| --- | --- | --- | --- | --- |
| 05/12/2013 15:15 | 21 | 6.61 | 5.74 | 0.70 |
| 09/02/1997 15:45 | 16 | 6.54 | 6.02 | 0.52 |
| 04/01/2014 16:15 | 10 | 6.52 | 5.95 | 0.51 |
| 11/01/1993 04:00 | 9 | 6.47 | 5.76 | 0.66 |
| 27/02/1990 16:00 | 6 | 6.42 | 5.99 | 0.43 |

**Supplementary Table B32b:** High water levels (m CD) that reached or exceeded a 1 in 5 year return level at this site.

| **Date and time (GMT)** | **Return period (years)** | **Skew surge (m)** | **Water**  **level (m CD)** | **Astronomical tide (m CD)** |
| --- | --- | --- | --- | --- |
| 16/01/1993 08:15 | 16 | 0.94 | 5.75 | 4.78 |
| 25/02/1997 16:15 | 12 | 0.90 | 6.23 | 5.31 |
| 01/01/1992 12:00 | 9 | 0.86 | 5.61 | 4.76 |

|  |
| --- |
| **Supplementary Figure B32:** Tracks of the storms that generated (a) high water levels and (b) skew surges that reached or exceeded the 1 in 5 year return level at Leigh (location shown with the blue dot). The red dot indicates the location of the storm centre at the time of highest water level or skew surge. |

**LOCATION**

** B33.** **North Shields**

*Chart Datum (CD) is 2.6m below Ordnance Datum Newlyn (ODN).*

| *Observational Period* |  | *Latitude & Longitude* |
| --- | --- | --- |
| ***1946-present*** |  | ***55° 00′ 26.8″ N, 01° 26′ 23.2″ W*** |

**Supplementary Table B33a:** High water levels (m CD) that reached or exceeded a 1 in 5 year return level at this site.

| **Date and time (GMT)** | **Return period (years)** | **Water**  **level (m CD)** | **Astronomical tide (m CD)** | **Skew surge (m)** |
| --- | --- | --- | --- | --- |
| 05/12/2013 16:15 | 405 | 6.58 | 5.42 | 1.08 |
| 31/01/1953 17:00 | 62 | 6.16 | 4.75 | 1.35 |
| 29/09/1969 05:00 | 29 | 6.10 | 5.25 | 0.85 |
| 12/01/2005 16:45 | 16 | 6.11 | 5.38 | 0.73 |
| 27/11/2011 16:30 | 9 | 6.05 | 5.31 | 0.74 |
| 01/02/1983 18:00 | 8 | 5.97 | 5.31 | 0.66 |
| 27/02/1990 17:00 | 7 | 5.97 | 5.57 | 0.39 |
| 09/02/1997 16:30 | 7 | 5.98 | 5.54 | 0.44 |

**Supplementary Table B33b:** Skew surges (m) that reached or exceeded a 1 in 5 year return level at this site.

| **Date and time (GMT)** | **Return period (years)** | **Skew surge (m)** | **Water**  **level (m CD)** | **Astronomical tide (m CD)** |
| --- | --- | --- | --- | --- |
| 31/01/1953 17:00 | 516 | 1.35 | 6.16 | 4.75 |
| 05/12/2013 16:15 | 51 | 1.08 | 6.58 | 5.42 |
| 01/03/2008 09:15 | 25 | 0.99 | 4.80 | 3.81 |
| 29/01/2000 22:30 | 14 | 0.92 | 5.08 | 4.13 |
| 16/02/1962 14:00 | 9 | 0.87 | 5.19 | 4.34 |
| 29/09/1969 05:00 | 7 | 0.85 | 6.10 | 5.25 |
| 01/03/1949 04:00 | 5 | 0.81 | 5.37 | 4.55 |

|  |
| --- |
| **Supplementary Figure B33:** Tracks of the storms that generated (a) high water levels and (b) skew surges that reached or exceeded the 1 in 5 year return level at North Shields (location shown with the blue dot). The red dot indicates the location of the storm centre at the time of highest water level or skew surge. |

**LOCATION**

** B34.** **Whitby**

*Chart Datum (CD) is 3.0m below Ordnance Datum Newlyn (ODN).*

| *Observational Period* |  | *Latitude & Longitude* |
| --- | --- | --- |
| ***1980-present*** |  | ***54° 29′ 24.0″ N, 00° 36′ 52.9″ W*** |

**Supplementary Table B34a:** High water levels (m CD) that reached or exceeded a 1 in 5 year return level at this site.

| **Date and time (GMT)** | **Return period (years)** | **Water**  **level (m CD)** | **Astronomical tide (m CD)** | **Skew surge (m)** |
| --- | --- | --- | --- | --- |
| 05/12/2013 17:15 | 568 | 7.32 | 6.08 | 1.24 |
| 01/02/1983 18:00 | 9 | 6.61 | 5.70 | 0.91 |

**Supplementary Table B34b:** Skew surges (m) that reached or exceeded a 1 in 5 year return level at this site.

| **Date and time (GMT)** | **Return period (years)** | **Skew surge (m)** | **Water**  **level (m CD)** | **Astronomical tide (m CD)** |
| --- | --- | --- | --- | --- |
| 05/12/2013 17:15 | 31 | 1.24 | 7.32 | 6.08 |
| 21/02/1993 03:45 | 17 | 1.13 | 6.23 | 5.07 |
| 12/12/1990 13:00 | 13 | 1.09 | 5.62 | 4.50 |
| 29/01/2000 22:45 | 10 | 1.06 | 5.64 | 4.57 |
| 01/03/2008 10:00 | 9 | 1.04 | 5.48 | 4.43 |
| 01/01/1992 14:00 | 8 | 1.00 | 5.68 | 4.67 |

|  |
| --- |
| **Supplementary Figure B34:** Tracks of the storms that generated (a) high water levels and (b) skew surges that reached or exceeded the 1 in 5 year return level at Whitby (location shown with the blue dot). The red dot indicates the location of the storm centre at the time of highest water level or skew surge. |

**LOCATION**

** B35.** **Immingham**

*Chart Datum (CD) is 3.90m below Ordnance Datum Newlyn (ODN).*

| *Observational Period* |  | *Latitude & Longitude* |
| --- | --- | --- |
| ***1953,1956-1958,1963-present*** |  | ***53° 37′ 49.5″ N, 00° 11′ 15.1″ W*** |

**Supplementary Table B35a:** High water levels (m CD) that reached or exceeded a 1 in 5 year return level at this site.

| **Date and time (GMT)** | **Return period (years)** | **Water**  **level (m CD)** | **Astronomical tide (m CD)** | **Skew surge (m)** |
| --- | --- | --- | --- | --- |
| 05/12/2013 19:15 | 787 | 9.12 | 7.50 | 1.62 |
| 01/02/1983 20:00 | 75 | 8.68 | 7.42 | 1.27 |
| 29/09/1969 07:00 | 43 | 8.56 | 7.34 | 1.16 |
| 11/01/1978 20:00 | 27 | 8.51 | 7.53 | 0.98 |
| 31/01/1953 19:00 | 21 | 8.42 | 6.84 | 1.58 |
| 03/01/1976 19:00 | 21 | 8.47 | 7.27 | 1.21 |
| 13/11/1977 07:00 | 7 | 8.29 | 7.56 | 0.73 |
| 09/02/1997 19:15 | 7 | 8.33 | 7.73 | 0.60 |
| 07/10/1990 07:00 | 6 | 8.31 | 7.62 | 0.69 |

**Supplementary Table B35b:** Skew surges (m) that reached or exceeded a 1 in 5 year return level at this site.

| **Date and time (GMT)** | **Return period (years)** | **Skew surge (m)** | **Water**  **level (m CD)** | **Astronomical tide (m CD)** |
| --- | --- | --- | --- | --- |
| 05/12/2013 19:15 | 75 | 1.62 | 9.12 | 7.50 |
| 31/01/1953 19:00 | 64 | 1.58 | 8.42 | 6.84 |
| 01/03/2008 11:00 | 37 | 1.45 | 6.82 | 5.31 |
| 01/02/1983 20:00 | 15 | 1.27 | 8.68 | 7.42 |
| 03/01/1976 19:00 | 11 | 1.21 | 8.47 | 7.27 |
| 18/01/1983 09:00 | 10 | 1.19 | 7.66 | 6.38 |
| 29/09/1969 07:00 | 9 | 1.16 | 8.56 | 7.34 |
| 21/02/1993 06:00 | 8 | 1.14 | 7.92 | 6.77 |
| 12/12/1990 02:00 | 8 | 1.13 | 7.26 | 6.14 |
| 21/11/1981 02:00 | 5 | 1.07 | 7.22 | 6.16 |

|  |
| --- |
| **Supplementary Figure B35:** Tracks of the storms that generated (a) high water levels and (b) skew surges that reached or exceeded the 1 in 5 year return level at Immingham (location shown with the blue dot). The red dot indicates the location of the storm centre at the time of highest water level or skew surge. |

**LOCATION**

** B36.** **Cromer**

*Chart Datum (CD) is 2.75m below Ordnance Datum Newlyn (ODN).*

| *Observational Period* |  | *Latitude & Longitude* |
| --- | --- | --- |
| ***1973,1974,1976,1988-present*** |  | ***52° 56′ 03.1″ N, 01° 18′ 05.9″ E*** |

**Supplementary Table B36a:** High water levels (m CD) that reached or exceeded a 1 in 5 year return level at this site.

| **Date and time (GMT)** | **Return period (years)** | **Water**  **level (m CD)** | **Astronomical tide (m CD)** | **Skew surge (m)** |
| --- | --- | --- | --- | --- |
| 21/02/1993 06:15 | 9 | 6.25 | 4.51 | 1.69 |
| 01/01/1995 18:00 | 6 | 6.17 | 5.03 | 1.13 |

**Supplementary Table B36b:** Skew surges (m) that reached or exceeded a 1 in 5 year return level at this site.

| **Date and time (GMT)** | **Return period (years)** | **Skew surge (m)** | **Water**  **level (m CD)** | **Astronomical tide (m CD)** |
| --- | --- | --- | --- | --- |
| 21/02/1993 06:15 | 61 | 1.69 | 6.25 | 4.51 |
| 30/01/2000 01:45 | 9 | 1.29 | 5.29 | 3.97 |
| 12/12/1990 15:00 | 6 | 1.20 | 5.08 | 3.88 |

|  |
| --- |
| **Supplementary Figure B36:** Tracks of the storms that generated (a) high water levels and (b) skew surges that reached or exceeded the 1 in 5 year return level at Cromer (location shown with the blue dot). The red dot indicates the location of the storm centre at the time of highest water level or skew surge. |

**LOCATION**

** B37.** **Lowestoft**

*Chart Datum (CD) is 1.5m below Ordnance Datum Newlyn (ODN).*

| *Observational Period* |  | *Latitude & Longitude* |
| --- | --- | --- |
| ***1964-present*** |  | ***52° 28′ 23.1″ N, 01° 45′ 00.9″ E*** |

**Supplementary Table B37:** High water levels (m CD) that reached or exceeded a 1 in 5 year return level at this site.

| **Date and time (GMT)** | **Return period (years)** | **Water**  **level (m CD)** | **Astronomical tide (m CD)** | **Skew surge (m)** |
| --- | --- | --- | --- | --- |
| 05/12/2013 22:30 | 188 | 4.76 | 2.79 | 1.93 |
| 29/09/1969 10:00 | 39 | 4.21 | 2.69 | 1.40 |
| 03/01/1976 21:00 | 33 | 4.18 | 2.32 | 1.66 |
| 01/02/1983 23:00 | 32 | 4.19 | 2.64 | 1.47 |
| 21/02/1993 09:00 | 27 | 4.18 | 2.32 | 1.77 |
| 09/11/2007 08:15 | 19 | 4.13 | 2.55 | 1.56 |
| 14/12/1973 00:00 | 14 | 3.97 | 2.70 | 1.27 |
| 28/01/1994 09:30 | 9 | 3.91 | 2.39 | 1.47 |
| 11/01/1978 23:00 | 7 | 3.83 | 2.66 | 1.18 |
| 01/01/1995 20:30 | 7 | 3.86 | 2.53 | 1.25 |
| 14/11/1993 21:00 | 6 | 3.83 | 2.56 | 1.21 |
| 21/11/1971 23:00 | 6 | 3.77 | 2.48 | 1.29 |
| 14/02/1989 06:00 | 6 | 3.81 | 1.94 | 1.41 |
| 29/10/1996 10:30 | 5 | 3.80 | 2.60 | 1.20 |

**Supplementary Table B37b:** Skew surges (m) that reached or exceeded a 1 in 5 year return level at this site.

| **Date and time (GMT)** | **Return period (years)** | **Skew surge (m)** | **Water**  **level (m CD)** | **Astronomical tide (m CD)** |
| --- | --- | --- | --- | --- |
| 05/12/2013 22:30 | 56 | 1.93 | 4.76 | 2.79 |
| 21/02/1993 09:00 | 29 | 1.77 | 4.18 | 2.32 |
| 03/01/1976 21:00 | 18 | 1.66 | 4.18 | 2.32 |
| 09/11/2007 08:15 | 11 | 1.56 | 4.13 | 2.55 |
| 01/03/2008 13:45 | 11 | 1.56 | 3.61 | 1.86 |
| 12/12/1990 19:00 | 10 | 1.54 | 3.74 | 2.19 |
| 01/02/1983 23:00 | 8 | 1.47 | 4.19 | 2.64 |
| 28/01/1994 09:30 | 8 | 1.47 | 3.91 | 2.39 |
| 30/01/2000 04:15 | 6 | 1.42 | 3.68 | 2.26 |
| 05/01/2012 18:15 | 6 | 1.41 | 3.64 | 2.19 |
| 14/02/1989 06:00 | 6 | 1.41 | 3.81 | 1.94 |
| 29/09/1969 10:00 | 5 | 1.40 | 4.21 | 2.69 |

|  |
| --- |
| **Supplementary Figure B37:** Tracks of the storms that generated (a) high water levels and (b) skew surges that reached or exceeded the 1 in 5 year return level at Lowestoft (location shown with the blue dot). The red dot indicates the location of the storm centre at the time of highest water level or skew surge. |

**LOCATION**

** B38.** **Harwich**

*Chart Datum (CD) is 2.02m below Ordnance Datum Newlyn (ODN).*

| *Observational Period* |  | *Latitude & Longitude* |
| --- | --- | --- |
| ***1954-1976, 2004-present*** |  | ***51° 56′ 52.8″ N, 01° 17′ 31.4″ E*** |

**Supplementary Table B38:** High water levels (m CD) that reached or exceeded a 1 in 5 year return level at this site.

| **Date and time (GMT)** | **Return period (years)** | **Water**  **level (m CD)** | **Astronomical tide (m CD)** | **Skew surge (m)** |
| --- | --- | --- | --- | --- |
| 05/10/1967 00:00 | 8 | 4.97 | 4.37 | 0.59 |
| 29/09/1969 14:00 | 8 | 4.96 | 4.13 | 0.83 |

**Supplementary Table B38:** Skew surges (m) that reached or exceeded a 1 in 5 year return level at this site.

| **Date and time (GMT)** | **Return period (years)** | **Skew surge (m)** | **Water**  **level (m CD)** | **Astronomical tide (m CD)** |
| --- | --- | --- | --- | --- |
| 22/12/1954 09:00 | 17 | 1.36 | 4.54 | 3.15 |
| 05/01/2012 19:45 | 10 | 1.24 | 4.54 | 3.08 |
| 01/11/2006 07:45 | 6 | 1.16 | 4.54 | 3.25 |

|  |
| --- |
| **Supplementary Figure B38:** Tracks of the storms that generated (a) high water levels and (b) skew surges that reached or exceeded the 1 in 5 year return level at Harwich (location shown with the blue dot). The red dot indicates the location of the storm centre at the time of highest water level or skew surge. |

**LOCATION**

** B39.** **Sheerness**

*Chart Datum (CD) is 2.91m below Ordnance Datum Newlyn (ODN).*

| *Observational Period* |  | *Latitude & Longitude* |
| --- | --- | --- |
| ***1976, 1994-present*** |  | ***49° 55′ 04.2″ N, 06° 19′ 01.7″ W*** |

**Supplementary Table B39a:** High water levels (m CD) that reached or exceeded a 1 in 5 year return level at this site.

| **Date and time (GMT)** | **Return period (years)** | **Water**  **level (m CD)** | **Astronomical tide (m CD)** | **Skew surge (m)** |
| --- | --- | --- | --- | --- |
| 10/12/1965 13:00 | 18 | 6.92 | 5.59 | 1.33 |
| 14/12/1973 03:00 | 9 | 6.80 | 5.72 | 1.08 |
| 14/12/1993 00:15 | 6 | 6.77 | 5.72 | 1.03 |
| 29/10/1996 13:30 | 6 | 6.76 | 5.76 | 0.87 |

**Supplementary Table B39b:** Skew surges (m) that reached or exceeded a 1 in 5 year return level at this site.

| **Date and time (GMT)** | **Return period (years)** | **Skew surge (m)** | **Water**  **level (m CD)** | **Astronomical tide (m CD)** |
| --- | --- | --- | --- | --- |
| 06/12/1973 21:00 | 16 | 1.41 | 6.30 | 4.89 |
| 10/12/1965 13:00 | 11 | 1.33 | 6.92 | 5.59 |
| 19/11/1973 20:00 | 7 | 1.23 | 6.30 | 5.06 |
| 14/02/1989 07:00 | 7 | 1.22 | 6.33 | 4.97 |
| 12/12/1990 21:00 | 7 | 1.22 | 6.04 | 4.83 |
| 12/12/1990 08:00 | 5 | 1.18 | 5.96 | 4.77 |

|  |
| --- |
| **Supplementary Figure B39:** Tracks of the storms that generated (a) high water levels and (b) skew surges that reached or exceeded the 1 in 5 year return level at Sheerness (location shown with the blue dot). The red dot indicates the location of the storm centre at the time of highest water level or skew surge. |

**LOCATION**

** B40.** **Dover**

*Chart Datum (CD) is 3.67m below Ordnance Datum Newlyn (ODN).*

| *Observational Period* |  | *Latitude & Longitude* |
| --- | --- | --- |
| ***1924,1926,1928,1930,1934-1936,1938, 1958-present*** |  | ***51° 06′ 51.8″ N, 01° 19′ 21.1″ E*** |

**Supplementary Table B40a:** High water levels (m CD) that reached or exceeded a 1 in 5 year return level at this site.

| **Date and time (GMT)** | **Return period (years)** | **Water**  **level (m CD)** | **Astronomical tide (m CD)** | **Skew surge (m)** |
| --- | --- | --- | --- | --- |
| 06/12/2013 00:45 | 843 | 8.45 | 6.81 | 1.64 |
| 02/02/1983 02:00 | 65 | 8.03 | 6.82 | 1.21 |
| 21/03/1961 02:00 | 38 | 7.90 | 6.81 | 1.10 |
| 01/01/1995 22:45 | 18 | 7.87 | 6.84 | 1.00 |
| 10/10/1926 12:00 | 17 | 7.72 | 6.69 | 0.86 |
| 04/01/1976 00:00 | 16 | 7.80 | 6.48 | 1.32 |
| 06/02/1924 00:00 | 15 | 7.69 | 6.75 | 0.94 |
| 14/11/1993 23:15 | 14 | 7.82 | 6.92 | 0.90 |
| 21/02/1993 11:00 | 14 | 7.81 | 6.27 | 1.50 |
| 07/10/1990 12:00 | 8 | 7.73 | 7.12 | 0.62 |
| 27/02/1990 00:00 | 8 | 7.73 | 7.16 | 0.56 |
| 29/09/1969 13:00 | 8 | 7.68 | 6.85 | 0.83 |
| 05/10/1967 12:00 | 6 | 7.65 | 7.24 | 0.41 |
| 13/11/1977 12:00 | 6 | 7.67 | 7.21 | 0.46 |
| 21/12/1926 00:00 | 6 | 7.57 | 6.63 | 0.94 |
| 28/01/1994 11:30 | 6 | 7.69 | 6.67 | 1.03 |
| 14/12/1973 02:00 | 6 | 7.65 | 6.69 | 0.98 |

**Supplementary Table B40b:** Skew surges (m) that reached or exceeded a 1 in 5 year return level at this site.

| **Date and time (GMT)** | **Return period (years)** | **Skew surge (m)** | **Water**  **level (m CD)** | **Astronomical tide (m CD)** |
| --- | --- | --- | --- | --- |
| 06/12/2013 00:45 | 103 | 1.64 | 8.45 | 6.81 |
| 21/02/1993 11:00 | 52 | 1.50 | 7.81 | 6.27 |
| 04/01/1976 00:00 | 20 | 1.32 | 7.80 | 6.48 |
| 16/02/1962 22:00 | 20 | 1.31 | 7.23 | 5.92 |
| 02/02/1983 02:00 | 12 | 1.21 | 8.03 | 6.82 |
| 05/01/2012 20:45 | 11 | 1.20 | 6.70 | 5.50 |
| 09/11/2007 10:30 | 8 | 1.13 | 7.69 | 6.55 |
| 01/03/2008 17:00 | 7 | 1.11 | 6.00 | 4.86 |
| 21/03/1961 02:00 | 6 | 1.10 | 7.90 | 6.81 |
| 19/11/1973 19:00 | 6 | 1.08 | 6.71 | 5.63 |
| 03/01/1976 12:00 | 5 | 1.06 | 7.52 | 6.46 |

|  |
| --- |
| **Supplementary Figure B40:** Tracks of the storms that generated (a) high water levels and (b) skew surges that reached or exceeded the 1 in 5 year return level at Dover (location shown with the blue dot). The red dot indicates the location of the storm centre at the time of highest water level or skew surge. |
